# Dissecting molecular network structures using a network subgraph approach

**DOI:** 10.7717/peerj.9556

**Published:** 2020-08-06

**Authors:** Chien-Hung Huang, Efendi Zaenudin, Jeffrey J.P. Tsai, Nilubon Kurubanjerdjit, Eskezeia Y. Dessie, Ka-Lok Ng

**Affiliations:** 1 Department of Computer Science and Information Engineering, National Formosa University, Yunlin, Taiwan; 2 Department of Bioinformatics and Medical Engineering, Asia University, Taichung, Taiwan; 3 Research Center for Informatics, Indonesian Institute of Sciences, Bandung, Indonesia; 4 School of Information Technology, Mae Fah Luang University, Chiang Rai, Thailand; 5 Department of Medical Research, China Medical University Hospital, China Medical University, Taichung, Taiwan

**Keywords:** Network motifs, Biological networks, Graph theory, Information theory, Network complexity, Entropy, Network subgraphs

## Abstract

Biological processes are based on molecular networks, which exhibit biological functions through interactions of genetic elements or proteins. This study presents a graph-based method to characterize molecular networks by decomposing the networks into directed multigraphs: network subgraphs. Spectral graph theory, reciprocity and complexity measures were used to quantify the network subgraphs. Graph energy, reciprocity and cyclomatic complexity can optimally specify network subgraphs with some degree of degeneracy. Seventy-one molecular networks were analyzed from three network types: cancer networks, signal transduction networks, and cellular processes. Molecular networks are built from a finite number of subgraph patterns and subgraphs with large graph energies are not present, which implies a graph energy cutoff. In addition, certain subgraph patterns are absent from the three network types. Thus, the Shannon entropy of the subgraph frequency distribution is not maximal. Furthermore, frequently-observed subgraphs are irreducible graphs. These novel findings warrant further investigation and may lead to important applications. Finally, we observed that cancer-related cellular processes are enriched with subgraph-associated driver genes. Our study provides a systematic approach for dissecting biological networks and supports the conclusion that there are organizational principles underlying molecular networks.

## Introduction

Molecular networks are the basis of biological processes, with biological functions emerging through interactions among the various genetic components. In network analysis, we often use hierarchical decomposition approaches (Freyre-González et al., 2008; Jothi et al., 2009) to simplify network complexity. Decomposition approaches assume that a complex network can be decomposed into small modules, providing explanations that can address increasing complexity. Based on this assumption, a network can be modeled by a collection of smaller modules known as network motifs. Each module is expected to perform specific functions, and is separable from the functions of other modules (Hartwell et al., 1999; Lauffenburger, 2000; Shen-Orr et al., 2002). Network motifs are modules (i.e., directed graphs with feedback interactions) embedded in a network that occur significantly more often than a randomized version of the network. These motifs show interesting dynamic behaviors, and cooperative effects between the motif components play a critical role in human diseases.

We classify network-based analysis into the following major categories: (1) motif identification and analysis, (2) global architecture study, (3) local topological properties and (4) network robustness under different perturbations. For the first category, the aim of the analysis is to develop an algorithm to detect network motifs within a large network by using the random network approach. There are several publicly available network motif detection tools, including MFINDER (Milo et al., 2002), MAVISTO (Schreiber & Schwöbbermeyer, 2005), FANMOD (Wernicke & Rasche, 2006), NetMatch (Ferro et al., 2007) and SNAVI (Ma’ayan, 2009). Tran et al. (2015) reported that MFINDER, MAVISTO and FANMOD are able to identify network motifs with size of 8 in reasonable running time. In our previous work, we observed that motif finding tools might have certain limitations. These limitations are: (1) not every motif identification tool is able to identify motifs associated with a significant *p*-value. Therefore, the tools cannot enumerate all possible network substructures; (2) the tools are not designed to identify motif structures within a motif; and (3) the tools do not report node identity, which does not identify the genetic elements embedded within a motif.

For the second category, many studies employed random graph theory to characterize the global structure of molecular networks. These analyses can determine whether a network is assortative or has small-world properties (Jeong et al., 2000; Lee, Kim & Jeong, 2011). For example, protein–protein interaction networks are scale-free or described by hierarchical network models (Lee et al., 2005). Instead of examining networks from a global perspective, the present study adopted a bottom-up approach, dissecting a network into local structures (direct subgraphs). The local approach has certain features; such as, source and target node identity information, and degree of feedback interaction between nodes, that are not explicit in the global description. Additionally, we do not use randomized networks in our analysis.

The aim of the third category is to use topological graph theory characterizes networks by computing topological parameters, such as betweenness centrality, closeness centrality, clustering coefficients, and eigenvector centrality (Bloch, Jackson & Tebaldi, 2016; Bonacich & Lloyd, 2001; Konganti et al., 2013; Pavlopoulos et al., 2011). Most previous studies have used graph metrics to analyze network topology, leaving a very relevant question unanswered: do these topological parameters convey enough information about the networks? The answer seems to be negative. For instance, closeness centrality of a node is determined by the shortest path from the node to other nodes in the network. Eigenvector centrality of a node is based on the importance of nodes. Little is known about the architectures or organizational principles of molecular networks based on the modular decomposition approach. For instance, can we have a unique label for different motifs? Do certain modular patterns occur in a network with unequal probability? These issues are addressed in the present study.

The aim of the last category is to examine whether molecular networks are robust under different types of perturbation. The work of Albert, Jeong & Barabasi (2000) considered four types of perturbations: random edge deletion, node deletion, edge rewiring and hub nodes removal. It was shown that molecular networks are robust under random perturbation but fragile under attack perturbation. In our previous work, we extended previous studies by demonstrating that molecular networks are also fragile under degree-based, betweenness-based and brokering coefficient-based perturbations (Huang, Chen & Ng, 2016).

Besides network motif description, Przulj (2007) and Yaveroğlu et al. (2014) utilized a graphlet-based approach to examine the network comparison problem. Directed graphlets are superior for comparing directed networks (Martin et al., 2017; Trpevski et al., 2016) and they are effective for studying brain networks (Sarajlić et al., 2016). Our study focused on networks composed of regulatory interactions (directed graphs), such as gene regulation networks and signal transduction networks, but not protein–protein interaction networks (undirected graphs).

Our goal was to apply rigorous mathematical approaches to characterize core network components—the so-called network subgraphs. Furthermore, by decomposing networks into subgraphs, we attempted to discover the underlying architectures and organizational principles of molecular networks. To demonstrate the effectiveness of the subgraph approach, we computed the odds ratio to quantify the level of enrichment of subgraph-associated driver genes embedded in molecular networks; and then studied their implication in cancer formation and invasion. We note that the present study does not aim to develop efficient algorithm to identify network subgraphs.

### Network subgraphs (N-node subgraphs) vs. network motifs

In this study, we hypothesized that network subgraphs are the fundamental building blocks of a network. In other words, subgraphs are treated as the core network components. This is similar to the work of Mowshowitz (Mowshowitz, 1968a), who proposed that a finite graph (*V* vertices and *E* edges) can be decomposed into equivalence classes (*h* classes). Each class contains *v*_i_ vertices and a probability is assigned to each class; that is, *p*_i_ = *v*_i_/*V*. The Shannon entropy associated with the graph can be viewed as a measure of the graph complexity relative to the given decomposition of its equivalence classes. Therefore, we propose that network properties are captured by subgraphs comprising *N* nodes, which are referred to as *N*-node subgraphs. To systematically characterize a large network, the 3-node subgraphs, 4-node subgraphs, and up to the *N*-node subgraphs embedded in the network are identified. These subgraph patterns are identical to the network motifs defined by (Alon, 2006).

We identify network subgraphs embedded within a molecular network by matching the network topology to the *N*-node subgraphs. However, we do not consider randomized versions of the studied network. Therefore, one question is whether the *N*-node subgraphs results differ from the *N*-node motif findings for a given network–that is, does the number of identified subgraph patterns match the number of motif patterns? We determined that the number of subgraph patterns identified in a network is the same as the number of motif patterns.

For a directed graph, a total of 2, 13, 199, 9,364 and 1,530,843 possible patterns can be defined for 2-node, 3-node, 4-node, 5-node and 6-node motifs, respectively (Harary & Palmer, 1973; Sloane & Plouffe, 1995). These sets of *N*-nodes motifs were used as our sets of *N*-node subgraphs. Since the problem of identifying *N*-node subgraphs in a large network is NP-complete (Kim, Diko & Rawson, 2013) we used 3-node and 4-node subgraphs only. Subgraphs composed of five or more nodes are neglected as a first approximation, which could provide useful insights into dissecting the design principles underlying molecular networks. Subgraphs composed of five nodes are currently under study.

An earlier work (Konagurthu & Lesk, 2008) showed that certain motifs do not appear significantly more frequently than motifs appearing in corresponding random networks. Nevertheless, those motifs still play functional roles. This justifies our approach because the present work identifies all possible 3-node and 4-node subgraphs, regardless of their occurrence frequency. In other words, we adopt the notion that *N*-node subgraphs are the basic building blocks, but do not necessarily occur frequently in a network.

Adami et al. (2011) studied undirected colored graphs (where nodes are labeled with different colors) and showed that the relative frequency of the colored motifs can be used to define the information content of the network. Here, we consider subgraphs that are *directed* graphs which could contain cycles.

### Spectral graph theory, reciprocity, complexity measures and information theory

Seminal works regarding the concepts of information content, topology and entropy in biology were carried out by Mowshowitz and Rashevsky (Mowshowitz, 1968a; Rashevsky, 1954, 1955). In particular, Mowshowitz presented an entropy-based method to measure the complexity of a graph by decomposing it into equivalence classes (Mowshowitz, 1968a). To characterize network subgraphs, we utilized the following concepts: spectral graph theory (SGT), reciprocity, and complexity measures. SGT is a powerful approach that has been applied in many areas, including computer science and computational biology (Banerjee & Jost, 2009; Cvetković & Simić, 2011). The energy of a graph is an invariant (Gutman, 1992, 2001; Li, Shi & Gutman, 2012) equal to the sum of the absolute values of the eigenvalues of the adjacency matrix *A*. Originally, the concept of graph energy introduced by Gutman was applied to study undirected graphs, and was used to estimate the 𝜋–electron energy of hydrocarbons (Gutman, 1992).

The eigenvalues of the adjacency matrix *A* defined on a graph play an essential role in inferring the structural properties of the chemical graphs (Estrada & Benzi, 2017). Estrada and Benzi showed that the graph energy is a weighted sum of the traces of even powers of the adjacency matrix. Indeed, this finding can be used to obtain energy bounds for hydrocarbon molecules. The graph energy concept was extended from undirected graphs to digraphs by Brualdi (2010).

Molecular networks are directed graphs composed of feedback interactions. Reciprocity is a parameter that quantifies the degree of bidirectional connection of a network subgraph. Complexity arises from interactions among the constituent components. Many complexity measures have been proposed, but there is no standard or formal definition of complexity metrics that can be applied in all circumstances. Each complexity measure has strengths and weaknesses (Weyuker, 1988). Early work on defining complexity for directed graphs and infinite graphs can be traced back to Mowshowitz (1968b). The concept of graph complexity indices has been applied to infer the hierarchical order of chemical structures (Minoli, 1976). Given a network subgraph pattern, we used two commonly used complexity measures to characterize subgraphs.

It is possible that some network subgraphs are associated with the same graph energy and spectrum (the set of eigenvalues). In other words, different subgraphs with the same energy and spectrum are indistinguishable; that is, the cospectrality problem. Wilson & Zhu (2008) proposed to combine the spectra of two of the four graph matrices (the adjacency, Laplacian, signless Laplacian, and normalized Laplacian matrices) to reduce the cospectrality problem for undirected graphs and trees. Their method can effectively reduce the number of cospectral pairs of graphs, although the pairs are not completely distinguishable.

In addition, graph descriptors are a useful concept to classify complex networks (Mueller et al., 2011) In this study, we used a greedy algorithm to search for optimal parameters that maximize degenerate subgraph removal. The parameters we suggested include the subgraph spectrum, graph energy, reciprocity, and complexity measures.

The information-theoretic quantity, Shannon entropy, was proposed by Cover & Thomas (1991) and Shannon (1948) to provide a precise definition of information randomness. Lower Shannon entropy indicates less information or more structure embedded within the data (i.e., a biased system). Shannon entropy has been extensively applied in cancer biology studies. For instance, a previous study reported a negative correlation between cancer protein–protein interaction network entropy and cancer aggressiveness (Conforte et al., 2019). In a recent study, Sen et al. (2019) studied the protein family sequences of four cancer types, and observed that the sequences tend to associate with higher Shannon entropy. Higher entropy implies a larger bias distribution of amino acid composition and protein structure disorder. Additionally, cancer networks exhibit high information entropy (Schramm, Kannabiran & König, 2010), increased network entropy (West et al., 2012), and signaling entropy (Teschendorff et al., 2015). Being motivated by the effectiveness of Shannon entropy in cancer studies, we used that quantity to measure the randomness of subgraphs frequency distributions of molecular networks.

In our previous work (Hsieh et al., 2015), we laid a foundation for the present study. In another recent work (Lee, Huang & Ng, 2016) we extended the previous work (Hsieh et al., 2015) by developing a subgraph-finding algorithm, *PatternFinder*, to identify 3-node and 4-node subgraphs in cancer networks, signal transduction networks, and cellular processes. Since the network size considered in this study is less than 160 nodes, computational time is not an issue.

## Methods

**Figure 1:**
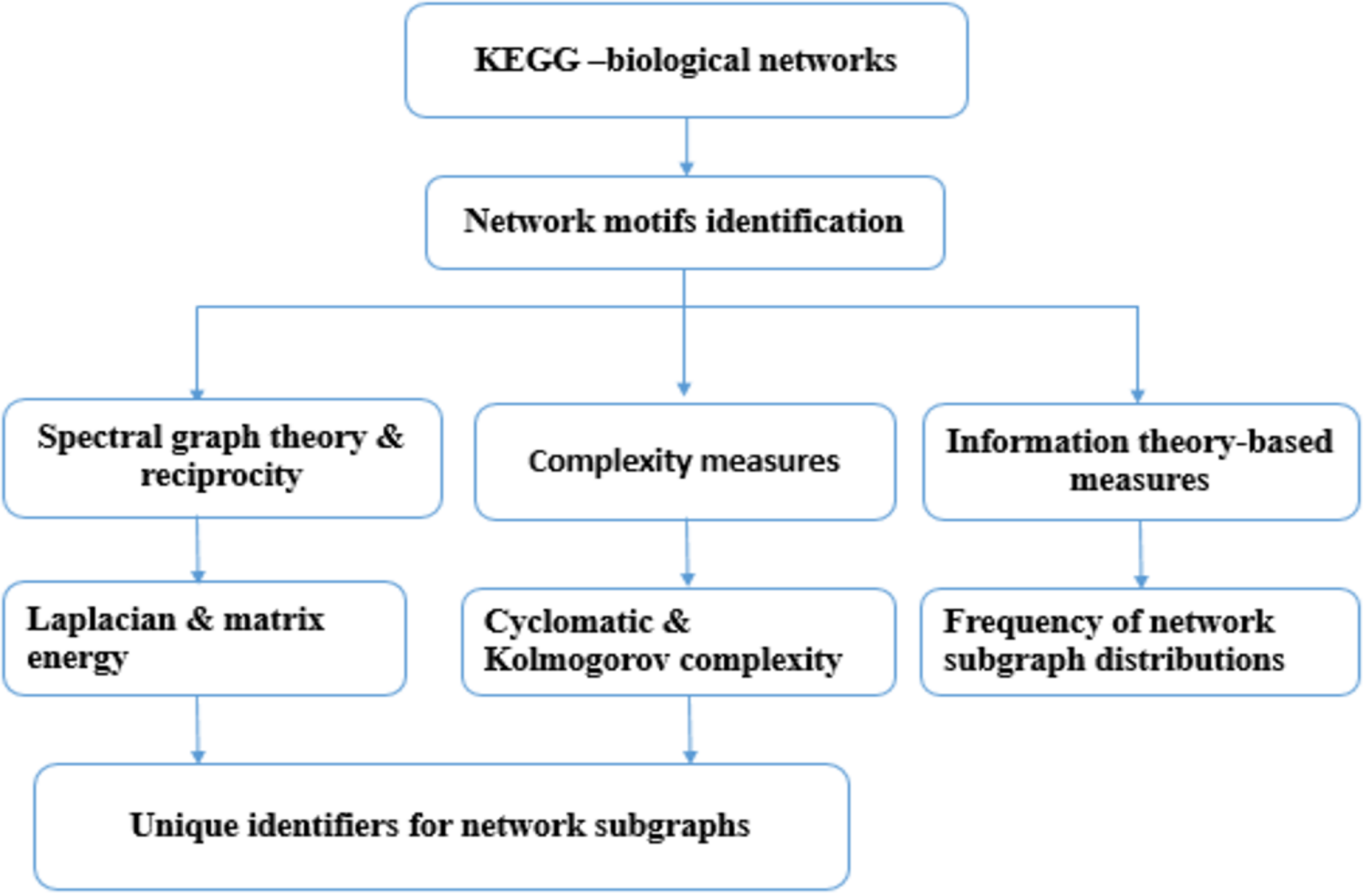
The workflow of the present study. Molecular network information were obtained from the KEGG database (August 2017). Network subgraphs were identified using *PatternFinder* and then network subgraphs were characterized using: graph energy, reciprocity and graph complexity. A code was developed to determine the minimal set of parameters required to label network subgraphs based on a greedy strategy. The Shannon entropy for 3-node subgraphs and 4-node subgraphs of 71 molecular networks were computed.

Figure 1 depicts the workflow of the present study.

### Input data

Cancer is a highly heterogenous and complicated disease. The signal transduction networks are critically involved in modulating cellular processes of lung cancer cell (Cui et al., 2007), in cancer invasion (Di Domenico & Giordano, 2017), and in cellular invasion (Neth et al., 2007). We selected cancer-related molecular networks and analyzed their network structures. The KEGG database (Nakaya et al., 2013) provides a comprehensive collection of biochemical network information that were prepared in the KGML format (August 2017). Four families of networks were employed in the present study, including: (i) Environmental Information Processing, (ii) Cellular Processes, (iii) Organismal Systems, and (iv) Human Cancers.

Not every network recorded by KEGG was imported. After inspection, we disregarded networks composed of several disjoint components with repetitive regulatory structures (“Two-component system” and “MicroRNAs in cancer”), small networks with size less than 10 (“Chemical carcinogenesis” and “Viral carcinogenesis”). In addition, we collected the networks labeled with the name “signaling pathway”, and called them “signal transduction networks (STNs)”. We note that STNs range across different families in the KEGG classification, including “Signal transduction”, “Immune system” and “Endocrine system”.

In total, we collected 17 cancer networks, 45 STNs and nine cellular processes. We downloaded KEGG pathway KGML files and made use of the KEGGScape (Nishida et al., 2014) and KEGGparser (Arakelyan & Nersisyan, 2013) packages to visualize and save the node and edge information for each network.

### Adjacency matrix

By analyzing the connectivity of each gene, one constructs an adjacency matrix *A*, to represent the interaction network. In total, there are 13 3-node subgraphs and 199 4-node subgraphs (Alon, 2006; Shen-Orr et al., 2002). In Supplemental File 1–Table S1 summarizes the nodes, edges, and subgraph-associated node information for the 17 cancer networks. The complete list of node and edge information of the 45 STNs and nine cellular processes can be found in Supplemental File 1–Tables S2 and S3, respectively. Real world molecular networks compose of thousands of genes, which is larger than the networks we analyzed; however, the regulatory and feedback interaction information among thousands of genes are not available in KEGG yet, and it can be considered if the data are available. Each subgraph can be represented by a decimal, the graphical representation of the 3-node subgraphs and 4-node subgraphs are depicted in Supplemental File 2.

It is possible that some subgraphs are subgraphs of other subgraphs (structural subgraphs). In a previous work (Sporns & Kötter, 2004), such subgraphs are called functional motifs. In a brain network, a structural motif and functional motif represent an anatomical building block and the elementary processing mode of a network, respectively.

We have developed an algorithm named *PatternFinder* to enumerate all possible subgraphs embedded in the 3-node subgraphs and 4-node subgraphs. Details about *PatternFinder* are given in Supplemental File 1–Table S4. As we highlighted in “Network subgraphs (N-node subgraphs) versus network motifs”, we addressed the question whether the number of subgraph patterns identified in a network is the same as the number of motif patterns or not. Firstly, we selected three cancer networks, that is, acute myeloid leukemia (AML), breast cancer and colorectal cancer, obtained from the KEGG database. Secondly, we extracted the subgraph patterns and the motif patterns from the three networks by using the *PatternFinder*, *LoTo* (Martin et al., 2017) and *acc-Motif* (Meira et al., 2014) algorithms respectively. The number of randomized networks is set equal to 1,000 times in the *acc-Motif* experiment. The *acc-Motif* algorithm identified network motifs with size up to five nodes, and the algorithm was improved to find motifs up to six nodes (Meira et al., 2014).

### Characterization of network subgraphs: graph energy, reciprocity and graph complexity

The adjacency matrix *A* can be expressed in terms of its eigenvectors and eigenvalues. Since *A* is a nonsymmetric matrix in general, its eigenvalues may be complex and all of its eigenvectors are nonorthogonal. Let *n, e* and *d*_i_ denote the number of nodes, number of edges and degree of the *i*th node of graph *G*, respectively; *G* is called an (*n, e*)-graph. The energy of a graph *G*, *E(G)*, is defined by

(1) $$E(G) = \sum\limits_{i = 1}^n {|{\rm \alpha _i}|}$$

where ${\alpha _{i\; }}$denotes the *i*th eigenvalue of *A*. The sum of all of the eigenvalues is always equal to zero.

Assume that the graph energy eigenvalues are labeled in descending order: that is, α_1_ ≥ α_2_ ≥ … ≥ α_n_, while the whole spectrum is denoted by *Sp(G)* = [α_1_, α_2_, … α_n_]. The largest eigenvalue is referred to as the spectral radius of graph *G* (Cvetkovic, Doob & Sachs, 1980).

In spectral graph theory, there are two other matrices—Laplacian (Chung & Graham, 1997) and signless Laplacian (Cvetkovic & Simic, 2009, 2011) that can be defined to characterize graphs. The Laplacian matrix *L* and signless Laplacian matrix *Q* of a graph G are defined as *L = D – A* and *Q = D + A* respectively, where *D* is a diagonal matrix in which the diagonal elements are the node degrees. The Laplacian energy of a graph *G*, *LE(G)*, is defined by

(2) $$LE(G) = \sum\limits_{i = 1}^n {|{\rm \beta _i}|} - \displaystyle{{2e} \over n}$$

where |β_i_| denotes the absolute value of the *i*th eigenvalue of *L*. There is an analogy between the properties of *E(G)* and *LE(G)*, but some significant differences remain between these two quantities (Gutman & Zhou, 2006). The signless Laplacian energy of graph *G*, *QE(G)*, is defined by

(3) $$QE(G) = \sum\limits_{i = 1}^n {|{\rm \gamma _i}|} - \displaystyle{{2e} \over n}$$

where $|{{\rm \gamma }_{\rm i}}|$ denotes the absolute value of the *i*th eigenvalue of *Q*.

A more general definition of graph energy was suggested by Nikiforov (Nikiforov, 2007, 2016). Let *M* be an *n × n* real matrix and the singular values be denoted by *s*_1_, *s*_2_, … *s*_n_. The singular values of *M* are equal to the positive square roots of the eigenvalues of *MM*^t^, where *t* denotes matrix transpose. Let *M* equals *A*, *L*, or *Q* and consider the eigenvalues of *AA*^t^, *LL*^t^, and *QQ*^t^. The total energy, *ME*, obtained from *M*, is defined by

(4) $$ME(G) = \sum\limits_{i = 1}^n {|{s_i}} |$$

*ME(G)* is called generalized energy. We extend the definition to consider matrix products of the form *MN*^t^, and therefore define three additional energies: *AL*^t^, *AQ*^t^, and *LQ*^t^. We call these asymmetric generalized energies. The sums of the absolute values of the eigenvalues of *MM*^t^ and *M*^t^*M* are the same. This also holds for *MN*^t^ and *NM*^t^. Therefore, one needs to compute *MM*^t^ and *NM*^t^ only. The advantages of using asymmetric generalized energies will be demonstrated later in this article. To the best of our knowledge, no (or few) previous studies have made use of asymmetric generalized energies to characterize network subgraphs. In total, we have devised nine graph energies to describe the subgraphs. We also note that Adiga, Balakrishnan & So (2010) proposed a parameter named skew energy, obtained from the skew-adjacency matrix, to characterize directed graphs; however, this parameter does not apply to graphs consisting of multiple arcs (multigraphs).

Several studies (Garlaschelli & Loffredo, 2004; Squartini et al., 2013) have suggested that reciprocal links in directed networks play an important role in dynamical processes and network growth. The traditional definition of reciprocity is *R* = ${L^ \leftrightarrow }/L$, where ${L^ \leftrightarrow }$ and $L$ denote the number of “edges pointing in both directions” and the total number of edges respectively. This definition of reciprocity was modified by Garlaschelli and Loffredo (McCabe, 1976), who defined reciprocity *r* as the correlation coefficient between the entries of the adjacency matrix *A*, given by

(5) $$r = \displaystyle{{\sum\nolimits_{i \ne j} {({a_{ij}} - \bar a)({a_{ji}} - \bar a)} } \over {\sum\nolimits_{i \ne j} {{{({a_{ij}} - \bar a)}^2}} }}$$

where *r* equals one if there is an edge from node *i* to node *j;* the average, $\bar a$, is defined by

(6) $$\bar a = \displaystyle{{\sum\nolimits_{i \ne j} {{a_{ij}}} } \over {N(N - 1)}}$$

A positive value of *r* indicates that the subgraph has bidirectional connections, whereas a negative *r* implies that the subgraph has either an in-connection or out-connection.

To further understand the connectivity structure of network subgraphs, we seek metrics that can be used to measure graph complexity. In software engineering, the cyclomatic complexity (*CC*) is a metric developed by (McCabe, 1976) to measure the complexity of a program by using its control flow graph. *CC* is defined by the expression *CC* = *e – N + 2P*, where *e* and *N* denote the number of edges and number of nodes of the graph, and *P* denotes the number of predicate/exit nodes (McCabe, 1976; Weyuker, 1988). Node and edge denote a program unit and the execution order of the program. *CC* depends only on the global decision structure (the number of edges and nodes) of a program. In addition to *CC*, we utilize the algorithmic complexity measure, the Kolmogorov complexity (*KC*), to characterize graph complexity. Essentially, the *KC* of a bit string is given by the smallest computer program that can generate the string. Given the adjacency matrix (or the equivalent bit string), we use the block decomposition method (BDM) (Soler-Toscano et al., 2014) to determine the *KC* for both 3-node (Zenil, Kiani & Tegner, 2016) and 4-node subgraphs. A bit string with a high *KC* has a higher degree of randomness, contains more information, and is less compressible. A complete graph has a smaller *KC* value, whereas a random graph has higher *KC* and is less compressible.

### Unique identifiers for network subgraphs

Every 3-node subgraph and 4-node subgraph has a different *KC* value, so the *KC* can be used as a unique identifier. However, given the graph energy, asymmetric graph energies, graph energy spectrum, reciprocity, and *CC*, we seek to determine a minimal set of parameters that can serve as a label of the network subgraphs. This set of parameters describes certain aspects of the subgraphs differently than the algorithmic complexity measure. To the best of our knowledge, the concept of using energy, reciprocity, and *CC* in labeling network subgraphs is novel. The pseudocode for determining the minimal set of parameters is based on a greedy strategy and is described in Supplemental File 1–Table S5.

### Frequently found subgraphs, network entropy

Given a molecular network, *PatternFinder* identifies both the sets of 3-node subgraphs and 4-node subgraphs. Two subgraphs with the same ID may partially embed the same genetic element(s); these two subgraphs are counted twice in our calculations. We expect that certain subgraph patterns that occur with higher probabilities are the dominant underlying network structure. Let ${\rm p}_3^{\left( {\rm k} \right)}$ denote the frequency (probability) distribution of a 3-node network subgraph, where *k* denotes one of the 13 patterns. The Shannon entropy for 3-node subgraphs and 4-node subgraphs, *H*_3_ and *H*_4_, of a molecular network are computed. The normalized Shannon entropies for the 3-node subgraphs and 4-node subgraphs are given by *H*_3R_ = *H*_3_/log_2_(13) and *H*_4R_ = *H*_4_/log_2_(199), respectively.

### Association of network subgraphs and driver genes

In this part of calculation, we propose to examine the association of network subgraphs and driver genes for cancer networks, STN and cellular processes. It is commonly believed that driver genes are genes that give selective advantage to cancer development, whereas passenger mutation genes do not alter selectivity pressure, they contribute indirectly to cancer formation (Bozic et al., 2010; Greenman et al., 2007).

We noted that most of the studies are focus on mutation driver genes prediction (Tokheim et al., 2016). Also, it was found that certain motif positions; such as, the source nodes and target nodes, are enriched in cancer-associated genes (Awan et al., 2007; Carson et al., 2015). There is no or relative few works on estimating how often is the driver genes embedded within network subgraphs. In this study, we conducted an analysis to determine whether driver genes are enriched or depleted in network subgraphs or not. The level of enrichment or depletion was evaluated by using odds ratio. Driver gene data were collected from the Cancer Gene Census (CGC) database (Futreal et al., 2004). The CGC resource provides list of genes known to be involved in cancer. CGC has documented the mutation information of cancer driver genes which are supported by rather extensive evidence in the literature (both from mutation studies and activity measurements). In the present CGC release, the database has collected 576 driver genes.

Given a molecular network, consider the 2 × 2 contingency table (Table 1), which depicts the statistics of driver genes and non-driver genes embedded in “subgraph module” and “non-subgraph module”. Subgraph module denotes the collection of the 3-node subgraphs and 4-node subgraphs found by *PatterFinder*; whereas, non-subgraph module denotes otherwise. We use the odds ratio (OR) to estimate the level of enrichment. OR measures the relative odds of finding driver genes embedded in network subgraph modules relative to non-subgraph modules. The *OR* is defined by

(7) $${\rm OR} = \displaystyle{{\displaystyle{{{p}({\rm driver}\_{\rm gene\; }|{\rm \; subgraph}\_{\rm module})} \over {1 - {p}({\rm driver}\_{\rm gene\; }|{\rm \; subgraph}\_{\rm module})}}} \over {\displaystyle{{{p}({\rm driver}\_{\rm gene\; }|{\rm \; non}\_{\rm subgraph}\_{\rm module})} \over {1 - {p}({\rm driver}\_{\rm gene\; }|{\rm \; non}\_{\rm subgraph}\_{\rm module})}}}} = \displaystyle{{{a} \times {d}} \over {{b} \times {c}}}$$

**Table 1:** The 2 × 2 contingency table for driver genes and non-driver genes embedded in “Subgraph module” and “non-subgraph module”.

	Subgraph module	Non-subgraph module	Total
Driver genes	*a*	*b*	*a + b*
Non-driver genes	*c*	*d*	*c + d*
Total	*a + c*	*b + d*	*a + b+ c + d*

If OR is greater than one, it means that network subgraphs are enriched with driver genes.

## Results

The results of subgraph and motif patterns identified by *PatternFinder*, *acc-Motif*, and *LoTo* are summarized in Table 2. We set the number of randomized networks equal to 1,000 when executing *acc-Motif*. Note that the tool *LoTo* identifies motifs composed of three nodes only. The number of subgraphs and motifs identified by the three algorithms are similar. However, there are two major differences. First, not every motif identified by *acc-Motif* is associated with a significant *p*-value. For instance, the number of 3-node subgraphs identified for AML is four. None of the identified motifs is significant according to *acc-Motif* (Table 2). In addition, both *PatternFinder* and *LoTo* identified four 3-node subgraphs for the breast cancer network, but *acc-Motif* identified only one significant 3-node motif. Second, the results for the 4-node subgraphs and motifs are given in the bottom half of Table 2. Clearly, *PatternFinder* can identify the complete set of subgraphs, but *acc-Motif* identified relatively few network motifs with *p* < 0.05. Furthermore, *acc-Motif* did identify two 4-node subgraphs, “id_456” and “id_2186”, for AML and breast cancer, respectively. This suggests that *acc-Motif* has certain limitations and cannot enumerate all possible network substructures. Essentially, the results suggest that the subgraph-based approach delivers similar results to the motif-based approach. The graphical and ID representation of the 3-node and 4-node motifs defined by *acc-Motif* are depicted in Supplemental File 3. This can help the reader to relate subgraph IDs to the IDs defined by *acc-Motif*.

**Table 2:** The results of the subgraph patterns and motif patterns identified by *PatternFinder, LoTo* and *acc-Motif*. The integers listed along the row “*PatternFinder*” and “*LoTo*” denote the decimal representation of the subgraphs and the integers listed along the row “*acc-Motif*” denote the *acc-Motif* IDs.

Cancer	Tools	3-Node subgraph pattern ID				
AML	*PatternFinder*	6	12	36	38	
	*LoTo*	6	12	36	38	
	*acc-Motif*	1,120	11,011	21,010	21,120	
	Frequency ± sd	72.73 ± 3.26	45.69 ± 5.58	20.54 ± 2.49	3.15 ± 1.88	
Breast	*PatternFinder*	6	12	36	38	
	*LoTo*	6	12	36	38	
	*acc-Motif*	1,120	11,011*	21,010	21,120	
	frequency ± sd	44.89 ± 5.62	42.24 ± 6.75	37.02 ± 3.14	3.53 ± 2.88	
Colorectal	*PatternFinder*	6	12	36	38	98
	*LoTo*	6	12	36	38	1
	*acc-Motif*	1,120	11,011	21,010*	21,120	111,111*
	frequency ± sd	29.71 ± 1.25	38.86 ± 3.53	24 ± 0.58	1.14 ± 0.9	0.43 ± 0.79

Cancer	Tools	4-node subgraph pattern ID																
AML	*PatternFinder*	14	28	74	76	78	92	204	206	280	328	344	392	408	456	472	2184	2186
	*acc-Motif*	A	B	C*	D	E	F	G*	H	I	J	K	L	M	***NA***	N	O*	P
	frequency ± sd	81.04 ± 5.4	34.63 ± 7.38	119.1 ± 21.72	101.75 ± 16.46	7.98 ± 4.85	3.65 ± 2.53	1.22 ± 1.35	0.20 ± 0.57	27.84 ± 7.22	31.95 ± 10.39	3.49 ± 2.75	27.68 ± 9.07	2.86 ± 2.09	***NA***	0.2 ± 0.49	8.67 ± 2.71	2.86 ± 1.96
Breast	*PatternFinder*	14	28	74	76	78	92	204	280	328	344	392	394	904	2184	2186	2190	
	*acc-Motif*	A*	B	C	D	E	F*	G*	I	J*	K*	L*	Q*	R*	O*	***NA***	S*	
	frequency ± sd	38.28 ± 4.34	33.24 ± 6.37	69.05 ± 16.89	106.82 ± 18.68	4.24 ± 3.03	2.49 ± 2.69	0.83 ± 1.16	16.63 ± 4.28	32.7 ± 7.34	0.8 ± 1.12	54.26 ± 11.31	1.29 ± 1.44	0.63 ± 0.94	25.35 ± 3.13	***NA***	0.09 ± 0.35	
Colorectal	*PatternFinder*	14	28	74	76	78	280	328	392	394	408	904	2184	2186				
	*acc-Motif*	A*	B*	C	D	E	I*	J*	L	Q	M	R*	O*	P				
	frequency ± sd	24.05 ± 2.98	13.28 ± 3.32	44.71 ± 11.56	42.93 ± 14.36	2.42 ± 2.62	12.28 ± 3.71	28.18 ± 6.8	34.19 ± 7.92	0.37 ± 0.68	0.55 ± 1.10	0.38 ± 0.582	15.37 ± 2.34	1.96 ± 1.95				

**Notes:**

* Indicates a significant pattern, *p* < 0.05.

Capital letters A–S denote the *acc-Motif* ID, that is, A = 905928928928; B = 243329587587; C = 6184; D = 4118; E = 4119; F = 4126; G = 682682684684; H = 507557800800; I = 80637637639; J = 4625; K = 4259; L = 5377; M = 4261; N = 4263; O = 423423423573; P = 4389; Q = 4197; R = 101223223480; S = 208208210840.

Given the 3-node and 4-node subgraphs, we used *PatternFinder* to identify all possible functional subgraphs. For the 3-node subgraphs, subgraph “id_6” (SIM, single input module), subgraph “id_12” (cascade), and subgraph “id_36” (MIM, multiple input module) are not composed of any 3-node functional subgraphs. For the 4-node subgraphs, there are eight subgraphs that are not composed of any 4-node functional subgraphs: subgraph “id_14” (SIM), subgraph “id_28,” subgraph “id_74,” subgraph “id_76” (MIM), subgraph “id_280,” subgraph “id_328” (cascade), subgraph “id_392,” and subgraph “id_2184”. Thus, these eight subgraphs exhibit *irreducibility*. However, each of the eight subgraphs is embedded with exactly one 3-node functional subgraph. In other words, given the 4-node subgraphs, the *irreducible* property does not apply if we consider subgraphs composed of three nodes. Supplemental File 4 summarizes the 3-node functional subgraphs embedded in the 3-node subgraphs. Supplemental File 5 summarizes the 3-node and 4-node functional subgraphs embedded within the 4-node subgraphs. In these files, integers “1” and “0” denote the presence and absence of a functional subgraph, respectively.

### Spectral graph theory, reciprocity, complexity measures

Table 3 summarizes the results of the nine graph energies and edge information for the 3-node subgraphs. First, since some matrices, such as *L* and *Q*, are asymmetric, their eigenvalues are complex. In fact, among the 3-node subgraphs, subgraph “id_98” has a pair of complex conjugate eigenvalues, and their associated eigenvectors are composed of complex components. Second, graph energy is correlated with the number of subgraph edges. For instance, the graph energies of fully-connected 3-node and 4-node subgraphs are maximal, despite having different energy definitions. Third, it is common for certain subgraphs to have the same graph energy. That is, energy-degenerated subgraphs are common. Two subgraphs are said to be *equienergetic* if they have the same total energy. For instance, two subgraph pairs (“id_6” and “id_36,” and “id_14” and “id_74”) are *equienergetic*, regardless of the graph energy definition. The 3-node and 4-node subgraph energy and eigenvector results are given in Supplemental File 6. Fourth, although the results of the nine graph energies are quite similar, there are differences among them. For instance, the energy level multiplicity is somewhat different. For the 3-node subgraphs, the multiplicities of graph energy *E*, 0, 2 and 2.83 are 4, 4 and 1, respectively. For *QE*, there are three energy values: 2.67, 4.00 and 5.33, which are associated with the multiplicity of 3, 3 and 3, respectively. Fifth, energy-degenerated subgraphs may or may not have identical spectra, *Sp*(*G*). This suggests that *Sp*(*G*) could allow for further distinction between subgraphs. More details are given below in the “Unique identifiers for network subgraphs” section.

**Table 3:** The results of the nine graph energies for the 3-node subgraphs. *E* is the graph energy, *LE* is the Laplacian energy, Q*E* is the signless Laplacian energy. Matrix product of the bilinear form *MM*^t^ is the so-called generalized energy, where *M* and *N = A, L* and *Q. t* denotes matrix transpose. The asymmetric generalized energy matrix is denoted by *NM*^t^.

ID	*E*	*LE*	*QE*	*AA*^t^	*LL*^t^	*QQ*^t^	*AL*^t^	*AQ*^t^	*LQ*^t^
6	0.00	2.67	2.67	1.41	4.32	4.32	1.41	1.41	3.83
12	0.00	2.67	2.67	2.00	4.34	4.34	2.00	2.00	3.93
14	2.00	4.00	4.00	2.41	6.13	6.13	3.00	3.00	5.45
36	0.00	2.67	2.67	1.41	4.32	4.32	1.41	1.41	3.83
38	0.00	4.00	4.00	2.24	6.39	6.34	2.63	2.37	6.01
46	2.00	5.33	5.33	2.73	8.24	8.16	2.00	3.86	7.59
74	2.00	4.00	4.00	2.41	6.13	6.13	3.00	3.00	5.45
78	2.83	5.33	5.33	2.83	8.00	8.00	3.86	3.86	7.29
98	3.00	4.29	4.46	3.00	6.29	6.46	4.25	4.36	6.01
102	3.06	5.33	5.56	3.24	8.17	8.25	4.76	5.02	7.57
108	2.00	5.33	5.33	2.73	8.24	8.16	2.00	3.86	7.59
110	3.24	6.67	6.72	3.49	10.09	10.09	5.38	5.70	9.40
238	4.00	8.00	8.00	4.00	12.00	12.00	6.47	6.93	11.21

In Supplemental File 1–Table S6 summarizes the lower (*E*_min_) and upper (*E*_max_) graph energy bounds and ratios for the 3-node and 4-node subgraphs. For the 3-node subgraphs, the ratios (*E*_max_/*E*_min_) are bounded between 2 and 4.91. The ratios are slightly larger for 4-node subgraphs. They are bounded between 3.00 and 6.88. We observed that most molecular biological networks are not composed of subgraphs with large graph energies. Therefore, the maximum ratio cannot be achieved. Details are reported below in the “Network subgraphs absent from the network” section.

### Subgraph reciprocity

Table 4 depicts the traditional reciprocity *R*, reciprocity *r*, and $\bar a$ for the 3-node subgraphs. Most *R* values are zero, which indicates that no edges point in both directions. Positive and negative values of *r* denote the presence of cycles. Of the 13 reciprocity values, nine are negative, meaning that most 3-node subgraphs have only in- or out-connections. We note that subgraphs containing one or two cycles can still have negative reciprocity values. The complete sets of *R*, *r*, edges, and $\bar a$ values of the 4-node subgraphs are given in Supplemental File 7.

**Table 4:** The results of traditional reciprocity (*R*), reciprocity (*r*), edge (*e*) and average reciprocity of the 3-node subgraphs.

ID	*R*	*r*	*e*	$\bar a$
6	0	−0.5	2	1/3
12	0	−0.5	2	1/3
14	0	1/3	3	0.5
36	0	−0.5	2	1/3
38	0	−1	3	0.5
46	0	−0.5	4	2/3
74	0	1/3	3	0.5
78	1	1	4	2/3
98	0	−1	3	0.5
102	0	−0.5	4	2/3
108	0	−0.5	4	2/3
110	0	−0.2	5	5/6
238	1	1	6	1

### Cyclomatic complexity and Kolmogorov complexity

For the 3-node subgraphs, Table 5 summarizes the results of the cyclomatic complexity (*CC*), Kolmogorov complexity (*KC*), and their rankings. The ranges of *CC* and *KC* values are 0–3 and 23.34–25.50, respectively. The complete sets of *CC* and *KC* values of the 4-node subgraphs are given in Supplemental File 8, where the ranges of *CC* and *KC* values are 0–8 and 33.80–43.74, respectively. These findings are compatible with the notion that subgraphs composed of more nodes have higher complexity. We had developed MATLAB programs to calculate the adjacency matrix, graph energy, reciprocity, graph complexity (*CC)* and used *The Online Algorithmic Complexity Calculator* (https://complexitycalculator.com/) to compute *KC*.

**Table 5:** The results of cyclomatic complexity (*CC*) and Kolmogorov complexity (*KC*) and their ranking for the 3-node subgraphs.

ID	*CC*	*KC*	Rank of *CC*	Rank of *KC*
6	3	23.34	11	1
12	1	23.83	3	3
14	2	24.30	8	6
36	1	23.55	3	2
38	2	24.87	8	8
46	3	25.50	11	13
74	0	23.85	1	4
78	1	25.00	3	9
98	0	24.82	1	7
102	1	25.01	3	10
108	1	25.11	3	11
110	2	25.25	8	12
238	3	24.14	11	5

A network subgraph with a large *CC* value suggests a more complex decision structure. From Table 5, it is apparent that *KC* can serve as a parameter for distinguishing subgraph patterns without any degeneracy. In other words, no two subgraphs have the same *KC*. This is also true for 4-node subgraphs. Subgraph “id_238” is a complete graph described by the binary string “011101110,” which corresponds to lower algorithmic complexity (fifth rank).

Next, we examined the correlations between the two complexity measures. We ranked *CC* and *KC* in ascending order and computed their Spearman Rank Correlation Coefficients (*SRCC*). We observed that the correlation is not perfect. For example, subgraph “id_238” is associated with the largest *CC* value (rank), but this is not the case for *KC* (fifth rank). *CC* and *KC* show a relatively weak correlation—0.083 and 0.381—at the 3-node and 4-node levels, respectively. This is because *CC* and *KC* have different meanings: *CC* measures the complexity of the subgraph decision structure (the number of independent gene regulation paths), while *KC* is an algorithmic measure which characterizes the randomness and compressibility of a bit string.

Finally, we investigated the relationship between graph energy and complexity (Supplemental File 1–Table S7). *KC* exhibits a modest correlation with all the graph energies at the 3-node and 4-node levels. In contrast, *CC* exhibits relatively weak and modest correlations with graph energy at the 3-node and 4-node subgraph levels.

Supplemental File 1–Table S8 summarizes the results of strength of *SRCC* (including minimum, maximum, and ranges) between graph complexity and energy for 3-node and 4-node subgraphs. Our results suggest that there are relatively weak (3-node *CC*) and modest correlations (3-node subgraph *KC* and 4-node subgraph *C. and KC*) between graph complexity and graph energy.

### Unique identifiers for network subgraphs

This section reports the results of determining an optimal parameter combination that maximizes the removal of degenerated subgraphs. As shown in Table 6, three cases are considered. “Case A” uses graph energy only, “Case B” utilizes graph energy, *r* and *CC*, and “Case C” employs energy, *r*, *CC*, and the energy spectrum. After including *r* and *CC*, we can distinguish more subgraphs. Using *AL*^t^, *r*, *CC*, and energy spectrum can fully distinguish the 3-node subgraphs. For 4-node subgraphs, the use of *LL*^t^, *QQ*^t^ and *LQ*^t^ achieves the best level of distinguishability: 136 out of 199 (68.3%). Compared with *E. LE*, and *QE*, both *symmetric* and *asymmetric* generalized energies serve as superior measures for distinguishing different subgraph patterns.

**Table 6:** The number of distinguishable subgraphs using optimal combinations of graph energy, *r, CC* and energy spectrum.

	3-Node subgraphs			4-Node subgraphs		
	Case A	Case B	Case C	Case A	Case B	Case C
*E*	7	11	11	42	57	60
*LE*	6	10	11	35	51	96
*QE*	9	11	11	51	67	72
*AA*^t^	10	12	12	74	86	92
*LL*^t^	10	12	12	94	103	136
*QQ*^t^	10	12	12	88	96	136
*AL*^t^	10	12	13	117	128	130
*AQ*^t^	10	12	12	120	129	131
*LQ*^t^	10	12	12	109	117	136

**Note:**

“Case A” uses graph energy only, “Case B” uses graph energy, *r* and *CC* and “Case C” uses graph energy, *r*, *CC*, and graph energy spectrum.

### Network subgraphs found with high occurrence frequency

Among the 17 cancer networks, 45 STNs, and nine cellular processes, there are 15 (88.2%), 40 (87.0%) and seven (77.8%) networks, respectively, where more than 70% of nodes are embedded in both 3-node subgraphs and 4-node subgraphs. Therefore, subgraph-associated nodes account for a major portion of each network. We enumerated all possible 3-node subgraphs and 4-node subgraphs for the 17 cancer networks, 45 STNs, and nine cellular processes.

**Figure 2:**
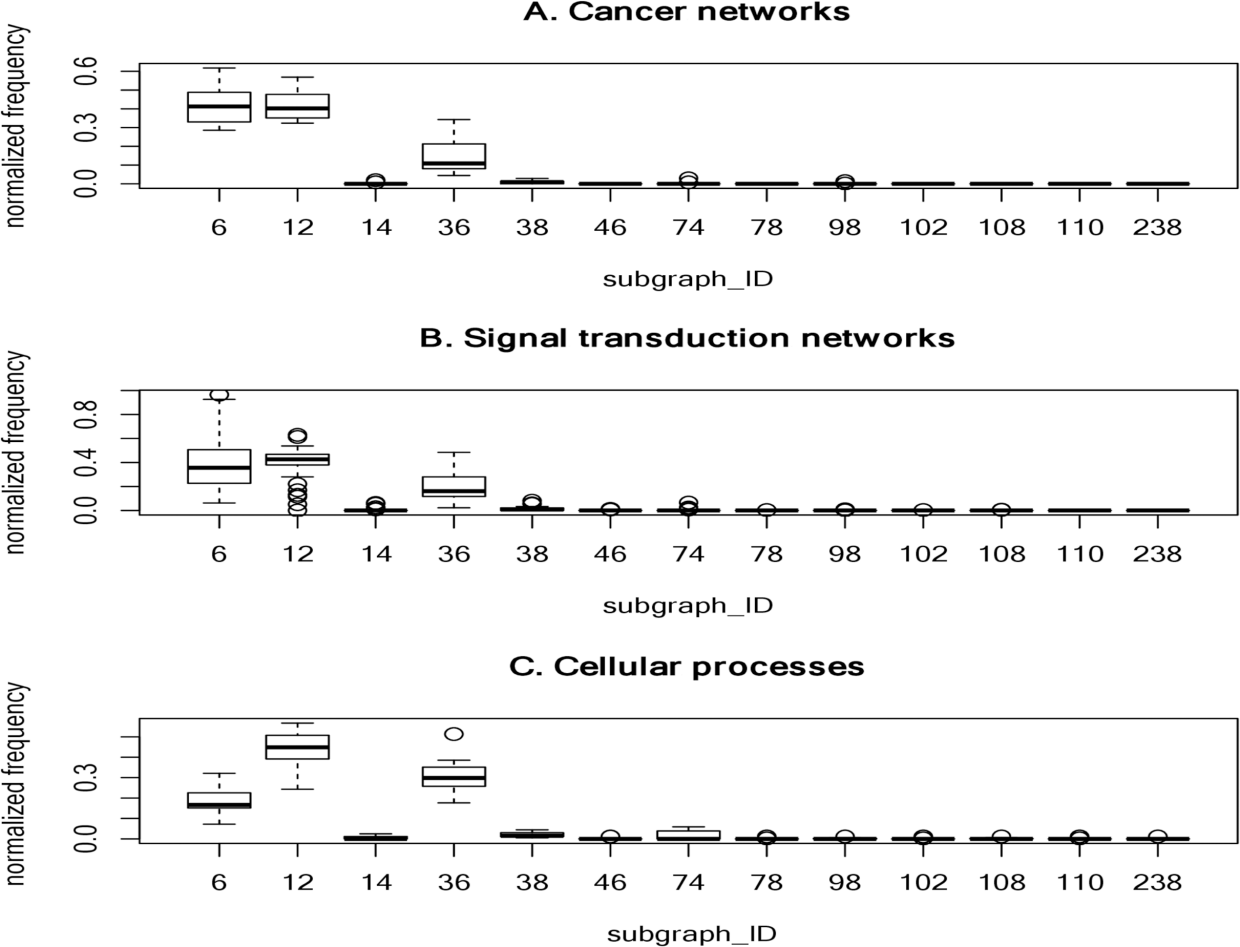
The plots of the normalized frequency of the thirteen 3-node subgraphs for (A) the cancer networks, (B) the Signal transduction networks and (C) the cellular processes. Certain subgraphs are never present in the three molecular network types. In other words, while networks are built from a finite number of subgraph patterns, certain subgraphs associated with large graph energies are not present.

To determine frequently-occurring subgraphs, we tabulated the occurrence frequency of each subgraph pattern, and normalized the frequency to one. Figure 2 summarizes the normalized frequency distribution of the 3-node subgraphs for the cancer networks, STNs, and cellular processes. Subgraphs id_6, id_12 and id_36 dominate the three classes of molecular networks.

For cancer networks, the three most frequently found 3-node subgraphs are id_6, id_12 and id_36 (Supplemental File 1–Table S9). By examining the top three subgraphs, we observed the following common features: (1) they do not contain any subgraphs (*irreducible*); (2) they are composed of a minimal edge number (*N*-1 edges for a *N*-node subgraph); (3) the reciprocity *r* values are negative (−1/2) and those subgraphs have *no* cycles; (4) they account for more than 15% of the frequency counts; and (5) they are associated with the lowest or the second lowest graph energies, regardless of the graph energy definition.

The subgraphs ranked 4 to 7 (“id_38”, “id_74”, “id_14” and “id_98”) have three edges. Subgraph “id_38” is the so-called feed-forward loop (FFL), which does not contain cycles, whereas both “id_74” and “id_14” contain cycles. The seventh-ranked subgraph (“id_98”) is the so-called 3-cycle. Subgraph “id_12” is a subgraph of “id_38”, “id_74” and “id_98”, while “id_36” (MIM) is a subgraph of FFL, and SIM is a subgraph of “id_14”. In other words, the frequently-occurring subgraphs are the simplest and are subgraphs of more complex subgraphs.

For 4-node subgraphs, the above features (1–4 but not feature 5) are also valid for the top seven most frequently occurring subgraphs. Interestingly, the *irreducible* and negative reciprocity value (−1/3) features hold at the 4-node level. In addition, feature (5) holds if we consider graph energies *E. LE* and *QE*, but not the other six graph energy definitions. Furthermore, the above five features also hold for STNs and cellular processes. Supplemental File 1–Tables S9–S11 summarize the top seven most frequently occurring 3-node subgraphs and 4-node subgraphs in cancer, STNs and cellular processes, respectively.

We observed that the normalized frequency of subgraphs id_6 and id_12 is higher than id_36 for cancer networks and STNs (Fig. 2), which suggests that the underlying network architecture is highly similar. In addition, the tails of the normalized distribution of the three types of networks are zero or nearly zero, indicating that molecular networks are composed of a *finite* number of subgraph patterns–approximately seven patterns.

We observed that only two of the cellular processes composed of 3-node subgraph patterns are associated with large graph energies. The first is the “adherens junction” network which consists of a 3-node subgraph (id_110) composed of three genes: *CTNNA1*, *ACTB* and *AFDN*. The second is the “Signaling pathways regulating pluripotency of stem cells” network. We identified a fully-connected 3-node subgraph (id_238) with three feedback loops connecting three genes: *Oct4, Sox2*, and *Nanog*. Including *LIN28*, these four genes can reprogram human somatic cells into pluripotent stem cells (Yu et al., 2007).

**Figure 3:**
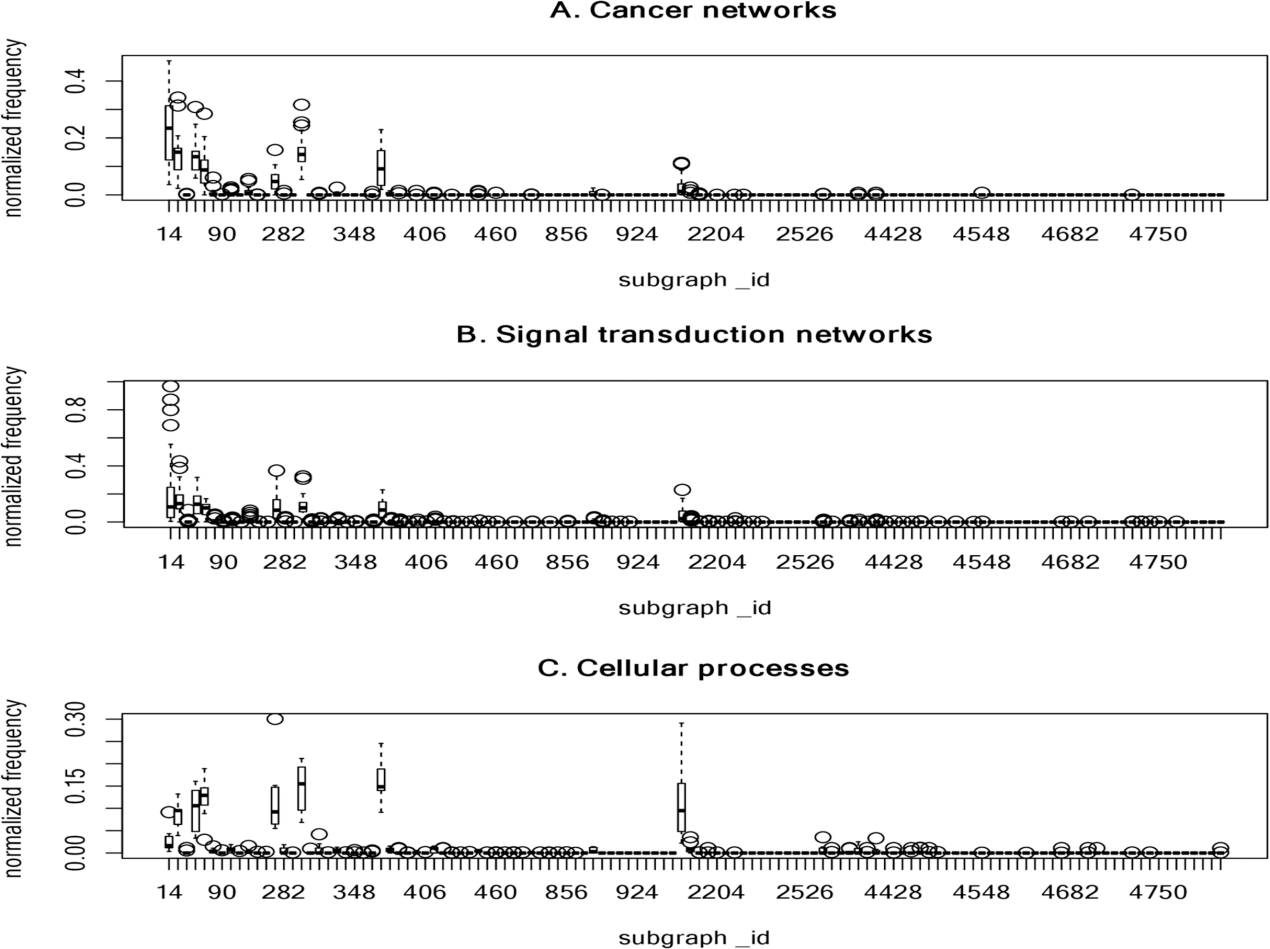
The plots of the normalized frequency of the 4-node subgraphs for (A) the cancer networks, (B) the signal transduction networks and (C) the cellular processes. Only the first 120 subgraphs’ normalized frequency are shown, the rest of the subgraphs have zero normalized frequency. Eight subgraphs (id_14, id_28, id_74, id_76, id_280, id_328, id_392, and id_2184) dominate the three molecular network classes. These results indicate that molecular networks are composed of a *finite* number of subgraph patterns.

Figure 3 summarizes the normalized frequency distribution of the 4-node subgraphs for the cancer networks, STNs and cellular processes. Eight subgraphs (id_14, id_28, id_74, id_76, id_280, id_328, id_392 and id_2184) dominate the three molecular network classes. The normalized frequency from the first 120 subgraphs is shown (Fig. 3). The rest of the subgraphs have zero or nearly-zero normalized frequency. Again, these results indicate that molecular networks are composed of a *finite* number of subgraph patterns.

Next, we examined the association of frequently-identified subgraphs and complexity measures. We observed that frequently-identified subgraphs have a lower *KC* ranking (smaller *KC* value) (Table 5). A smaller *KC* value implies a lower degree of randomness, less information, and higher compressibility. However, this observation is not exact at the 4-node level. Indeed, there are three instances where the rank of *KC* is larger. For instance, the *KC* rank is as high as 16, 24 and 33 for id_2184, id_280 and id_392, respectively. No obvious association exists between frequently-occurring subgraphs and *CC* measures. The ranking information can be found in Supplemental File 8.

### Network subgraphs that are absent from the networks

It is interesting that certain subgraphs are never present in the three molecular network types. In other words, while networks are built from a finite number of subgraph patterns, certain subgraphs associated with large graph energies are not present. In Table 7, we summarized the results of the 3-node and 4-node subgraph patterns present in the 17 cancer networks, 45 STNs, and 9 cellular processes. Among the 13 possible 3-node subgraph patterns, only 7 patterns can be identified in the 17 cancer networks. The absent patterns are: id_46, id_78, id_102, id_108, id_110 and id_238. These six missing patterns are shown in Fig. 2 as boxes with zero height. For the cellular processes, the complete set of 3-node subgraphs were observed. In the case of 4-node subgraphs, only certain patterns were identified in the three network types. If there are subgraphs absent in a network, there is an associated graph energy cutoff. Hence, the network is characterized by the graph energy cutoff. To the best of our knowledge, we are the first to discover this feature. We depict the results of the energy cutoffs (cutoff), maximum graph energies (max), and ratios (cutoff/max) for the cancer networks in Supplemental File 1–Table S12. Among the nine graph energy definitions, the ratios may be as high as 0.750 and 0.667 for 3-node subgraphs and 4-node subgraphs, respectively. However, they can also be as low as 0.536 (*LQ*^t^ energy) and 0.481 (*AQ*^t^ energy) for the 3-node subgraphs and 4-node subgraphs, respectively. The results of the STN and cellular process graph energy cutoffs and ratios are given in Supplemental File 1–Tables S13 and S14. For cellular processes, the cutoff ratio may be as high as 1.00 for the 3-node subgraphs, because we identified a fully connected 3-node subgraph (id_238). At the 3-node level, two of the cellular processes (“adherens junction” and the “Signaling pathways regulating pluripotency of stem cells”) exhibit peculiar network structures. This findings may worth for further investigation in future study. Our results suggest that there is an energy cutoff or ratio that constrains the presence of certain subgraphs embedded within a molecular network. In addition, the data indicate that the ratio for 3-node subgraphs is slightly higher than for 4-node subgraphs. Furthermore, the subgraph-normalized frequency distribution and the graph energy obeys an inverse relationship, with smaller probability associated with higher graph energy.

**Table 7:** The results of the number of possible 3-node subgraph patterns and 4-node subgraph patterns found in the 17 cancer networks, 45 STNs and nine cellular processes.

	Cancer network	STN	Cellular process
3-node subgraph	7/13	11/13	13/13
4-node subgraph	38/199	88/199	77/199

**Note:**

The maximal number of possible 3-node subgraphs and 4-node subgraphs are thirteen and 199 respectively.

### Characterizing the subgraph frequency distributions

**Figure 4:**
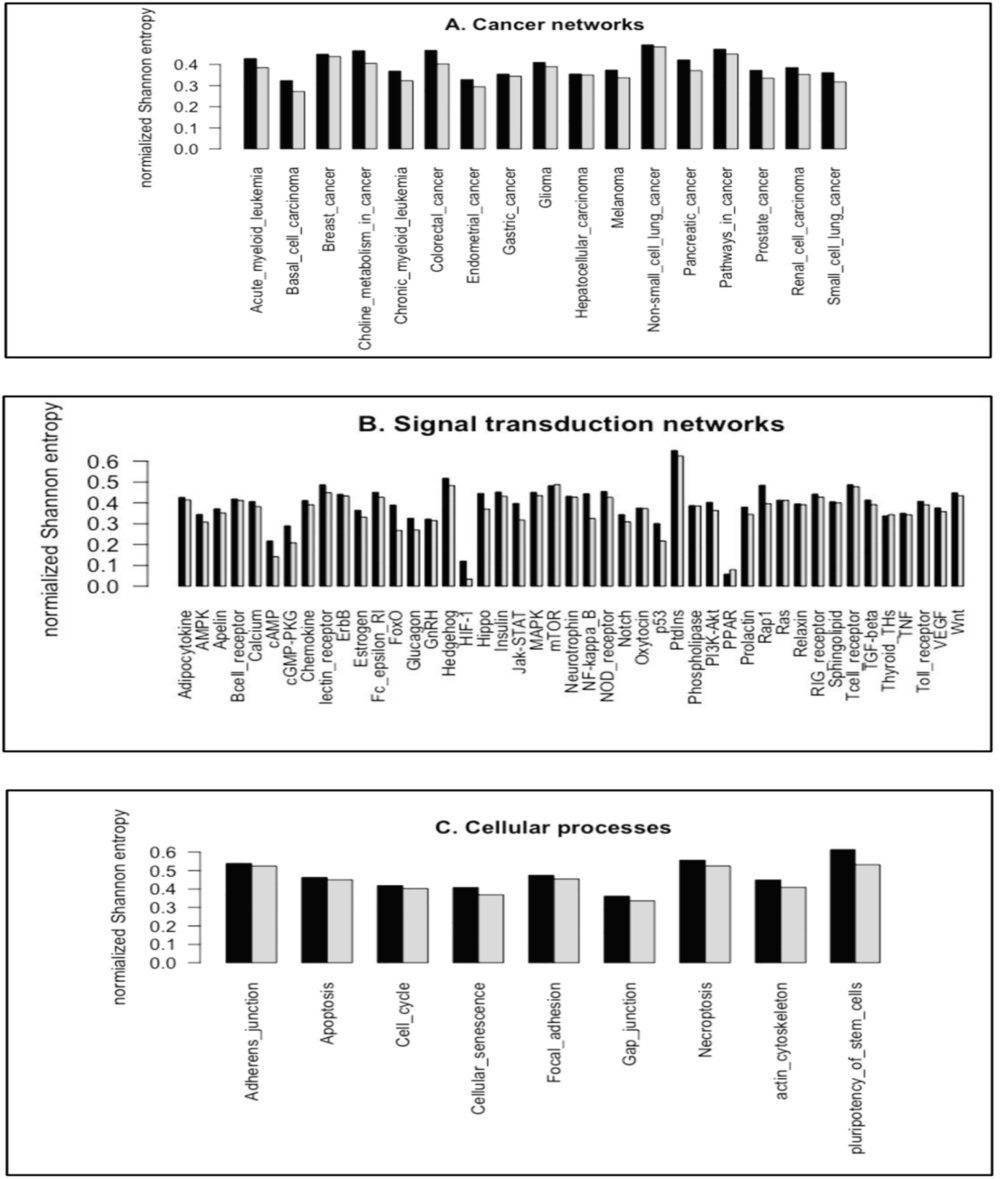
The plots of the normalized Shannon entropy for the 3-node subgraphs (black, H3R) and the 4-node subgraphs (grey, H4R), where (A) the cancer networks, (B) the signal transduction networks, and (C) the cellular processes. The normalized frequency distributions are not uniformly distributed among the subgraph patterns. Therefore, H3R and H4R are different from zero and one.

We utilized the entropy-based quantity, normalized Shannon entropy, *H*_R_, to quantify the frequency distributions of the subgraph occurrence for the cancer networks. For a randomized distribution, *H* achieves the maximal values, 3.700 (log_2_(13)) and 7.637 (log_2_(199)), for 3-node subgraphs and 4-node subgraphs, respectively. Figs. 4A–4C depict the *H*_R_ plots for 3-node subgraphs (*H*_3R_) and 4-node subgraphs (*H*_4R_) for the cancer networks, STNs (Supplemental File 1–Tables S15) and cellular processes (Supplemental File 1–Table S16).

For the networks we studied, the normalized frequency distributions are not uniformly distributed among the subgraph patterns. Therefore, *H*_3R_ and *H*_4R_ are different from one. We also note that the HIF-1 signaling pathway and PPAR signaling pathway have relatively small *H*_3R_ and *H*_4R_ values. This is likely because the transcription factors HIF-1 and PPAR function as master regulators of many genes. Therefore, the SIM subgraph is the dominant subgraph at both 3-node and 4-node levels. The median *H*_3R_ and *H*_4R_ values for the three network types range from 0.35 to 0.46 (Supplemental File 1–Table S17). This result suggests that the network subgraph distributions are not uniform, and that certain subgraph patterns have a higher occurrence probability.

### Association of network subgraphs and driver genes

The results of the association of network subgraphs and driver genes for cancer networks, STNs and cellular processes are given in Supplemental File 9. The odds ratios are listed in the last column. An odds ratio greater than one indicates that driver genes are enriched in the subgraph module (consisting of 3-node and 4-node subgraph genes, but the subgraphs are not necessarily interconnected). Among the 17 cancer networks, seven have an odds ratio greater than 1. For the 45 STNs, 21 have an odds ratio larger than 1. Among the nine cellular processes, six have an odds ratio greater than 1.

We noted that there are four networks have unusually large odds ratios (Supplemental File 9). These four networks are: “Calcium signaling pathway”, “Fc epsilon RI signaling pathway”, “Signaling pathways regulating pluripotency of stem cells” and “Regulation of actin cytoskeleton” (5.263, 5.000, 5.700 and 2.933, respectively). Large odds ratios indicate that the four networks are highly enriched with subgraph-associated driver genes. Calcium signaling proteins are related to cell migration, metastasis, and cancer progression (Faris et al., 2018; White, 2017). According to the “Oncology Drug Pipeline Project”, the Fc epsilon RI signaling pathway is a major target for cancer drug development (https://www.prnewswire.com/news-releases/fc-epsilon-ri-signaling-pathway-in-oncology-drug-pipeline-update-2015-300114859.html). Also, this pathway is the most significant interaction pathway in sorafenib-treated hepatocellular carcinoma (Liu et al., 2015). Regulation of pluripotent stem cells is critically related to oncogenesis (Katoh, 2011; Klein et al., 2018). A proposed anticancer therapy strategy involves killing cancer cells by suppressing the activity of pluripotency transcription factors (Liu, Yu & Liu, 2013). Regulation of the actin cytoskeleton plays important role in cancer cell migration, invasion (Lorente, Syriani & Morales, 2014; Yamaguchi & Condeelis, 2007), and epithelial‑mesenchymal transition (Shankar & Nabi, 2015; Sun et al., 2015). Our study enhances previous study findings by showing the importance of subgraph-associated driver genes.

## Discussion

Network subgraphs play an important role in biological networks. We used a rigorous mathematical and systematic approach—spectral graph theory—to characterize 3-node and 4-node network subgraphs. Further, we introduced nine graph energies and reciprocity to characterize network subgraphs. We stated in “Network subgraphs (N-node subgraphs) versus network motifs” that our analysis does not consider randomized version of subgraphs analysis. In case of using the randomization approach, different results were obtained. Description of the randomization steps and the results of the graph energies and reciprocity were given in Supplemental File 10. In addition, we characterized network complexity using two widely-accepted complexity measures, *CC* and *KC*. Chemical molecule complexity indices are useful measures that have been used in predictive pharmacology and toxicology (Basak, 1987) and to characterize the structural features of chemical structures (Randic & Plavsic, 2002; Yamaguchi, Aoki & Mamitsuka, 2003). Similar in spirit to Minoli (1976) and the previous three references, a possible application of *CC* and *KC* measures is to infer the hierarchical order of molecular network structures.

The concept of a unique identifier or descriptor was introduced to label network subgraphs. This novel idea combines four parameters—graph energy, reciprocity, *CC*, and eigenvalue spectrum—to characterize a network subgraph. Such molecular descriptors have been utilized to design inhibitors against Alzheimer’s disease (Gurung et al., 2017) and kidney cancer (Feng et al., 2010). A foreseeable application of our unique descriptor is to examine the transition between subgraphs and mutated subgraphs found in normal and disease networks. In disease states, the regulatory interactions among genetic elements may be disrupted or activated because of genetic mutation or epigenetic modification, resulting in different interactions among the nodes. In addition, driver mutations are likely enriched or depleted in certain subgraph positions, such as the source node of a subgraph (Awan et al., 2007; Carson et al., 2015). A source node is a node that has only outgoing edges. In other words, a mutated driver gene acts as an upstream regulator. Previous studies reported that certain subgraph positions, such as source and target nodes, are enriched in cancer-associated genes. To examine this issue, cancer-specific gene mutation data (provided by the GDC-TCGA database) can be combined with subgraph node identities. Such work will be performed in our next study.

Some 3-node and 4-node subgraphs are interconnected through shared genetic elements. These modules are reported in our previous work (Hsieh et al., 2015). Interconnected subgraphs can be merged to form higher-order network structures. Indeed, coupled subgraphs perform specific functions. For instance, coupled FBLs form dynamic motifs that are often found in cellular networks (Kim, Yoon & Cho, 2008) and show oscillatory behavior (Tsai et al., 2008).

We extended our algorithm, *PatternFinder*, to identify complete sets of 3-node and 4-node subgraphs for 17 cancer networks, 45 STNs and nine cellular processes. Except for a few networks, 3-node and 4-node subgraphs account for more than 70% of the nodes in the studied networks. Additionally, our study revealed the following features: (1) the relative entropies of the subgraph distributions are not equal or close to one, indicating that the identified subgraphs are not uniformly distributed among the 13 and 199 patterns; (2) molecular biological networks are built from a finite number of subgraph patterns and certain subgraphs with large graph energies are not present. Hence, each network can be characterized by a graph energy cutoff; (3) *irreducible* subgraph patterns are the most frequently-observed subgraphs. For instance, the cascade pattern is the most frequently found subgraph, followed by the SIM and the MIM subgraphs; and (4) the three network families exhibit the above features, suggesting that there is a universal organization principle determining the underlying network architecture.

## Conclusions

In conclusion, this study provides a systematic and rigorous approach to dissecting the underlying structure of biological molecular networks. SGT serves as a powerful approach to distinguish different subgraph topologies or connectivity patterns, which inspired our hypothesis that network structures can be understood in terms of 3-node and 4-node subgraphs. The next step is to test our hypothesis by analyzing 5-node subgraphs (Zaenudin et al., 2019 ). We expect that our efforts will further elucidate the complex nature of molecular networks.

## Supplemental Information

10.7717/peerj.9556/supp-1 Supplemental Information 1 MATLAB code.

10.7717/peerj.9556/supp-2 Supplemental Information 2 Methods.

10.7717/peerj.9556/supp-3 Supplemental Information 3 Figure 3-and 4-nodes.

10.7717/peerj.9556/supp-4 Supplemental Information 4 Figure from acc-motif 3-and 4-nodes.

10.7717/peerj.9556/supp-5 Supplemental Information 5 Functional subgraph.

10.7717/peerj.9556/supp-6 Supplemental Information 6 Reciprocal Links for 3-node.

10.7717/peerj.9556/supp-7 Supplemental Information 7 Energy and eigenvectors.

10.7717/peerj.9556/supp-8 Supplemental Information 8 Reciprocal Links for 4-node.

10.7717/peerj.9556/supp-9 Supplemental Information 9 CC and KC 4-node.

10.7717/peerj.9556/supp-10 Supplemental Information 10 The results of driver genes embedded in subgraph module and non-subgraph module. A pseudo-count was added if the number of embedded is zero, because the odds ratio is not well-defined. Odds ratio and total number of genes are listed in the last column. An odds ratio greater than one implies that driver genes are enriched in subgraph module.

10.7717/peerj.9556/supp-11 Supplemental Information 11 The theoretical results of the mean and standard deviation of the nine graph energies and reciprocity for the 3-node subgraphs after using the randomization concept. Table R1 summarized the results of subgraphs associated with two to six edges, the in-degree and out-degree information. Table R2 summarized the theoretical results of the statistical average and standard deviation of the graph energies for the 3-node subgraphs. Table R3 summarized the theoretical results of the statistical average and standard deviation of the reciprocity for the 3-node subgraphs after using the randomization concept.

10.7717/peerj.9556/supp-12 Supplemental Information 12 Specific data links used from the KEGG PATHWAY Database.

## Additional Information and Declarations

### Data Availability

The following information was supplied regarding data availability: The program coding files are available in the Supplemental Files. We used publicly available raw data from https://www.genome.jp/kegg/pathway.html#environmental (specific links are available in the Supplemental Files).
