# Supplementary material for: Dissecting molecular network structures using a network subgraph approach
Source: PeerJ. 2020 Aug 6;8:e9556. doi: 10.7717/peerj.9556 (PMC7512139; doi:10.7717/peerj.9556)
Supplement: Supplemental Information 2 [file peerj-08-9556-s002.docx]

**Supplementary File 1**

**Dissecting molecular network structures using a network subgraph approach**

**Methods**

Supplementary File 1 – Supplementary Table S1 summarizes the nodes, edges and subgraph-associated node information for the 17 cancer networks. Besides the ‘Hepatocellular carcinoma’ and ‘Prostate cancer’ networks, the percentages of nodes associated with subgraphs are more than 70%. The results suggest that genes embedded in the 3-node subgraphs and 4-node subgraphs account for the major portion of the cancer networks.

We noticed that a few of the networks’ KGML files have missing information. Feedback loop information is not recorded in the KGML, but it is displayed on the KEGG webpage. For instance, the ‘Signaling pathways regulating pluripotency of stem cells’ pathway consists of the feedback regulatory relations among three genes, *Nanog, Oct4* and *Sox2.* The KGML file provides the regulatory relations among *Oct4 🡪 Nanog 🡪 Sox2* but not *Nanog 🡪 Oct4 🡪 Sox2.* To remedy this problem, we have to examine each analyzed network manually, and insert the missing information back in the KGML files.

Supplementary Table S1. List of the nodes, edges and subgraph-associated nodes information for the 17 cancer networks we studied.

| Cancer networks | nodes | edges | subgraph-associated nodes |
| --- | --- | --- | --- |
| Acute myeloid leukemia [hsa05221] | 43 | 53 | 100% |
| Basal cell carcinoma [hsa05217] | 23 | 20 | 91.3% |
| Breast cancer [hsa05224] | 63 | 60 | 81.0% |
| Choline metabolism in cancer [hsa05231] | 37 | 34 | 73.0% |
| Chronic myeloid leukemia [hsa05220] | 47 | 39 | 78.7% |
| Colorectal cancer [hsa05210] | 48 | 39 | 77.1% |
| Endometrial cancer [hsa05213] | 37 | 27 | 78.4% |
| Gastric cancer [hsa05226] | 73 | 51 | 71.2% |
| Glioma [hsa05214] | 36 | 36 | 86.1% |
| Hepatocellular carcinoma [hsa05225] | 82 | 49 | 64.6% |
| Melanoma [hsa05218] | 28 | 25 | 89.3% |
| Non-small cell lung cancer [hsa05223] | 43 | 43 | 83.7% |
| Pancreatic cancer [hsa05212] | 50 | 46 | 92.0% |
| Pathways in cancer [hsa05200] | 152 | 197 | 94.7% |
| Prostate cancer [hsa05215] | 53 | 38 | 67.9% |
| Renal cell carcinoma [hsa05211] | 40 | 27 | 72.5% |
| Small cell lung cancer [hsa05222] | 48 | 34 | 75.0% |

Supplementary Table S2. List of nodes, edges and subgraph-associated nodes information for the 46 STNs we studied.

| STN | nodes | edges | subgraph-associated nodes |
| --- | --- | --- | --- |
| Adipocytokine signaling pathway [hsa04920] | 36 | 43 | 91.7% |
| AMPK signaling pathway [hsa04152] | 61 | 52 | 75.4% |
| Apelin signaling pathway [hsa04371] | 55 | 56 | 85.5% |
| B cell receptor signaling pathway [hsa04662] | 46 | 44 | 73.9% |
| Calcium signaling pathway [hsa04020] | 44 | 28 | 54.5% |
| cAMP signaling pathway [hsa04024] | 74 | 71 | 90.5% |
| cGMP-PKG signaling pathway [hsa04022] | 59 | 52 | 83.1% |
| Chemokine signaling pathway [hsa04062] | 51 | 58 | 94.1% |
| C-type lectin receptor signaling pathway [hsa04625] | 75 | 90 | 96.0% |
| ErbB signaling pathway [hsa04012] | 53 | 82 | 98.1% |
| Estrogen signaling pathway [hsa04915] | 34 | 33 | 88.2% |
| Fc epsilon RI signaling pathway [hsa04664] | 40 | 35 | 75.0% |
| FoxO signaling pathway [hsa04068] | 74 | 69 | 91.9% |
| Glucagon signaling pathway [hsa04922] | 45 | 38 | 68.9% |
| GnRH signaling pathway [hsa04912] | 40 | 38 | 90.0% |
| Hedgehog signaling pathway [hsa04340] | 23 | 35 | 100.0% |
| HIF-1 signaling pathway [hsa04066] | 62 | 53 | 83.9% |
| Hippo signaling pathway [hsa04390] | 78 | 60 | 76.9% |
| Insulin signaling pathway [hsa04910] | 63 | 69 | 87.3% |
| Jak-STAT signaling pathway [hsa04630] | 34 | 34 | 97.1% |
| MAPK signaling pathway [hsa04010] | 115 | 163 | 98.3% |
| mTOR signaling pathway [hsa04150] | 67 | 72 | 83.6% |
| Neurotrophin signaling pathway [hsa04722] | 74 | 112 | 95.9% |
| NF-kappa B signaling pathway [hsa04064] | 101 | 76 | 69.3% |
| NOD-like receptor signaling pathway [hsa04621] | 114 | 131 | 84.2% |
| Notch signaling pathway [hsa04330] | 27 | 16 | 63.0% |
| Oxytocin signaling pathway [hsa04921] | 52 | 56 | 88.5% |
| p53 signaling pathway [hsa04115] | 53 | 57 | 88.1% |
| Phosphatidylinositol signaling system [hsa04070] | 30 | 60 | 90.0% |
| Phospholipase D signaling pathway [hsa04072] | 51 | 45 | 84.3% |
| PI3K-Akt signaling pathway [hsa04151] | 90 | 77 | 76.7% |
| PPAR signaling pathway [hsa03320] | 51 | 55 | 98.0% |
| Prolactin signaling pathway [hsa04917] | 39 | 44 | 97.4% |
| Rap1 signaling pathway [hsa04015] | 81 | 88 | 81.5% |
| Ras signaling pathway [hsa04014] | 68 | 96 | 95.6% |
| Relaxin signaling pathway [hsa04926] | 55 | 68 | 89.1% |
| RIG-I-like receptor signaling pathway [hsa04622] | 56 | 40 | 58.9% |
| Sphingolipid signaling pathway [hsa04071] | 55 | 51 | 81.8% |
| T cell receptor signaling pathway [hsa04660] | 67 | 67 | 76.1% |
| TGF-beta signaling pathway [hsa04350] | 50 | 38 | 74.0% |
| Thyroid hormone signaling pathway [hsa04919] | 65 | 56 | 75.4% |
| TNF signaling pathway [hsa04668] | 77 | 48 | 54.5% |
| Toll-like receptor signaling pathway (hsa 04620) | 73 | 91 | 90.0% |
| VEGF signaling pathway [hsa04370] | 28 | 32 | 100.0% |
| Wnt signaling pathway [hsa04310] | 69 | 84 | 95.7% |

Supplementary Table S3. List of the nodes, edges and subgraph-associated nodes information for the nine cellular processes we studied.

| Cell cycle | nodes | edges | subgraph-associated nodes |
| --- | --- | --- | --- |
| Adherens junction [hsa04520] | 83 | 80 | 84.6% |
| Apoptosis [hsa04210] | 88 | 132 | 89.8% |
| Cell cycle [hsa04110] | 95 | 79 | 61.1% |
| Cellular senescence [hsa04218] | 87 | 104 | 78.2% |
| Focal adhesion [hsa04510] | 61 | 101 | 98.4% |
| Gap junction [hsa04540] | 34 | 56 | 100.0% |
| Necroptosis [hsa04217] | 66 | 77 | 89.4% |
| Regulation of actin cytoskeleton [hsa04810] | 68 | 75 | 82.4% |
| Signaling pathways regulating pluripotency of stem cells [hsa04550] | 67 | 64 | 58.2% |

Supplementary Table S4. Subgraph identification tool – the *PatternFinder* algorithm

| There are many network motif detection tools, such as, FANMOD, MAVISTO, MFINDER, NetMatch and SNAVI. As we have reported that those tools have a few limitations^1^ on motif identification: (i) the motifs may not be recoverable due to the use of randomize algorithm, and (ii) nodes’ identities were missing.  An algorithm named *PatternFinder* was developed to identify: (i) functional subgraphs embedded in the 3-node subgraphs and 4-node subgraphs, and (ii) subgraphs compose of three nodes and four nodes in a network^2^. The advantage of *PatternFinder* is its ability to identify subgraphs that are not identified by MFINDER. As shown in Supplementary Figure 1, given the ”Input network”, *PatternFinder* is able to identify two four-node subgraphs, i.e. subgraph ‘id_904’ and subgraph ‘id_906’, whereas MFINDER can identify subgraph ‘id_906’ only. It is because MFINDER recognizes subgraph ‘id_904’ is a subgraph of subgraph ‘id_906’. In other words, *PatternFinder* is able to identify subgraphs embedded in a subgraph.  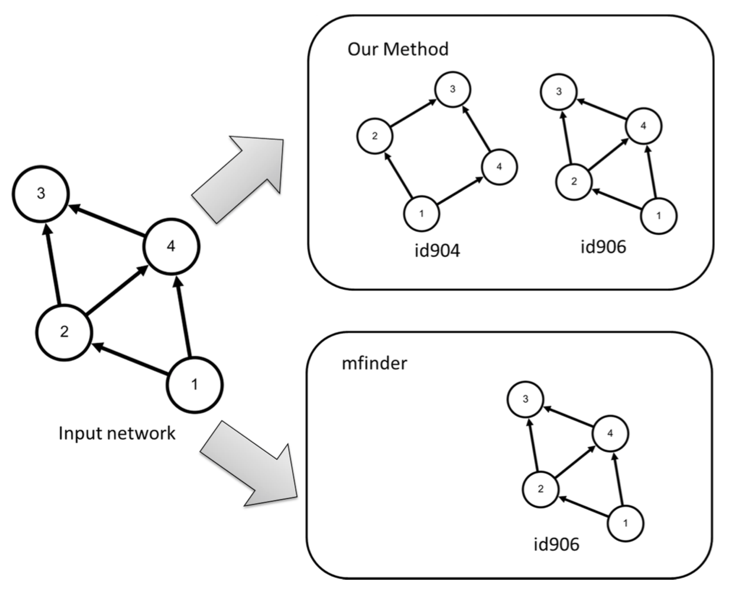  Supplementary Figure 1. A comparison of two subgraph identification algorithms: *mfinder* and *PatternFinder. PatternFinder* was designed to identify subgraphs within a subgraph.  In the following, a 4-node subgraph is used as an example to illustrate the basic concept behind the *PatternFinder* algorithm. Given a network called ‘*net’* composes of 20 nodes, an adjacency matrix can be constructed. Let *n* denotes the total number of nodes. Assuming that we want to identify a subgraph which is denoted by the integer ‘2204’, then *PatternFinder* read in the ‘2204’ pattern. This subgraph composes of four nodes and five edges, where the edges are denoted by $\boldsymbol{t}_{\boldsymbol{0}}$, $\boldsymbol{t}_{\boldsymbol{1}}$, $\boldsymbol{t}_{\boldsymbol{2}}$, $\boldsymbol{tr}_{\boldsymbol{0}}$ and $\boldsymbol{tr}_{\boldsymbol{1}}$. Starting from node A, *PatternFinder* begins to examine the following patterns: (i) is node A and node B connect with an edge $\boldsymbol{t}_{\boldsymbol{0}}$, (ii) is node B and node C connect with an edge $\boldsymbol{t}_{\boldsymbol{1}}$, (iii) is node C and node D connect with an edge $\boldsymbol{t}_{\boldsymbol{2}}$, and (iv) is node D and node A connect with an edge $\mathbf{t}_{\mathbf{0}}$ $\mathbf{t}_{\mathbf{0}}$, and node D and node B connect with an edge $\boldsymbol{tr}_{\boldsymbol{1}}$.  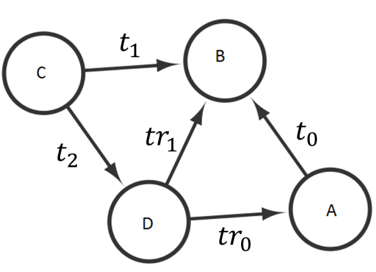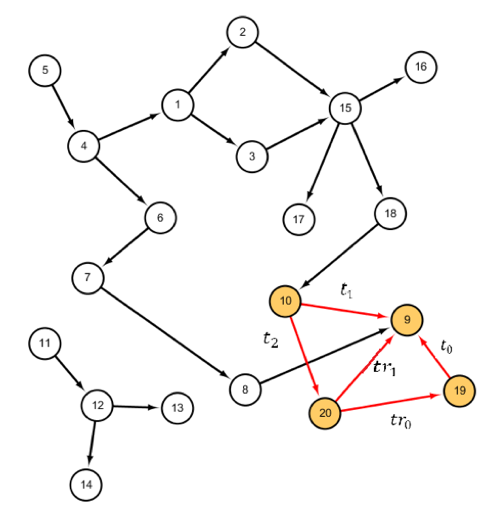  Supplementary Figure 2. (a) an input network named ‘*net’* composes of 20 nodes, where nodes 9, 10, 19 and 20 form a 4-node subgraph and (b) a 4-node subgraph with an accession ID ‘id_2204’  Starting from the network named ‘*net’*, the algorithm begins the search from node 1 and labels it as node A. Node 1 and node 2 or node B are linked, the edge is denoted by *edge*(1, 2). The algorithm continues to search if there is a node links to node B, if not, the algorithm will relabel node B to node 3 and repeat the search. From Supplementary Figure 2, it was found that A=19, B=9, C=10, and D=20 are connected by three edges, i.e. *edge*(19,9)=$\boldsymbol{t}_{\boldsymbol{0}}$ , *edge*(9,10)=$\boldsymbol{t}_{\boldsymbol{1}}$, *edge*(10,20)=$\boldsymbol{t}_{\boldsymbol{2}}$ , hence, four nodes are identified. However, according to the ‘id_2204’ subgraph, there are two more edges need to be determined, i.e. *edge*(20,9)=$\boldsymbol{tr}_{\boldsymbol{1}}$ and *edge*(20,19)=$\boldsymbol{tr}_{\boldsymbol{2}}$. The computational time complexity of the algorithm *PatternFinder* is O(*n^4^*).  References   1. Hsieh, W. T. et al. Transcription factor and microRNA-regulated network motifs for cancer and signal transduction networks. BMC Systems Biology. 9(1), S5 (2015). 2. Li, H. X. In silico study of significant network motifs in the cancer networks. Master Thesis, National Formosa University, Taiwan (2016). Advisor: Huang, C. H., co-advisor: Ng, K. L. |
| --- |

Supplementary Table S5. Algorithm for finding a unique identifier for the 3-node subgraphs

| The following algorithm is used to derive the subsets of parameters (energy parameters in *ES,* *r* and *CC*), which could separate the most number of subgraph types. In this algorithm, *ES* denotes the set of the graph energy parameters.  Input:  *ES*={*E_1_*, *E_2_*, … , *E_6_*}, parameter *r*, parameter *CC* and all of the thirteen 3-node subgraphs;  Output:  Fingerprint parameter set *MS* for the 3-node subgraph;  Step 1: For each parameter (energy parameter in *ES*, parameters *r* or *CC*), generate 13 sets corresponding to the 13 subgraph types; that is, one set corresponds to one subgraph type;  Step 2: For each parameter, calculate the corresponding values for all of the 13 subgraph types, and merge the corresponding sets generated in Step 1 into one set if they have the same computed value (this means the corresponding subgraph types cannot be distinguished by this parameter);  Step 3: *MS*={*CC*};  Step 4: For the current sets of parameter *CC*, if any set has more than one element (subgraph type) and some of the corresponding subgraph types can be distinguished by parameter *r*, then divide the set into the corresponding disjoint sets;  Step 5: *MS*=*MS*$\cup\{r\}$;  Step 6: Find the element in *ES* called *E_i_*, which can separate the highest number of sets for the current sets of parameter *CC*; return *MS*=*MS*$\cup\{E_{i}\}$; |
| --- |

Supplementary Table S6. The results of the lower (*E_min_*) and upper (*E_max_*) bounds of the nine graph energies and ratios for the 3-node subgraphs and 4-node subgraphs.

|  |  | *E* | *LE* | *QE* | *AA^t^* | *LL^t^* | *QQ^t^* | *AL^t^* | *AQ^t^* | *LQ^t^* |
| --- | --- | --- | --- | --- | --- | --- | --- | --- | --- | --- |
| 3-node | *E_min_* | 0.00 | 2.67 | 2.67 | 1.41 | 4.32 | 4.32 | 1.41 | 1.41 | 3.83 |
|  | *E_max_* | 4.00 | 8.00 | 8.00 | 4.00 | 12.00 | 12.00 | 6.47 | 6.93 | 11.21 |
|  | *ratio* | 2.00 * | 3.00 | 3.00 | 3.05 | 2.78 | 2.78 | 4.59 | 4.91 | 2.93 |
| 4-node | *E_min_* | 0.00 | 4.50 | 4.50 | 1.73 | 6.36 | 6.36 | 1.73 | 1.73 | 5.61 |
|  | *E_max_* | 6 | 18 | 18 | 6 | 24 | 24 | 10.94 | 11.9 | 22.94 |
|  | *ratio* | 3.00 | 4.00 | 4.00 | 3.47 | 3.77 | 3.77 | 6.32 | 6.88 | 4.09 |

The symbol ‘*’ means *E_min_* is given by the second smallest graph energy *E*

Supplementary Table S7. The results of the correlation strength (*SRCC*) between graph complexity and graph energy for the 3-node subgraphs and 4-node subgraphs.

|  |  | *E* | *LE* | *QE* | *AA^t^* | *LL^t^* | *QQ^t^* | *AL^t^* | *AQ^t^* | *LQ^t^* |
| --- | --- | --- | --- | --- | --- | --- | --- | --- | --- | --- |
| 3-node | *CC* | -0.003 | 0.224 | 0.171 | 0.032 | ***0.270^*^*** | 0.188 | -0.023 | 0.062 | 0.257 |
|  | *KC* | 0.484 | 0.685 | 0.698 | 0.617 | ***0.779^*^*** | 0.748 | 0.359 | 0.604 | 0.773 |
| 4-node | *CC* | 0.332 | 0.630 | 0.614 | 0.453 | ***0.686^*^*** | 0.651 | 0.384 | 0.452 | 0.670 |
|  | *KC* | 0.602 | 0.764 | 0.766 | 0.712 | 0.771 | 0.782 | 0.648 | 0.684 | ***0.773^*^*** |

***^*^*** Bold-faced and italic numbers denote the maximum *SRCC* for 3-node subgraphs and 4-node subgraphs among the nine energies.

Supplementary Table S8. The results of the correlation strength (including minimum, maximum and ranges) between graph complexity and graph energy for the 3-node subgraphs and 4-node subgraphs.

|  |  | minimum | maximum | range |
| --- | --- | --- | --- | --- |
| 3-node | (*CC*, energy) | -0.023 | 0.270 | 0.293 |
|  | (*KC*, energy) | 0.359 | 0.779 | 0.420 |
| 4-node | (*CC*, energy) | 0.332 | 0.686 | 0.354 |
|  | (*KC*, energy) | 0.602 | 0.782 | 0.180 |

Supplementary Table S9. The top seven most frequently found 3-node subgraphs and 4-node subgraphs for cancer networks.

|  | ID | average probability | *r* | Name, embedded subgraph ID |
| --- | --- | --- | --- | --- |
| 3-node |  |  |  |  |
| 1 | 6 | 0.421 | -1/2 | SIM |
| 2 | 12 | 0.414 | -1/2 | Cascade |
| 3 | 36 | 0.152 | -1/2 | MIM |
| 4 | 38 | 0.0091 | -1 | FFL, 12, 36 |
| 5 | 74 | 0.0022 | 1/3 | 12 |
| 6 | 14 | 0.0016 | 1/3 | 6 |
| 7 | 98 | 0.00092 | -1 | 3-cycle, 12 |
| 4-node |  |  |  |  |
| 1 | 14 | 0.224 | -1/3 | SIM |
| 2 | 328 | 0.158 | -1/3 | Cascade |
| 3 | 28 | 0.148 | -1/3 | - |
| 4 | 74 | 0.137 | -1/3 | - |
| 5 | 76 | 0.100 | -1/3 | MIM |
| 6 | 392 | 0.099 | -1/3 | - |
| 7 | 280 | 0.0864 | -1/3 | - |

SIM denotes simple input module, MIM denotes multiple input module, FFL denotes feed-forward loop

Supplementary Table S10. The top seven most frequently found 3-node subgraphs and

4-node subgraphs for STNs.

| rank | ID | average probability | *r* | Name & embedded subgraph ID |
| --- | --- | --- | --- | --- |
| 3-node |  |  |  |  |
| 1 | 12 | 0.402 | -1/2 | Cascade |
| 2 | 6 | 0.380 | -1/2 | SIM |
| 3 | 36 | 0.194 | -1/2 | MIM |
| 4 | 38 | 0.0154 | -1 | FFL, 12, 36 |
| 5 | 14 | 0.00360 | 1/3 | 6 |
| 6 | 74 | 0.00250 | 1/3 | 12, 36 |
| 7 | 98 | 0.00138 | -1 | 12 |
| 4-node |  |  |  |  |
| 1 | 14 | 0.260 | -1/3 | SIM |
| 2 | 28 | 0.150 | -1/3 | - |
| 3 | 74 | 0.127 | -1/3 | null |
| 4 | 328 | 0.112 | -1/3 | Cascade |
| 5 | 280 | 0.103 | -1/3 | - |
| 6 | 76 | 0.0932 | -1/3 | MIM |
| 7 | 392 | 0.0908 | -1/3 | - |

Supplementary Table S11. The top seven most frequently found 3-node subgraphs and 4-node subgraphs for cellular processes.

| rank | ID | average probability | *r* | Name & embedded subgraph ID |
| --- | --- | --- | --- | --- |
| 3-node |  |  |  |  |
| 1 | 12 | 0.443 | -1/2 | Cascade |
| 2 | 36 | 0.316 | -1/2 | MIM |
| 3 | 6 | 0.183 | -1/2 | SIM |
| 4 | 38 | 0.0206 | -1 | FFL, 12, 36 |
| 5 | 74 | 0.0167 | 1/3 | 12, 36 |
| 6 | 14 | 0.00790 | 1/3 | 6 |
| 7 | 46 | 0.00250 | -1/2 | 6, 12, 14, 36, 38 |
| 4-node |  |  |  |  |
| 1 | 392 | 0.159 | -1/3 | - |
| 2 | 328 | 0.145 | -1/3 | Cascade |
| 3 | 76 | 0.121 | -1/3 | MIM |
| 4 | 280 | 0.120 | -1/3 | - |
| 5 | 2184 | 0.115 | -1/3 | MIM |
| 6 | 74 | 0.0990 | -1/3 | - |
| 7 | 28 | 0.0861 | -1/3 | - |

Supplementary Table S12. The results of the cutoffs, maximum graph energies and ratios for the 3-node subgraph and 4-node subgraph found in the 17 cancer networks.

|  | energy | *E* | *LE* | *QE* | *AA^t^* | *LL^t^* | *QQ^t^* | *AL^t^* | *AQ^t^* | *LQ^t^* |
| --- | --- | --- | --- | --- | --- | --- | --- | --- | --- | --- |
| 3-node | cutoff | 3 | 4.292 | 4.464 | 3 | 6.292 | 6.464 | 4.252 | 4.363 | 6.012 |
|  | max | 4 | 8 | 8 | 4 | 12 | 12 | 6.472 | 6.928 | 11.21 |
|  | ratio | 0.750 | 0.537 | 0.558 | 0.750 | 0.524 | 0.539 | 0.657 | 0.630 | 0.536 |
| 4-node | cutoff | 4.00 | 9.00 | 9.00 | 4.00 | 12.54 | 12.44 | 5.723 | 5.723 | 12 |
|  | max | 6 | 18 | 18 | 6 | 24 | 24 | 10.94 | 11.9 | 22.94 |
|  | ratio | 0.667 | 0.500 | 0.500 | 0.667 | 0.523 | 0.518 | 0.523 | 0.481 | 0.523 |

ratio = cutoff / max

Supplementary Table S13. The results of the cutoffs, maximum graph energies and ratios for the 3-node subgraph and 4-node subgraph found in the 46 STNs.

|  |  | *E* | *LE* | *QE* | *AA^t^* | *LL^t^* | *QQ^t^* | *AL^t^* | *AQ^t^* | *LQ^t^* |
| --- | --- | --- | --- | --- | --- | --- | --- | --- | --- | --- |
| 3-node | cutoff | 3.062 | 5.333 | 5.56 | 3.236 | 8.243 | 8.247 | 4.762 | 5.022 | 7.586 |
|  | max | 4 | 8 | 8 | 4 | 12 | 12 | 6.472 | 6.928 | 11.21 |
|  | ratio | 0.766 | 0.667 | 0.695 | 0.809 | 0.687 | 0.687 | 0.736 | 0.725 | 0.677 |
| 4-node | cutoff | 4.395 | 10.58 | 10.64 | 4.705 | 14.23 | 14.23 | 7.453 | 7.73 | 13.68 |
|  | max | 6 | 18 | 18 | 6 | 24 | 24 | 10.94 | 11.9 | 22.94 |
|  | ratio | 0.733 | 0.588 | 0.591 | 0.784 | 0.593 | 0.593 | 0.681 | 0.650 | 0.596 |

ratio = cutoff / max

Supplementary Table S14. The results of the cutoffs, maximum graph energies and ratios for the 3-node subgraph and 4-node subgraph found in the nine cellular processes.

|  |  | *E* | *LE* | *QE* | *AA^t^* | *LL^t^* | *QQ^t^* | *AL^t^* | *AQ^t^* | *LQ^t^* |
| --- | --- | --- | --- | --- | --- | --- | --- | --- | --- | --- |
| 3-node | cutoff | 4 | 8 | 8 | 4 | 12 | 12 | 6.472 | 6.928 | 11.21 |
|  | max | 4 | 8 | 8 | 4 | 12 | 12 | 6.472 | 6.928 | 11.21 |
|  | ratio | 1 | 1 | 1 | 1 | 1 | 1 | 1 | 1 | 1 |
| 4-node | cutoff | 4.116 | 10.5 | 10.5 | 4.36 | 14.26 | 14.21 | 6.815 | 7.07 | 13.28 |
|  | max | 6 | 18 | 18 | 6 | 24 | 24 | 10.94 | 11.9 | 22.94 |
|  | ratio | 0.686 | 0.583 | 0.583 | 0.727 | 0.594 | 0.592 | 0.623 | 0.594 | 0.579 |

ratio = cutoff / max

Supplementary Table S15. The results of *N_3_*, *N_4_*, *H_3R_* and *H_4R_* for 46 STNs.

| STN | *N_3_* | *H_3R_* | *N_4_* | *H_4R_* |
| --- | --- | --- | --- | --- |
| Adipocytokine_signaling_pathway_[hsa04920] | 118 | 0.426 | 386 | 0.414 |
| AMPK_signaling_pathway_[hsa04152] | 262 | 0.345 | 1830 | 0.308 |
| Apelin_signaling_pathway_[hsa04371] | 127 | 0.372 | 358 | 0.351 |
| B_cell_receptor_signaling_pathway_[hsa04662] | 96 | 0.419 | 311 | 0.411 |
| Calcium_signaling_pathway_[hsa04020] | 60 | 0.406 | 124 | 0.382 |
| cAMP_signaling_pathway_[hsa04024] | 353 | 0.217 | 2289 | 0.142 |
| cGMP-PKG_signaling_pathway_[hsa04022] | 202 | 0.290 | 1002 | 0.209 |
| Chemokine_signaling_pathway_[hsa04062] | 144 | 0.412 | 508 | 0.391 |
| C-type_lectin_receptor_signaling_pathway_[hsa04625] | 239 | 0.486 | 716 | 0.449 |
| ErbB_signaling_pathway_[hsa04012] | 375 | 0.442 | 2054 | 0.433 |
| Estrogen_signaling_pathway_[hsa04915] | 49 | 0.365 | 97 | 0.332 |
| Fc_epsilon_RI_signaling_pathway_[hsa04664] | 87 | 0.451 | 321 | 0.427 |
| FoxO_signaling_pathway_[hsa04068] | 902 | 0.389 | 12174 | 0.268 |
| Glucagon_signaling_pathway_[hsa04922] | 78 | 0.326 | 182 | 0.270 |
| GnRH_signaling_pathway_[hsa04912] | 76 | 0.323 | 212 | 0.315 |
| Hedgehog_signaling_pathway_[hsa04340] | 147 | 0.518 | 752 | 0.484 |
| HIF-1_signaling_pathway_[hsa04066] | 407 | **0.120** | 3382 | **0.034** |
| Hippo_signaling_pathway_[hsa04390] | 162 | 0.446 | 511 | 0.371 |
| Insulin_signaling_pathway_[hsa04910] | 188 | 0.452 | 659 | 0.431 |
| Jak-STAT_signaling_pathway_[hsa04630] | 179 | 0.398 | 894 | 0.318 |
| MAPK_signaling_pathway_[hsa04010] | 745 | 0.451 | 4280 | 0.436 |
| mTOR_signaling_pathway_[hsa04150] | 209 | 0.483 | 801 | 0.488 |
| Neurotrophin_signaling_pathway_[hsa04722] | 525 | 0.432 | 2957 | 0.427 |
| NF-kappa_B_signaling_pathway_[hsa04064] | 304 | 0.443 | 1586 | 0.325 |
| NOD-like_receptor_signaling_pathway_[hsa04621] | 566 | 0.454 | 2851 | 0.426 |
| Notch_signaling_pathway_[hsa04330] | 64 | 0.344 | 196 | 0.310 |
| Oxytocin_signaling_pathway_[hsa04921] | 154 | 0.376 | 456 | 0.373 |
| p53_signaling_pathway_[hsa04115] | 710 | 0.301 | 9841 | 0.217 |
| Phosphatidylinositol_signaling_system_[hsa04070] | 290 | **0.652** | 1892 | **0.625** |
| Phospholipase_D_signaling_pathway_[hsa04072] | 106 | 0.388 | 264 | 0.386 |
| PI3K-Akt_signaling_pathway_[hsa04151] | 308 | 0.403 | 1956 | 0.364 |
| PPAR_signaling_pathway_[hsa03320] | 968 | 0.643 | 4608 | 0.159 |
| Prolactin_signaling_pathway_[hsa04917] | 170 | 0.380 | 884 | 0.345 |
| Rap1_signaling_pathway_[hsa04015] | 570 | 0.485 | 5707 | 0.397 |
| Ras_signaling_pathway_[hsa04014] | 479 | 0.413 | 3337 | 0.412 |
| Relaxin_signaling_pathway_[hsa04926] | 170 | 0.395 | 558 | 0.392 |
| RIG-I-like_receptor_signaling_pathway_[hsa04622] | 101 | 0.442 | 355 | 0.427 |
| Sphingolipid_signaling_pathway_[hsa04071] | 115 | 0.405 | 301 | 0.400 |
| T_cell_receptor_signaling_pathway_[hsa04660] | 176 | 0.487 | 676 | 0.478 |
| TGF-beta_signaling_pathway_[hsa04350] | 75 | 0.414 | 170 | 0.392 |
| Thyroid_hormone_signaling_pathway_[hsa04919] | 207 | 0.338 | 848 | 0.344 |
| TNF_signaling_pathway_[hsa04668] | 84 | 0.350 | 172 | 0.343 |
| Toll-like receptor signaling pathway [hsa04620] | 146 | 0.460 | 409 | 0.457 |
| VEGF_signaling_pathway_[hsa04370] | 84 | 0.376 | 278 | 0.358 |
| Wnt_signaling_pathway_[hsa04310] | 426 | 0.448 | 2529 | 0.435 |

Supplementary Table S16. The results of *N_3_*, *N_4_*, *H_3R_* and *H_4R_* for nine cellular processes.

| Cellular processes | *N_3_* | *H_3R_* | *N_4_* | *H_4R_* |
| --- | --- | --- | --- | --- |
| Adherens_junction_[hsa04520] | 362 | 0.539 | 2273 | 0.524 |
| Apoptosis_[hsa04210] | 567 | 0.463 | 3214 | 0.450 |
| Cell_cycle_[hsa04110] | 205 | 0.419 | 780 | 0.402 |
| Cellular_senescence_[hsa04218] | 186 | 0.408 | 595 | 0.369 |
| Focal_adhesion_[hsa04510] | 359 | 0.474 | 1759 | 0.455 |
| Gap_junction_[hsa04540] | 97 | **0.361**^*^ | 303 | **0.336^*^** |
| Necroptosis_[hsa04217] | 336 | 0.556 | 2471 | 0.525 |
| Regulation_of_actin_cytoskeleton_[hsa04810] | 193 | 0.449 | 727 | 0.409 |
| Signaling_pathways_regulating_pluripotency_of_stem_cells_[hsa04550] | 85 | **0.614 ^¶^** | 182 | **0.532^¶^** |

The symbols **^*^** and ^¶^ denote the minimum and maximum of normalized Shannon entropy respectively.

Supplementary Table S17. The ranges of *H_3R_* and *H_4R_* for cancer networks, STNs and cellular processes.

| Network type | range of *H_3R_* | range of *H_4R_* |
| --- | --- | --- |
| cancer networks | 0.323 ~ 0.493 = 0.170 | 0.273 ~ 0.483 = 0.210 |
| STN | 0.120 ~ 0.652 = 0.532 | 0.034 ~ 0.625 = 0.622 |
| cellular processes | 0.361 ~ 0.614 = 0.253 | 0.336 ~ 0.532 = 0.196 |
