# Supplementary material for: Dissecting molecular network structures using a network subgraph approach
Source: PeerJ. 2020 Aug 6;8:e9556. doi: 10.7717/peerj.9556 (PMC7512139; doi:10.7717/peerj.9556)
Supplement: Supplemental Information 4 [file peerj-08-9556-s004.pdf]

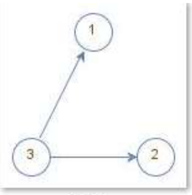

1120.jpeg

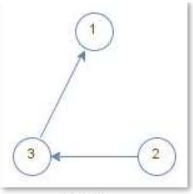

11011.jpeg

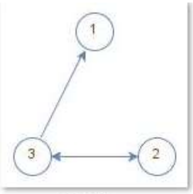

11121.jpeg

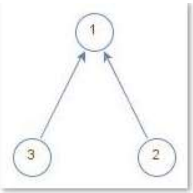

21010.jpeg

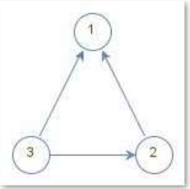

21120.jpeg

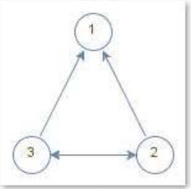

22121.jpeg

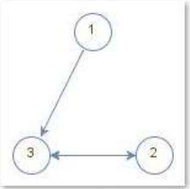

101112.jpeg

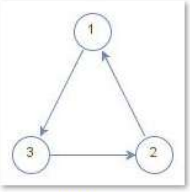

111111.jpeg

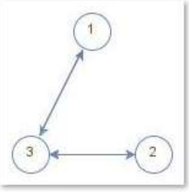

111122.jpeg

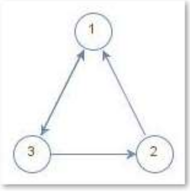

111221.jpeg

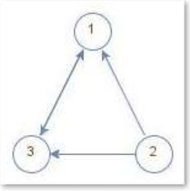

121220.jpeg

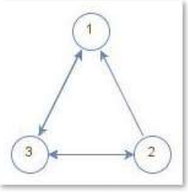

122122.jpeg

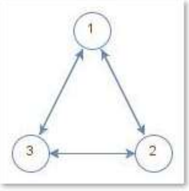

222222.jpeg

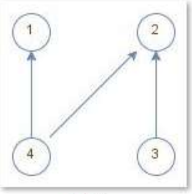

4118.jpeg

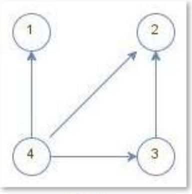

4119.jpeg

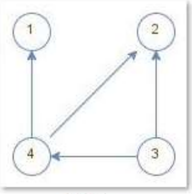

4126.jpeg

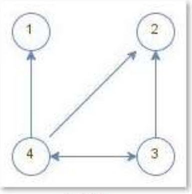

4127.jpeg

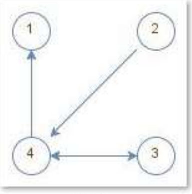

4173.jpeg

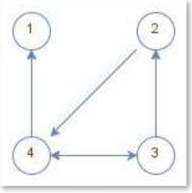

4189.jpeg

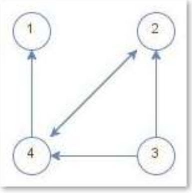

4190.jpeg

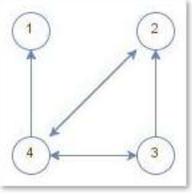

4191.jpeg

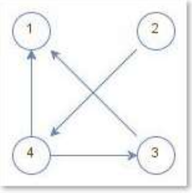

4197.jpeg

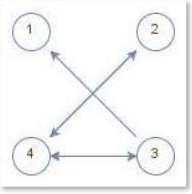

4203.jpeg

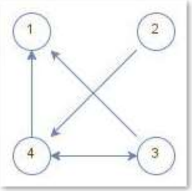

4205.jpeg

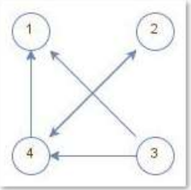

4206.jpeg

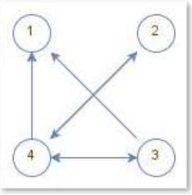

4207.jpeg

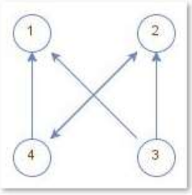

4214.jpeg

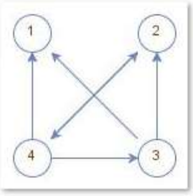

4215.jpeg

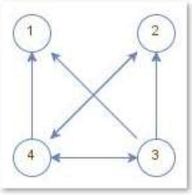

4223.jpeg

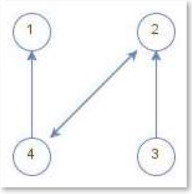

4237.jpeg

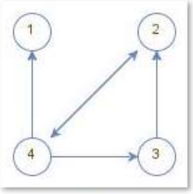

4239.jpeg

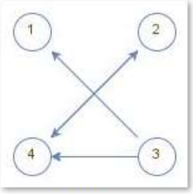

4245.jpeg

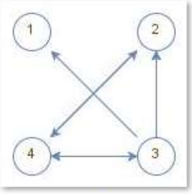

4255.jpeg

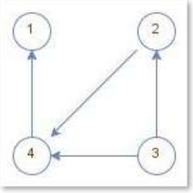

4259.jpeg

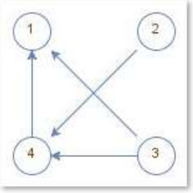

4261.jpeg

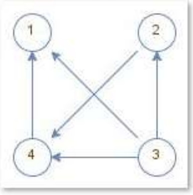

4263.jpeg

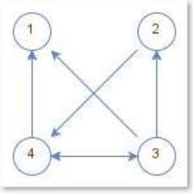

4271.jpeg

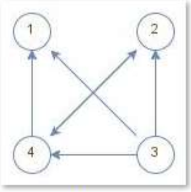

4279.jpeg

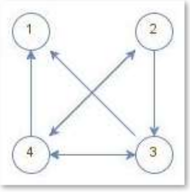

4335.jpeg

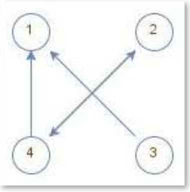

4365.jpeg

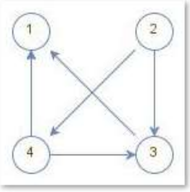

4382.jpeg

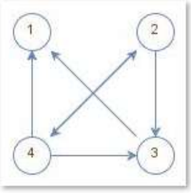

4383.jpeg

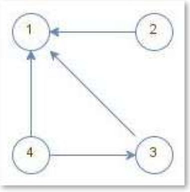

4389.jpeg

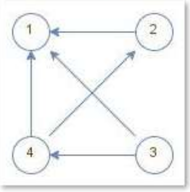

4398.jpeg

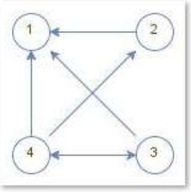

4399.jpeg

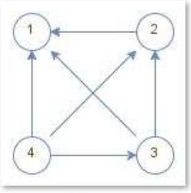

4407.jpeg

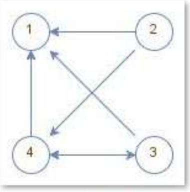

4461.jpeg

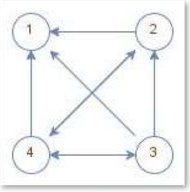

4479.jpeg

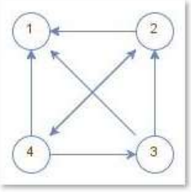

4527.jpeg

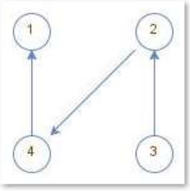

4625.jpeg

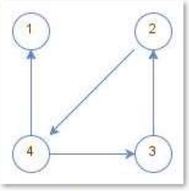

4657.jpeg

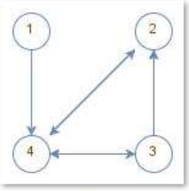

4699.jpeg

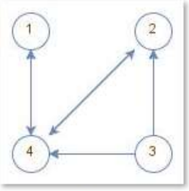

4702.jpeg

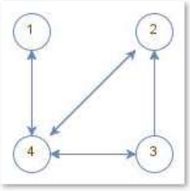

4703.jpeg

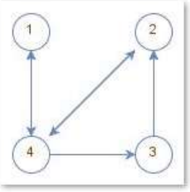

4711.jpeg

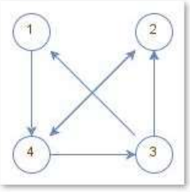

4723.jpeg

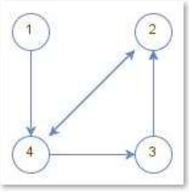

4747.jpeg

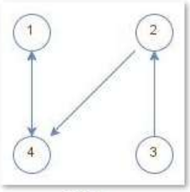

4753.jpeg

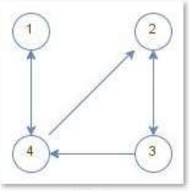

4766.jpeg

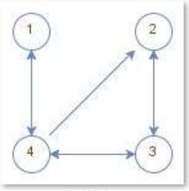

4767.jpeg

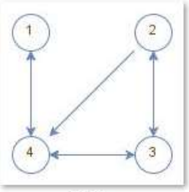

4829.jpeg

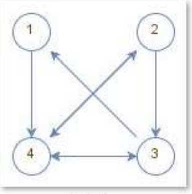

4843.jpeg

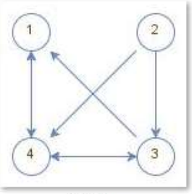

4845.jpeg

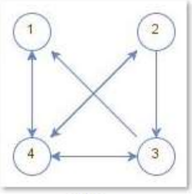

4847.jpeg

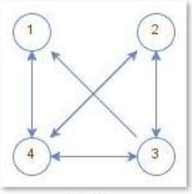

4863.jpeg

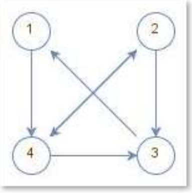

4891.jpeg

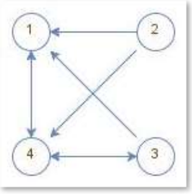

4973.jpeg

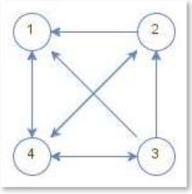

4991.jpeg

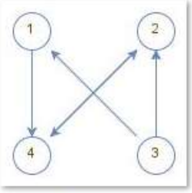

5004.jpeg

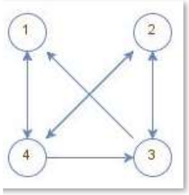

5023.jpeg

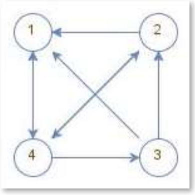

5039.jpeg

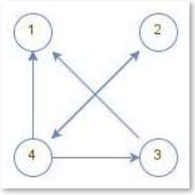

5178.jpeg

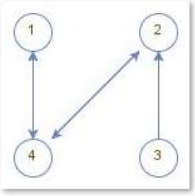

5195.jpeg

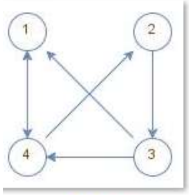

5214.jpeg

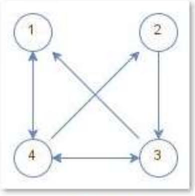

5215.jpeg

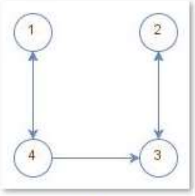

5219.jpeg

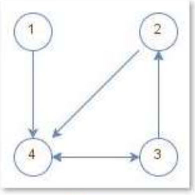

5259.jpeg

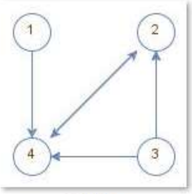

5267.jpeg

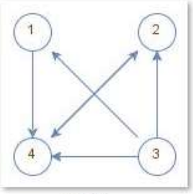

5271.jpeg

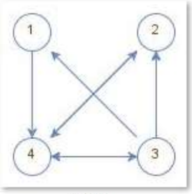

5279.jpeg

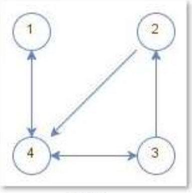

5291.jpeg

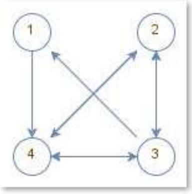

5343.jpeg

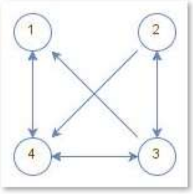

5359.jpeg

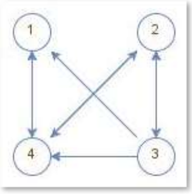

5367.jpeg

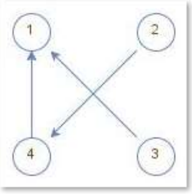

5377.jpeg

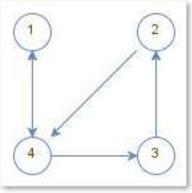

5401.jpeg

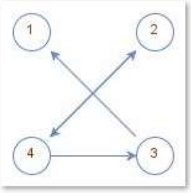

5442.jpeg

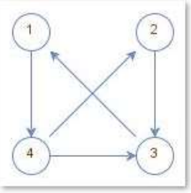

5460.jpeg

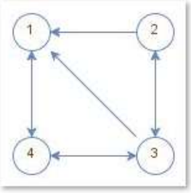

5487.jpeg

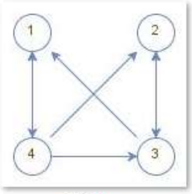

5498.jpeg

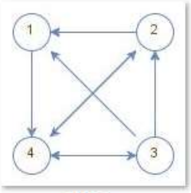

5535.jpeg

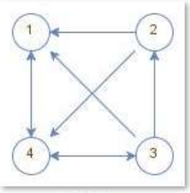

5551.jpeg

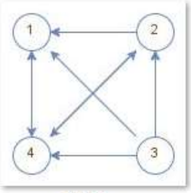

5559.jpeg

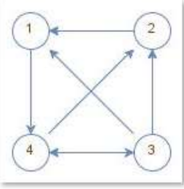

5582.jpeg

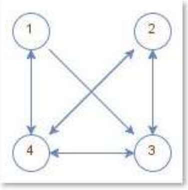

5855.jpeg

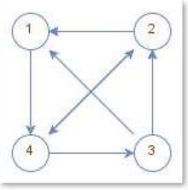

5971.jpeg

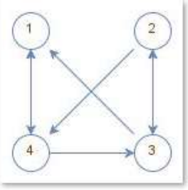

6002.jpeg

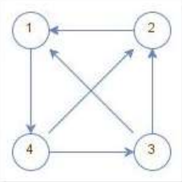

6026.jpeg

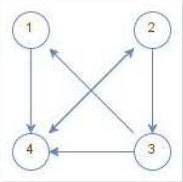

6033.jpeg

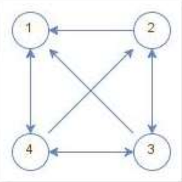

6138.jpeg

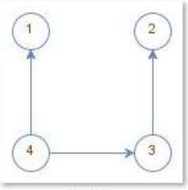

6184.jpeg

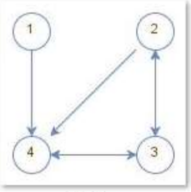

6235.jpeg

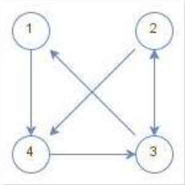

6237.jpeg

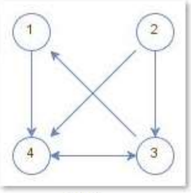

6238.jpeg

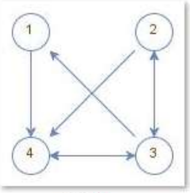

6239.jpeg

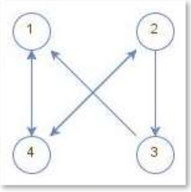

6251.jpeg

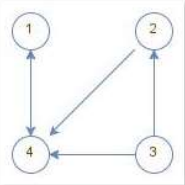

6258.jpeg

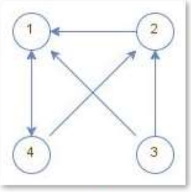

6262.jpeg

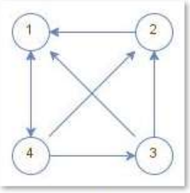

6263.jpeg

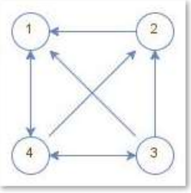

6271.jpeg

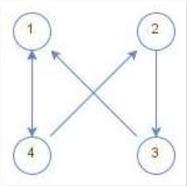

6285.jpeg

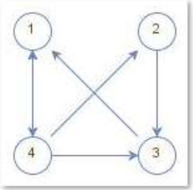

6287.jpeg

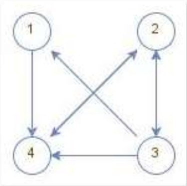

6303.jpeg

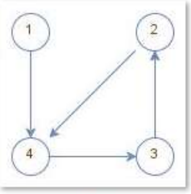

6306.jpeg

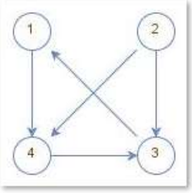

6307.jpeg

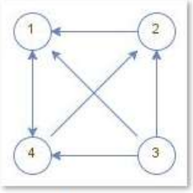

6327.jpeg

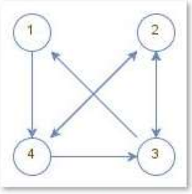

6379.jpeg

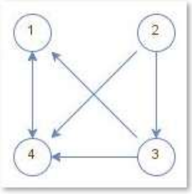

6430.jpeg

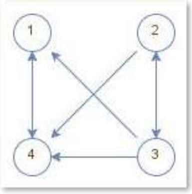

6431.jpeg

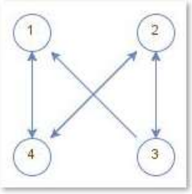

6507.jpeg

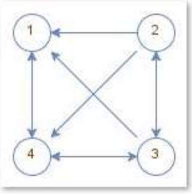

6527.jpeg

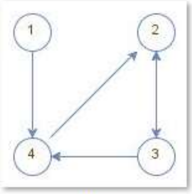

6561.jpeg

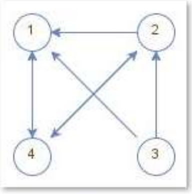

6581.jpeg

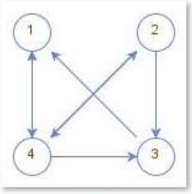

6627.jpeg

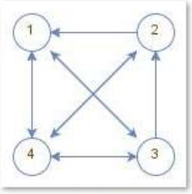

6911.jpeg

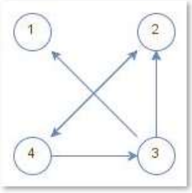

6915.jpeg

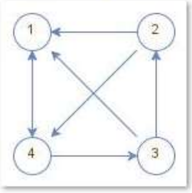

6963.jpeg

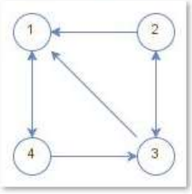

7001.jpeg

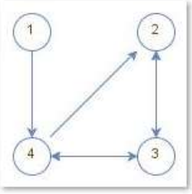

7010.jpeg

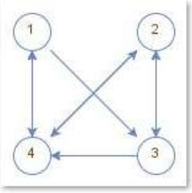

7019.jpeg

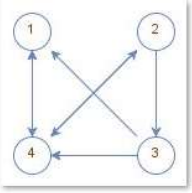

7059.jpeg

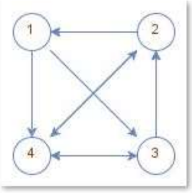

7086.jpeg

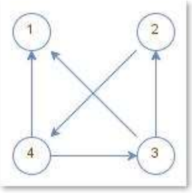

7196.jpeg

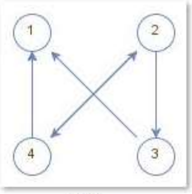

7210.jpeg

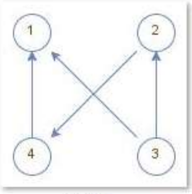

7233.jpeg

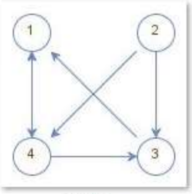

7370.jpeg

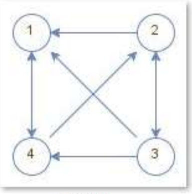

7399.jpeg

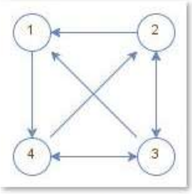

8021.jpeg

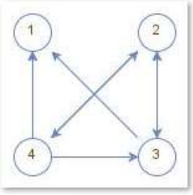

8088.jpeg

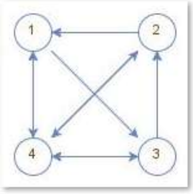

787878520.jpeg

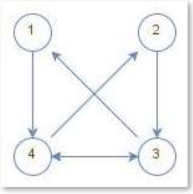

8888672672.jpeg

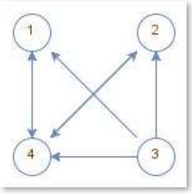

9292327461.jpeg

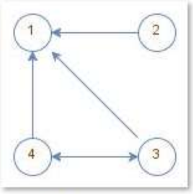

59131814814.jpeg

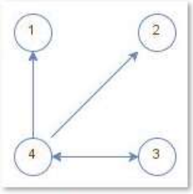

61198749749.jpeg

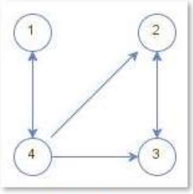

61642795795.jpeg

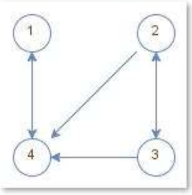

76845845849.jpeg

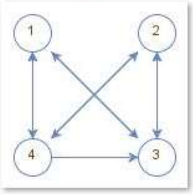

79517517929.jpeg

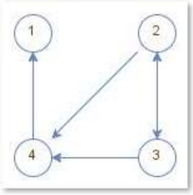

80122122312.jpeg

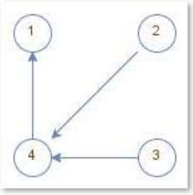

80637637639.jpeg

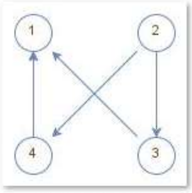

101223223480.jpeg

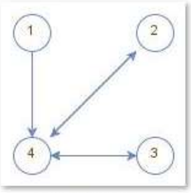

111111455624.jpeg

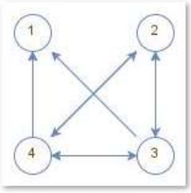

118118725866.jpeg

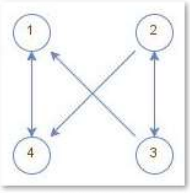

123123618618.jpeg

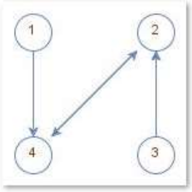

128128413413.jpeg

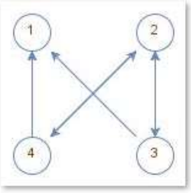

152294648648.jpeg

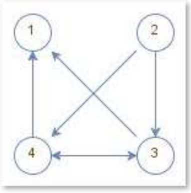

152663663673.jpeg

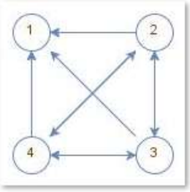

176176176546.jpeg

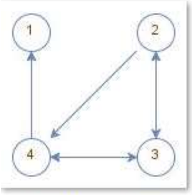

180243709709.jpeg

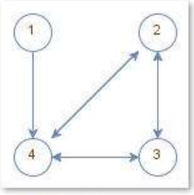

188188297455.jpeg

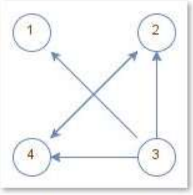

193239239877.jpeg

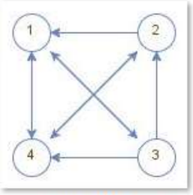

196196330697.jpeg

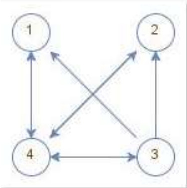

196279912912.jpeg

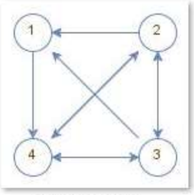

201300300414.jpeg

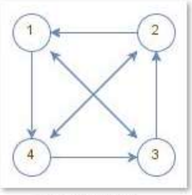

203203203203.jpeg

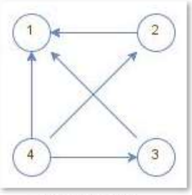

208208210840.jpeg

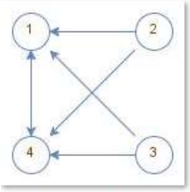

209209751751.jpeg

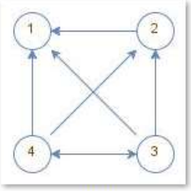

216863914914.jpeg

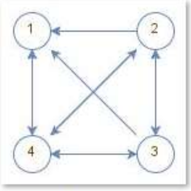

219469469929.jpeg

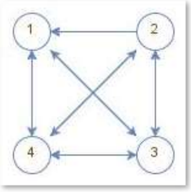

234234399651.jpeg

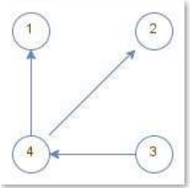

243329587587.jpeg

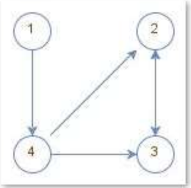

243624632632.jpeg

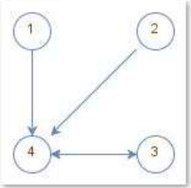

255293293849.jpeg

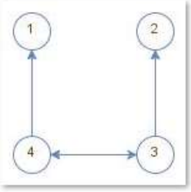

259259276276.jpeg

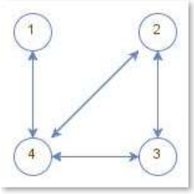

274314350350.jpeg

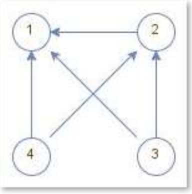

291544652652.jpeg

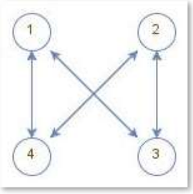

293293293293.jpeg

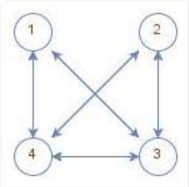

293293726726.jpeg

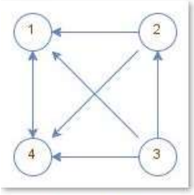

294294327377.jpeg

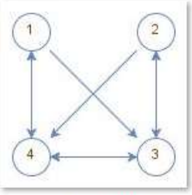

302302551551.jpeg

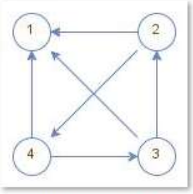

310894894894.jpeg

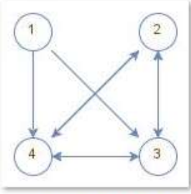

312312532911.jpeg

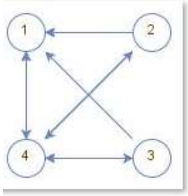

320320411547.jpeg

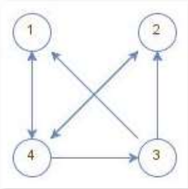

325325377411.jpeg

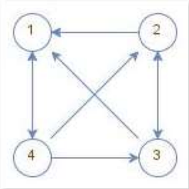

327547914914.jpeg

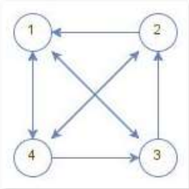

330446530530.jpeg

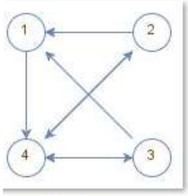

334334529592.jpeg

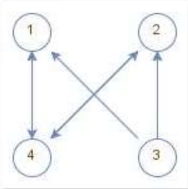

339340340860.jpeg

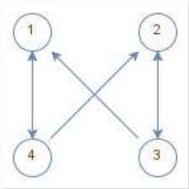

348348811811.jpeg

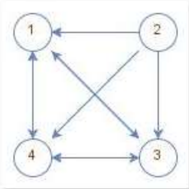

387650650650.jpeg

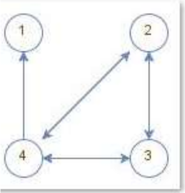

405549694694.jpeg

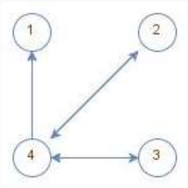

405618618877.jpeg

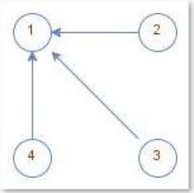

423423423573.jpeg

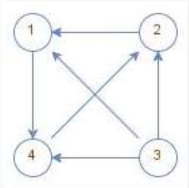

444444444746.jpeg

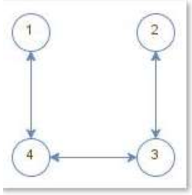

453453843843.jpeg

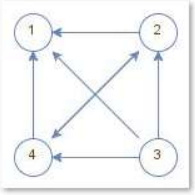

459782899899.jpeg

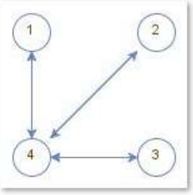

494618618618.jpeg

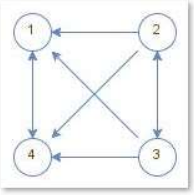

497497789789.jpeg

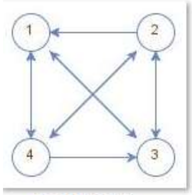

498498808808.jpeg

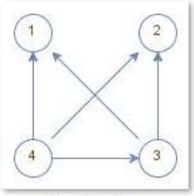

507557800800.jpeg

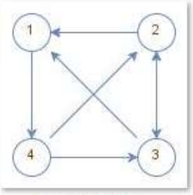

509529845845.jpeg

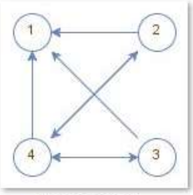

542633633782.jpeg

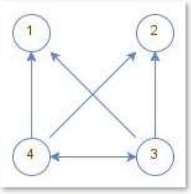

567567781781.jpeg

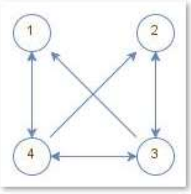

632632671671.jpeg

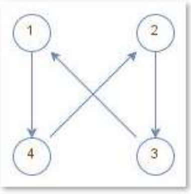

637637637637.jpeg

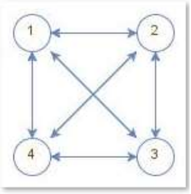

677677677677.jpeg

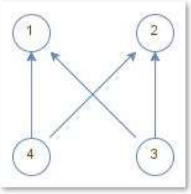

682682684684.jpeg

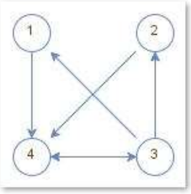

709726795795.jpeg

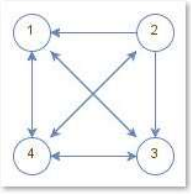

714765765798.jpeg

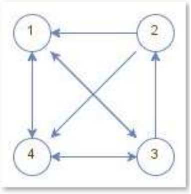

747929947947.jpeg

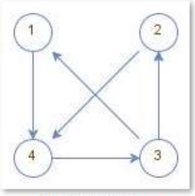

809809857890.jpeg

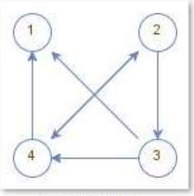

830830850886.jpeg

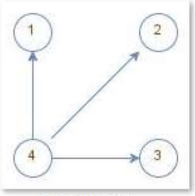

905928928928.jpeg
