# Supplementary material for: Dissecting molecular network structures using a network subgraph approach
Source: PeerJ. 2020 Aug 6;8:e9556. doi: 10.7717/peerj.9556 (PMC7512139; doi:10.7717/peerj.9556)
Supplement: Supplemental Information 5 [file peerj-08-9556-s005.pdf]

pattern id	SUM	6	12	14	36	38	46	74
6	1	1	0	0	0	0	0	0
12	1	0	1	0	0	0	0	0
36	1	0	0	0	1	0	0	0
98	2	0	1	0	0	0	0	0
14	3	1	1	1	0	0	0	0
74	3	0	1	0	1	0	0	1
38	4	1	1	0	1	1	0	0
46	6	1	1	1	1	1	1	0
78	6	1	1	1	1	0	0	1
108	6	1	1	0	1	1	0	1
102	8	1	1	1	1	1	0	1
110	12	1	1	1	1	1	1	1
238	13	1	1	1	1	1	1	1

78	98	102	108	110	238
0	0	0	0	0	0
0	0	0	0	0	0
0	0	0	0	0	0
0	1	0	0	0	0
0	0	0	0	0	0
0	0	0	0	0	0
0	0	0	0	0	0
0	0	0	0	0	0
1	0	0	0	0	0
0	0	0	1	0	0
0	1	1	0	0	0
1	1	1	1	1	0
1	1	1	1	1	1
