# Supplementary material for: Dissecting molecular network structures using a network subgraph approach
Source: PeerJ. 2020 Aug 6;8:e9556. doi: 10.7717/peerj.9556 (PMC7512139; doi:10.7717/peerj.9556)
Supplement: Supplemental Information 6 [file peerj-08-9556-s006.pdf]

pattern id	SUM	6	12	14	36	38	46	74
14	1	1	0	0	0	0	0	0
328	1	0	1	0	0	0	0	0
2184	1	0	0	0	1	0	0	0
4740	1	0	1	0	0	0	0	0
28	2	1	1	0	0	0	0	0
74	2	1	1	0	0	0	0	0
76	2	1	0	0	1	0	0	0
204	2	1	0	0	1	0	0	0
280	2	0	1	0	1	0	0	0
392	2	0	1	0	1	0	0	0
30	3	1	1	1	0	0	0	0
90	3	1	1	1	0	0	0	0
330	3	1	1	0	0	0	0	0
390	3	1	1	1	0	0	0	0
456	3	1	1	0	1	0	0	0
904	3	1	1	0	1	0	0	0
4370	3	0	1	0	1	0	0	1
4418	3	0	1	0	1	0	0	0
4420	3	0	1	0	1	0	0	1
4424	3	0	1	0	1	0	0	1
78	4	1	1	0	1	1	0	0
92	4	1	1	0	1	1	0	0
206	4	1	1	0	1	1	0	0
344	4	1	1	0	1	1	0	0
394	4	1	1	0	1	1	0	0
396	4	1	1	1	1	0	0	0
404	4	1	1	0	1	0	0	1
408	4	1	1	0	1	1	0	0
472	4	1	1	0	1	1	0	0
906	4	1	1	0	1	1	0	0
908	4	1	1	1	1	0	0	0
2186	4	1	1	0	1	1	0	0
2190	4	1	1	0	1	1	0	0
2204	4	1	1	0	1	1	0	0
2252	4	1	1	0	1	1	0	0
2254	4	1	1	0	1	1	0	0
4546	4	1	1	0	1	0	0	0
4548	4	1	1	0	1	0	0	1
282	5	1	1	1	1	0	0	1
332	5	1	1	1	1	0	0	1
398	5	1	1	1	1	1	0	0
458	5	1	1	0	1	1	0	0
460	5	1	1	1	1	0	0	1
2506	5	1	1	0	1	1	0	0

4440	5	1	1	0	1	1	0	1
4682	5	1	1	1	1	0	0	1
4742	5	1	1	0	1	1	0	0
4748	5	1	1	1	1	0	0	1
4812	5	1	1	1	1	0	0	1
4994	5	1	1	0	1	1	0	0
5064	5	1	1	1	1	0	0	1
6342	5	1	1	0	1	1	0	0
6356	5	1	1	0	1	1	0	0
94	6	1	1	1	1	1	1	0
222	6	1	1	1	1	1	1	0
286	6	1	1	1	1	0	0	1
406	6	1	1	1	1	0	0	1
412	6	1	1	1	1	1	0	1
468	6	1	1	0	1	1	0	1
856	6	1	1	1	1	1	1	0
910	6	1	1	1	1	1	1	0
972	6	1	1	1	1	0	0	1
2202	6	1	1	1	1	1	1	0
2206	6	1	1	1	1	1	1	0
2270	6	1	1	1	1	1	1	0
4374	6	1	1	1	1	0	0	1
4382	6	1	1	1	1	0	0	1
4426	6	1	1	1	1	0	0	1
4428	6	1	1	1	1	0	0	1
4436	6	1	1	0	1	1	0	1
4556	6	1	1	1	1	0	0	1
4564	6	1	1	0	1	1	0	1
4678	6	1	1	0	1	1	0	1
4698	6	1	1	1	1	0	0	1
5004	6	1	1	1	1	0	0	1
5016	6	1	1	0	1	1	0	1
5068	6	1	1	1	1	0	0	1
6552	6	1	1	0	1	1	0	1
6616	6	1	1	0	1	1	0	1
13260	6	1	1	1	1	0	0	1
348	7	1	1	1	1	1	0	1
410	7	1	1	1	1	1	1	1
476	7	1	1	1	1	1	0	1
2458	7	1	1	1	1	1	1	1
4686	7	1	1	1	1	1	0	1
4952	7	1	1	1	1	1	1	1
334	8	1	1	1	1	1	0	1
346	8	1	1	1	1	1	0	1
414	8	1	1	1	1	1	1	1

454	8	1	1	1	1	1	0	1
462	8	1	1	1	1	1	0	1
922	8	1	1	1	1	1	1	1
924	8	1	1	1	1	1	0	1
2462	8	1	1	1	1	1	1	1
2524	8	1	1	1	1	1	1	1
4422	8	1	1	1	1	1	0	1
4434	8	1	1	1	1	1	0	1
4444	8	1	1	1	1	1	0	1
4550	8	1	1	1	1	1	0	1
4562	8	1	1	1	1	1	0	1
4572	8	1	1	1	1	1	0	1
4692	8	1	1	1	1	1	0	1
4700	8	1	1	1	1	1	0	1
4750	8	1	1	1	1	1	0	1
4758	8	1	1	1	1	1	0	1
4764	8	1	1	1	1	1	0	1
4998	8	1	1	1	1	1	1	1
5002	8	1	1	1	1	1	0	1
5012	8	1	1	1	1	1	0	1
5058	8	1	1	1	1	1	0	1
5066	8	1	1	1	1	1	0	1
5080	8	1	1	1	1	1	1	1
6348	8	1	1	1	1	1	0	1
6350	8	1	1	1	1	1	0	1
6598	8	1	1	1	1	1	0	1
7128	8	1	1	1	1	1	1	1
14790	8	1	1	1	1	1	0	1
474	9	1	1	1	1	1	1	1
2510	9	1	1	1	1	1	1	1
4430	9	1	1	1	1	1	0	1
4442	9	1	1	1	1	1	0	1
4558	9	1	1	1	1	1	0	1
4814	9	1	1	1	1	1	0	1
5010	9	1	1	1	1	1	0	1
6358	9	1	1	1	1	1	1	1
6364	9	1	1	1	1	1	0	1
6550	9	1	1	1	1	1	0	1
6602	9	1	1	1	1	1	0	1
6854	9	1	1	1	1	1	1	1
6858	9	1	1	1	1	1	1	1
5006	10	1	1	1	1	1	1	1
5020	10	1	1	1	1	1	0	1
6862	10	1	1	1	1	1	1	1
6876	10	1	1	1	1	1	1	1

350	12	1	1	1	1	1	1	1
470	12	1	1	1	1	1	1	1
478	12	1	1	1	1	1	1	1
858	12	1	1	1	1	1	1	1
926	12	1	1	1	1	1	1	1
974	12	1	1	1	1	1	1	1
2526	12	1	1	1	1	1	1	1
4438	12	1	1	1	1	1	1	1
4446	12	1	1	1	1	1	1	1
4566	12	1	1	1	1	1	1	1
4574	12	1	1	1	1	1	1	1
4694	12	1	1	1	1	1	1	1
4702	12	1	1	1	1	1	1	1
4766	12	1	1	1	1	1	1	1
4830	12	1	1	1	1	1	1	1
4946	12	1	1	1	1	1	1	1
4954	12	1	1	1	1	1	1	1
5014	12	1	1	1	1	1	1	1
5018	12	1	1	1	1	1	1	1
5022	12	1	1	1	1	1	1	1
5062	12	1	1	1	1	1	1	1
5070	12	1	1	1	1	1	1	1
5074	12	1	1	1	1	1	1	1
5076	12	1	1	1	1	1	1	1
5082	12	1	1	1	1	1	1	1
5084	12	1	1	1	1	1	1	1
6366	12	1	1	1	1	1	1	1
6554	12	1	1	1	1	1	1	1
6558	12	1	1	1	1	1	1	1
6604	12	1	1	1	1	1	1	1
6606	12	1	1	1	1	1	1	1
6614	12	1	1	1	1	1	1	1
6618	12	1	1	1	1	1	1	1
6620	12	1	1	1	1	1	1	1
6622	12	1	1	1	1	1	1	1
6870	12	1	1	1	1	1	1	1
6874	12	1	1	1	1	1	1	1
6878	12	1	1	1	1	1	1	1
7130	12	1	1	1	1	1	1	1
13146	12	1	1	1	1	1	1	1
13148	12	1	1	1	1	1	1	1
13262	12	1	1	1	1	1	1	1
14678	12	1	1	1	1	1	1	1
14686	12	1	1	1	1	1	1	1
14798	12	1	1	1	1	1	1	1

14812	12	1	1	1	1	1	1	1
15310	12	1	1	1	1	1	1	1
862	13	1	1	1	1	1	1	1
990	13	1	1	1	1	1	1	1
3038	13	1	1	1	1	1	1	1
4950	13	1	1	1	1	1	1	1
4958	13	1	1	1	1	1	1	1
5078	13	1	1	1	1	1	1	1
5086	13	1	1	1	1	1	1	1
7126	13	1	1	1	1	1	1	1
7134	13	1	1	1	1	1	1	1
13142	13	1	1	1	1	1	1	1
13150	13	1	1	1	1	1	1	1
13278	13	1	1	1	1	1	1	1
14810	13	1	1	1	1	1	1	1
14814	13	1	1	1	1	1	1	1
15258	13	1	1	1	1	1	1	1
15262	13	1	1	1	1	1	1	1
15326	13	1	1	1	1	1	1	1
31710	13	1	1	1	1	1	1	1

78	98	102	108	110	238
0	0	0	0	0	0
0	0	0	0	0	0
0	0	0	0	0	0
0	0	0	0	0	0
0	0	0	0	0	0
0	0	0	0	0	0
0	0	0	0	0	0
0	0	0	0	0	0
0	0	0	0	0	0
0	0	0	0	0	0
0	0	0	0	0	0
0	0	0	0	0	0
0	1	0	0	0	0
0	0	0	0	0	0
0	0	0	0	0	0
0	0	0	0	0	0
0	0	0	0	0	0
0	1	0	0	0	0
0	0	0	0	0	0
0	0	0	0	0	0
0	0	0	0	0	0
0	0	0	0	0	0
0	0	0	0	0	0
0	0	0	0	0	0
0	0	0	0	0	0
0	0	0	0	0	0
0	0	0	0	0	0
0	0	0	0	0	0
0	0	0	0	0	0
0	0	0	0	0	0
0	0	0	0	0	0
0	0	0	0	0	0
0	0	0	0	0	0
0	0	0	0	0	0
0	0	0	0	0	0
0	0	0	0	0	0
0	1	0	0	0	0
0	0	0	0	0	0
0	0	0	0	0	0
0	0	0	0	0	0
0	0	0	0	0	0
0	1	0	0	0	0
0	0	0	0	0	0
0	1	0	0	0	0

0	0	0	0	0	0
0	0	0	0	0	0
0	1	0	0	0	0
0	0	0	0	0	0
0	0	0	0	0	0
0	1	0	0	0	0
0	0	0	0	0	0
0	1	0	0	0	0
0	1	0	0	0	0
0	0	0	0	0	0
0	0	0	0	0	0
1	0	0	0	0	0
1	0	0	0	0	0
0	0	0	0	0	0
0	0	0	1	0	0
0	0	0	0	0	0
0	0	0	0	0	0
1	0	0	0	0	0
0	0	0	0	0	0
0	0	0	0	0	0
0	0	0	0	0	0
1	0	0	0	0	0
1	0	0	0	0	0
0	1	0	0	0	0
1	0	0	0	0	0
0	0	0	1	0	0
1	0	0	0	0	0
0	0	0	1	0	0
0	0	0	1	0	0
1	0	0	0	0	0
1	0	0	0	0	0
0	0	0	1	0	0
1	0	0	0	0	0
0	0	0	1	0	0
0	0	0	1	0	0
1	0	0	0	0	0
0	0	0	1	0	0
0	0	0	0	0	0
0	0	0	1	0	0
0	0	0	0	0	0
0	0	0	1	0	0
0	0	0	0	0	0
0	1	1	0	0	0
0	1	1	0	0	0
1	0	0	0	0	0

0	1	1	0	0	0
0	1	1	0	0	0
0	0	0	1	0	0
0	1	1	0	0	0
1	0	0	0	0	0
0	0	0	1	0	0
0	1	1	0	0	0
0	1	1	0	0	0
1	0	0	1	0	0
0	1	1	0	0	0
0	1	1	0	0	0
1	0	0	1	0	0
0	1	1	0	0	0
0	1	1	0	0	0
0	1	1	0	0	0
0	1	1	0	0	0
0	1	0	1	0	0
0	1	0	0	0	0
0	1	1	0	0	0
0	1	1	0	0	0
0	1	1	0	0	0
0	1	1	0	0	0
0	0	0	1	0	0
0	1	1	0	0	0
0	1	1	0	0	0
0	1	1	0	0	0
0	0	0	1	0	0
0	1	1	0	0	0
0	1	1	0	0	0
0	1	1	0	0	0
1	1	1	0	0	0
1	1	1	0	0	0
1	1	1	0	0	0
0	1	1	1	0	0
0	1	1	1	0	0
0	1	1	0	0	0
0	1	1	1	0	0
1	1	1	0	0	0
0	1	1	1	0	0
0	1	0	1	0	0
0	1	1	0	0	0
1	1	1	0	0	0
1	1	1	1	0	0
0	1	1	1	0	0
0	1	1	1	0	0

1	1	1	1	1	0
1	1	1	1	1	0
1	1	1	1	1	0
1	1	1	1	1	0
1	1	1	1	1	0
1	1	1	1	1	0
1	1	1	1	1	0
1	1	1	1	1	0
1	1	1	1	1	0
1	1	1	1	1	0
1	1	1	1	1	0
1	1	1	1	1	0
1	1	1	1	1	0
1	1	1	1	1	0
1	1	1	1	1	0
1	1	1	1	1	0
1	1	1	1	1	0
1	1	1	1	1	0
1	1	1	1	1	0
1	1	1	1	1	0
1	1	1	1	1	0
1	1	1	1	1	0
1	1	1	1	1	0
1	1	1	1	1	0
1	1	1	1	1	0
1	1	1	1	1	0
1	1	1	1	1	0
1	1	1	1	1	0
1	1	1	1	1	0
1	1	1	1	1	0
1	1	1	1	1	0
1	1	1	1	1	0
1	1	1	1	1	0
1	1	1	1	1	0
1	1	1	1	1	0
1	1	1	1	1	0
1	1	1	1	1	0
1	1	1	1	1	0
1	1	1	1	1	0
1	1	1	1	1	0
1	1	1	1	1	0
1	1	1	1	1	0
1	1	1	1	1	0
1	1	1	1	1	0
1	1	1	1	1	0

1	1	1	1	1	0
1	1	1	1	1	0
1	1	1	1	1	1
1	1	1	1	1	1
1	1	1	1	1	1
1	1	1	1	1	1
1	1	1	1	1	1
1	1	1	1	1	1
1	1	1	1	1	1
1	1	1	1	1	1
1	1	1	1	1	1
1	1	1	1	1	1
1	1	1	1	1	1
1	1	1	1	1	1
1	1	1	1	1	1
1	1	1	1	1	1
1	1	1	1	1	1
1	1	1	1	1	1
1	1	1	1	1	1
1	1	1	1	1	1
