# Supplementary material for: Dissecting molecular network structures using a network subgraph approach
Source: PeerJ. 2020 Aug 6;8:e9556. doi: 10.7717/peerj.9556 (PMC7512139; doi:10.7717/peerj.9556)
Supplement: Supplemental Information 7 [file peerj-08-9556-s007.pdf]

pattern id	6	12	14	36	38	46	74
E	0	0	-1	0	0	-1	-1
	0	0	0	0	0	0	0
	0	0	1	0	0	1	1
LE							
	1	1	1	1	2	2	1
	1	1	1.38	1	2	2	1.38
	2	2	3.62	2	2	4	3.62
QE							
	1	1	1	1	2	2	1
	1	1	1.38	1	2	2	1.38
	2	2	3.62	2	2	4	3.62
AAt							
	0	0	0	0	0	0	0
	0	1	1	0	0.382	1	1
	2	1	2	2	2.62	3	2
LLt							
	0.628	0.475	0.769	0.628	1.39	2	0.769
	1	1.37	2.34	1	6.34	8	2.34
	6.37	6.16	13.9	6.37	7.27	16	13.9
energy	4.32	4.34	6.13	4.32	6.39	8.24	6.13
QQt							
	0.628	0.475	0.769	0.628	2.32	3.45	0.769
	1	1.37	2.34	1	2.79	4	2.34
	6.37	6.16	13.9	6.37	9.89	18.5	13.9
energy	4.32	4.34	6.13	4.32	6.34	8.16	6.13
LQt							
	-2	-1	-4	-2	-1.5+0.866	-4	-4
	0	-1	0	0	-1.5-0.866i	0	0
	0	0	1	0	0	0	1
Aqt							
	0	0	-1	0	-0.303	-2	-1
	0	1	0	0	0	0	0
	2	1	4	2	3.3	6	4
ALt							
	1	1	1	1	4	4	1
	1	1	3.21	1	4	8	3.21
	4	4	7.79	4	4	8	7.79

78	98	102	108	110	238
-1.41	-0.5+0.866i	-0.662+0.562i	-1	-1	-1
0	-0.5-0.866i	-0.662-0.562i	0	-0.618	-1
1.41	1	1.32	1	1.62	2
1.27	1	1.38	2	1.75	2
2	2.5+0.866i	3	2	3.45	5
4.73	2.5-0.866i	3.62	4	4.8	5
1.27	1.5+0.866i	1.9+0.665i	2	[illegible].43+0.369i	3
2	1.5-0.866i	1.9-0.665i	2	[illegible].43-0.369i	3
4.73	3	4.21	4	5.15	6
0	1	0.382	0.00E+00	0.198	1
2	1	1	1	1.55	1
2	1	2.62	3	3.25	4
1.61	1	1.79	2	2.84	4
4	7	7.54	8	12.6	25
22.4	7	16.7	16	23.6	25
8	6.29	8.17	8.24	10.1	12
1.61	3	3.58	3.45	5.49	9
4	3	4.51	4	6.47	9
22.4	9	17.9	18.5	27	36
8	6.46	8.25	8.16	10.1	12
-6	-2+1.73i	-3.72	-4	-5.5	-5
0	-2-1.73i	-2.15	0	-2.06	-5
2	1	1.87	0	2.56	4
-2	0+1.73i	-0.868+1.49i	-2	[illegible].83+0.501i	-3
0	0-1.73i	-0.868-1.49i	0	[illegible].83-0.501i	-3
6	3	5.74	6	8.65	12
2.71	3	3.94	4	7.21	12
4	4.5+0.866i	8.03+0.495i	8	9.12	15
13.3	4.5-0.866i	8.03-0.495i	8	13.7	15

pattern id	6	12	14
E	[0;0;1]	[0;1;0]	[0;0.707;-0.707]
	[0;0;-1]	[0;-1;0]	[0.707;-0.707;0]
	[0;0;-1]	[0;1;0]	[0;0.707;0.707]
LE			
	[0.707;0;0.707]	[0;1;0]	[0.577;0.577;0.577]
	[0;0.707;0.707]	[0;1;0]	[0;0.851;0.526]
	[0;0;1]	[0;-0.707;0.707]	[0;-0.526;0.851]
QE			
	[0.707;0;-0.707]	[0;1;0]	[0.577;0.577;-0.577]
	[0;0.707;-0.707]	[0;1;0]	[0;0.851;-0.526]
	[0;0;1]	[0;0.707;0.707]	[0;0.526;0.851]
AAt			
	[1;0;0]	[1;0;0]	[1;0;0]
	[0;1;0]	[0;1;0]	[0;1;0]
	[0;0;1]	[0;0;1]	[0;0;1]
LLt			
	[0.684;0.684;0.255]	[-0.756;-0.521;-0.397]	[0.941;0.257;0.218]
	[0.707;-0.707;0]	[-0.632;0.739;0.233]	[-0.33;0.833;0.443]
	[-0.18;-0.18;0.967]	[-0.172;-0.427;0.888]	[-0.0675;-0.489;0.87]
QQt			
	[0.684;0.684;-0.255]	[0.756;0.521;-0.397]	[0.941;0.257;-0.218]
	[0.707;-0.707;0]	[0.632;-0.739;0.233]	[-0.33;0.833;-0.443]
	[0.18;0.18;0.967]	[0.172;0.427;0.888]	[0.0675;0.489;0.87]
LQt			
	[0;0;1]	[0;1;0]	[0;0.707;-0.707]
	[0.894;0;0.447]	[0;-1;0]	[0.784;-0.588;-0.196]
	[0;0.894;0.447]	[0.408;0.816;0.408]	[0;-0.832;-0.555]
AQt			
	[0.894;0;-0.447]	[0.408;0.816;-0.408]	[0;0.832;-0.555]
	[0;0.894;-0.447]	[0;1;0]	[0.784;-0.588;0.196]
	[0;0;1]	[0;-1;0]	[0;0.707;0.707]
ALt			
	[0.949;0;-0.316]	[0;1;0]	[0.905;0.302;-0.302]
	[0;0.949;-0.316]	[0;1;0]	[0;0.979;-0.204]
	[0;0;1]	[0;-0.316;0.949]	[0;-0.204;0.979]

36	38	46	74
[0;0;1]	[0;0;1]	[0;0.707;-0	[-0.577;-0.577;0.577]
[0;1;0]	[0;0;-1]	[0.577;-0.5	[1;0;0]
[0;-0.707;-0.707]	[0;0;1]	[0;0.707;0.	[0.577;0.577;0.577]
[0;0;1]	[0;0;1]	[0;0.707;0.	[1;0;0]
[0;1;0]	[0;0;1]	[0;0.707;0.	[0.809;-0.5;-0.309]
[0.577;-0.577;-0.577]	[0;0;1]	[0;-0.707;0	[0.309;0.5;-0.809]
[0;0;1]	[0;0;1]	[0;0.707;-0	[1;0;0]
[0;1;0]	[0;0;-1]	[0.816;-0.4	[0.809;-0.5;0.309]
[0.577;0.577;0.577]	[0;0;1]	[0;0.707;0.	[-0.309;-0.5;-0.809]
[1;0;0]	[1;0;0]	[1;0;0]	[-0.707;0.707;0]
[0;-0.707;0.707]	[0;-0.851;0.526]	[0;-0.707;0	[0;0;1]
[0;0.707;0.707]	[0;0.526;0.851]	[0;0.707;0.	[0.707;0.707;0]
[-0.643;-0.542;-0.542]	[-0.733;-0.525;-0.432]	[0.816;0.4	[-0.825;-0.339;-0.452]
[0;-0.707;0.707]	[0.343;-0.834;0.432]	[-0.577;0.5	[-0.505;0.8;0.324]
[-0.766;0.454;0.454]	[-0.587;0.169;0.792]	[0;-0.707;0	[-0.251;-0.496;0.831]
[-0.643;0.542;0.542]	[0.491;-0.786;0.374]	[0.982;-0.1	[0.825;0.339;-0.452]
[0;-0.707;0.707]	[0.756;0.172;-0.631]	[0;0.707;-0	[0.505;-0.8;0.324]
[0.766;0.454;0.454]	[0.432;0.593;0.679]	[0.191;0.6	[0.251;0.496;0.831]
[0;0.707;0.707]	[0;0.354+0.612i;0.707]	[0;0.707;-0	[0.64;0.64;-0.426]
[0;-0.707;0.707]	[0;0.354-0.612i;0.707]	[0;0.707;0.	[-0.913;-0.183;-0.365]
[0.577;0.577;0.577]	[0.557;0.371;0.743]	[0;-0.707;-	[0.577;0.577;0.577]
[0;0.707;-0.707]	[0;0.609;-0.793]	[0;0.707;-0	[-0.577;-0.577;0.577]
[0.577;-0.577;-0.577]	[0.218;0.436;-0.873]	[0.905;-0.3	[0.913;0.183;-0.365]
[0;0.707;0.707]	[0;0.398;0.917]	[0;0.707;0.	[0.64;0.64;0.426]
[0;0;1]	[0;0;1]	[1;0;0]	[1;0;0]
[0;1;0]	[0;0;1]	[0;1;0]	[0.25;-0.948;0.198]
[0.905;-0.302;-0.302]	[0;1;0]	[0;0;1]	[0.25;0.198;-0.948]

78	98	102
[0.5;0.5;-0.707]	[0.577;-0.289-0.5i;-0.289+0.5i]	[0.434-0.369i;-0.656;-0.0804+0.489i]
[0.707;-0.707;0]	[0.577;-0.289+0.5i;-0.289-0.5i]	[0.434+0.369i;-0.656;-0.0804-0.489i]
[0.5;0.5;0.707]	[-0.577;-0.577;-0.577]	[-0.548;-0.414;-0.727]
[0.628;0.628;0.46]	[-0.577;-0.577;-0.577]	[-0.4;-0.648;-0.648]
[0.707;-0.707;0]	[-0.289+0.5i;-0.289-0.5i;0.577]	[0.707;-0.707;0]
[-0.325;-0.325;0.888]	[-0.289-0.5i;-0.289+0.5i;0.577]	[0.753;-0.465;-0.465]
[0.628;0.628;-0.46]	[-0.289-0.5i;-0.289+0.5i;0.577]	[-0.0692+0.448i;0.673;-0.222-0.54i]
[0.707;-0.707;0]	[-0.289+0.5i;-0.289-0.5i;0.577]	[-0.0692-0.448i;0.673;-0.222+0.54i]
[0.325;0.325;0.888]	[-0.577;-0.577;-0.577]	[0.613;0.278;0.739]
[-0.707;0.707;0]	[1;0;0]	[0;-0.851;0.526]
[0.707;0.707;0]	[0;1;0]	[1;0;0]
[0;0;1]	[0;0;1]	[0;0.526;0.851]
[0.628;0.628;0.46]	[-0.577;-0.577;-0.577]	[-0.578;-0.681;-0.45]
[0.707;-0.707;0]	[0.187;-0.782;0.595]	[0.432;-0.723;0.539]
[-0.325;-0.325;0.888]	[0.795;-0.235;-0.559]	[-0.693;0.117;0.712]
[0.628;0.628;-0.46]	[0.04;-0.726;0.686]	[0.342;-0.934;0.101]
[0.707;-0.707;0]	[0.816;-0.373;-0.442]	[0.682;0.172;-0.71]
[0.325;0.325;0.888]	[0.577;0.577;0.577]	[0.646;0.312;0.696]
[0.577;0.577;-0.577]	[0.577;-0.289-0.5i;-0.289+0.5i]	[0.507;-0.728;-0.461]
[0.707;-0.707;0]	[0.577;-0.289+0.5i;-0.289-0.5i]	[0.319;-0.94;-0.122]
[0.577;0.577;0.577]	[-0.577;-0.577;-0.577]	[0.642;0.457;0.615]
[-0.577;-0.577;0.577]	[-0.289-0.5i;-0.289+0.5i;0.577]	[0.391-0.471i;-0.623;-0.0104+0.487i]
[-0.707;0.707;0]	[-0.289+0.5i;-0.289-0.5i;0.577]	[0.391+0.471i;-0.623;-0.0104-0.487i]
[-0.577;-0.577;-0.577]	[-0.577;-0.577;-0.577]	[0.475;0.459;0.75]
[-0.686;-0.686;0.243]	[-0.577;-0.577;-0.577]	[-0.235;-0.956;0.177]
[-0.707;0.707;0]	[-0.289-0.5i;-0.289+0.5i;0.577]	[-0.19-0.341i;-0.163+0.105i;0.9]
[-0.172;-0.172;0.97]	[-0.289+0.5i;-0.289-0.5i;0.577]	[-0.19+0.341i;-0.163-0.105i;0.9]

108	110	238
[-0.707;0;0.707]	[-0.707;0;0.707]	[-0.715;0.0166;0.699]
[0;1;0]	[-0.753;0.465;0.465]	[0.394;-0.816;0.423]
[0.408;0.816;0.408]	[0.4;0.648;0.648]	[0.577;0.577;0.577]
[0;1;0]	[0.415;0.748;0.518]	[0.577;0.577;0.577]
[0;1;0]	[0.603;-0.752;-0.268]	[0.154;-0.772;0.617]
[0.707;0;-0.707]	[-0.474;-0.211;0.855]	[-0.802;0.267;0.535]
[0;1;0]	[0.682;-0.159-0.541i;-0.392+0.25	[-0.816;0.39;0.426]
[-0.707;0;0.707]	[0.682;-0.159+0.541i;-0.392-0.25	[0.0211;-0.717;0.696]
[0.577;0.577;0.577]	[0.359;0.526;0.771]	[0.577;0.577;0.577]
[0.577;-0.577;0.577]	[0.737;-0.591;0.328]	[0.408;0.408;-0.816]
[-0.707;0;0.707]	[-0.591;-0.328;0.737]	[0.707;-0.707;0]
[0.408;0.816;0.408]	[0.328;0.737;0.591]	[0.577;0.577;0.577]
[0.577;0.577;0.577]	[0.659;0.543;0.519]	[0.577;0.577;0.577]
[0.408;-0.816;0.408]	[0.643;-0.765;-0.0162]	[-0.408;-0.408;0.816]
[-0.707;0;0.707]	[-0.389;-0.345;0.854]	[0.707;-0.707;0]
[0.291;-0.912;0.291]	[0.481;0.591;-0.647]	[-0.401;0.816;-0.415]
[0.707;0;-0.707]	[0.766;-0.643;-0.0188]	[0.711;-0.00834;-0.703]
[0.645;0.411;0.645]	[0.427;0.487;0.762]	[0.577;0.577;0.577]
[-0.707;0;0.707]	[-0.675;-0.218;0.704]	[-0.756;0.11;0.646]
[0.408+6.04e-09i;0.816;0.408+6.0	[-0.527;0.781;0.335]	[-0.309;0.809;-0.5]
[0.408-6.04e-09i;0.816;0.408-6.0	[-0.504;-0.618;-0.603]	[0.577;0.577;0.577]
[-0.707;0;0.707]	[0.796;-0.103-0.214i;-0.536+0.15	[-0.458;0.814;-0.356]
[0.236;-0.943;0.236]	[0.796;-0.103+0.214i;-0.536-0.15	[0.676;0.0588;-0.735]
[-0.408;-0.816;-0.408]	[0.403;0.692;0.598]	[0.577;0.577;0.577]
[0;1;0]	[-0.731;-0.675;0.0995]	[0.577;0.577;0.577]
[0.894;-0.447;0]	[0.571;-0.804;0.165]	[-0.408;-0.408;0.816]
[0;-0.447;0.894]	[-0.111;-0.313;0.943]	[0.707;-0.707;0]

pattern id	14	28	30	74	76	78	90
E	0	0	-1	0	0	0	-1
	0	0	0	0	0	0	0
	0	0	0	0	0	0	0
	0	0	1	0	0	0	1
LE	1	1	1	1	1	1	1
	1	1	1	1	1	2	1
	1	1	1.59	2	2	2	2
	3	3	4.41	2	2	3	4
QE	1	1	1	1	1	1	1
	1	1	1	1	1	2	1
	1	1	1.59	2	2	2	2
	3	3	4.41	2	2	3	4
AAt	0	0	0	0	0	0	0
	0	0	0	0	0	0	0
	0	1	1	1	0.382	0.586	2
	3	2	3	2	2.62	3.41	2
LLt	0.734	0.536	0.793	0.642	0.71	0.875	0.764
	1	1	1	0.851	0.804	2	0.938
	1	1.39	2.9	3.8	3.52	6.53	5.24
	12.3	12.1	21.3	7.71	7.97	12.6	17.1
QQt	0.734	0.536	0.793	0.642	0.71	0.886	0.764
	1	1	1	0.851	0.804	2.43	0.938
	1	1.39	2.9	3.8	3.52	4.83	5.24
	12.3	12.1	21.3	7.71	7.97	13.9	17.1
LQt	1	0.697	1	1.19+0.33[illegible]	1.34+0.27[illegible]	1.13	1.17
	1	1	1.14	1.19-0.331	1.34-0.275	3.86+3.13i	1.17
	1.7	3	3.46	2.31+2.27i	2.16+1.97i	3.86-3.13i	6.83
	5.3	4.3	12.4	2.31-2.27i	2.16-1.97i	5.14	6.83
AQt	0	0	-1	0	0	0	-1
	0	0	0	0	0	0	0
	0	1	0	1	0.382	0	0
	3	2	5	2	2.62	4	5
ALt	-3	-2	-5	-2	-2.62	-2	-5
	0	-1	0	-1	-0.382	-2	0
	0	0	0	0	0	0	0
	0	0	1	0	0	0	1

92	94	204	206	222	280	282	286	328
0	-1	0	0	-1	0	-1	-1.41	0
0	0	0	0	0	0	0	0	0
0	0	0	0	0	0	0	0	0
0	1	0	0	1	0	1	1.41	0
1	1	2	2	2	1	1	1	1
2	2	2	2	2	1	1	1.44	1
2	2.38	2	3	3	1	1.59	2	2
3	4.62	2	3	5	3	4.41	5.56	2
1	1	2	2	2	1	1	1	1
2	2	2	2	2	1	1	1.44	1
2	2.38	2	3	3	1	1.59	2	2
3	4.62	2	3	5	3	4.41	5.56	2
0	0	0	0	0	0	0	0	0
0	0	0	0	0	0	0	0	1
1	1.38	0	0.438	1	1	2	2	1
3	3.62	4	4.56	5	2	2	3	1
0.838	0.907	1.53	2.09	2.48	0.536	0.656	0.875	0.536
2	2.6	4	4	4	1	1.23	2.3	1
7.16	9.21	4	12	14.5	1.39	2.85	4	4
12	22.3	10.5	12.9	25	12.1	21.3	31.8	7.46
0.856	0.914	1.53	2.82	3.38	0.536	0.656	0.875	0.536
2.73	3.47	4	4	4	1	1.23	2.3	1
4.4	6.27	4	6.48	9	1.39	2.85	4	4
14	24.3	10.5	17.7	29.6	12.1	21.3	31.8	7.46
1.09	1.07	2+3.46i	4	4	0.697	0.779	1.03	1.02+0.48i
3.94+3.28i	5.64+1.95i	2-3.46i	4.82+4.56i	7.5+1.94i	1	1.5	2.97	1.02-0.48i
3.94-3.28i	5.64-1.95i	4	4.82-4.56i	7.5-1.94i	3	3.42	4	2.48+2.54i
5.02	12.6	4	7.35	15	4.3	12.3	21	2.48-2.54i
0	-2	0	-0.702	-3	0	-0.828	-2	0
0	0	0	0	0	0	0	0	1
0	0	0	0	0	1	0	0	1
4	7	4	5.7	9	2	4.83	7	1
-2+1.41i	-5	-4	-2.5+1.32i	-5	-2	-4.83	-7	-1
-2-1.41i	0	0	-2.5-1.32i	-1	-1	0	0	-1
0	0	0	0	0	0	0	0	-1
0	0	0	0	0	0	0.828	2	0

330	332	334	344	346	348	350	390	392
-0.5+0.866	-1	-0.662+0.5	0	-0.662+0.5	-1	-1	-1	0
-0.5-0.866	0	-0.662-0.5	0	-0.662-0.5	0	-0.618	0	0
0	0	0	0	0	0	0	0	0
1	1	1.32	0	1.32	1	1.62	1	0
1	1	1	1	1	1	1	1	1
1.25	1	1.59	2	1.59	2	2	1.38	1
2.88+0.74	2	3	2	3	2.38	3.38	2	2
2.88-0.745	4	4.41	3	4.41	4.62	5.62	3.62	2
1	1	1	1	1	1	1	1	1
1.77+0.79	1	2.12+0.59i	2	2.12+0.59i	2	2.58+0.42	1.38	1
1.77-0.793	2	2.12-0.59i	2	2.12-0.59i	2.38	2.58-0.425	2	2
3.47	4	4.77	3	4.77	4.62	5.83	3.62	2
0	0	0	0	0	0	0	0	0
1	0.382	0.586	0.382	0.382	0.198	0.268	1	0
1	1	1	1	2	1.55	2	1	1
2	2.62	3.41	2.62	2.62	3.25	3.73	2	2
0.778	0.719	0.892	0.77	0.855	0.865	0.917	0.706	0.642
1.79	1	2.47	2.31	2.59	2.76	3.84	1.92	0.851
6.93	5.21	8.77	6.54	8.71	9.12	12.6	5.21	3.8
12.5	17.1	22.9	12.4	22.8	22.3	32.7	14.2	7.71
0.873	0.719	0.92	0.852	0.91	0.906	0.939	0.706	0.642
2.91	1	3.59	2.44	3.62	3.48	6.47	1.92	0.851
5.06	5.21	6.75	5.1	6.79	6.32	7.51	5.21	3.8
13.2	17.1	23.7	13.6	23.7	24.3	35.1	14.2	7.71
1.11	1.15+0.18	1.08	1.06	1.05	1.05	1.05	1.46	1.19+0.33
4.03	1.15-0.189	5.79+1.66i	4.06+3.41i	5.85+1.83i	5.7+2.06i	7.75+2.76i	2.84	1.19-0.331
4.43+3.52i	6.85+0.45	5.79-1.66i	4.06-3.41i	5.85-1.83i	5.7-2.06i	7.75-2.76i	4.85+0.79	2.31+2.27i
4.43-3.52i	6.85-0.459	12.3	4.82	12.3	12.5	21.4	4.85-0.795	2.31-2.27i
0	-0.777	-0.861+1.6	-0.303	-0.787+1.6	-2	-1.85+0.7i	-1	0
0.162+1.9	-0.256	-0.861-1.6	0	-0.787-1.6	0	-1.85-0.7i	0	0
0.162-1.94	0	0	1	0	0.146	0	1	1
3.68	5.03	6.72	3.3	6.57	6.85	9.7	4	2
-2.5+1.94i	-5.03	-5	-1.5+0.866	-4.85	-4.96	-6.74	-4	-2
-2.5-1.94i	0	-2	-1.5-0.866i	-2	-0.0195+0.	-1.91	-1	-1
0	0.256	0	-1	0	-0.0195-0.	0	0	0
1	0.777	2	0	1.85	0	2.64	1	0

394	396	398	404	406	408	410	412	414
0	-1	-1	-1	-1.41	0	-1	-1	-1.41
0	0	0	0	0	0	0	0	0
0	0	0	0	0	0	0	0	0
0	1	1	1	1.41	0	1	1	1.41
1	1	1.59	1	1	1	1	1.59	1.62
2	1.38	2	1.38	1.47	2	2	2	2
2	2	2	2	2.65	2	2.38	2	2.73
3	3.62	4.41	3.62	4.88	3	4.62	4.41	5.65
1	1	1.59	1	1	1	1	1.59	1.62
2	1.38	2	1.38	1.47	2	2	2	2
2	2	2	2	2.65	2	2.38	2	2.73
3	3.62	4.41	3.62	4.88	3	4.62	4.41	5.65
0	0	0	0	0	0	0	0	0
0.382	0.382	0.586	0.382	0.382	0	0.198	0.198	0.268
1	1	1	1	2	1	1.55	1.55	2
2.62	2.62	3.41	2.62	2.62	3	3.25	3.25	3.73
0.77	0.752	1.69	0.752	0.841	0.838	0.865	1.69	2.1
2.31	1.8	3.3	1.8	2.27	2	2.76	3.11	3.76
6.54	5.2	6.56	5.2	7.86	7.16	9.12	7.07	9.82
12.4	14.3	21.5	14.3	24	12	22.3	21.1	32.3
0.852	0.752	2.28	0.752	0.841	0.856	0.906	2.27	2.61
2.44	1.8	2.5	1.8	2.27	2.73	3.48	2.67	3.47
5.1	5.2	6.25	5.2	7.86	4.4	6.32	5.81	8.18
13.6	14.3	22	14.3	24	14	24.3	22.2	33.7
1.06	1.62	3.33	1.62	1.18	1.09	1.05	3.26	3.34
4.06+3.41i	2.73	3.91+2.14i	2.73	3.18	3.94+3.28i	5.7+2.06i	3.93+2.19i	5.76+1.49i
4.06-3.41i	4	3.91-2.14i	4	7.12	3.94-3.28i	5.7-2.06i	3.93-2.19i	5.76-1.49i
4.82	5.65	11.9	5.65	13.5	5.02	12.5	11.9	21.1
-0.303	-1.19	-1.46	-1.19	-2.19	0	-2	-1.46	-3
0	0	0	0	0	0	0	0	0
1	1	1	1	0.404	0	0.146	0.583	0.347
3.3	4.19	5.46	4.19	6.79	4	6.85	5.88	8.65
-1.5+0.866	-4.19	-4.83	-4.19	-6.79	-2+1.41i	-4.96	-3.89	-7
-1.5-0.866i	-1	-1	-1	-0.404	-2-1.41i	-0.0195+0.	-2.17	-0.618
-1	0	0	0	0	0	-0.0195-0.	0	0
0	1.19	0.828	1.19	2.19	0	0	1.06	1.62

454	456	458	460	462	468	470	472	474
-0.662+0.5	0	-0.5+0.866	-1	-0.662+0.5	-1	-1	0	-0.662+0.5
-0.662-0.5	0	-0.5-0.866i	0	-0.662-0.5	0	-0.618	0	-0.662-0.5
0	0	0	0	0	0	0	0	0
1.32	0	1	1	1.32	1	1.62	0	1.32
1	2	1.53	2	2	1	1	2	1.75
1.68	2	2	2	2	2	2	2	2
3.66+0.56	2	3.23+0.79	2	4	3	4	3	3.45
3.66-0.562	2	3.23-0.793	4	4	4	5	3	4.8
1	2	2	2	2	1	1	2	2
2.34+0.56	2	2.12+0.74	2	2.58+0.60	2	2.59	2	2.43+0.36
2.34-0.562	2	2.12-0.745	2	2.58-0.606	3	3	3	2.43-0.369
4.32	2	3.75	4	4.84	4	5.41	3	5.15
0	0	0	0	0	0	0	0	0
1	0.382	1	0	0.438	0.268	0.468	0.268	0.468
1	1	1	1	1	1	1.65	1	1.65
3	2.62	3	4	4.56	3.73	3.88	3.73	3.88
0.849	1.3	1.7	1.78	2.37	0.883	0.912	1.95	2.19
2.99	4.45	4.42	5.41	5.9	3.14	3.97	4.4	4.35
12.6	4.58	11.8	5.93	12.7	13	16.8	11.3	13.7
16.6	9.67	13.1	17.9	23	16	26.3	13.3	25.7
0.882	1.3	2.75	1.78	3.14	0.889	0.925	2.47	3.19
4.43	4.45	4.4	5.41	6.5	4	5.79	4.4	4.35
8.22	4.58	7.15	5.93	8.88	8.09	10.8	6.95	9.61
19.5	9.67	16.7	17.9	25.5	20	30.5	17.2	28.9
1.12	2.65+2.53i	4.45+1.56i	3.18+2.89i	6.13	1.14	1.08	4.78+1.53i	4.6
7.2+4.12i	2.65-2.53i	4.45-1.56i	3.18-2.89i	7.27+3.13i	6.93+3.9i	10.5+1.32i	4.78-1.53i	5.47
7.2-4.12i	3.35+2.8i	6.05+4.63i	7.32+1.35i	7.27-3.13i	6.93-3.9i	10.5-1.32i	5.72+4.33i	11.4
7.48	3.35-2.8i	6.05-4.63i	7.32-1.35i	11.3	8	13.9	5.72-4.33i	12.5
-0.629+1.5	0	-0.0659+2.	-0.854	-0.952+1.7	-2	-1.75+0.53	-0.093+0.9	-1.24+1.6i
-0.629-1.5	0.379+1.1	-0.0659-2.	0	-0.952-1.7	0	-1.75-0.53	-0.093-0.9	-1.24-1.6i
0	0.379-1.18	0	0	0	0.628	0	0	0
6.26	3.24	5.13	5.85	7.9	6.37	9.51	5.19	8.49
-3.5+1.66i	-3.24	-2.74+2.26	-5.85	-4	-4	-5.93	-2.39	-4.82
-3.5-1.66i	-0.379+1.1	-2.74-2.26i	0	-3.83	-2	-2.94	-1.3+1.44i	-2.34
0	-0.379-1.1	0	0	0	0	0	-1.3-1.44i	0
2	0	0.476	0.854	1.83	1	2.87	0	1.15

476	478	856	858	862	904	906	908	910
-1	-1	-1	-1	-1	0	0	-1	-1
0	-0.618	0	-0.618	-1	0	0	0	0
0	0	0	0	0	0	0	0	0
1	1.62	1	1.62	2	0	0	1	1
2	2	1	1	1	2	2	2	2
2.38	2.27	2	2	2.27	2	2	2	2.38
3	4	3	4	5	2	3	2	3
4.62	5.73	4	5	5.73	2	3	4	4.62
2	2	1	1	1	2	2	2	2
2.38	3	2	2.59	3	2	2	2	2.38
3	3	3	3	3.59	2	3	2	3
4.62	6	4	5.41	6.41	2	3	4	4.62
0	0	0	0	0	0	0	0	0
0	0.238	1	0.586	1	0	0.198	0.382	0.468
1.27	1.64	1	2	1.59	2	1.55	2	1.65
4.73	5.12	3	3.41	4.41	2	3.25	2.62	3.88
2.45	2.81	0.824	0.891	0.93	1.31	1.79	1.7	2.35
6	6.23	3.42	4.09	5.39	4	4.9	5.53	6.49
13.3	18.4	12.8	16.7	25	5.06	11.7	6.17	12.8
22.3	33.5	16	26.4	33.7	9.63	12.6	17.6	22.4
3.11	3.59	0.876	0.924	0.948	1.31	2.44	1.7	2.97
6.34	7.09	4	5.76	9	4	4.68	5.53	6.47
8.46	11.9	8.28	10.9	13.4	5.06	6.56	6.17	8.79
26.1	38.4	19.8	30.4	41.7	9.63	17.3	17.6	25.8
5.85	4.8	1.09	1.06	1.05	2.47+3.85i	3.25	3.81+2.82i	4.76
7.1+3.45i	10.2+1.74i	6.95+4.18i	10.5+1.77i	13	2.47-3.85i	5.04+5.16i	3.81-2.82i	7.49+4.31i
7.1-3.45i	10.2-1.74i	6.95-4.18i	10.5-1.77i	15	3.07	5.04-5.16i	6.69+0.88	7.49-4.31i
11.9	21.7	8	14	22	4	7.67	6.69-0.886	12.3
-2.1	-2.86	-2	-2	-3	0	-0.919	-0.635	-2.16
0	-1.56	0	-1.39	-3	0	0	0	0
0	0	1	0	0	2	1.14	0.295	0.613
8.1	11.4	6	9.39	13	2	4.78	5.34	7.55
-5	-6.5	-4	-6	-6.29	-2	-2.09+2.05	-5.34	-5
-1	-2.95	-1	-2.65	-5	-2	-2.09-2.05i	-0.295	-0.5+1.32i
0	0	0	0	0	0	-0.815	0	-0.5-1.32i
0	2.45	0	2.65	4.29	0	0	0.635	0

922	924	926	972	974	990	2184	2186	2190
-1	-0.662+0.5	-1	-1.41	-1	-1	0	0	0
0	-0.662-0.5	-0.618	0	-0.618	-1	0	0	0
0	0	0	0	0	0	0	0	0
1	1.32	1.62	1.41	1.62	2	0	0	0
2	2	2	2	2	2	1	1	2
2	2	2.27	2	2.38	2.59	1	2	2
3	4	4	3	4.62	5.41	1	2	3
5	4	5.73	5	5	6	3	3	3
2	2	2	2	2	2	1	1	2
2	2.58+0.60	3	2	3	3.27	1	2	2
3	2.58-0.606	3	3	3.38	4	1	2	3
5	4.84	6	5	5.62	6.73	3	3	3
0	0	0	0	0	0	0	0	0
1	0.586	1	0	0.209	1	0	0	0
1	2	1.59	2	2	1.44	0	0.586	1
4	3.41	4.41	4	4.79	5.56	3	3.41	4
2.12	2.25	2.68	2.24	2.74	3.05	0.734	0.875	2.13
4.85	6.27	6.62	6.91	7.67	8.43	1	2	4
14	12.8	18.2	9	20.5	30.5	1	6.53	10.8
25	22.6	33.5	25.9	28	36	12.3	12.6	14.1
2.95	3.06	3.51	2.24	3.3	3.64	0.734	0.886	2.56
4.6	6.96	7.28	6.91	9.31	11.3	1	2.43	4
9	8.54	11.9	9	12.5	16	1	4.83	7.43
29.4	25.4	38.3	25.9	33.9	47.1	12.3	13.9	17
3.36	4.78	4.43	4.6+2.79i	5.01	4.52	1	1.13	4
7.82+3.21i	7.67+4.22i	10.4+2.86i	4.6-2.79i	13+3.16i	14	1	3.86+3.13i	5.1+4.65i
7.82-3.21i	7.67-4.22i	10.4-2.86i	9	13-3.16i	19.5	1.7	3.86-3.13i	5.1-4.65i
15	11.9	21.7	13.8	14	24	5.3	5.14	6.8
-3	-0.84+1.79	-3	-2	-1.92+0.96	-4	0	0	0
0	-0.84-1.79i	-1.16	0	-1.92-0.96	-2.72	0	0	0
0.469	0	0	0	0	0	0	0	0
8.53	7.68	11.2	8	10.8	14.7	3	4	5
-5	-3.81+1.68	-6.4	-8	-6.73	-6	-3	-2+2.17e-0	-2.5+1.32i
-0.5+1.94i	-3.81-1.68i	-2.52	0	-2.76	-6	0	-2-2.17e-0	-2.5-1.32i
-0.5-1.94i	0	0	0	0	0	0	0	0
0	1.62	1.92	2	2.48	4	0	0	0

2202	2204	2206	2252	2254	2270	2458	2462	2506
-1	0	-1	0	0	-1	-1	-1.41	-0.5+0.866
0	0	0	0	0	0	0	0	-0.5-0.866i
0	0	0	0	0	0	0	0	0
1	0	1	0	0	1	1	1.41	1
1	2	2	2	3	3	2	2.27	2
2	2	2.38	2	3	3	2.38	3	3
3	3	3	3	3	3	3	3	3.5+0.866i
4	3	4.62	3	3	5	4.62	5.73	3.5-0.866i
1	2	2	2	3	3	2	2.27	2.5+0.866i
2	2	2.38	2	3	3	2.38	3	2.5-0.866i
3	3	3	3	3	3	3	3	3
4	3	4.62	3	3	5	4.62	5.73	4
0	0	0	0	0	0	0	0	0
0.268	0.268	0.325	0	0.308	0.551	0	0	1
1	1	1.46	0.438	0.643	1	1.27	2	1
3.73	3.73	4.21	4.56	5.05	5.45	4.73	5	4
0.883	1.95	2.53	2.09	3.01	3.69	2.45	3.38	2.71
3.14	4.4	5.8	4	12.3	12.5	6	9	13
13	11.3	13.3	12	12.5	15.8	13.3	13.7	13
16	13.3	22.4	12.9	14.2	25	22.3	32.9	13.3
0.889	2.47	2.97	2.82	6.29	6.45	3.11	4.46	6.79
4	4.4	6.34	4	6.48	9	6.34	9	7
8.09	6.95	9	6.48	7.36	9.93	8.46	9.44	7
20	17.2	25.7	17.7	21.9	31.6	26.1	36.1	21.2
1.14	4.78+1.53i	5.44	4	6.44+6.8i	9.06+6.39i	5.85	7.43+4.1i	7+4.8i
6.93+3.9i	4.78-1.53i	7.47+3.62i	4.82+4.56i	6.44-6.8i	9.06-6.39i	7.1+3.45i	7.43-4.1i	7-4.8i
6.93-3.9i	5.72+4.33i	7.47-3.62i	4.82-4.56i	8.56+1.23i	9.88	7.1-3.45i	9	8+5.2i
8	5.72-4.33i	11.6	7.35	8.56-1.23i	15	11.9	21.1	8-5.2i
-2	-0.093+0.9	-1.91	-0.702	-0.535+0.5	-3	-2.1	-3.45	-0.5+2.6i
0	-0.093-0.9	0	0	-0.535-0.5	0	0	0	-0.5-2.6i
0.628	0	0.275	0	0	0.101	0	0	0
6.37	5.19	7.63	5.7	7.07	9.9	8.1	10.4	7
-4	-2.39	-5	-2.5+1.32i	-2.19+2.26	-5	-5	-7.53	-2.5+2.6i
-2	-1.3+1.44i	-1	-2.5-1.32i	-2.19-2.26i	-1+2i	-1	0	-2.5-2.6i
0	-1.3-1.44i	0	0	-1.61	-1-2i	0	0	-1
1	0	0	0	0	0	0	0.531	0

2510	2524	2526	3038	4370	4374	4382	4418	4420
-0.662+0.5	-1	-1	-1	-1	-1.41	-1.73	-0.5+0.866	-1
-0.662-0.5	0	-0.618	-1	0	0	0	-0.5-0.866i	0
0	0	0	0	0	0	0	0	0
1.32	1	1.62	2	1	1.41	1.73	1	1
2.38	3	2.75	3	1	1	1.35	1	1
3	3	3	3	1	1.44	2	1.25	1
4	3	4.45	6	1.59	2	2	2.88+0.745	2
4.62	5	5.8	6	4.41	5.56	6.65	2.88-0.745	4
2.9+0.665i	3	3	3	1	1	1.35	1	1
2.9-0.665i	3	3.43+0.369	4	1	1.44	2	1.77+0.793	1
3	3	3.43-0.369	4	1.59	2	2	1.77-0.793	2
5.21	5	6.15	7	4.41	5.56	6.65	3.47	4
0	0	0	0	0	0	0	0	0
0.412	0.172	0.313	1	0	0	0	1	0
1.41	1	1.57	1	1	2	3	1	2
5.18	5.83	6.12	7	3	3	3	2	2
3.52	3.62	4.36	5.09	0.793	0.875	1.83	0.778	0.764
13	13.8	14.7	15.9	1	2.3	4	1.79	0.938
14.8	14.6	20.6	36	2.9	4	4	6.93	5.24
25.6	25	34.3	36	21.3	31.8	44.2	12.5	17.1
6.79	7.04	7.83	8.38	0.793	0.875	1.83	0.873	0.764
9.27	9	11.7	16	1	2.3	4	2.91	0.938
9.68	9	12.5	16	2.9	4	4	5.06	5.24
31.3	32	42	52.6	21.3	31.8	44.2	13.2	17.1
8.63+5.49i	8.82+6.28i	11.1+5.44i	13.5+2.6i	1	1.03	2.58	1.11	1.17
8.63-5.49i	8.82-6.28i	11.1-5.44i	13.5-2.6i	1.14	2.97	4	4.03	1.17
12.9+2.91i	10.4	13.9	24	3.46	4	4	4.43+3.52i	6.83
12.9-2.91i	15	21.8	24	12.4	21	31.4	4.43-3.52i	6.83
-1.51+1.98	-3	-2.64+0.34	-4	-1	-2	-3	0	-1
-1.51-1.98i	-0.292	-2.64-0.34i	-4	0	0	0	0.162+1.94	0
0	0	0	0	0	0	0	0.162-1.94	0
10	10.3	13.3	17	5	7	9	3.68	5
-3.75+1.31	-5	-6.56	-6	-5	-7	-9	-2.5+1.94i	-5
-3.75-1.31i	-1+2i	-2.44	-6	0	0	0	-2.5-1.94i	0
0	-1-2i	0	0	0	0	0	0	0
0.506	0	1	3	1	2	3	1	1

4422	4424	4426	4428	4430	4434	4436	4438	4440
-0.662+0.5	-1	-0.662+0.5	-1.41	-1	-0.662+0.5	-1	-1	-1
-0.662-0.5	0	-0.662-0.5	0	-0.618	-0.662-0.5	0	-0.618	0
0	0	0	0	0	0	0	0	0
1.32	1	1.32	1.41	1.62	1.32	1	1.62	1
1	1	1.2	1	1.44	1	1	1	1.59
1.59	1.38	2	1.47	2	1.59	2	2	2
3	2	2.55	2.65	3	3	2.38	3.38	2
4.41	3.62	4.25	4.88	5.56	4.41	4.62	5.62	4.41
1	1	1.73+0.56	1	2	1	1	1	1.59
2.12+0.59i	1.38	1.73-0.564	1.47	2	2.12+0.59i	2	2.58+0.42	2
2.12-0.59i	2	2	2.65	2.27	2.12-0.59i	2.38	2.58-0.425	2
4.77	3.62	4.55	4.88	5.73	4.77	4.62	5.83	4.41
0	0	0	0	0	0	0	0	0
0.382	1	1	0.382	0.586	0.586	0	0.268	0.586
2	1	2	2	2	1	1.38	2	1
2.62	2	2	2.62	3.41	3.41	3.62	3.73	3.41
0.855	0.706	1.42	0.841	1.98	0.892	0.907	0.917	1.69
2.59	1.92	3.23	2.27	3.73	2.47	2.6	3.84	3.3
8.71	5.21	6.88	7.86	9.53	8.77	9.21	12.6	6.56
22.8	14.2	21.5	24	32.8	22.9	22.3	32.7	21.5
0.91	0.706	2.43	0.841	2.67	0.92	0.914	0.939	2.28
3.62	1.92	2.82	2.27	3.59	3.59	3.47	6.47	2.5
6.79	5.21	6.07	7.86	8.48	6.75	6.27	7.51	6.25
23.7	14.2	21.7	24	33.3	23.7	24.3	35.1	22
1.05	1.46	3.28	1.18	3.35	1.08	1.07	1.05	3.33
5.85+1.83i	2.84	4.12+1.93i	3.18	5.8+1.33i	5.79+1.66i	5.64+1.95i	7.75+2.76i	3.91+2.14i
5.85-1.83i	4.85+0.79	4.12-1.93i	7.12	5.8-1.33i	5.79-1.66i	5.64-1.95i	7.75-2.76i	3.91-2.14i
12.3	4.85-0.795	11.5	13.5	21	12.3	12.6	21.4	11.9
-0.787+1.6	-1	-0.205+1.4	-2.19	-1.16+1.02	-0.861+1.6	-2	-1.85+0.7i	-1.46
-0.787-1.6	0	-0.205-1.4	0	-1.16-1.02i	-0.861-1.6	0	-1.85-0.7i	0
0	1	0	0.404	0	0	0	0	1
6.57	4	5.41	6.79	8.33	6.72	7	9.7	5.46
-4.85	-4	-3.36+0.54	-6.79	-7.19	-5	-5	-6.74	-4.83
-2	-1	-3.36-0.54i	-0.404	-1.6	-2	0	-1.91	-1
0	0	0	0	0	0	0	0	0
1.85	1	1.72	2.19	2.79	2	0	2.64	0.828

4442	4444	4446	4546	4548	4550	4556	4558	4562
-1	-1.41	-1.53	-0.63+1.09	-1	-0.761+0.8	-1.41	-0.885+0.5	-0.761+0.8
-0.618	0	-0.347	-0.63-1.09i	0	-0.761-0.8	0	-0.885-0.5	-0.761-0.8
0	0	0	0	0	0	0	0	0
1.62	1.41	1.88	1.26	1	1.52	1.41	1.77	1.52
1.44	1.62	1.6	1.3	2	1.51	2	1.83	1.59
2	2	2.4	2	2	2.48	2	3	2
3	2.73	3.32	3.35+1.03i	2	4.01+0.51	3	3.69	4
5.56	5.65	6.68	3.35-1.03i	4	4.01-0.513	5	5.48	4.41
2+1.98e-0	1.62	1.7	2	2	2.25+0.75i	2	2.54+0.68	2
2-1.98e-08	2	2.75+0.37	2+1i	2	2.25-0.75i	2	2.54-0.688	2.37+0.69
2.27	2.73	2.75-0.373	2-1i	2	2.54	3	3	2.37-0.692
5.73	5.65	6.8	4	4	4.95	5	5.92	5.27
0	0	0	0	0	0	0	0	0
0.586	0.268	0.382	1	0.382	1	0	0.438	1
2	2	2.62	2	2	2	2	2	1
3.41	3.73	4	2	2.62	3	4	4.56	4
1.98	2.1	2.49	1.6	1.7	2.11	2.24	3.07	2.22
3.73	3.76	5.17	4	5.53	5.89	6.91	9	4
9.53	9.82	12.6	11.8	6.17	13.1	9	13.2	13.3
32.8	32.3	44.8	13.6	17.6	22.9	25.9	33.7	26.4
2.67	2.61	2.86	3.21	1.7	3.4	2.24	4.84	3.55
3.59	3.47	6.47	4	5.53	6.45	6.91	9	4
8.48	8.18	9.22	7.79	6.17	9.2	9	9.84	10.2
33.3	33.7	46.5	16	17.6	24.9	25.9	35.3	28.2
3.35	3.34	3.39	4	3.81+2.82i	5.12	4.6+2.79i	7.54+3.96i	4
5.8+1.33i	5.76+1.49i	7.9+2.78i	5.6	3.81-2.82i	8.14+3.68i	4.6-2.79i	7.54-3.96i	7
5.8-1.33i	5.76-1.49i	7.9-2.78i	5.7+4.97i	6.69+0.88	8.14-3.68i	9	9	9.44
21	21.1	31.8	5.7-4.97i	6.69-0.886	10.6	13.8	20.9	13.6
-1.16+1.02	-3	-3.5	-5.55e-17	-0.635	-0.761+2.6	-2	-1.58+2.36	-1.13+2.54
-1.16-1.02	0	-0.903	-5.55e-17-	0	-0.761-2.6	0	-1.58-2.36	-1.13-2.54
0	0.347	0	0	0.295	0	0	0	0
8.33	8.65	11.4	5	5.34	7.52	8	10.2	8.27
-7.19	-7	-8.92	-3.34+2.83	-5.34	-4.19+2.09	-8	-7.25	-4.36+1.29
-1.6	-0.618	-1.64	-3.34-2.83	-0.295	-4.19-2.09	0	-3.07	-4.36-1.29
0	0	0	0	0	0	0	0	0
2.79	1.62	3.56	1.67	0.635	2.38	2	3.32	2.71

4564	4566	4572	4574	4678	4682	4686	4692	4694
-1	-0.885+0.5	-1.41	-1	-1	-1	-1	-0.662+0.5	-1
0	-0.885-0.5	0	-1	0	-1	-1	-0.662-0.5	-0.618
0	0	0	0	0	1	1	0	0
1	1.77	1.41	2	1	1	1	1.32	1.62
2	1.74	2.27	2.1	1	1.38	1.59	1	1
2.38	2.48	3	3	2	1.38	2	1.68	2
3	4.18	3	4.19	3	3.62	4	3.66+0.56	4
4.62	5.6	5.73	6.71	4	3.62	4.41	3.66-0.562	5
2	2.46+0.18	2.27	3	1	1.38	1.59	1	1
2.38	2.46-0.183	3	3+9.44e-0	2	1.38	2	2.34+0.56	2.59
3	3	3	3-9.44e-0	3	3.62	4	2.34-0.562	3
4.62	6.08	5.73	7	4	3.62	4.41	4.32	5.41
0	0	0	0	0	0.382	0.268	0	0
0.325	0.697	0	0.319	1	1	1	1	0.586
1.46	2	2	2.36	1	1	1	1	2
4.21	4.3	5	5.32	3	2.62	3.73	3	3.41
2.53	2.69	3.38	3.91	0.824	1.54	2.14	0.849	0.891
5.8	6.14	9	9	3.42	2.29	4.24	2.99	4.09
13.3	18.1	13.7	19.6	12.8	10.9	16	12.6	16.7
22.4	34.1	32.9	45.5	16	16.3	21.6	16.6	26.4
2.97	3.7	4.46	5.97	0.876	1.54	2.46	0.882	0.924
6.34	7.09	9	9	4	2.29	4	4.43	5.76
9	12.5	9.44	13.4	8.28	10.9	13	8.22	10.9
25.7	37.8	36.1	49.6	19.8	16.3	24.6	19.5	30.4
5.44	4.66	7.43+4.1i	9	1.09	3.18+0.06	3.31	1.12	1.06
7.47+3.62i	10.5+1.65i	7.43-4.1i	10.4+2.71i	6.95+4.18i	3.18-0.069	8	7.2+4.12i	10.5+1.77i
7.47-3.62i	10.5-1.65i	9	10.4-2.71i	6.95-4.18i	7.32+2.86i	10.3+3.4i	7.2-4.12i	10.5-1.77i
11.6	21.4	21.1	32.2	8	7.32-2.86i	10.3-3.4i	7.48	14
-1.91	-1.99+1.96	-3.45	-2.9+0.839	-2	-1.27+0.54	-2	-0.629+1.5	-2
0	-1.99-1.96i	0	-2.9-0.839i	0	-1.27-0.54	-1.56	-0.629-1.5	-1.39
0.275	0	0	0	1	2.76	2.56	0	0
7.63	11	10.4	13.8	6	4.77	7	6.26	9.39
-5	-6	-7.53	-8.62	-4	-4.77	-4.61	-3.5+1.66i	-6
-1	-4.27	0	-3.41	-1	-2.76	-4	-3.5-1.66i	-2.65
0	0	0	0	0	1.27+0.54	1.31+1.15i	0	0
0	3.27	0.531	4.02	0	1.27-0.546	1.31-1.15i	2	2.65

4698	4700	4702	4740	4742	4748	4750	4758	4764
-1.62	-1.01+0.51	-1.25	-1	-0.724	-1.27	-1	-0.618	-1
-0.618	-1.01-0.51	-1	5.55e-17+	-0.248+1.0	-2.92e-17+	-0.233+0.7	-0.5+0.866	-0.233+0.7
0.618	0.525	0.445	5.55e-17-1	-0.248-1.0	-2.92e-17-	-0.233-0.7	-0.5-0.866	-0.233-0.7
1.62	1.49	1.8	1	1.22	1.27	1.47	1.62	1.47
1.38	1.45	1.59	1	1.25	1.28	1.59	1.38	1.45
1.7	2	2.27	2+1i	2.88+0.74	2.3+0.755i	3	2.38	3+0.644i
3.62	4.27+0.56	4.41	2-1i	2.88-0.745	2.3-0.755i	3	3.62	3-0.644i
5.3	4.27-0.564	5.73	3	3	4.11	4.41	4.62	4.55
1.38	1.75	1.68	1	1.52	1.28	1.71	1.7	2
1.7	2	2.42	2+1i	2.29+1i	2.3+0.755i	2.69+0.85	2.5+0.866i	2.58+0.60
3.62	3.45	3.87	2-1i	2.29-1i	2.3-0.755i	2.69-0.851	2.5-0.866i	2.58-0.606
5.3	4.8	6.03	3	3.9	4.11	4.9	5.3	4.84
0.382	0.198	0.173	1	0.382	0.382	0.268	0.382	0.198
0.382	1	0.662	1	1	1	1	0.382	1
2.62	1.55	2.21	1	1	1	1	2.62	1.55
2.62	3.25	3.96	1	2.62	2.62	3.73	2.62	3.25
1.91	2.06	2.45	1	1.52	1.52	2.24	1.91	2.03
2.88	4	5.13	5	4.73	5.88	6.01	4.58	6.42
13.1	16.5	19.4	5	10.9	6.16	12.9	13.1	13.1
28.1	21.4	34	9	13.8	17.4	22.9	26.4	22.4
1.91	2.48	2.76	1	2.27	1.52	2.82	2.88	3.1
2.88	4.35	5.79	5	4.89	5.88	6.94	4.81	6.5
13.1	12.7	15.1	5	7.57	6.16	9.12	10.2	9.07
28.1	24.4	37.3	9	16.3	17.4	25.1	28.1	25.3
3.28	3.29	3.37	3+4i	3.17	4.6+2.52i	4.58	3.28	4.38
3.28	7.62	8.77	3-4i	5.62+5.18i	4.6-2.52i	8+4.08i	8.5+2.18i	8.05+4.33i
13.7	10.5+3.69i	14	3	5.62-5.18i	5.9+0.332i	8-4.08i	8.5-2.18i	8.05-4.33i
13.7	10.5-3.69i	20.8	3	6.59	5.9-0.332i	11.4	13.7	11.5
-2.79	-1.5+1.6i	-2.52+0.64	-1	-1.11	-1.72	-1.7	-1.11	-2.07
-1.11	-1.5-1.6i	-2.52-0.64	1+2i	0.614+2.5	0.666+1.7	0.0599+2.	-0.5+2.6i	0.285+2.0
1.79	1.65	1.3	1-2i	0.614-2.54	0.666-1.71	0.0599-2.2	-0.5-2.6i	0.285-2.08
8.11	7.34	10.7	3	4.89	5.38	7.58	8.11	7.5
-8.11	-4.6+2.03i	-7.43	-3	-2.29+2.41	-5.38	-4.64	-4.62	-4.74
-1.79	-4.6-2.03i	-3.8	-1+2i	-2.29-2.41i	-0.666+1.7	-1.88+1.46	-2.38	-1.63+1.97
1.11	0.955	1	-1-2i	-1.95	-0.666-1.7	-1.88-1.46i	-1.79	-1.63-1.97i
2.79	2.24	3.23	1	1.53	1.72	2.39	2.79	2

4766	4812	4814	4830	4946	4950	4952	4954	4958
-1	-1.41	-1	-1	-1	-1	-1	-1.25	-1.48
-0.42+0.60	0	-0.618	-1	-0.618	-1	-1	-1	-1
-0.42-0.60	0	0	0	0	0	1+7.34e-0	0.445	0.311
1.84	1.41	1.62	2	1.62	2	1-7.34e-09	1.8	2.17
1.62	1.59	1.85	2	1	1	1.59	1.59	1.65
2.73	3	3	3	2	2.27	2	2.27	2.6
4	3	4.57+0.36	5	4	5	4	4.41	5
5.65	4.41	4.57-0.369	6	5	5.73	4.41	5.73	6.74
2	1.59	2.2	2.44	1	1	1.59	1.68	1.73
2.95+0.56	3	3	3	2.59	3	2	2.42	3
2.95-0.565	3	3.55	4	3	3.59	4	3.87	4.14
6.1	4.41	5.25	6.56	5.41	6.41	4.41	6.03	7.13
0.173	0	0	0	0	0	0.268	0.173	0.0968
0.662	1	1	0.764	0.468	1	1	0.662	1
2.21	1	1.27	2	1.65	1.59	1	2.21	2.19
3.96	4	4.73	5.24	3.88	4.41	3.73	3.96	4.71
2.54	2.38	3.18	3.82	0.912	0.93	2.14	2.45	2.73
6.43	6.46	8.33	8.68	3.97	5.39	4.24	5.13	6.78
18	12.5	22.1	26.2	16.8	25	16	19.4	25
34.1	20.6	23.4	37.3	26.3	33.7	21.6	34	45.5
3.53	2.38	4.48	5.79	0.925	0.948	2.46	2.76	2.98
7.29	6.46	7.51	8	5.79	9	4	5.79	9
12.5	12.5	15	18	10.8	13.4	13	15.1	17.1
37.7	20.6	30	44.2	30.5	41.7	24.6	37.3	50.9
4.29	6.5+2.6i	8.88+0.70	8	1.08	1.05	3.31	3.37	3.44
10.6+2.52i	6.5-2.6i	8.88-0.704	9.68	10.5+1.32i	13	8	8.77	13.4
10.6-2.52i	8.5+2.96i	12.6+4.71i	20	10.5-1.32i	15	10.3+3.4i	14	15
21.4	8.5-2.96i	12.6-4.71i	22.3	13.9	22	10.3-3.4i	20.8	32.2
-2.36	-2	-1.3+0.536	-3	-1.75+0.53	-3	-2	-2.52+0.64	-4.26
-0.784+1.9	0	-1.3-0.536i	-2.39	-1.75-0.53	-3	-1.56	-2.52-0.64	-3
-0.784-1.9	1	0	0	0	0	2.56	1.3	0.83
10.9	7	9.6	13.4	9.51	13	7	10.7	14.4
-6.71	-7	-5	-7	-5.93	-6.29	-4.61	-7.43	-8.61
-1.89+0.82	-1	-4.46	-5	-2.94	-5	-4	-3.8	-5
-1.89-0.82	0	0	0	0	0	1.31+1.15i	1	0.683
3.49	2	2.46	4	2.87	4.29	1.31-1.15i	3.23	4.93

4994	4998	5002	5004	5006	5010	5012	5014	5016
-0.5+0.866	-1	-0.662+0.5	-1.55	-1.35	-0.662+0.5	-1	-1	-1
-0.5-0.866i	-0.233+0.7	-0.662-0.5	-5.38e-17+	-0.183+0.6	-0.662-0.5	-0.233+0.7	-0.42+0.60	0
0	-0.233-0.7	0	-5.38e-17-	-0.183-0.6	0	-0.233-0.7	-0.42-0.60	0
1	1.47	1.32	1.55	1.71	1.32	1.47	1.84	1
1.53	1.45	2	1.55	1.85	1.75	1.59	1.62	2
2	3+0.644i	2	2.7+0.495i	3.24+0.51	2	3+3.34e-0	2.73	2.38
3.23+0.79	3-0.644i	4	2.7-0.495i	3.24-0.517	3.45	3-3.34e-08	4	3
3.23-0.793	4.55	4	5.05	5.66	4.8	4.41	5.65	4.62
2	2	2	1.55	2.19	2	1.71	2	2
2.12+0.74	2.58+0.60	2.58+0.60	2.7+0.495i	2.97+0.65	2.43+0.36	2.69+0.85	2.95+0.56	2.38
2.12-0.745	2.58-0.606	2.58-0.606	2.7-0.495i	2.97-0.655	2.43-0.369	2.69-0.851	2.95-0.565	3
3.75	4.84	4.84	5.05	5.86	5.15	4.9	6.1	4.62
0	0.198	0	0.382	0.173	0	0.268	0.173	0
1	1	0.586	0.382	0.662	0.468	1	0.662	0.468
1	1.55	2	2.62	2.21	1.65	1	2.21	1.65
3	3.25	3.41	2.62	3.96	3.88	3.73	3.96	3.88
1.7	2.03	2.25	2.12	3.06	2.19	2.24	2.54	2.35
4.42	6.42	6.27	6.7	9.5	4.35	6.01	6.43	6.49
11.8	13.1	12.8	9.61	13.2	13.7	12.9	18	12.8
13.1	22.4	22.6	25.6	33.2	25.7	22.9	34.1	22.4
2.75	3.1	3.06	2.12	4.39	3.19	2.82	3.53	2.97
4.4	6.5	6.96	6.7	9.39	4.35	6.94	7.29	6.47
7.15	9.07	8.54	9.61	9.67	9.61	9.12	12.5	8.79
16.7	25.3	25.4	25.6	35.6	28.9	25.1	37.7	25.8
4.45+1.56i	4.38	4.78	5.76+2.12i	7	4.6	4.58	4.29	4.76
4.45-1.56i	8.05+4.33i	7.67+4.22i	5.76-2.12i	8.47+4.41i	5.47	8+4.08i	10.6+2.52i	7.49+4.31i
6.05+4.63i	8.05-4.33i	7.67-4.22i	6.71	8.47-4.41i	11.4	8-4.08i	10.6-2.52i	7.49-4.31i
6.05-4.63i	11.5	11.9	13.8	21.1	12.5	11.4	21.4	12.3
-0.0659+2.	-2.07	-0.84+1.79	-2.69	-3.07	-1.24+1.6i	-1.7	-2.36	-2.16
-0.0659-2.	0.285+2.0	-0.84-1.79	0.489+1.6	-5.12e-05+	-1.24-1.6i	0.0599+2.	-0.784+1.9	0
0	0.285-2.08	0	0.489-1.61	-5.12e-05-	0	0.0599-2.2	-0.784-1.9	0.613
5.13	7.5	7.68	7.71	10.1	8.49	7.58	10.9	7.55
-2.74+2.26	-4.74	-3.81+1.68	-7.71	-7.32	-4.82	-4.64	-6.71	-5
-2.74-2.26i	-1.63+1.97	-3.81-1.68	-0.489+1.6	-1.33+1.86	-2.34	-1.88+1.46	-1.89+0.82	-0.5+1.32i
0	-1.63-1.97i	0	-0.489-1.6	-1.33-1.86i	0	-1.88-1.46i	-1.89-0.82	-0.5-1.32i
0.476	2	1.62	2.69	2.97	1.15	2.39	3.49	0

5018	5020	5022	5058	5062	5064	5066	5068	5070
-1	-1.35	-1.45	-0.761+0.8	-1	-1	-1.03+0.73	-1.73	-1
-0.618	-0.183+0.6	-0.302+0.4	-0.761-0.8	-0.42+0.60	-1	-1.03-0.73	0	-1
0	-0.183-0.6	-0.302-0.4	0	-0.42-0.60	1	0.372	0	0
1.62	1.71	2.05	1.52	1.84	1	1.68	1.73	2
2	1.85	2	1.51	1.59	2+1.83e-0	1.83	1.83	2
2.27	3.24+0.51	3.27	2.48	3	2-1.83e-08	2.76	3	3.38
4	3.24-0.517	4	4.01+0.51	4.41	4+3.74e-0	4.7+0.785i	3.69	5
5.73	5.66	6.73	4.01-0.513	5	4-3.74e-08	4.7-0.785i	5.48	5.62
2	2.19	2.4	2.25+0.75i	2.27	2+1.83e-0	2.47+0.55	1.83	2.7
3+2.28e-0	2.97+0.65	3.29+0.57	2.25-0.75i	3	2-1.83e-08	2.47-0.552	3	3
3-2.28e-08	2.97-0.655	3.29-0.573	2.54	3	4+3.74e-0	3.72	3.69	4
6	5.86	7.02	4.95	5.73	4-3.74e-08	5.35	5.48	6.3
0	0.173	0.0968	0	0.121	0.382	0.121	0	0
1	0.662	1	1	1	0.382	1	0.382	0.657
1.59	2.21	2.19	2	2.35	2.62	2.35	2.62	2.53
4.41	3.96	4.71	3	3.53	2.62	3.53	4	4.81
2.68	3.06	3.73	2.11	2.47	2.44	3.07	3.06	3.87
6.62	9.5	9.44	5.89	7.6	6.56	8.27	9.22	11.2
18.2	13.2	19.3	13.1	21.1	12.5	22.2	14.4	23.8
33.5	33.2	45.5	22.9	27.8	20.5	23.4	30.3	35.2
3.51	4.39	5.66	3.4	3.46	2.44	4.74	3.06	5.47
7.28	9.39	9.44	6.45	9	6.56	7.75	9.22	11.1
11.9	9.67	13.4	9.2	13.2	12.5	14.8	14.4	17
38.3	35.6	49.5	24.9	33.3	20.5	29.7	30.3	40.5
4.43	7	7.19	5.12	4.55	7.5+2.78i	8.27	7.38+2.71i	9.25
10.4+2.86i	8.47+4.41i	11.3+3.89i	8.14+3.68i	13.1+4.24i	7.5-2.78i	9.73	7.38-2.71i	14.6+1.22i
10.4-2.86i	8.47-4.41i	11.3-3.89i	8.14-3.68i	13.1-4.24i	7.5+2.78i	12.5+5.03i	14.1+0.07	14.6-1.22i
21.7	21.1	32.2	10.6	14.2	7.5-2.78i	12.5-5.03i	14.1-0.072	19.6
-3	-3.07	-4.37	-0.761+2.6	-2.36	-1.5+1.32i	-1.85+2.72	-3.05	-2.33+1.62
-1.16	-5.12e-05+	-0.628+1.6	-0.761-2.6	-0.558+2.1	-1.5-1.32i	-1.85-2.72i	0	-2.33-1.62i
0	-5.12e-05-	-0.628-1.6	0	-0.558-2.1i	2.44	1.24	0.373	0
11.2	10.1	13.6	7.52	10.5	6.56	9.45	9.67	12.7
-6.4	-7.32	-8.73	-4.19+2.09	-6.23	-6.56	-5.37+2.67	-9.67	-8.24
-2.52	-1.33+1.86	-1.72+1.36	-4.19-2.09i	-2.12+0.61	-2.44	-5.37-2.67i	-0.373	-3.76
0	-1.33-1.86i	-1.72-1.36i	0	-2.12-0.61	1.5+1.32i	1.33	0	0
1.92	2.97	4.18	2.38	3.46	1.5-1.32i	2.41	3.05	4

5074	5076	5078	5080	5082	5084	5086	6342	6348
-0.885+0.5	-1.35	-1	-1+4.84e-0	-1.14+0.48	-1.53	-1.3	-0.475	-0.662+0.5
-0.885-0.5	-0.183+0.6	-0.574+0.3	-1-4.84e-0	-1.14-0.48	-0.347	-1	-0.46+1.14	-0.662-0.5
0	-0.183-0.6	-0.574-0.3	1+1.37e-0	0.337	0	0	-0.46-1.14	0
1.77	1.71	2.15	1-1.37e-08	1.94	1.88	2.3	1.4	1.32
1.74	1.81	1.75	2.38+8.04	2.1	2.15	2.21	1.6	2
2.48	2.71	2.89	2.38-8.04e	2.82	3.23	3.38	3.46+1.14i	2
4.18	4.29	5.52	4.62	5.18	4.77	5.62	3.46-1.14i	4
5.6	5.19	5.84	4.62	5.9	5.85	6.79	3.47	4
2.46+0.18	1.85	2.09	2.38+7.68	2.63+0.31	2.32	2.67	2.53	2
2.46-0.183	3.24+0.51	3.42	2.38-7.68e	2.63-0.318	3.46	3.48	2.54+1.14i	2.58+0.606
3	3.24-0.517	3.68	4.62	4.2	4	4.42	2.54-1.14i	2.58-0.606
6.08	5.66	6.81	4.62	6.53	6.21	7.43	4.4	4.84
0	0.228	0.087	0.228	0.087	0	0	0.198	0
0.697	0.544	1.42	0.544	1.42	0.486	1.21	1	0.438
2	1.84	1.68	1.84	1.68	2.43	2	1.55	1
4.3	4.39	4.81	4.39	4.81	5.09	5.79	3.25	4.56
2.69	2.78	2.93	3.38	3.84	4.04	4.65	2.39	2.37
6.14	7.26	8.19	8.54	8.76	11.2	11.7	12.3	5.9
18.1	21.3	30.1	22.1	26.1	23.9	32.3	13.5	12.7
34.1	27.6	36.8	22.9	37.3	34.9	46.4	13.8	23
3.7	3.15	3.7	4.38	5.86	5.25	6.64	6.28	3.14
7.09	9.27	11.2	7.35	8.01	11	12.3	7.47	6.5
12.5	13	16.7	15	18	17	20.4	7.65	8.88
37.8	33.5	46.4	30.2	44.2	40.7	55.6	20.6	25.5
4.66	4.69	4.37	8.64	7.99	9.56	8.69	7.15+7.15i	6.13
10.5+1.65i	13.2+3.8i	14.1	9.97	9.9	14.3+0.47	14.5	7.15-7.15i	7.27+3.13i
10.5-1.65i	13.2-3.8i	21.4	12.2+4.61i	19.7	14.3-0.479	21.1	7.85+0.82i	7.27-3.13i
21.4	13.9	22.2	12.2-4.61i	22.4	19.9	32.7	7.85-0.82i	11.3
-1.99+1.96	-3.07	-3.04	-2.26+0.75	-3.17+1.78	-3.72	-4	-1.17	-0.952+1.7
-1.99-1.96i	-0.26+1.84	-1.66+1.17	-2.26-0.75	-3.17-1.78i	-0.973	-3.76	0.187+3.2	-0.952-1.7
0	-0.26-1.84i	-1.66-1.17i	2.32	1.1	0	0	0.187-3.29	0
11	10.6	14.4	9.2	13.2	12.7	16.8	6.79	7.9
-6	-6.76	-6.05+0.63	-5.75	-6.26+1.41	-8.66	-8.37	-3.11+3.48	-4
-4.27	-1.76+1.35	-6.05-0.63	-3.8	-6.26-1.41i	-2.57	-6	-3.11-3.48i	-3.83
0	-1.76-1.35i	-0.884	1.28+1.98i	1.42	0	0	-1.73	0
3.27	3.28	4.98	1.28-1.98i	3.1	3.23	5.37	1.96	1.83

6350	6356	6358	6364	6366	6550	6552	6554	6558
-0.618	-0.5+0.866	-0.618	-0.662+0.5	-1	-0.885+0.5	-1	-1	-1
-0.5+0.866	-0.5-0.866i	-0.5+0.866	-0.662-0.5	-0.42+0.60	-0.885-0.5	0	-0.618	-1
-0.5-0.866i	0	-0.5-0.866i	0	-0.42-0.60	0	0	0	0
1.62	1	1.62	1.32	1.84	1.77	1	1.62	2
2	2	1.89	2.38	2.27	1.83	2	2	2.1
4+9.2e-06i	3	3.7+0.755i	3	4	3	2	2.27	3
4-9.2e-06i	3.5+0.866i	3.7-0.755i	4	4	3.69	3	4	4.19
4	3.5-0.866i	4.72	4.62	5.73	5.48	5	5.73	6.71
2.59	2.5+0.866i	2.82+0.90	2.9+0.665i	3	2.54+0.68	2	2	3+1.18e-0
3+1i	2.5-0.866i	2.82-0.903	2.9-0.665i	3.37+0.69	2.54-0.688	2	3	3-1.18e-0
3-1i	3	3	3	3.37-0.692	3	3	3	3
5.41	4	5.36	5.21	6.27	5.92	5	6	7
0.308	0	0.139	0	0.18	0	0	0	0
0.643	1	1	0.412	0.599	0.438	0	0.238	0.319
1	1	1.75	1.41	1.67	2	1	1.64	2.36
5.05	4	4.11	5.18	5.55	4.56	5	5.12	5.32
3.37	2.71	3.19	3.52	4.22	3.07	2.48	2.81	3.91
12.5	13	12.9	13	14	9	4	6.23	9
14.7	13	15.2	14.8	21.2	13.2	14.5	18.4	19.6
26.4	13.3	25.6	25.6	34.6	33.7	25	33.5	45.5
6.41	6.79	6.88	6.79	7.69	4.84	3.38	3.59	5.97
10	7	9.38	9.27	11.9	9	4	7.09	9
10	7	10	9.68	13	9.84	9	11.9	13.4
30.6	21.2	30.7	31.3	41.4	35.3	29.6	38.4	49.6
9.49+6.06i	7+4.8i	9.86+5.52i	8.63+5.49i	11.8+5.21i	7.54+3.96i	4	4.8	9
9.49-6.06i	7-4.8i	9.86-5.52i	8.63-5.49i	11.8-5.21i	7.54-3.96i	7.5+1.94i	10.2+1.74i	10.4+2.71i
10.3	8+5.2i	11.6+2.18i	12.9+2.91i	12.8	9	7.5-1.94i	10.2-1.74i	10.4-2.71i
13.7	8-5.2i	11.6-2.18i	12.9-2.91i	21.5	20.9	15	21.7	32.2
-1.6	-0.5+2.6i	-1.68	-1.51+1.98	-3	-1.58+2.36	-3	-2.86	-2.9+0.839
-0.621+2.9	-0.5-2.6i	-0.514+2.8	-1.51-1.98i	-0.99+2.17	-1.58-2.36i	0	-1.56	-2.9-0.839i
-0.621-2.9	0	-0.514-2.8	0	-0.99-2.17i	0	0	0	0
9.84	7	9.71	10	13	10.2	9	11.4	13.8
-3.77+1.98	-2.5+2.6i	-3.8	-3.75+1.31	-6.36	-7.25	-5	-6.5	-8.62
-3.77-1.98i	-2.5-2.6i	-2.82+2.38	-3.75-1.31i	-2.52+1.82	-3.07	-1	-2.95	-3.41
-2.4	-1	-2.82-2.38i	0	-2.52-1.82i	0	0	0	0
2.94	0	2.45	0.506	3.39	3.32	0	2.45	4.02

6598	6602	6604	6606	6614	6616	6618	6620	6622
-0.686+1.0	-0.618	-1	-0.745+0.7	-0.745+0.7	-1	-1	-1	-1
-0.686-1.0	-0.5+0.866	-0.618	-0.745-0.7	-0.745-0.7	0	-0.42+0.60	-0.618	-0.574+0.3
-0.373	-0.5-0.866i	0	-0.459	-0.459	0	-0.42-0.60	0	-0.574-0.3
1.75	1.62	1.62	1.95	1.95	1	1.84	1.62	2.15
1.79	1.89	2	2.12	2.12	3	2.27	2.75	2.47
4	3.7+0.755i	2.38	4	4	3	4	3	4.39+0.29
4.1+0.665i	3.7-0.755i	4.62	4.35	4.35	3	4	4.45	4.39-0.291
4.1-0.665i	4.72	5	5.53	5.53	5	5.73	5.8	6.74
2.76+1.07i	2.82+0.90	2	3.12+0.83	3.12+0.83	3	3	3	3.53
2.76-1.07i	2.82-0.903	3	3.12-0.834	3.12-0.834	3	3.37+0.69	3.43+0.36	3.61+0.71
3	3	3.38	3.42	3.42	3	3.37-0.692	3.43-0.369	3.61-0.714
5.49	5.36	5.62	6.34	6.34	5	6.27	6.15	7.24
0.121	0.139	0	0.191	0.191	0	0.18	0	0.235
1	1	0.209	0.473	0.473	0.551	0.599	0.313	0.321
2.35	1.75	2	2.11	2.11	1	1.67	1.57	2.08
3.53	4.11	4.79	5.22	5.22	5.45	5.55	6.12	6.37
3.05	3.19	2.74	4.04	4.04	3.69	4.22	4.36	5.24
13.4	12.9	7.67	14.2	14.2	12.5	14	14.7	20.3
14.4	15.2	20.5	20.6	20.6	15.8	21.2	20.6	21.2
26.2	25.6	28	35.2	35.2	25	34.6	34.3	46.3
7.38	6.88	3.3	8.03	8.03	6.45	7.69	7.83	12.3
9	9.38	9.31	12.3	12.3	9	11.9	11.7	12.9
10.3	10	12.5	12.6	12.6	9.93	13	12.5	14.1
30.3	30.7	33.9	41.1	41.1	31.6	41.4	42	53.7
8.77	9.86+5.52i	5.01	11.9+5.02i	11.9+5.02i	9.06+6.39i	11.8+5.21i	11.1+5.44i	13.5+6.38i
10+6.57i	9.86-5.52i	13+3.16i	11.9-5.02i	11.9-5.02i	9.06-6.39i	11.8-5.21i	11.1-5.44i	13.5-6.38i
10-6.57i	11.6+2.18i	13-3.16i	13.1	13.1	9.88	12.8	13.9	15.4
14.2	11.6-2.18i	14	21.1	21.1	15	21.5	21.8	32.6
-1.14	-1.68	-1.92+0.96	-1.69+2.91	-1.69+2.91	-3	-3	-2.64+0.34	-3.57
-0.762+3.5	-0.514+2.8	-1.92-0.96	-1.69-2.91i	-1.69-2.91i	0	-0.99+2.17	-2.64-0.34	-1.87+1.57
-0.762-3.5	-0.514-2.8	0	-1.55	-1.55	0.101	-0.99-2.17i	0	-1.87-1.57i
9.67	9.71	10.8	12.9	12.9	9.9	13	13.3	16.3
-4.43+3.16	-3.8	-6.73	-4.92+0.99	-4.92+0.99	-5	-6.36	-6.56	-8.05
-4.43-3.16i	-2.82+2.38	-2.76	-4.92-0.99	-4.92-0.99	-1+2i	-2.52+1.82	-2.44	-2.73+1.22
-1.32	-2.82-2.38i	0	-2.07	-2.07	-1-2i	-2.52-1.82i	0	-2.73-1.22i
3.17	2.45	2.48	3.91	3.91	0	3.39	1	4.5

6854	6858	6862	6870	6874	6876	6878	7126	7128
-1	-1	-1	-1	-1.53	-0.885+0.5	-1	-1	-1
-0.348+1.0	-0.618	-0.42+0.60	-0.5+0.866	-0.347	-0.885-0.5	-0.574+0.3	-0.635+0.6	-1
-0.348-1.0	0	-0.42-0.60	-0.5-0.866i	0	0	-0.574-0.3	-0.635-0.6	1+1.18e-08
1.7	1.62	1.84	2	1.88	1.77	2.15	2.27	1-1.18e-08
1.74	1.85	2.16	2	2.15	2.23	2.38	2.27	3
3.63+1.09i	3	4.42+0.60	4.12+0.74	3.23	4	4.62	4	3
3.63-1.09i	4.57+0.36	4.42-0.606	4.12-0.745	4.77	4.88+0.59i	5	5.73	5
5	4.57-0.369	5	5.75	5.85	4.88-0.59i	6	6	5
2.82+0.90	2.2	3	3.12+0.83	2.32	3.12+0.59i	3.58+0.42	3.42+0.55i	3
2.82-0.903	3	3.58+0.60	3.12-0.834	3.46	3.12-0.59i	3.58-0.425	3.42-0.55i	3
3	3.55	3.58-0.606	3.42	4	4	4	4	5+2.11e-0
5.36	5.25	5.84	6.34	6.21	5.77	6.83	7.15	5-2.11e-08
1	0	0.172	0.438	0	0	0.13	0.402	0.172
1	1	1	1	0.486	1	0.593	1	1
1	1.27	1	2	2.43	1.7	2.09	1.68	1
4	4.73	5.83	4.56	5.09	5.3	6.19	5.92	5.83
2.98	3.18	4.28	3.86	4.04	4.31	5.21	4.78	4.68
13.5	8.33	16.5	14.5	11.2	16.6	19.4	15.3	17.3
15.5	22.1	25	21.1	23.9	25.5	28.2	36	25
25	23.4	26.3	34.6	34.9	25.6	38.2	36.9	25
7.27	4.48	9	8.16	5.25	9.42	10.5	8.53	9
9	7.51	9.77	11.6	11	9.46	13.1	16	9
9.84	15	16.6	13.1	17	16.5	18.9	16.8	16.8
30.9	30	36.7	41.1	40.7	36.6	48.4	51.7	37.2
7.83	8.88+0.70	13.6+7.4i	10.3	9.56	13.2+5.07i	15.5+5.02i	11.5	13+7.48i
10.1+6.95i	8.88-0.704	13.6-7.4i	13+5.93i	14.3+0.47	13.2-5.07i	15.5-5.02i	18.5	13-7.48i
10.1-6.95i	12.6+4.71i	13.9	13-5.93i	14.3-0.479	14.8+5.61i	21+1.27i	21	15
15	12.6-4.71i	15	21.7	19.9	14.8-5.61i	21-1.27i	24	15
-3	-1.3+0.536	-3	-3.33	-3.72	-2.06+2.39	-3.22	-4	-3
0.169+3.1	-1.3-0.536	-0.624+2.4	-0.735+3.1	-0.973	-2.06-2.39	-1.74+2i	-1.77+2.7i	-3
0.169-3.11	0	-0.624-2.4	-0.735-3.1	0	0	-1.74-2i	-1.77-2.7i	2.1
9.66	9.6	12.2	12.8	12.7	12.1	15.7	16.5	11.9
-5	-5	-5	-6.28	-8.66	-5.13+2.22	-7.31	-6	-5
-2.38+3.53	-4.46	-3.2+1.57i	-2.86+2.81	-2.57	-5.13-2.22	-3.08+0.80	-4.2+1.29i	-5
-2.38-3.53	0	-3.2-1.57i	-2.86-2.81	0	0	-3.08-0.80	-4.2-1.29i	1+2.83i
2.76	2.46	3.39	4	3.23	2.27	4.46	5.39	1-2.83i

7130	7134	13140	13150	13150	13150	13260	13260	13280
-1+1.24e-0	-1	-1	-1	-1	-1.3	-2	-1.68	-1.56
-1-1.24e-0	-1	-1	-1	-1	-1	0	-0.539	-1
0	-0.414	0	0	0	0	0	0	0
2	2.41	2	2	2	2.3	2	2.21	2.56
2.59	2.66	2	2	2	2.21	2	2.27	2.44
4	4.53	2.59	3	3.38	3.38	4	4	4
5.41	6	5.41	5	5	5.62	4	5.73	6.56
6	6.81	6	6	5.62	6.79	6	6	7
3.27	4	2	2.44	2.7	2.67	2	2.66	3
4+3.72e-08	4	3.27	3	3	3.48	4	4	4
4-3.72e-08	4.27	4	4	4	4.42	4	4.53	5
6.73	7.73	6.73	6.56	6.3	7.43	6	6.81	8
0	0.0815	0	0	0	0	0	0	0
0.773	1	1	0.764	0.657	1.21	0	0.222	1
1.82	1.7	1.44	2	2.53	2	4	3.29	2.44
6.41	7.22	5.56	5.24	4.81	5.79	4	5.49	6.56
5.4	6.33	3.05	3.82	3.87	4.65	4	5.06	5.95
19.7	22.5	8.43	8.68	11.2	11.7	16	16	16
27.9	36	30.5	26.2	23.8	32.3	16	30.5	43.1
38	47.1	36	37.3	35.2	46.4	36	39.4	49
10.3	13.1	3.64	5.79	5.47	6.64	4	6.89	9
13	16	11.3	8	11.1	12.3	16	16	16
19.1	21.7	16	18	17	20.4	16	20.5	25
48.7	61.2	47.1	44.2	40.5	55.6	36	47.6	64
14.9+5.44i	17.5+4.66i	4.52	8	9.25	8.69	12	12.4	12.7
14.9-5.44i	17.5-4.66i	14	9.68	14.6+1.22i	14.5	12	16	16
21.6+1.92i	24	19.5	20	14.6-1.22i	21.1	16	22.3+4.34i	30.3
21.6-1.92i	33	24	22.3	19.6	32.7	16	22.3-4.34i	35
-3.36+1.1i	-4	-4	-3	-2.33+1.62	-4	-4	-3.2+0.628	-5
-3.36-1.1i	-3.85	-2.72	-2.39	-2.33-1.62i	-3.76	0	-3.2-0.628i	-4.84
0	-1.75	0	0	0	0	0	0	0
15.7	19.6	14.7	13.4	12.7	16.8	12	15.4	19.8
-7.37	-7.69	-6	-7	-8.24	-8.37	-12	-10.6	-9.64
-4.52	-6	-6	-5	-3.76	-6	0	-3.1	-7
0	-1.91	0	0	0	0	0	0	0
2.88	5.59	4	4	4	5.37	4	4.73	6.64

14680	14690	14790	14800	14810	14810	14810	15260	15260
-1	-1.3	-1+1i	-0.953+0.6	-1	-1	-1	-1	-1
-0.5+0.866	-0.5+0.866	-1-1i	-0.953-0.6	-0.635+0.6	-0.574+0.3	-0.756+0.4	-1	-1
-0.5-0.866i	-0.5-0.866i	0	-0.328	-0.635-0.6	-0.574-0.3	-0.756-0.4	0	-0.414
2	2.3	2	2.23	2.27	2.15	2.51	2	2.41
2	2.21	2	2.27	2.27	2.38	2.51	3	2.66
4.12+0.74	4.5+0.866i	4	5	4	4.62	5	3	4.53
4.12-0.745	4.5-0.866i	5+1i	5	5.73	5	5.66	6	6
5.75	6.79	5-1i	5.73	6	6	6.83	6	6.81
3.12+0.83	3.5+0.866i	3+1i	3.38+0.78	3.42+0.55i	3.58+0.42	3.8+0.629i	3	4
3.12-0.834	3.5-0.866i	3-1i	3.38-0.786	3.42-0.55i	3.58-0.425	3.8-0.629i	4	4
3.42	3.7	4	4.35	4	4	4.61	4	4.27
6.34	7.3	6	6.89	7.15	6.83	7.79	7	7.73
0.438	1	0	0.0688	0.402	0.13	0.266	0	0.0815
1	1	2	1.2	1	0.593	1.4	1	1
2	1.7	2	2.18	1.68	2.09	1.6	1	1.7
4.56	5.3	4	5.55	5.92	6.19	6.74	7	7.22
3.86	4.88	4	5.05	4.78	5.21	6.16	5.09	6.33
14.5	21	16	19.2	15.3	19.4	22.2	15.9	22.5
21.1	21	26	28.4	36	28.2	36.6	36	36
34.6	46.1	26	38.3	36.9	38.2	47.1	36	47.1
8.16	13	10	10.8	8.53	10.5	13.2	8.38	13.1
11.6	13	10	13.5	16	13.1	16.5	16	16
13.1	13.7	16	18.6	16.8	18.9	21.4	16	21.7
41.1	53.3	36	48.1	51.7	48.4	60.9	52.6	61.2
10.3	12.4	12	16.8+5i	11.5	15.5+5.02i	19.3+3.48i	13.5+2.6i	17.5+4.66i
13+5.93i	15+6.93i	14+8i	16.8-5i	18.5	15.5-5.02i	19.3-3.48i	13.5-2.6i	17.5-4.66i
13-5.93i	15-6.93i	14-8i	17.2	21	21+1.27i	20.3	24	24
21.7	32.6	16	22.2	24	21-1.27i	33.1	24	33
-3.33	-5.05	-2+4i	-2.53+3.02	-4	-3.22	-4.63	-4	-4
-0.735+3.1	-1+3.46i	-2-4i	-2.53-3.02i	-1.77+2.7i	-1.74+2i	-2.42+2.45	-4	-3.85
-0.735-3.1	-1-3.46i	0	-1.5	-1.77-2.7i	-1.74-2i	-2.42-2.45i	0	-1.75
12.8	16	12	15.6	16.5	15.7	19.5	17	19.6
-6.28	-8.37	-6+4i	-6.2+2.09i	-6	-7.31	-7.24	-6	-7.69
-2.86+2.81	-3+3.46i	-6-4i	-6.2-2.09i	-4.2+1.29i	-3.08+0.80	-5	-6	-6
-2.86-2.81i	-3-3.46i	0	-1.54	-4.2-1.29i	-3.08-0.80	-4.18	0	-1.91
4	5.37	4	4.94	5.39	4.46	6.42	3	5.59

15310	15330	31710
-1	-1	-1
-1	-1	-1
-0.414	-0.732	-1
2.41	2.73	3
2.59	2.79	3
5.41	5.54	7
6	6.68	7
6	7	7
4	4.32+0.358	5
4	4.32-0.358	5
4.59	5	5
7.41	8.37	9
0.146	0.28	1
0.382	1	1
2.62	1.8	1
6.85	7.92	9
6.41	7.57	9
30.5	30.5	49
30.6	45.9	49
42.5	49	49
12.5	17.1	25
20.4	20.5	25
20.5	25	25
56.6	70.4	81
21.5+3.71i	22.4+4.08i	27
21.5-3.71i	22.4-4.08i	35
23.5+4.87i	31.2	35
23.5-4.87i	35	35
-3.5+1.94i	-5	-5
-3.5-1.94i	-3.53+1.09	-5
-1.81	-3.53-1.09i	-5
18.8	23.1	27
-9	-7.91	-7
-4	-7	-7
-2.53	-3.51	-7
5.53	7.42	9

pattern id	14	28	30	74	76	78	90	92
E	[0;0;0;1]	[0;0;1;0]	[0;0;-0.707	[0;0;0;1]	[0;0;0;1]	[0;0;0;1]	[0;0;-0.707	[0;0;1;0]
	[0;0;0;-1]	[0;0;-1;0]	[0.707;0;-0	[0;0;0;-1]	[0;0;0;-1]	[0;0;0;-1]	[0.707;0;-0	[0;0;-1;0]
	[0;0;0;-1]	[0;0;1;0]	[0;0.707;-0	[0;0;0;-1]	[0;0;1;0]	[0;0;0;-1]	[0;0.707;0;	[0;0;1;0]
	[0;0;0;-1]	[0;0;1;0]	[0;0;0.707;	[0;0;0;1]	[0;0;-0.707	[0;0;0;1]	[0;0;0.707;	[0;0;1;0]
LE	[0.8944;0;0	[0;0;1;0]	[0.8165;0;0	[0.7071;0;0	[0.7071;0;0	[0.8944;0;0	[0.8018;0;0	[0.8165;0;0
	[0;0.8944;0	[0;0;1;0]	[0;0.8165;0	[0;0.5774;0	[0;0;1;0]	[0;0;0.707	[0;0.8018;0	[0;0;1;0]
	[0;0;0.894	[0;0;1;0]	[0;0;-0.923	[0;0;0;1]	[0;0;0;1]	[0;0;0.707	[0;0;0.707	[0;0;1;0]
	[0;0;0;1]	[0;0;-0.447	[0;0;0.382	[0;0;0;1]	[0;0;0;1]	[0;0;0;1]	[0;0;0.707	[0;0;-0.707
QE	[0.8944;0;0	[0;0;1;0]	[0.8165;0;0	[0.7071;0;0	[0.7071;0;0	[0.8944;0;0	[0.8018;0;0	[0.8165;0;0
	[0;0.8944;0	[0;0;1;0]	[0;0.8165;0	[0;0.5774;-	[0;0;1;0]	[0;0;0.707	[0;0.8018;-	[0;0;1;0]
	[0;0;0.894	[0;0;1;0]	[0;0;-0.923	[0;0;0;1]	[0;0;0;1]	[0;0;-0.707	[0;0;-0.707	[0;0.7071;0
	[0;0;0;1]	[0;0;0.447	[0;0;-0.382	[0;0;0;-1]	[0;0;0;-1]	[0;0;0;1]	[0;0;0.707	[0;0;0.707
AAt	[1;0;0;0]	[1;0;0;0]	[1;0;0;0]	[1;0;0;0]	[1;0;0;0]	[1;0;0;0]	[1;0;0;0]	[1;0;0;0]
	[0;1;0;0]	[0;1;0;0]	[0;1;0;0]	[0;1;0;0]	[0;1;0;0]	[0;1;0;0]	[0;1;0;0]	[0;1;0;0]
	[0;0;1;0]	[0;0;1;0]	[0;0;1;0]	[0;0;1;0]	[0;0;-0.851	[0;0;-0.924	[0;0;1;0]	[0;0;-0.707
	[0;0;0;1]	[0;0;0;1]	[0;0;0;1]	[0;0;0;1]	[0;0;0.526;	[0;0;0.383;	[0;0;0;1]	[0;0;0.707;
LLt	[0.571;0.5	[-0.566;-0.	[-0.685;-0.	[-0.579;-0.	[0.664;0.4	[0.978;0.1	[-0.688;-0.	[-0.962;-0.
	[-0.211;0.7	[0.707;-0.7	[0.707;-0.7	[0.78;-0.60	[-0.701;0.3	[-0.192;0.7	[0.706;-0.7	[-0.258;0.7
	[0.789;-0.2	[0.415;0.4	[-0.169;-0.	[-0.207;-0.	[0.235;-0.6	[-0.0117;0	[-0.162;-0.	[-0.0183;-0
	[-0.0877;-0	[-0.0859;-0	[-0.0461;-0	[-0.116;0.0	[-0.111;-0.	[-0.0838;-0	[-0.0439;0	[-0.0861;-0
QQt	[0.571;0.5	[0.566;0.5	[-0.685;-0.	[0.579;-0.7	[-0.664;-0.	[0.991;0.0	[0.688;-0.6	[-0.985;-0.
	[-0.211;0.7	[0.707;-0.7	[0.707;-0.7	[0.78;0.60	[0.701;-0.3	[0.0264;0.	[0.706;0.7	[0.0517;0.
	[0.789;-0.2	[-0.415;-0.	[-0.169;-0.	[-0.207;0.2	[-0.235;0.6	[-0.112;0.5	[-0.162;0.1	[0.155;-0.5
	[0.0877;0.0	[0.0859;0.0	[0.0461;0.0	[0.116;0.0	[0.111;0.5	[0.0696;0.	[0.0439;0.0	[0.064;0.2
LQt	[0;0;0;-1]	[0;0;1;0]	[0;0.707;-0	[0;0;0;-1]	[0;0;1;0]	[0;0;0;1]	[0;0;0.707;	[0;0;1;0]
	[0;0;0;-1]	[0;0;1;0]	[0.707;0;-0	[0;0;0;1]	[0;0;-0.707	[0;0;0;-1]	[0;0.707;0;	[0;0;-1;0]
	[0;0;0;-1]	[0;0;-1;0]	[0;0;-0.707	[0;0;0;1]	[0;0;0;1]	[0;0;0;1]	[0;0;-0.707	[0;0;1;0]
	[0;0;0;1]	[0;0;1;0]	[0;0;0.707;	[0;0;0;-1]	[0;0;0;-1]	[0;0;0;-1]	[0.707;0;-0	[0;0;1;0]
AQt	[0.948683	[0.534522	[0;0;-0.894	[0.894427	[0.577350	[0;0;-0.707	[0;0;-0.707	[0;0;-0.894
	[0;0.94868	[0;0.53452	[0.771516	[0;0.57735	[0;0.44721	[1.256073	[0.811107	[3.972054
	[0;0;0.948	[0;0;1;0]	[0;0.77151	[0;0;0.447	[0;0;-0.850	[0;3.14018	[0;0.81110	[0;3.97205
	[0;0;0;1]	[0;0;0.948	[0;0;-0.707	[0;0;0;1]	[0;0;-0.525	[0;0;-0.316	[0;0;0.707	[0;0;0.894
ALt	[0;0;0;1]	[0;0;-0.948	[0;0;-0.707	[0;0;0;1]	[0;0;0.525	[0;0;0.707	[0;0;-0.707	[0;0;0.816
	[0.948683	[0;0;1;0]	[0.771516	[0;0;0.447	[0;0;0.850	[0;0;0.707	[0.811107	[0;0;0.816
	[0;0.94868	[0.534522	[0;0.77151	[0.894427	[0.577350	[0.942809	[0;0.81110	[0.904534
	[0;0;0.948	[0;0.53452	[0;0;0.894	[0;0.57735	[0;0.44721	[0;0.57735	[0;0;0.707	[0;0.58834

94	204	206	222	280	282	286	328	330
[0;0;-0.707	[0;0;1;0]	[0;0;0;1]	[0;0;-0.707	[0;0;1;0]	[0;0.577;0.	[0;-0.5;-0.5	[0;0;1;0]	[0;0.577;-0
[0.707;0;-0	[0;0;0;1]	[0;0;0;-1]	[0.577;0;-0	[0;1;0;0]	[0;1;0;0]	[0;-0.707;0	[0;0;-1;0]	[0;0.577;-0
[0;0.577;-0	[0;0;-0.707	[0;0;0;1]	[0;0.577;-0	[0;-0.707;-	[0.707;0;-0	[0.816;-0.4	[0;0;1;0]	[0.707;0;-0
[0;0;0.707;	[0;0;-0.707	[0;0;0;1]	[0;0;0.707;	[0;0.707;0.	[0;0.577;0.	[0;0.5;0.5;0	[0;0;-1;0]	[0;-0.577;-
[0.9129;0;0	[0;0;1;0]	[0.4082;0;0	[0.5774;0;0	[0;0;1;0]	[0;1;0;0]	[0.7559;0.3	[0;0;1;0]	[0.5;0.5;0.5
[0;0.2673;0	[0;0;0;1]	[0;0.4082;0	[0;0.5774;0	[0;1;0;0]	[0;1;0;0]	[0;0.6572;0	[0;0;1;0]	[0;0.5484;0
[0;0;-0.850	[0;0;0.7071	[0;0;0;1]	[0;0;0.7071	[0;0.7071;0	[0;-0.5469	[0;-0.7071	[0;0.7071;-	[0;0.4344-0
[0;0;0.5257	[0;0;0.7071	[0;0;0;1]	[0;0;0.7071	[0;-0.4082	[0;-0.2612	[0;0.261;0.	[0;0.7071;-	[0;0.4344+
[0.9129;0;0	[0;0;1;0]	[0.7071;0;-	[0.9045;0;-	[0;0;1;0]	[0;1;0;0]	[0.7559;0.3	[0;0;1;0]	[0.866;0.28
[0;0.7071;-	[0;0;0;1]	[0;0.7071;-	[0;0.9045;-	[0;1;0;0]	[0;1;0;0]	[0;0.6572;0	[0;0;-1;0]	[0;0.1588-0
[0;0;-0.850	[0;0;-0.707	[0;0;0;1]	[0;0;-0.707	[0;0.7071;0	[0;-0.5469	[0;-0.7071	[0;0.7071;0	[0;0.1588+
[0;0;-0.525	[0;0;-0.707	[0;0;0;-1]	[0;0;0.7071	[0;0.4082;0	[0;0.2612;0	[0;-0.261;-	[0;-0.7071	[0;-0.5261
[1;0;0;0]	[1;0;0;0]	[1;0;0;0]	[1;0;0;0]	[1;0;0;0]	[1;0;0;0]	[1;0;0;0]	[1;0;0;0]	[1;0;0;0]
[0;1;0;0]	[0;1;0;0]	[0;1;0;0]	[0;1;0;0]	[0;-0.707;0	[0;-0.707;0	[0;-0.707;0	[0;1;0;0]	[0;1;0;0]
[0;0;-0.851	[0;0;-0.707	[0;0;-0.788	[0;0;-0.707	[0;0;0;1]	[0;0.707;0.	[0;0.707;0.	[0;0;1;0]	[0;0;1;0]
[0;0;0.526;	[0;0;0.707;	[0;0;0.615;	[0;0;0.707;	[0;0.707;0.	[0;0;0;1]	[0;0;0;1]	[0;0;0;1]	[0;0;0;1]
[0.987;0.10	[0.602;0.60	[0.638;0.63	[-0.661;-0.	[-0.735;-0.	[-0.734;-0.	[0.965;0.10	[0.7;0.375;	[0.923;0.24
[0.148;-0.8	[0;0;-0.707	[-0.707;0.7	[0.707;-0.7	[0;0.707;-0	[0.661;-0.7	[-0.261;0.6	[0.667;-0.3	[-0.376;0.6
[-0.0476;-0	[0.707;-0.7	[0.236;0.23	[-0.251;-0.	[-0.673;0.4	[-0.153;-0.	[0;0.707;-0	[-0.236;0.4	[-0.0159;0.
[-0.0416;-0	[-0.372;-0.	[-0.193;-0.	[0;0;-0.707	[-0.0815;-0	[-0.045;-0.	[-0.0303;-0	[-0.0971;-0	[-0.0807;-0
[0.994;0.01	[0.602;0.60	[-0.678;-0.	[-0.699;-0.	[0.735;0.43	[0.734;0.59	[0.965;0.10	[0.7;0.375;	[0.988;0.08
[0.0065;0.9	[0;0;-0.707	[0.707;-0.7	[0.707;-0.7	[0;0.707;-0	[0.661;-0.7	[-0.261;0.6	[0.667;-0.3	[0.0656;-0.
[-0.101;0.2	[0.707;-0.7	[0.0345;0.0	[0;0;0.707;	[0.673;-0.4	[0.153;0.25	[0;0.707;-0	[0.236;-0.4	[0.116;-0.4
[0.036;0.13	[0.372;0.37	[0.198;0.19	[0.109;0.10	[0.0815;0.1	[0.045;0.20	[0.0303;0.1	[0.0971;0.1	[0.071;0.39
[0;0;-0.707	[0;0;1;0]	[0;0;0;1]	[0.577;0;-0	[0;0;1;0]	[0;0.577;0.	[0;-0.5;-0.5	[0;0;1;0]	[0;0.577;-0
[0.707;0;-0	[0;0;0;1]	[0;0;0;1]	[0;0;0.707;	[0;0.707;0.	[0;0.577;0.	[0;-0.707;0	[0;0;-1;0]	[0.707;0;-0
[0;0.577;-0	[0;0;-0.707	[0;0;0;-1]	[0;0;-0.707	[0;-0.707;-	[0.707;0;-0	[0.816;-0.4	[0;0;1;0]	[0;0.577;-0
[0;0;0.707;	[0;0;-0.707	[0;0;0;1]	[0;0.577;-0	[0;1;0;0]	[0;1;0;0]	[0;0.5;0.5;0	[0;0;-1;0]	[0;-0.577;-
[0;0;-0.780	[0;0;-0.707	[0;0;-0.595	[0;0;-0.707	[0;0.70710	[0;0.63245	[0;-0.6154	[0.2132007	[0.8999540
[0.9333333	[0.6324555	[0.5345224	[0.9539980	[0.3922322	[0;-0.9045	[0;-0.70710	[0;0;1;0]	[0;0.64681
[0;0.92717	[0;0.63245	[0;0.53452	[0;0.95399	[0;-0.6882	[0.8241633	[9.1049502	[0;1.11022	[0;0.64681
[0;0;-0.707	[0;0;0.7071	[0;0;-0.475	[0;0;0.7071	[0;0.70710	[0;-0.6324	[0;0.57735	[0;-1.1102	[0;0.65174
[0;0;-0.707	[0;0;0.7071	[0;0;0.8164	[0;0;-0.707	[0;0.70710	[0;-0.6324	[0;0.57735	[0;0;1;0]	[0;0.65465
[0;0;0.8320	[0;0;0.7071	[0;0;0.8164	[0;0;0.7071	[0;0.68824	[0;-0.9045	[0;-0.70710	[0;1.11022	[0;0.65465
[5.5585629	[0.6324555	[0.7844645	[0.3333333	[0;-0.70710	[0.8241633	[9.1049502	[0;-1.1102	[0.8276058
[0;1.38964	[0;0.63245	[0;0.78446	[0;0.33333	[0.3922322	[0;-0.6324	[0;0.61545	[0.2132007	[0;0.63960

332	334	344	346	348	350	390	392	394
[0;-0.577;0	[0;-0.434+	[0;0;1;0]	[0;-0.656;-	[0;-0.707;0	[0;-0.707;0	[0;0.707;0;	[0;1;0;0]	[0;1;0;0]
[0;0;1;0]	[0;-0.434-0	[0;0;-1;0]	[0;-0.656;-	[0;0;1;0]	[0;-0.753;0	[0;-0.707;0	[0;-1;0;0]	[0;-1;0;0]
[0;0;1;0]	[0.707;0;-0	[0;0;1;0]	[0.707;0;-0	[0;0;1;0]	[0.707;0;-0	[0;0.707;-0	[0;0;1;0]	[0;1;0;0]
[0;0.577;0.	[0;-0.548;-	[0;0;-1;0]	[0;-0.414;-	[0;0.408;0.	[0;0.4;0.64	[0;0.707;0;	[0;1;0;0]	[0;-1;0;0]
[0;0;1;0]	[0.8528;0.	[0.6325;0.	[0.7559;0.	[0.8006;0.	[0.8981;0.	[0.5;0.5;0.	[0;1;0;0]	[0;1;0;0]
[0;0;1;0]	[0;0.3366;	[0;0;1;0]	[0;-0.6786	[0;0;1;0]	[0;0.4082;	[0;0.8507;	[0;0;1;0]	[0;-0.5774
[0;0.5774;-	[0;-0.7071	[0;0;1;0]	[0;-0.5774	[0;-0.2275	[0;-0.5154	[0;-0.7071	[0;-0.7071	[0;-0.5774
[0;0.6882;-	[0;0.5615;-	[0;-0.7071	[0;-0.3574	[0;0.5217;	[0;0.3484;	[0;-0.5257	[0;-0.7071	[0;-0.4472
[0;0;1;0]	[0.9258;0.	[0.8165;0.	[0.9045;0.	[0.898;0.1	[0.9431;0.	[0.5;-0.5;-	[0;1;0;0]	[0;1;0;0]
[0;0;-1;0]	[0;0.08672	[0;0;1;0]	[0;0.2862-	[0;0;1;0]	[0;-0.0863	[0;0.8507;	[0;0;1;0]	[0;-0.5774
[0;-0.5774	[0;0.08672	[0;0;-1;0]	[0;0.2862+	[0;-0.6479	[0;-0.0863	[0;-0.7071	[0;0.7071;	[0;0.5774;-
[0;0.6882;	[0;0.4845;	[0;0.4082;	[0;0.2752;	[0;-0.4653	[0;0.3036;	[0;0.5257;	[0;-0.7071	[0;0.4472;
[1;0;0;0]	[1;0;0;0]	[1;0;0;0]	[1;0;0;0]	[1;0;0;0]	[1;0;0;0]	[1;0;0;0]	[1;0;0;0]	[1;0;0;0]
[0;0;-0.851	[0;0;-0.924	[0;-0.851;0	[0;-0.851;0	[0;0.737;-0	[0;0.789;-0	[0;1;0;0]	[0;0;-0.707	[0;0;-0.851
[0;1;0;0]	[0;1;0;0]	[0;0;0;1]	[0;0;0;1]	[0;-0.591;-	[0;-0.577;-	[0;0;1;0]	[0;1;0;0]	[0;1;0;0]
[0;0;0.526;	[0;0;0.383;	[0;0.526;0.	[0;0.526;0.	[0;0.328;0.	[0;0.211;0.	[0;0;0;1]	[0;0;0.707;	[0;0;0.526;
[-0.706;-0.	[0.981;0.1	[0.943;0.1	[0.97;0.16	[0.975;0.1	[-0.99;-0.0	[-0.942;-0.	[-0.517;-0.	[0.367;0.8
[0.688;-0.2	[-0.184;0.5	[0.322;-0.6	[0.237;-0.7	[-0.217;0.5	[-0.139;0.6	[-0.256;0.7	[-0.252;0.5	[0.689;-0.4
[0.161;-0.6	[-0.0501;0	[-0.015;-0.	[-0.0426;-0	[-0.0347;0	[-0.0012;-0	[-0.217;-0.	[0.595;-0.5	[0.589;-0.0
[-0.0441;-0	[-0.04;-0.4	[-0.077;-0.	[-0.0391;-0	[-0.0409;-0	[-0.0289;-0	[0.0144;-0	[-0.562;-0.	[-0.207;-0.
[-0.706;-0.	[-0.995;-0.	[-0.985;-0.	[-0.993;-0.	[0.993;0.0	[-0.997;-0.	[0.942;-0.1	[0.517;0.5	[0.0882;0.
[0.688;-0.2	[0.0116;-0	[0.0455;-0	[0.0519;-0	[0.0341;0.	[-0.00202;	[0.256;0.7	[0.252;-0.5	[0.781;0.0
[-0.161;0.6	[-0.0903;0	[-0.152;0.4	[-0.0952;0	[-0.108;0.7	[-0.0772;0	[-0.217;0.3	[-0.595;0.5	[-0.563;0.3
[0.0441;0.0	[0.0374;0.	[0.0608;0.	[0.0361;0.	[0.0347;0.	[0.0253;0.	[0.0144;0.	[0.562;0.2	[0.256;0.2
[0;0;1;0]	[0;-0.434+	[0;0;1;0]	[0;-0.656;-	[0;0.408;0.	[0;-0.707;0	[0;0.707;0;	[0;0;1;0]	[0;1;0;0]
[0;0.577;0.	[0;-0.434-0	[0;0;-1;0]	[0;-0.656;-	[0;-0.707;0	[0;-0.753;0	[0;0.707;-0	[0;1;0;0]	[0;1;0;0]
[0;-0.577;0	[0.707;0;-0	[0;0;1;0]	[0.707;0;-0	[0;0;1;0]	[0.707;0;-0	[0;0.707;0;	[0;1;0;0]	[0;-1;0;0]
[0;0;1;0]	[0;-0.548;-	[0;0;-1;0]	[0;-0.414;-	[0;0;1;0]	[0;0.4;0.64	[0;-0.707;0	[0;-1;0;0]	[0;-1;0;0]
[0;-0.5602	[0;-0.3980	[0;0.60889	[0;-0.6568	[0;0.80655	[0;-0.8220	[0;0.83205	[0;0.81649	[0;0.87716
[0;-0.4302	[0;-0.3980	[0.145864	[0;-0.6568	[0.170251	[0;-0.8220	[0.471404	[0.377964	[0.077849
[0.115470	[0.898177	[0;3.51083	[0.925546	[0;-0.1977	[0.932673	[0;-0.7071	[0;1;0;0]	[0;1;0;0]
[0;0.50204	[0;-0.5237	[0;0.39832	[0;0.47759	[0;-0.4288	[0;0.42118	[0;0.70710	[0;0.81649	[0;0.76689
[0;0.50204	[0;-0.5773	[0;0.35355	[0;-0.6813	[0;0.72098	[0;0.65532	[0;0.70710	[0;-0.8164	[0;-0.9045
[0.115470	[0;-0.2932	[0;0.35355	[0;-0.9701	[0;0.41904	[0;-0.4706	[0;-0.7071	[0;1;0;0]	[0;-0.9045
[0;0.43024	[0.862261	[0;2.10650	[0.885722	[0;0.41904	[0.907392	[0.471404	[0;0.81649	[0;1;0;0]
[0;0.56025	[0;0.68599	[0.377964	[0;0.50453	[0.299812	[0;0.52673	[0;-0.8320	[0.377964	[0.228085

396	398	404	406	408	410	412	414	454
[0;0.707;0	[0;0.707;0	[0;-0.577;-	[0;-0.5;-0.5	[0;0;1;0]	[0;0.577;0.	[0;-0.577;-	[0;-0.5;-0.5	[0;-0.434+
[0;0;1;0]	[0;-0.707;0	[0;0;1;0]	[0;-0.707;0	[0;1;0;0]	[0;1;0;0]	[0;0;1;0]	[0;-0.707;0	[0;-0.434-0
[0;0;-1;0]	[0;0.707;-0	[0;0;-1;0]	[0;0.707;-0	[0;-0.707;-	[0;1;0;0]	[0;0;-1;0]	[0;0.707;-0	[0.577;-0.5
[0;0.707;0	[0;0.707;0	[0;0.577;0.	[0;0.5;0.5;	[0;0.707;0.	[0;0.577;0.	[0;0.577;0.	[0;0.5;0.5;	[0;-0.548;-
[0;0;1;0]	[0;0.9239;	[0.7071;0;	[0.7746;0.	[0;1;0;0]	[0;1;0;0]	[0;-0.6786	[0;0.9064;	[0.8006;0.
[0;0.8507;	[0;-0.7071	[0;-0.6479	[0;0.844;0.	[0;0;1;0]	[0.2357;-0	[0;0;1;0]	[0.4082;-0	[0;-0.5484
[0.5774;-0	[0;-0.7071	[0;0;1;0]	[0;-0.4491	[0;0;1;0]	[0;-0.3556	[0;0;1;0]	[0;-0.3419	[0;-0.4344
[0;-0.5257	[0;-0.3827	[0;0.4653;	[0;0.2931;	[0;-0.3333	[0;-0.2289	[0;0.3574;	[0;0.2482;	[0;-0.4344
[0;0;1;0]	[0;0.9239;	[0.7071;0;	[0.7746;-0	[0;1;0;0]	[0;1;0;0]	[0;-0.6786	[0;0.9064;	[0.8819;-0.
[0;0.8507;	[0;-0.7071	[0;-0.6479	[0;0.844;0.	[0;0;1;0]	[0.7071;0;	[0;0;1;0]	[0.7071;0;	[0;-0.4344
[0.5774;-0	[0;0.7071;	[0;0;1;0]	[0;-0.4491	[0.5774;-0	[0;-0.3556	[0;0;-1;0]	[0;-0.3419	[0;-0.4344
[0;0.5257;	[0;0.3827;	[0;-0.4653	[0;-0.2931	[0;0.3333;	[0;0.2289;	[0;-0.3574	[0;-0.2482	[0;-0.5484
[1;0;0;0]	[1;0;0;0]	[1;0;0;0]	[1;0;0;0]	[1;0;0;0]	[1;0;0;0]	[1;0;0;0]	[1;0;0;0]	[1;0;0;0]
[0;0;-0.851	[0;0;-0.924	[0;-0.851;0	[0;-0.851;0	[0;0.577;-0	[0;0.737;-0	[0;0.737;-0	[0;0.789;-0	[0;1;0;0]
[0;1;0;0]	[0;1;0;0]	[0;0;0;1]	[0;0;0;1]	[0;-0.707;0	[0;-0.591;-	[0;-0.591;-	[0;-0.577;-	[0;0;-0.707
[0;0;0.526;	[0;0;0.383;	[0;0.526;0.	[0;0.526;0.	[0;0.408;0.	[0;0.328;0.	[0;0.328;0.	[0;0.211;0.	[0;0;0.707;
[-0.549;-0.	[0.659;0.5	[0.958;0.0	[0.977;0.1	[-0.286;-0.	[-0.273;-0.	[-0.674;-0.	[-0.649;-0.	[0.978;0.1
[-0.227;-0.	[0.429;-0.7	[0.191;-0.8	[0.176;-0.8	[0.73;-0.36	[0.843;-0.3	[0.429;-0.7	[0.656;-0.7	[-0.191;0.6
[0.782;-0.3	[0.61;-0.04	[-0.212;-0.	[-0.122;-0.	[-0.602;0.0	[-0.46;-0.1	[-0.596;0.0	[-0.383;-0.	[0.0782;0.
[-0.187;-0.	[-0.102;-0.	[0.0267;-0	[0.0205;-0	[-0.149;-0.	[-0.043;-0.	[-0.084;-0.	[-0.043;-0.	[-0.00795;
[-0.549;-0.	[-0.27;0.71	[0.958;-0.0	[0.977;-0.1	[0.216;0.9	[0.113;0.9	[0.259;0.8	[0.181;0.8	[0.992;0.0
[0.227;0.7	[0.766;0.4	[-0.191;-0.	[-0.176;-0.	[0.722;-0.0	[0.951;-0.0	[0.769;-0.0	[0.942;-0.0	[-0.0301;-0
[0.782;-0.3	[-0.571;0.3	[0.212;-0.3	[0.122;-0.4	[-0.605;0.3	[-0.256;0.2	[-0.57;0.41	[-0.269;0.3	[0.117;-0.4
[0.187;0.4	[0.122;0.3	[0.0267;0.	[0.0205;0.	[0.257;0.2	[0.133;0.1	[0.131;0.3	[0.0852;0.	[0.0276;0.
[0;0.707;0	[0;0.707;-0	[0;0.577;0.	[0;-0.707;0	[0;0.707;0.	[0;1;0;0]	[0;0;-1;0]	[0;0.707;-0	[0;-0.548;-
[0;0;1;0]	[0;0.707;0	[0;0;-1;0]	[0;0.707;-0	[0;0;1;0]	[0;0.577;0.	[0;0.577;0.	[0;-0.5;-0.5	[0;-0.434+
[0;0;-1;0]	[0;-0.707;0	[0;-0.577;-	[0;-0.5;-0.5	[0;1;0;0]	[0;0.577;0.	[0;-0.577;-	[0;-0.707;0	[0;-0.434-0
[0;0.707;0	[0;0.707;0	[0;0;1;0]	[0;0.5;0.5;	[0;-0.707;-	[0;1;0;0]	[0;0;1;0]	[0;0.5;0.5;	[0.577;-0.5
[0;-0.7796	[0;-0.8327	[0;0.68552	[0;-0.6441	[0;-0.8164	[0;0.57735	[0;0.61980	[0;-0.5773	[0;0.66778
[0.447213	[0.365148	[0.674199	[0.456171	[0;-0.8164	[0.087038	[0.322078	[0.282216	[0;0.66778
[0;-0.4472	[0;-0.8320	[0;2.18451	[0;-0.8277	[0.195740	[0;-0.9663	[0;-0.3226	[0;-0.9022	[0.779666
[0;0.66761	[0;0.65823	[0;-0.5664	[0;0.51949	[0;0.53452	[0;-0.4928	[0;-0.5214	[0;0.48236	[0;-0.4336
[0;0.66761	[0;0.71092	[0;-0.5664	[0;0.51949	[0;-0.4714	[0;-0.6123	[0;-0.6180	[0;-0.5773	[0;-0.3442
[0;0.44721	[0;-0.4472	[0;2.18451	[0;-0.8277	[0;-0.4714	[0;0.84023	[0;0.43864	[0;-0.6388	[0;-0.3442
[0.447213	[0.192450	[0.674199	[0.456171	[0;-0.9428	[0;0.84023	[0.522232	[0.223038	[0.953944
[0;0.77961	[0;0.88685	[0;-0.6855	[0;0.64414	[0.207598	[0.172132	[0;0.84906	[0;-0.8131	[0;0.66666

456	458	460	462	468	470	472	474	476
[0;0;1;0]	[0;0.577;-0	[0;-0.577;0	[0;-0.434+	[0;-0.707;0	[0;-0.707;0	[0;0;1;0]	[0;-0.656;-	[0;-0.707;0
[0;0;-1;0]	[0;0.577;-0	[0;0;1;0]	[0;-0.434-0	[0;0;1;0]	[0;-0.753;0	[0;0;-1;0]	[0;-0.656;-	[0;0;1;0]
[0;0;1;0]	[0.577;-0.5	[0;0;-1;0]	[0.707;-0.7	[0;0;-1;0]	[0.577;-0.5	[0;0;1;0]	[0.577;-0.5	[0;0;-1;0]
[0;0;-1;0]	[0;-0.577;-	[0;0.577;0.	[0;-0.548;-	[0;0.408;0.	[0;0.4;0.64	[0;0;-1;0]	[0;-0.414;-	[0;0.408;0.
[0;0;1;0]	[0;-0.771;-	[0;0;1;0]	[0;0.5774;	[0.8944;0;	[0.9097;0.	[0;0.7071;	[0;0.8547;	[0.2887;0.
[0;0;1;0]	[0.4082;-0	[0;0;1;0]	[0;0.5774;	[0;0.4082;	[0;-0.5774	[0;0.7071;	[0.3015;-0	[0;-0.3484
[0;0;1;0]	[0;0.3916-	[0;0;1;0]	[0;-0.5774	[0;0;1;0]	[0;-0.5774	[0;0;1;0]	[0;-0.5177	[0;0;1;0]
[0;0;1;0]	[0;0.3916+	[0;0.6667;	[0;0.5774;	[0;0.7071;	[0;-0.4082	[0;-0.7071	[0;-0.2682	[0;0.5154;
[0;0;1;0]	[0.7071;0;	[0;0;1;0]	[0.7071;0;	[0.8944;0;	[0.9313;-0	[0;0.7071;	[0.5774;0.	[0.5;0.5;-0
[0;0;-1;0]	[0;0.6559;	[0;0;1;0]	[0;0.3094-	[0;-0.7071	[0;-0.8629	[0;0.7071;	[0;0.6823;	[0;-0.7529
[0;0;1;0]	[0;0.6559;	[0;0;-1;0]	[0;0.3094+	[0;0;1;0]	[0;-0.7071	[0;0;1;0]	[0;0.6823;	[0;0;1;0]
[0;0;-1;0]	[0;0.414;0.	[0;0.6667;	[0;0.4623;	[0;0.4082;	[0;0.2811;	[0;0;-1;0]	[0;0.2043;	[0;-0.4004
[1;0;0;0]	[1;0;0;0]	[1;0;0;0]	[1;0;0;0]	[1;0;0;0]	[1;0;0;0]	[1;0;0;0]	[1;0;0;0]	[0;-0.577;0
[0;0;0.526	[0;1;0;0]	[0;0;-0.707	[0;0;-0.788	[0;0.628;-0	[0;0.844;-0	[0;0.628;-0	[0;0.844;-0	[1;0;0;0]
[0;1;0;0]	[0;0;-0.707	[0;1;0;0]	[0;1;0;0]	[0;-0.707;0	[0;-0.449;-	[0;-0.707;0	[0;-0.449;-	[0;-0.789;-
[0;0;-0.851	[0;0;0.707;	[0;0;0.707;	[0;0;0.615;	[0;0.325;0.	[0;0.293;0.	[0;0.325;0.	[0;0.293;0.	[0;0.211;0.
[-0.637;-0.	[-0.639;-0.	[-0.714;-0.	[0.792;0.4	[0.988;0.0	[0.992;0.0	[-0.689;-0.	[-0.676;-0.	[0.814;0.3
[-0.475;0.3	[0.705;-0.6	[-0.339;0.5	[0.585;-0.7	[-0.134;0.6	[0.117;-0.7	[0.645;-0.7	[0.689;-0.7	[0.548;-0.7
[0.465;-0.6	[-0.302;-0.	[0.595;-0.3	[0.147;0.3	[0.0794;0.	[-0.0432;-0	[-0.325;-0.	[-0.26;-0.3	[0.173;0.3;
[-0.391;-0.	[-0.0545;-0	[-0.143;-0.	[-0.098;-0.	[0;-0.707;0	[0.0252;-0	[0.0606;-0	[0.00135;-	[-0.0888;-0
[0.637;0.4	[0.756;0.5	[0.714;0.4	[0.921;0.2	[0.993;0.0	[0.996;0.0	[0.685;0.6	[-0.748;-0.	[0.907;0.3
[-0.475;0.3	[0.617;-0.7	[-0.339;0.5	[0.3;-0.837	[0;-0.707;0	[-0.0289;-0	[0.7;-0.706	[0.654;-0.7	[0.365;-0.8
[0.465;-0.6	[-0.0628;0	[0.595;-0.3	[-0.221;0.1	[0.114;-0.4	[0.0757;-0	[0.0476;0.	[0.00527;-	[-0.181;0.1
[0.391;0.4	[0.208;0.2	[0.143;0.6	[0.111;0.4	[0.0304;0.	[0.0221;0.	[0.198;0.3	[0.111;0.2	[0.111;0.4
[0;0;1;0]	[0;-0.577;-	[0;0;1;0]	[0;-0.548;-	[0;0;1;0]	[0;0.4;0.64	[0;0;1;0]	[0;-0.414;-	[0;0;1;0]
[0;0;-1;0]	[0.577;-0.5	[0;0.577;0.	[0;-0.434+	[0;0.408;0.	[0;-0.707;0	[0;0;-1;0]	[0;-0.656;-	[0;0.408;0.
[0;0;1;0]	[0;0.577;-0	[0;-0.577;0	[0;-0.434-0	[0;-0.707;0	[0;-0.753;0	[0;0;1;0]	[0;-0.656;-	[0;-0.707;0
[0;0;-1;0]	[0;0.577;-0	[0;0;-1;0]	[0.707;-0.7	[0;0;-1;0]	[0.577;-0.5	[0;0;-1;0]	[0.577;-0.5	[0;0;-1;0]
[0.478913	[0;-0.7060	[0;0.75293	[0;0.73594	[0;0.7071	[0;-0.8093	[0;0.22526	[0;0.70297	[0;-0.7659
[0;0.66787	[0;-0.7060	[0;-0.6359	[0;0.73594	[0.685994	[0;-0.8093	[0;0.22526	[0;0.70297	[0;-0.5126
[0;0.66787	[0.786146	[0.695313	[0.790183	[0;-0.2734	[0.839782	[0.345857	[0.757831	[0.285032
[0;0.34155	[0;-0.4963	[0;-0.4004	[0;-0.4396	[0;-0.3620	[0;0.37012	[0;0.37049	[0;0.39643	[0;0.36722
[0;-0.3415	[0;-0.3165	[0;-0.4004	[0;-0.3885	[0;-0.7071	[0;-0.6377	[0;-0.5584	[0;0.68618	[0;0.7071
[0;0.66787	[0;-0.3165	[0;0.63599	[0;-0.3693	[0;0.2357	[0;-0.6929	[0;-0.7607	[0;-0.9503	[0;0.58834
[0;0.66787	[0.203185	[0.695313	[0.748331	[0.632455	[0.962250	[0;-0.7607	[0.281480	[0;0.66666
[0.478913	[0;0.78737	[0;-0.7529	[0;0.77345	[0;0.57735	[0;0.54454	[0.522232	[0;-0.6861	[0.842281

478	856	858	862	904	906	908	910	922
[0;-0.707;	[0;0.707;-	[0;0;0.707	[0;0.408;0.	[0;1;0;0]	[0;1;0;0]	[0;0.707;0	[0;0.707;0	[0;0;0.707
[0;-0.753;	[0;-0.577;-	[0;0.465;0.	[0;-0.793;	[0;-1;0;0]	[0;-1;0;0]	[0;0;0.707	[0;-0.577;	[0;1;0;0]
[0.707;-0.7	[0;0.577;0.	[0.5;-0.5;-	[0.756;-0.	[0;-1;0;0]	[0;1;0;0]	[0;0;-0.707	[0;0.577;-	[0;1;0;0]
[0;0.4;0.64	[0;0.707;0.	[0;0.648;0.	[0;0.577;0.	[0;1;0;0]	[0;-1;0;0]	[0;0.707;0	[0;0.707;0	[0;0.816;0.
[0.3244;0.	[0.7559;0.	[0.8615;0.	[0.9258;0.	[0;1;0;0]	[0;1;0;0]	[0;0.7071;	[0.1925;0.	[0;1;0;0]
[0;-0.628;-	[0;0.7071;	[0;0.8018;	[0;-0.628;-	[0;1;0;0]	[0;1;0;0]	[0;0.7071;	[0;0.8507;	[0;1;0;0]
[0;-0.4082	[0;-0.5774	[0;-0.7071	[0;-0.7071	[0;1;0;0]	[0;-0.7071	[0;0.7071;	[0;-0.8165	[0;-0.8165
[0;-0.3251	[0;-0.7071	[0;0;0.707	[0;-0.3251	[0;1;0;0]	[0;-0.7071	[0;-0.7071	[0;-0.5257	[0;0;-0.707
[0.8528;0.	[0.8752;0.	[0.9313;0.	[0.957;0.0	[0;1;0;0]	[0;1;0;0]	[0;0.7071;	[0.378;0.7	[0;1;0;0]
[0;0.7071;	[0;0.7071;	[0;-0.5;0.7	[0;-0.7071	[0;-1;0;0]	[0;1;0;0]	[0;-0.7071	[0;0.8507;	[0;1;0;0]
[0;-0.7071	[0;-0.5774	[0;0;0.707	[0;0.5;0.5;	[0;-1;0;0]	[0;0.7071;	[0;0.7071;	[0;0;0.707	[0;0;0.707
[0;-0.2673	[0;0.7071;	[0;-0.5;-0.	[0;-0.5;-0.	[0;1;0;0]	[0;-0.7071	[0;0.7071;	[0;0.5257;	[0;0.4264;
[1;0;0;0]	[1;0;0;0]	[1;0;0;0]	[1;0;0;0]	[1;0;0;0]	[1;0;0;0]	[1;0;0;0]	[1;0;0;0]	[1;0;0;0]
[0;0.729;-	[0;-0.707;	[0;-0.707;	[0;0.707;-	[0;0;-0.707	[0;0.328;0.	[0;0;-0.851	[0;0.293;0.	[0;0.408;0.
[0;-0.663;-	[0;0;0;1]	[0;0;-0.707	[0;0.5;0.5;	[0;0;0.707	[0;0.737;-	[0;1;0;0]	[0;0.844;-	[0;0.707;-
[0;0.174;0.	[0;0.707;0.	[0;0.707;0.	[0;0.5;0.5;	[0;1;0;0]	[0;0.591;0.	[0;0;0.526	[0;0.449;0.	[0;0.577;0.
[-0.862;-0.	[-0.975;-0.	[0.988;0.0	[0.995;0.0	[-0.67;-0.3	[-0.696;-0.	[-0.727;-0.	[-0.812;-0.	[-0.712;-0.
[-0.482;0.7	[0.209;-0.5	[0.147;-0.6	[-0.101;0.6	[0;0;-0.707	[0.653;-0.7	[0.0425;0.	[-0.562;0.6	[0.659;-0.7
[-0.152;-0.	[-0.0719;-	[-0.0381;-	[0;0.707;-	[0.645;-0.7	[-0.296;-0.	[0.673;-0.4	[0.127;0.4	[-0.242;-0.
[-0.0338;-	[0;-0.707;	[-0.0257;0.	[-0.0274;-	[-0.368;-0.	[-0.0313;0.	[-0.129;-0.	[-0.0943;-	[0;0;-0.707
[0.973;0.1	[0.992;0.0	[0.997;0.0	[0.998;0.0	[0.67;0.38	[-0.722;-0.	[0.727;0.3	[0.898;0.2	[-0.73;-0.6
[0.195;-0.	[0;0.707;-	[0.00554;	[0;0.707;-	[0;0;0.707	[0.652;-0.7	[0.0425;0.	[0.346;-0.	[0.675;-0.7
[0.0926;-0.	[-0.125;0.2	[-0.08;0.35	[-0.0559;0	[0.645;-0.7	[-0.132;0.1	[0.673;-0.4	[-0.252;0.1	[0;0;0.707
[0.0766;0.	[0.0211;0.	[0.0206;0.	[0.0177;0.	[0.368;0.5	[0.19;0.39	[0.129;0.6	[0.102;0.4	[0.106;0.2
[0;-0.707;	[0;0.707;0.	[0;0.465;0.	[0;0.408;0.	[0;-1;0;0]	[0;1;0;0]	[0;0.707;0	[0;0.707;0	[0;1;0;0]
[0;-0.753;	[0;0.707;-	[0;0.648;0.	[0;-0.793;	[0;-1;0;0]	[0;1;0;0]	[0;0.707;0	[0;0.707;0	[0;0.816;0.
[0.707;-0.7	[0;-0.577;-	[0;0;0.707	[0.756;-0.	[0;1;0;0]	[0;-1;0;0]	[0;0;0.707	[0;-0.577;	[0;0;0.707
[0;0.4;0.64	[0;0.577;0.	[0.5;-0.5;-	[0;0.577;0.	[0;1;0;0]	[0;-1;0;0]	[0;0;-0.707	[0;0.577;-	[0;1;0;0]
[0;0.74671	[0;0.70710	[0;-1.4424	[0;0.45749	[0;0;0.707	[0;0.59499	[0;0.49859	[0;0.68034	[0;3.64799
[0;-0.8930	[0.632455	[0;0.27593	[0;-0.6074	[0.377964	[0.249688	[0.052925	[0.250627	[0.188982
[0.795226	[0;-0.4574	[0.758175	[0.962449	[0;1;0;0]	[0;-0.9712	[0;0.05488	[0;-0.5829	[0;-0.9773
[0;-0.3653	[0;0.70710	[0;-0.6859	[0;0.57735	[0;-1;1.110	[0;-0.8574	[0;-0.7105	[0;-0.6972	[0;-0.7345
[0;-0.6777	[0;0.70710	[0;5.20282	[0;0.43343	[0;1;0;0]	[0;0.78617	[0;-0.7105	[0;0.77151	[0;-1.3303
[0;-0.5139	[0;-0.6882	[0;-0.8382	[0;-0.7071	[0;-1;1.110	[0;0.78617	[0;-0.0548	[0;0.77459	[0;0.86602
[0.770491	[0;0.70710	[0.878054	[0.978106	[0;0;-0.707	[0;0.90909	[0.052925	[0;0.77459	[0;0.86602
[0;0.64012	[1.046728	[0;0.66529	[0;0.61117	[0.377964	[0.321860	[0;-0.4985	[0.250627	[0.288675

924	926	972	974	990	2184	2186	2190	2202
[0;-0.0804	[0;0;-0.707	[0;-0.707;0	[0;-0.707;0	[0;0.408;0.	[0;0;0;1]	[0;0;0;1]	[0;0;0;1]	[0;0;0.707;
[0;-0.0804	[0;-0.465;0	[0.687;-0.6	[0;0.465;-0	[0;-0.793;0	[0;1;0;0]	[0;1;0;0]	[0;0;0;-1]	[0;1;0;0]
[0.5;-0.5;0	[0.378;-0.7	[0;0;-0.707	[0.707;-0.7	[0.707;-0.7	[0;0;1;0]	[0;0;0;-1]	[0;0;0;-1]	[0;-1;0;0]
[0;-0.727;-	[0;0.648;0.	[0;0.707;0.	[0;0.648;0.	[0;0.577;0.	[0;-0.577;-	[0;0;0;1]	[0;0;0;1]	[0;0;0.707;
[0;0.8165;0	[0.2265;0.7	[0;0.5774;0	[0.3235;0.5	[0.5;0.5;0.5	[0;0;0;1]	[0;1;0;0]	[0;0.7071;0	[0;1;0;0]
[0;-0.8165	[0;0.8814;0	[0;0.5774;0	[0;-0.6479	[0;0.7071;0	[0;1;0;0]	[0;0;0;1]	[0;0;0.707	[0;0;0.707
[0;0;0.707	[0;-0.7071	[0;0;0.707	[0;-0.4653	[0;-0.7071	[0;0;1;0]	[0;0;0;1]	[0;0;0;1]	[0.5547;-0
[0;0;-0.707	[0;0.1328;0	[0;0.8165;-	[0;0.7071;-	[0;0;-0.707	[0.7559;-0	[0.6667;-0	[0;0;0;-1]	[0;0;-0.707
[0.5774;0.5	[0.7303;0.5	[0;0.5774;-	[0.6804;0.4	[0.866;0.28	[0;0;0;1]	[0;1;0;0]	[0;0.7071;0	[0;1;0;0]
[0;-0.6755	[0;-0.5774	[0;-0.5774	[0;-0.7071	[0;-0.8881	[0;1;0;0]	[0;0;0;1]	[0;0;0.707	[0;0;0.707
[0;-0.6755	[0;0.5774;0	[0;0;-0.707	[0;0.4653;-	[0;0;0.707	[0;0;1;0]	[0;0;0;-1]	[0;0;0;1]	[0.5547;0.
[0;0.5935;0	[0;-0.4082	[0;-0.8165	[0;0.6479;0	[0;-0.4597	[0.7559;0.	[0.4;0.2;0.	[0;0;0;-1]	[0;0;0.707
[1;0;0;0]	[1;0;0;0]	[1;0;0;0]	[1;0;0;0]	[1;0;0;0]	[0;0.408;0.	[1;0;0;0]	[1;0;0;0]	[1;0;0;0]
[0;0.5;-0.7	[0;0.707;-0	[0;0;-0.707	[0;0.349;0.	[0;0;0.707;	[0;0.707;-0	[0;0.707;-0	[0;0.707;-0	[0;0.888;-0
[0;-0.707;0	[0;0.5;0.5;-	[0;1;0;0]	[0;0.894;-0	[0;0.929;-0	[1;0;0;0]	[0;0.5;0.5;-	[0;0.577;0.	[0;0;0.707;
[0;0.5;0.70	[0;0.5;0.5;0	[0;0;0.707;	[0;0.28;0.5	[0;0.369;0.	[0;0.577;0.	[0;0.5;0.5;0	[0;0.408;0.	[0;0.46;0.6
[0.797;0.34	[-0.856;-0.	[-0.806;-0.	[0.878;0.2	[-0.921;-0.	[-0.532;-0.	[0.391;0.9	[-0.591;-0.	[0.369;0.9
[0.575;-0.6	[-0.496;0.7	[-0.587;0.5	[-0.471;0.6	[-0.381;0.7	[0;0.781;-0	[-0.408;0.2	[0;0.707;-0	[0.487;-0.2
[0.167;0.4	[-0.143;-0.	[0;0;0.707;	[-0.0103;0	[-0.0789;-0	[0;-0.239;-	[0.076;-0.0	[0.523;-0.4	[-0.792;0.2
[-0.0788;-0	[-0.0322;-0	[-0.0802;-0	[-0.0805;-0	[0;0;-0.707	[-0.847;0.3	[-0.821;0.2	[-0.614;0.1	[0;0;-0.707
[0.909;0.2	[0.962;0.2	[0.806;0.3	[0.952;0.1	[-0.985;-0.	[0.532;-0.4	[0.359;-0.9	[0.453;-0.6	[0.351;-0.9
[-0.369;0.8	[0.244;-0.9	[0.587;-0.5	[-0.295;0.7	[-0.164;0.8	[0;0.781;-0	[-0.0726;-0	[0;0.707;-0	[0;0;0.707;
[-0.161;-0.	[0.0938;-0	[0;0;0.707;	[-0.026;0.0	[0;0;0.707;	[0;0.239;0.	[0.588;0.2	[0.726;0.1	[0.852;0.3
[0.11;0.48	[0.0748;0.	[0.0802;0.	[0.0751;0.	[0.059;0.4	[0.847;0.3	[0.721;0.2	[0.518;0.3	[0.388;0.1
[0.5;-0.5;0	[0;0.648;0.	[0;-0.707;0	[0;-0.707;0	[0;0.577;0.	[0;0;1;0]	[0;0;0;1]	[0;0;0;1]	[0;1;0;0]
[0;-0.0804	[0;0;-0.707	[0;0;-0.707	[0;0.465;-0	[0;0.408;0.	[0;-0.577;-	[0;0;0;1]	[0;0;0;1]	[0;0;0.707;
[0;-0.0804	[0;-0.465;0	[0.687;-0.6	[0;0.648;0.	[0;-0.793;0	[0;1;0;0]	[0;1;0;0]	[0;0;0;-1]	[0;0;0.707;
[0;-0.727;-	[0.378;-0.7	[0;0.707;0.	[0.707;-0.7	[0.707;-0.7	[0;0;0;1]	[0;0;0;-1]	[0;0;0;-1]	[0;-1;0;0]
[0;-0.0874	[0;3.71081	[0;-0.7276	[0;0.65394	[0;2.06966	[0;0.40824	[0;-0.4082	[0;-0.4082	[0;-9.5424
[0;-0.0874	[0;-0.6204	[0;-1.1107	[0;0.65394	[0;-0.8739	[0;-0.7071	[0;-0.4082	[0;-0.7071	[0.316227
[0.620173	[0.422890	[0.251025	[0.830569	[0.851256	[0.495572	[2.417315	[7.554110	[0;-0.9670
[0;0.70481	[0;-0.6483	[0;-0.5773	[0;0.58341	[0;-0.5550	[0;0.57735	[0;0.40824	[0;0.30151	[0;-0.2545
[0;0.29770	[0;0.17722	[0;-0.5773	[0;-0.7040	[0;0.46499	[0;-0.5773	[0;0.70710	[0;0.17677	[0;6.11274
[0;0.29770	[0;-0.8203	[0;1.11075	[0;-0.4476	[0;-0.7634	[0;-0.4082	[0;0.70710	[0;0.17677	[0;0.81649
[0.609271	[0.324167	[0.251025	[0.800272	[0.816496	[0;-0.7071	[0;-0.8017	[0;-0.7071	[0.316227
[0;-0.7773	[0;0.78600	[0;0.72760	[0;0.73043	[0;0.68599	[0.495572	[0.478091	[0.478091	[0;0.57735

2204	2206	2252	2254	2270	2458	2462	2506	2510
[0;0;1;0]	[0;0;0.707	[0;0;0;1]	[0;0;0;1]	[0;0;0.707	[0;0.577;0.	[0;-0.5;-0.	[0;0.577;-0	[0;-0.434+
[0;0;-1;0]	[0;0.707;-0	[0;0;1;0]	[0;0;0;-1]	[0;0.577;-0	[0;1;0;0]	[0;-0.707;0	[0;0.577;-0	[0;-0.434-0
[0;0;1;0]	[0;-0.707;0	[0;0;-0.707	[0;0;0;1]	[0;-0.577;0	[0;-1;0;0]	[0.632;-0.	[0.5;-0.5;-	[0.577;-0.
[0;0;-1;0]	[0;0;0.707	[0;0;0.707	[0;0;0;-1]	[0;0;0.707	[0;0.577;0.	[0;0.5;0.5;	[0;-0.577;-	[0;-0.548;-
[0;0;1;0]	[0;0.5774;	[0;0;0;1]	[0;0;0;1]	[0;0;0.707	[0;1;0;0]	[0;-0.628;-	[0;-0.5774	[0;0.4004;
[0;0;1;0]	[0;0;0.850	[0;0;1;0]	[0;0;0;1]	[0;0;0.707	[0;-0.809;0	[0;-0.7071	[0.5;-0.5;-	[0.3162;-0.
[0;0;-0.707	[0.5;-0.5;-	[0;0.5774;-	[0;0;0;1]	[0;0;0.707	[0.4082;0;-	[0.3922;-0.	[0;-0.2887	[0;0.7071;-
[0.5774;-0.	[0;0;-0.525	[0;0.5774;-	[0;0;0;1]	[0;0;-0.707	[0;-0.309;-	[0;-0.3251	[0;-0.2887	[0;-0.7529
[0;0;1;0]	[0;0.5774;	[0;0;0;1]	[0;0;0;1]	[0;0;0.707	[0;1;0;0]	[0;-0.628;-	[0;0.5774;-	[0;-0.0692
[0;0;1;0]	[0;0;0.850	[0;0;1;0]	[0;0;0;-1]	[0;0.8165;-	[0;-0.809;0	[0;-0.7071	[0;0.5774;-	[0;-0.0692
[0;0;0.707	[0.5;0.5;-0	[0;0.5774;	[0;0;0;1]	[0;-0.8165	[0.7071;0;	[0.6325;0.	[0.5;-0.5;-	[0.7071;-0.
[0;0;-0.707	[0;0;0.525	[0;-0.5774	[0;0;0;-1]	[0;0;0.707	[0;0.309;0.	[0;0.3251;	[0;-0.5774	[0;0.6133;
[1;0;0;0]	[1;0;0;0]	[0;0;0.707	[1;0;0;0]	[1;0;0;0]	[1;0;0;0]	[1;0;0;0]	[1;0;0;0]	[1;0;0;0]
[0;0.888;-0	[0;0.888;-0	[1;0;0;0]	[0;0.591;-0	[0;0.953;-0	[0;0.707;-0	[0;0.707;-0	[0;0.408;0.	[0;-0.153;0
[0;0;0.707	[0;0.233;0.	[0;0.929;-0	[0;0.737;0.	[0;0;0.707	[0;0.325;0.	[0;0.408;0.	[0;0.707;-0	[0;0.902;-0
[0;0.46;0.6	[0;0.397;0.	[0;0.369;0.	[0;0.328;0.	[0;0.303;0.	[0;0.628;0.	[0;0.577;0.	[0;0.577;0.	[0;0.404;0.
[-0.589;-0.	[0.59;0.69	[-0.601;-0.	[-0.652;-0.	[-0.69;-0.5	[-0.603;-0.	[0.675;0.4	[0.637;0.4	[-0.685;-0.
[-0.0425;0	[0.283;-0.6	[0;0;0.707	[0.238;-0.6	[0.561;-0.8	[0.224;-0.6	[0;0.707;-0	[0;0.707;-0	[0.539;-0.0
[0.433;-0.3	[-0.752;0.3	[0;-0.816;0	[0.44;-0.55	[0.458;0.1	[-0.765;0.3	[0.737;-0.4	[0;-0.408;-	[-0.49;0.59
[-0.681;0.1	[-0.0838;-0	[-0.799;0.3	[-0.57;-0.1	[0;0;-0.707	[-0.0363;-0	[-0.0349;-0	[-0.771;0.3	[-0.0232;-0
[-0.391;0.6	[-0.444;0.8	[0.307;0.5	[0.311;-0.6	[0.74;-0.67	[-0.381;0.8	[-0.497;0.5	[0.92;-0.22	[0.754;0.0
[-0.0619;0	[0.0188;0.	[0;0;0.707	[-0.563;0.4	[0;0;0.707	[-0.012;0.4	[0;0.707;-0	[0;-0.0351	[-0.458;0.7
[-0.758;-0.	[-0.855;-0.	[0.806;-0.5	[0.669;0.4	[-0.638;-0.	[-0.883;-0.	[0.85;0.25	[0;0.816;-0	[-0.415;-0.
[0.518;0.3	[0.267;0.1	[-0.505;-0.	[0.373;0.4	[0.212;0.2	[0.273;0.2	[0.172;0.3	[0.392;0.5	[0.221;0.6
[0;0;1;0]	[0;-0.707;0	[0;0;0.707	[0;0;0;1]	[0;0;0.707	[0;-1;0;0]	[0;0.5;0.5;	[0;0.577;-0	[0;-0.548;-
[0;0;-1;0]	[0;0;0.707	[0;0;0;1]	[0;0;0;-1]	[0;0;0.707	[0;0.577;0.	[0;-0.5;-0.	[0;0.577;-0	[0;-0.434+
[0;0;1;0]	[0;0.707;-0	[0;0;1;0]	[0;0;0;1]	[0;0.577;-0	[0;0.577;0.	[0.632;-0.	[0;-0.577;-	[0;-0.434-0
[0;0;-1;0]	[0;0;0.707	[0;0;-0.707	[0;0;0;-1]	[0;-0.577;0	[0;1;0;0]	[0;-0.707;0	[0.5;-0.5;-	[0.577;-0.
[0;-0.0788	[0;-0.0638	[0;-0.6391	[0;-0.1571	[0;8.97336	[0;-0.5354	[0;-0.5314	[0;0.57735	[0;-0.6200
[0;-0.0788	[0.1023289	[0;-4.2561	[0;-0.1571	[0.040689	[0;-0.9347	[0;-0.7071	[0;0.57735	[0;-0.6200
[0.251896	[0;-0.7785	[0.246914	[0.136240	[0;-0.8439	[0.693375	[1.300707	[0.802955	[0.831933
[0;0.31553	[0;-0.2083	[0;-0.2880	[0;0.22056	[0;-0.1569	[0;0.61175	[0;0.56305	[0;0.57735	[0;0.50423
[0;0.65750	[0;1.16009	[0;0.43301	[0;0.34978	[0;3.59783	[0;0.68824	[0;-0.5998	[0;-0.2886	[0;0.26378
[0;0.44997	[0;0.94280	[0;0.43301	[0;0.34978	[0;-7.6911	[0;0.57735	[0;-0.7071	[0;-0.2886	[0;0.26378
[0;0.44997	[0;0.77151	[0;-2.5961	[0;-0.6417	[0;-7.6911	[0;0.21199	[0.104186	[0;0.57735	[0.117723
[0.424264	[1.820459	[0.280567	[0.459967	[0.357142	[0.363803	[0;0.55188	[0.188982	[0;0.68579

2524	2526	3038	4370	4374	4382	4418	4420	4422
[0;-0.707;	[0;-0.707;	[0;0.408;0.	[-0.5;-0.5;-	[0.447;0.4	[0.408;0.4	[0.5+8.33e	[-0.5;-0.5;	[0.377-0.3
[0;0;1;0]	[0;-0.753;	[0;-0.793;	[1;0;0;0]	[1;0;0;0]	[-0.211;0.7	[0.5-8.33e-	[1;0;0;0]	[0.377+0.3
[0;0;1;0]	[0.577;0;-	[0.756;-0.3	[0;1;0;0]	[-0.678;-0.	[0.789;-0.2	[1;0;0;0]	[0;0;1;0]	[1;0;0;0]
[0;0.408;0.	[0;0.4;0.64	[0;0.577;0.	[0.5;0.5;0.	[-0.447;-0.	[0.408;0.4	[-0.5;-0.5;-	[0.5;0.5;0.	[-0.481;-0.
[0;0;1;0]	[0;0.4152;	[0;-0.5774	[1;0;0;0]	[1;0;0;0]	[0.541;0.5	[1;0;0;0]	[1;0;0;0]	[1;0;0;0]
[0;0;1;0]	[0.127;-0.3	[0;-0.5774	[0;1;0;0]	[-0.644;0.5	[-0.2113;0.	[0.8605;-0.	[0;0;1;0]	[-0.6306;0.
[0;0;1;0]	[0;-0.6027	[0;-0.4082	[0.4798;0.	[0;-0.7071	[0.7887;-0.	[0.2871-0.	[-0.5;0.5;-	[0;0.7071;-
[0;0.7071;	[0;0.4743;	[0;-0.8066	[0.2527;0.	[-0.1996;-	[-0.2017;-	[0.2871+0.	[0.2236;0.	[-0.2265;-
[0;0;1;0]	[0.7071;-0.	[0.9177;-0.	[1;0;0;0]	[1;0;0;0]	[0.541;0.5	[1;0;0;0]	[1;0;0;0]	[1;0;0;0]
[0;-0.7071	[0;-0.6823	[0;0.4082;	[0;1;0;0]	[0.644;-0.5	[-0.2113;0.	[-0.3786+	[0;0;1;0]	[-0.3396-0
[0.7878;-0.	[0;-0.6823	[0;-0.4082	[0.4798;0.	[0;-0.7071	[0.7887;-0.	[-0.3786-0	[0.5;-0.5;-	[-0.3396+
[0;0.5774;	[0;0.3589;	[0;0.5774;	[-0.2527;-	[-0.1996;-	[0.2017;0.	[0.2984;0.	[0.2236;0.	[0.2217;0.
[1;0;0;0]	[1;0;0;0]	[1;0;0;0]	[0.408;0.4	[0.408;0.4	[0.408;0.4	[-0.707;0.7	[-0.707;0.7	[-0.707;0.7
[0;-0.5;0.7	[0;0.665;-	[0;0.58;0.2	[0.707;-0.7	[0.707;-0.7	[0.707;-0.7	[0;0;1;0]	[0;0;-0.707	[0;0;-0.851
[0;-0.707;	[0;0.584;0.	[0;0.575;-	[0;0;0;1]	[0;0;0;1]	[0.577;0.5	[0;0;0;1]	[0;0;0.707;	[0.707;0.7
[0;0.5;0.7	[0;0.466;0.	[0;0.577;0.	[0.577;0.5	[0.577;0.5	[0;0;0;1]	[0.707;0.7	[0.707;0.7	[0;0;0.526;
[0.695;0.4	[0.745;0.4	[-0.799;-0.	[0.61;0.61;	[0.903;0.2	[0.541;0.5	[0.814;0.2	[0.602;0.3	[0.897;0.2
[0.62;-0.11	[0.666;-0.5	[0.601;-0.4	[0.707;-0.7	[0.395;-0.6	[-0.0481;0.	[0.504;-0.6	[0.683;-0.1	[0.381;-0.5
[0.365;-0.5	[0.0425;0.	[0;-0.533;-	[-0.287;-0.	[0;0.707;-	[-0.815;0.3	[0.042;0.6	[-0.372;0.6	[-0.126;0.6
[0;-0.707;	[-0.017;-0.	[0;0.619;-	[-0.213;-0.	[-0.171;-0.	[-0.202;-0.	[-0.285;-0.	[-0.181;-0.	[-0.187;-0.
[-0.744;-0.	[-0.937;0.2	[0.993;-0.	[0.61;0.61;	[0.903;0.2	[0.541;0.5	[-0.923;0.	[0.602;0.3	[-0.948;-0.
[0.544;-0.6	[-0.305;-0.	[0;-0.0195	[0.707;-0.7	[0.395;-0.6	[-0.0481;0.	[0.112;0.7	[0.683;-0.1	[0.0987;0.
[-0.322;-0.	[-0.0748;-	[0;-0.816;	[-0.287;-0.	[0;0.707;-	[-0.815;0.3	[0.262;-0.4	[0.372;-0.6	[0.248;-0.7
[0.217;0.6	[0.152;0.4	[0.118;0.5	[0.213;0.2	[0.171;0.2	[0.202;0.2	[0.258;0.4	[0.181;0.6	[0.176;0.4
[0;0;1;0]	[0;0.4;0.64	[0;0.408;0.	[-0.5;-0.5;-	[0.447;0.4	[0.789;-0.2	[0.5-8.33e-	[1;0;0;0]	[-0.481;-0.
[0;0.408;0.	[0;-0.707;	[0;0.577;0.	[0.5;0.5;0.	[-0.678;-0.	[-0.211;0.7	[-0.5;-0.5;-	[0;0;1;0]	[0.377-0.3
[0;-0.707;	[0.577;0;-	[0;-0.793;	[0;1;0;0]	[-0.447;-0.	[0.408;0.4	[1;0;0;0]	[0.5;0.5;0.	[0.377+0.3
[0;0;1;0]	[0;-0.753;	[0.756;-0.3	[1;0;0;0]	[1;0;0;0]	[0.408;0.4	[0.5+8.33e	[-0.5;-0.5;	[1;0;0;0]
[0;0.70710	[0;-0.7975	[0;0.40824	[-0.5;-0.5;-	[-0.500000	[0.5;0.5;0.	[0.943119	[-0.5;-0.5;	[-0.528462
[0;-0.2557	[0;-0.7975	[0;-0.5795	[-0.925820	[-0.956182	[0.614086	[0.520751	[0.142764	[-0.528462
[0.188982	[0.830105	[0.956324	[0.150344	[0;-0.7071	[-0.072029	[0.520751	[0.142764	[0.959166
[0;-0.4608	[0;0.43880	[0;0.57735	[-0.554700	[0.524142	[-0.5;-0.5;-	[-0.590209	[0.5;0.5;0.	[-0.508977
[0;-0.7071	[0;0.69356	[0;0.40824	[0.554700	[-0.524142	[0.5;0.5;0.	[0.566946	[-0.5;-0.5;	[0.539359
[0;0.35355	[0;-0.4397	[0;-0.8065	[0.925820	[0.956182	[0.072029	[0.566946	[-0.142764	[0.301511
[0;0.35355	[0.159403	[0.5;-0.5;-	[0.150344	[0;-0.7071	[-0.614086	[0.923099	[-0.142764	[-0.943879
[0.449013	[0;0.39374	[0;0.57735	[0.5;0.5;0.	[0.500000	[-0.5;-0.5;-	[0.499999	[0.5;0.5;0.	[0.539359

4424	4426	4428	4430	4434	4436	4438	4440	4442
[-0.5;-0.5;	[0.377-0.3	[0.471;0.4	[-0.5;-0.5;	[0.548-2.2	[-0.577;-0.	[-0.577;-0.	[-0.577;-0.	[-0.577;-0.
[0;0;1;0]	[0.377+0.3	[0;0;1;0]	[-0.447;-0.	[0.548+2.2	[1;0;0;0]	[-0.602;-0.	[0;0;1;0]	[-0.602;-0.
[0;0;-1;0]	[0.707;0;-	[0;0;-1;0]	[0.707;0;-	[1;0;0;0]	[0;0;1;0]	[1;0;0;0]	[0;0;-1;0]	[0.707;0;-
[0.5;0.5;0.	[0.481;0.4	[-0.471;-0.	[-0.447;-0.	[0.383;0.3	[0.378;0.3	[0.372;0.3	[0.378;0.3	[-0.372;-0.
[0;0;1;0]	[0.4881;0.	[0;0;1;0]	[0.7343;0.	[1;0;0;0]	[1;0;0;0]	[1;0;0;0]	[-0.2689;-	[-0.5492;-
[-0.329;-0.	[-0.7071;0	[0.7153;0.	[-0.7071;0	[-0.4326;0	[0;0;1;0]	[-0.378;0.3	[0;0;1;0]	[-0.5774;0
[0;0.7071;	[0.4243;0.	[0.4;-0.751	[0;-0.7071	[-0.2774;-	[0.1012;-0	[0.08237;0	[0;0;1;0]	[0.2887;0.
[0.4582;0.	[0.3715;0.	[-0.2912;-	[0.2518;0.	[-0.245;-0.	[0.2272;0.	[-0.1938;-	[0.3498;0.	[-0.2525;-
[0;0;1;0]	[-0.1963+	[0;0;1;0]	[0.7071;0;	[1;0;0;0]	[1;0;0;0]	[1;0;0;0]	[-0.4896;-	[-0.5774+3
[-0.329;-0.	[-0.1963-0	[-0.7153;-	[-0.7071;0	[-0.2706+	[0;0;1;0]	[0.1665+0.	[0;0;1;0]	[-0.5774-3
[0;0.7071;	[-0.7071;0	[0.4;-0.751	[-0.5706;0	[-0.2706-0	[0.2783;-0	[0.1665-0.	[0;0;-1;0]	[-0.444;-0.
[-0.4582;-	[-0.3402;-	[-0.2912;-	[-0.243;-0.	[0.1982;0.	[-0.2037;-	[0.1752;0.	[-0.319;-0.	[-0.2299;-
[-0.707;0.	[-0.707;0.	[-0.707;0.	[-0.707;0.	[0.707;-0.	[-0.316;-0.	[0.707;-0.	[0.707;-0.	[0.707;-0.
[0;0;1;0]	[0;0;1;0]	[0;0;-0.851	[0;0;-0.92	[0.5;0.5;-0	[0.707;-0.	[-0.408;-0.	[0.5;0.5;-0	[0.5;0.5;-0
[0;0;0;1]	[0.707;0.7	[0.707;0.7	[0.707;0.7	[0;0;0;1]	[-0.447;-0.	[-0.408;-0.	[0;0;0;1]	[0;0;0;1]
[0.707;0.7	[0;0;0;1]	[0;0;0.526	[0;0;0.383	[0.5;0.5;0.	[0.447;0.4	[0.408;0.4	[0.5;0.5;0.	[0.5;0.5;0.
[-0.196;-0.	[0.465;0.5	[0.127;0.3	[0.601;0.4	[-0.926;-0.	[0.94;0.14	[0.948;0.1	[0.564;0.5	[-0.623;-0.
[0.797;-0.	[0.816;-0.4	[-0.855;-0.	[0.738;-0.2	[0.289;-0.	[-0.239;0.5	[0.272;-0.	[0.748;-0.	[0.716;-0.
[-0.311;0.	[0.0212;0.	[-0.413;0.	[-0.189;0.	[-0.148;-0.	[-0.144;0.	[-0.00424;	[-0.0409;-	[-0.197;-0.
[-0.479;-0.	[-0.344;-0.	[-0.287;-0.	[-0.242;-0.	[-0.191;-0.	[-0.196;-0.	[-0.165;-0.	[-0.349;-0.	[-0.244;-0.
[0.196;0.5	[-0.884;-0.	[0.127;0.3	[-0.905;-0.	[0.955;-0.	[-0.951;-0.	[0.966;0.0	[-0.733;-0.	[0.913;0.1
[-0.797;0.	[0.0398;0.	[-0.855;-0.	[0.141;0.5	[-0.022;-0.	[-0.0121;0	[0.00514;0	[-0.486;0.5	[0.0961;-0
[0.311;-0.	[0.322;-0.	[0.413;-0.	[0.325;-0.	[0.235;-0.1	[0.254;-0.	[0.211;-0.	[0.338;-0.	[0.318;-0.2
[0.479;0.3	[0.337;0.2	[0.287;0.4	[0.235;0.3	[0.182;0.2	[0.178;0.4	[0.15;0.33	[0.335;0.2	[0.238;0.2
[0;0;1;0]	[0.377-0.3	[-0.471;-0.	[0.707;0;-	[0.548-2.2	[0;0;1;0]	[-0.602;-0.	[0;0;-1;0]	[-0.577;-0.
[0;0;-1;0]	[0.377+0.3	[0;0;-1;0]	[-0.5;-0.5;	[0.548+2.2	[0.378;0.3	[0.372;0.3	[0.378;0.3	[-0.602;-0.
[0.5;0.5;0.	[0.707;0;-	[0.471;0.4	[-0.447;-0.	[0.383;0.3	[-0.577;-0.	[-0.577;-0.	[-0.577;-0.	[0.707;0;-
[-0.5;-0.5;	[0.481;0.4	[0;0;1;0]	[-0.447;-0.	[1;0;0;0]	[1;0;0;0]	[1;0;0;0]	[0;0;1;0]	[-0.372;-0.
[-0.5;-0.5;	[-0.33612	[-0.536691	[-0.54015	[0.527459	[-0.57735	[0.605012	[0.607896	[0.600985
[0.087705	[-0.33612	[0.049088	[-0.54015	[0.527459	[-0.843742	[0.605012	[0.063245	[0.600985
[0;0;1;0]	[0.774596	[-0.217207	[-0.762492	[-0.96336	[-0.843742	[0.971859	[3.291061	[-0.83666
[0.588348	[-0.58396	[-0.502682	[-0.50371	[-0.448901	[-0.41239	[-0.406587	[0.447374	[0.449266
[0.588348	[0.595978	[0.502682	[-0.53431	[0.589367	[0.625543	[-0.58026	[0.595625	[0.537595
[0;0;1;0]	[0.595978	[0.217207	[-0.196532	[0.707106	[-0.377964	[0.478373	[3.285631	[0.637945
[-0.08770	[0.718184	[-0.04908	[0.746830	[0.956523	[-0.377964	[0.965200	[-0.17616	[0.801783
[0.5;0.5;0.	[0.541858	[0.536691	[-0.558367	[-0.424264	[-0.95980	[0.452527	[0.422976	[0.444451

4444	4446	4546	4548	4550	4556	4558	4562	4564
[0.495;0.4	[-0.473;-0.	[-0.202+0.	[-0.378;-0.	[0.263-0.2	[0.408;0.4	[-0.322+0.	[-0.0533+	[-0.5;-0.5;
[0;0;1;0]	[-0.421;-0.	[-0.202-0.3	[0;0;1;0]	[0.263+0.2	[0;0;1;0]	[-0.322-0.2	[-0.0533-0	[0;0;1;0]
[0;0;-1;0]	[0.707;0;-0	[0.707;-0.7	[0;0;-1;0]	[-0.577;0.5	[0.642;-0.6	[0.707;-0.7	[0.707;-0.7	[0;0;-1;0]
[-0.38;-0.3	[-0.356;-0.	[-0.405;-0.	[0.378;0.3	[-0.407;-0.	[-0.408;-0.	[-0.395;-0.	[-0.322;-0.	[0.289;0.2
[0.4881;0.	[-0.8194;-	[-0.508;-0.	[0;0;1;0]	[-0.683;-0.	[0;0;1;0]	[0.3987;0.	[-0.5615;-	[0.7071;0;
[0;0;1;0]	[-0.459;0.3	[-0.7071;0	[0;0;1;0]	[-0.6938;0	[0;0;1;0]	[-0.7071;0	[-0.7071;0	[0.809;-0.5
[-0.182;0.4	[0.1029;0.	[-0.1114-0	[0;0;1;0]	[0.212+0.1	[0.7071;-0	[0.3028;0.	[-0.4082;-	[0;0;1;0]
[0.2453;0.	[-0.1984;-	[-0.1114+	[0.2981;0.	[0.212-0.1	[-0.3939;-	[0.337;0.3	[-0.3366;-	[0.309;0.5;
[-0.8797;-	[0.9347;0.	[0.7071;-0	[0;0;1;0]	[-0.464+0.	[0;0;1;0]	[-0.1903+	[0.7071;-0	[0.7071;0;
[0;0;1;0]	[-0.2339-0	[-0.3162+	[0;0;-1;0]	[-0.464-0.1	[0;0;1;0]	[-0.1903-0	[-0.3636+	[0.5721;-0.
[-0.2533;0	[-0.2339+	[-0.3162-0	[0;0;1;0]	[0.594;-0.7	[0.7071;-0	[0.7071;-0.	[-0.3636-0	[0;0;1;0]
[0.2349;0.	[0.1883;0.	[-0.3162;-	[0.2981;0.	[0.2707;0.	[-0.3939;-	[-0.3016;-	[-0.1828;-	[-0.2185;-
[0.707;-0.7	[0.707;-0.7	[-0.707;0.7	[-0.707;0.7	[-0.707;0.7	[-0.707;0.7	[-0.707;0.7	[0.707;-0.7	[0.707;-0.7
[-0.408;-0.	[-0.447;-0.	[0;0;0;1]	[0;0;0.526;	[0;0;-0.707	[0;0;-0.707	[0;0;-0.788	[0.577;0.5	[-0.302;-0.
[-0.408;-0.	[-0.447;-0.	[0;0;1;0]	[0.707;0.7	[0.707;0.7	[0.707;0.7	[0.707;0.7	[0;0;0;1]	[-0.523;-0.
[0.408;0.4	[0.316;0.3	[0.707;0.7	[0;0;-0.851	[0;0;0.707;	[0;0;0.707;	[0;0;0.615;	[0.408;0.4	[0.368;0.3
[0.651;0.4	[0.8;0.382;	[0.535;0.5	[-0.588;-0.	[0.698;0.4	[0.501;0.5	[0.562;0.5	[-0.585;-0.	[0.709;0.4
[0.695;-0.3	[0.566;-0.6	[0.707;-0.7	[-0.74;0.60	[0.667;-0.6	[0.282;0.2	[0.707;-0.7	[0.707;-0.7	[0.663;-0.6
[-0.177;0.7	[-0.00233;	[-0.448;-0.	[-0.208;-0.	[0.141;0.3	[0.707;-0.7	[-0.286;-0.	[-0.385;-0.	[0.109;0.3
[-0.248;-0.	[-0.198;-0.	[-0.116;-0.	[-0.252;-0.	[-0.218;-0.	[-0.412;-0.	[-0.32;-0.3	[-0.0997;-	[-0.214;-0.
[-0.905;-0.	[0.932;0.1	[0.612;0.6	[0.588;0.4	[0.873;0.3	[0.501;0.5	[0.621;0.6	[-0.661;-0.	[0.843;0.3
[0.063;0.4	[0.00321;0	[0.707;-0.7	[0.74;-0.60	[0.434;-0.8	[-0.282;-0.	[0.707;-0.7	[0.707;-0.7	[0.471;-0.8
[0.351;-0.7	[0.31;-0.57	[0.159;0.1	[0.208;0.0	[-0.0483;0	[0.707;-0.7	[-0.124;-0.	[-0.137;-0.	[0.122;-0.1
[0.234;0.3	[0.187;0.2	[0.316;0.3	[0.252;0.6	[0.216;0.4	[0.412;0.4	[0.314;0.3	[0.211;0.2	[0.229;0.4
[0.495;0.4	[-0.473;-0.	[0.707;-0.7	[0;0;1;0]	[0.263-0.2	[0;0;1;0]	[-0.322+0.	[-0.322;-0.	[0;0;-1;0]
[0;0;-1;0]	[-0.421;-0.	[-0.405;-0.	[0;0;-1;0]	[0.263+0.2	[0.408;0.4	[-0.322-0.2	[-0.0533+	[0.289;0.2
[-0.38;-0.3	[0.707;0;-0	[-0.202+0.	[0.378;0.3	[-0.577;0.5	[0.642;-0.6	[0.707;-0.7	[-0.0533-0	[-0.5;-0.5;
[0;0;1;0]	[-0.356;-0.	[-0.202-0.3	[-0.378;-0.	[-0.407;-0.	[-0.408;-0.	[-0.395;-0.	[0.707;-0.7	[0;0;1;0]
[-0.561011	[-0.572752	[-0.267261	[0.437736	[-0.29880	[0.5;0.499	[-0.356473	[0.316946	[0.574740
[-0.07591	[-0.580141	[-0.267261	[-0.403034	[-0.29880	[-0.404113	[-0.356473	[0.316946	[0.429805
[-0.145453	[0.838884	[0.707106	[0.331862	[-0.519655	[0.785772	[0.707106	[-0.707106	[-0.278148
[-0.41763	[-0.407841	[0.457495	[-0.389541	[-0.436819	[-0.392232	[-0.409918	[0.378175	[-0.345967
[0.570605	[-0.535755	[-0.196287	[0.389541	[-0.32136	[0.392232	[0.427034	[0.625673	[0.603703
[-0.249298	[0.318532	[-0.196287	[-0.331862	[-0.32136	[0.404113	[0.162533	[0.625673	[-0.288675
[-0.139177	[-0.825246	[-0.707106	[0.403034	[-0.847318	[-0.785772	[-0.707106	[-0.707106	[-0.377964
[0.451301	[-0.461911	[0.462020	[-0.437736	[0.500406	[-0.5;-0.49	[0.522567	[0.395808	[-0.377964

4566	4572	4574	4678	4682	4686	4692	4694	4698
[-0.224+0.4	[0.49;0.49;	[0.5;0.5;-0	[0;0.707;-0	[0.707;0;0;	[0.707;0;0;	[0.602;0.07	[0;0.707;-0	[-0.372;0.3
[-0.224-0.4	[0;0;1;0]	[0.5;0.5;-0	[1;0;0;0]	[0.707;0;0;	[0;-0.707;0	[0.602;0.07	[-0.602;-0.	[-0.602;-0.
[0.577;-0.5	[0.444;-0.4	[-0.707;0.7	[-1;0;0;0]	[0.707;0;0;	[0.707;0;0;	[1;0;0;0]	[1;0;0;0]	[0.602;-0.6
[-0.322;-0.	[-0.319;-0.	[-0.316;-0.	[0.632;0.3	[-0.707;0;0	[-0.707;0;0	[-0.298;-0.	[0.372;0.37	[0.372;0.37
[-0.8071;-0	[0.2465;0.2	[0.441;0.44	[1;0;0;0]	[-0.8507;0	[-0.9239;0	[1;0;0;0]	[1;0;0;0]	[-0.6015;-0
[-0.6751;0	[0.5846;-0	[-0.7071;0	[-0.6325;0	[0.8507;0;0	[-0.8165;0	[0.5227;-0	[0.5;-0.5;-0	[0.6768;-0.
[-0.2116;-0	[0;0;1;0]	[-0.2198;-0	[-0.4472;0	[0.5257;0;0	[0;-0.7071	[0.2293-0.0	[0.189;0.50	[-0.3717;-0
[0.2467;0.3	[-0.3238;-0	[0.2488;0.2	[0;-0.7071	[-0.5257;0	[0.3827;0;0	[0.2293+0.	[-0.1015;0	[-0.2049;0
[-0.6559;0	[-0.425;-0.	[0.6126;0.1	[1;0;0;0]	[-0.8507;0	[-0.9239;0	[1;0;0;0]	[1;0;0;0]	[-0.6015;0.
[-0.6559;0	[0.6841;-0	[0.4082-1.1	[0;0.7071;-	[0.8507;0;0	[0;-0.7071	[-0.3797+0	[0.2199;-0	[0.6768;0.0
[0;-0.7071	[0;0;1;0]	[0.4082+1.	[0.4472;0;0	[0.5257;0;0	[-0.4264;0	[-0.3797-0	[0.3333;-0	[0.3717;-0.
[-0.1861;-0	[-0.2832;-0	[0.2132;0.2	[0.2626;0.3	[-0.5257;0	[0.3827;0;0	[-0.1236;-0	[-0.1519;-0	[0.2049;0.2
[0.707;-0.7	[-0.316;-0.	[0.707;-0.7	[0;0.577;0.	[0;0.851;0;	[0;0.628;0.	[-0.577;0;0	[-0.5;0.5;0	[-0.851;0;0
[-0.461;-0.	[0.707;-0.7	[-0.395;-0.	[0;0.707;-0	[0;0;-1;0]	[0;0.707;-0	[0;-1;0;0]	[0.653;0.65	[0;0.851;0;
[-0.408;-0.	[0.577;0.57	[-0.545;-0.	[1;0;0;0]	[1;0;0;0]	[1;0;0;0]	[-0.707;0;0	[-0.5;0.5;-0	[0;0.526;0;
[0.347;0.34	[0.258;0.25	[0.217;0.21	[0;0.408;0.	[0;0.526;0;	[0;0.325;0.	[0.408;0;0.	[0.271;0.27	[-0.526;0;-
[-0.787;-0.	[0.565;0.56	[0.598;0.59	[-0.885;-0.	[-0.547;-0.	[0.755;0.38	[-0.902;-0.	[-0.933;-0.	[-0.602;-0.
[0.571;-0.7	[0.707;-0.7	[0.707;-0.7	[-0.387;0.6	[0.646;-0.6	[0.563;-0.5	[0.33;-0.65	[0.298;-0.7	[0.677;-0.6
[-0.188;-0.	[-0.281;-0.	[-0.303;-0.	[-0.257;-0.	[-0.437;-0.	[0;0.707;-0	[-0.277;-0.	[-0.155;-0.	[-0.372;-0.
[-0.143;-0.	[-0.318;-0.	[-0.225;-0.	[0;-0.707;0	[-0.303;0.3	[-0.336;-0.	[-0.0324;0	[-0.132;0.2	[-0.205;0.2
[-0.946;-0.	[0.62;0.62;	[0.665;0.66	[-0.928;-0.	[0.547;-0.5	[-0.909;-0.	[-0.932;-0.	[-0.958;-0.	[-0.602;0.6
[-0.272;0.9	[0.707;-0.7	[0.707;-0.7	[0;0.707;-0	[-0.646;-0.	[0;0.707;-0	[-0.0634;0	[0.0133;0.8	[0.677;0.67
[-0.00533;	[0.123;0.12	[0.0227;0.0	[0.359;-0.3	[0.437;-0.4	[0.335;-0.5	[0.336;-0.4	[0.264;-0.4	[0.372;-0.3
[0.176;0.30	[0.318;0.31	[0.24;0.24;	[0.094;0.58	[0.303;0.32	[0.248;0.37	[0.122;0.50	[0.114;0.35	[0.205;0.20
[-0.224-0.4	[0.49;0.49;	[0.5;0.5;-0	[1;0;0;0]	[-0.707;0;0	[0.707;0;0;	[1;0;0;0]	[1;0;0;0]	[-0.372;0.3
[0.577;-0.5	[0.444;-0.4	[-0.707;0.7	[-1;0;0;0]	[0.707;0;0;	[0;-0.707;0	[-0.298;-0.	[-0.602;-0.	[0.372;0.37
[-0.322;-0.	[-0.319;-0.	[0.5;0.5;-0	[0.632;0.3	[0.707;0;0;	[0.707;0;0;	[0.602;0.07	[0.372;0.37	[-0.602;-0.
[-0.224+0.4	[0;0;1;0]	[-0.316;-0.	[0;0.707;-0	[0.707;0;0;	[-0.707;0;0	[0.602;0.07	[0;0.707;-0	[0.602;-0.6
[-0.539628	[0.562561	[0.598687	[-1.150973	[0.650442	[-2.791275	[0.654130	[-0.281439	[0.438774
[-0.539628	[-0.045760	[0.598687	[-0.942809	[0.650442	[0.834314	[0.654130	[-0.638209	[0.651484
[-0.606360	[-0.045760	[0.707106	[1;0;0;0]	[0.783671	[-0.877154	[0.945586	[-0.964053	[-0.683723
[-0.359521	[-0.341744	[0.342603	[-0.439941	[0.576605	[-0.516397	[0.358437	[-0.362145	[-0.406762
[-0.617091	[0.550412	[-0.559357	[-4.114338	[-0.576605	[0.734803	[-0.346410	[0.317180	[0.406762
[-0.614654	[0.777737	[0.306273	[1;0;0;0]	[0.783671	[5.582550	[-0.346410	[-0.619948	[-0.683723
[0.864594	[-0.482886	[0.707106	[0.925820	[-0.650442	[-0.730654	[0.936632	[-0.952921	[0.651484
[0.427253	[-0.397647	[0.444391	[0.925820	[-0.650442	[-0.730654	[0.208514	[0.449300	[-0.438774

4700	4702	4740	4742	4748	4750	4758	4764	4766
[0.295-0.3	[-0.254;0.5	[-0.5;-0.5;	[-0.376;-0.	[-0.526;-0.	[0.5;0.5;-0	[-0.602;-0.	[-0.707;0;	[0.707;0;0;
[0.295+0.3	[0;0.707;-	[-0.5;0.5+7	[-0.12-0.4	[-0.413+7.	[0.366+0.2	[-0.5-5.55	[-0.395+0.	[-0.402-0.1
[0.717;-0.5	[0.799;-0.4	[-0.5;0.5-7	[-0.12+0.4	[-0.413-7.4	[0.366-0.2	[-0.5+5.55	[-0.395-0.	[-0.402+0.
[-0.353;-0.	[0.379;0.3	[0.5;0.5;0.	[0.526;0.3	[0.526;0.3	[0.511;0.2	[0.372;0.3	[-0.37;-0.4	[-0.366;-0.
[-0.7408;-	[-0.8909;-	[-0.5;-0.5;-	[-0.637;-0.	[-0.3213;-	[-0.3948;-	[0.6015;0.	[-0.2589;-	[0.3025;0.
[-0.5774;0	[-0.6193;0	[-0.5-2.22	[0.06984-	[-0.2976+	[0;-0.7071	[-0.6606;0	[-0.2011+	[-0.5018;0
[-0.2597+	[-0.0959;-	[-0.5+2.22	[0.06984+	[-0.2976-0	[0;0.7071;	[-0.3717;-	[-0.2011-0	[0.2132;0.
[-0.2597-0	[-0.2068;0	[-0.5;-0.5;	[0;-0.7071	[-0.6323;-	[-0.5281;-	[-0.2523;0	[0.5299;-0	[-0.3314;0
[-0.7988;0	[-0.9264;0	[-0.5;-0.5;	[-0.5251;-	[-0.3213;-	[0.3065;0.	[-0.6768;-	[0.5774;0.	[0.4082;0.
[-0.5774;0	[0.1715;-0	[-0.5-2.22	[0.1533-0.	[-0.2976+	[-0.1683+	[-0.25+0.4	[-0.2631+	[0.5895;-0
[0.3786;-0	[-0.3268;0	[-0.5+2.22	[0.1533+0.	[-0.2976-0	[-0.1683-0	[-0.25-0.4	[-0.2631-0	[0.5895;-0
[0.2544;0.	[-0.2064;-	[0.5;0.5;0.	[-0.402;-0.	[-0.6323;-	[0.4509;0.	[-0.2049;-	[-0.3763;-	[-0.2611;-
[-0.737;0;	[-0.655;0.4	[1;0;0;0]	[0;0.851;0	[0;0;-0.851	[0;0.628;0.	[-0.851;0;	[-0.737;0;	[-0.655;0.4
[0;-1;0;0]	[0.498;0.8	[0;1;0;0]	[0;0;-1;0]	[0;1;0;0]	[0;0.707;-	[0;0.851;0	[0;-1;0;0]	[0.498;0.8
[-0.591;0;-	[-0.542;0.3	[0;0;1;0]	[1;0;0;0]	[1;0;0;0]	[1;0;0;0]	[0;0.526;0	[-0.591;0;-	[-0.542;0.3
[0.328;0;0.	[0.168;0.2	[0;0;0;1]	[0;0.526;0	[0;0;0.526	[0;0.325;0.	[-0.526;0;-	[0.328;0;0.	[0.168;0.2
[-0.74;-0.4	[-0.826;-0.	[0.5;0.5;0.	[-0.606;-0.	[-0.458;-0.	[-0.463;-0.	[-0.602;-0.	[0.429;0.6	[0.414;0.7
[0.577;-0.5	[0.498;-0.7	[0;0;-0.707	[0.637;-0.7	[0.542;-0.2	[-0.644;0.6	[0.7;-0.7;-	[0.685;-0.6	[0.744;-0.5
[0.0149;-0	[-0.14;-0.5	[0.707;-0.7	[-0.464;-0.	[0.252;-0.7	[0.382;0.2	[-0.372;-0.	[0.355;0.3	[-0.458;-0.
[0.344;0.1	[-0.222;0.0	[-0.5;-0.5;	[-0.111;0.1	[-0.659;-0.	[-0.475;-0.	[-0.0981;0	[-0.471;-0.	[-0.257;0.0
[-0.912;-0.	[-0.939;-0.	[0.5;0.5;-0	[0.58;0.68	[0.458;0.5	[0.371;0.8	[-0.677;-0.	[0.396;0.8	[-0.283;-0.
[-0.0298;0	[0.0439;0.	[0;0;-0.707	[0.747;-0.6	[0.542;-0.2	[-0.801;0.4	[0.694;-0.6	[0.786;-0.4	[-0.887;0.2
[0.315;-0.6	[0.284;-0.4	[0.707;-0.7	[0.157;-0.1	[0.252;-0.7	[-0.157;0.3	[-0.134;0.1	[-0.115;0.3	[-0.202;0.2
[0.262;0.3	[0.189;0.2	[0.5;0.5;0.	[0.285;0.3	[0.659;0.1	[0.443;0.1	[0.205;0.2	[0.461;0.1	[0.303;0.1
[0.295-0.3	[-0.254;0.5	[-0.5;-0.5;	[-0.12+0.4	[-0.526;-0.	[0.366-0.2	[0.372;0.3	[-0.37;-0.4	[-0.366;-0.
[0.717;-0.5	[0.379;0.3	[-0.5;0.5+7	[0.526;0.3	[0.526;0.3	[0.5;0.5;-0	[-0.5-5.55	[-0.707;0;	[0.707;0;0;
[0.295+0.3	[0.799;-0.4	[-0.5;0.5-7	[-0.12-0.4	[-0.413+7.	[0.366+0.2	[-0.5+5.55	[-0.395+0.	[-0.402-0.1
[-0.353;-0.	[0;0.707;-	[0.5;0.5;0.	[-0.376;-0.	[-0.413-7.4	[0.511;0.2	[-0.602;-0.	[-0.395-0.	[-0.402+0.
[0.554504	[-0.050835	[-0.5;-0.5;	[-0.438293	[0.604466	[0.523391	[-0.651484	[-0.79757	[-0.819103
[0.554504	[-0.050835	[-0.5;0.5+4	[0.608406	[-0.347105	[0.378777	[0.5;-0.5-4	[-0.280744	[0.349660
[-0.824253	[0.904203	[-0.5;0.5-4	[0.608406	[-0.347105	[0.378777	[0.5;-0.5+4	[-0.280744	[0.349660
[-0.409064	[0.388407	[0.5;0.5;0.	[-0.536339	[0.477449	[0.469718	[-0.406762	[-0.398519	[0.380536
[0.365621	[-0.428116	[-0.5;-0.5;	[-0.410789	[-0.477449	[0.543285	[0.572860	[-0.734040	[0.622983
[0.365621	[0.223448	[0.5;-0.5+1	[-0.410789	[-0.347105	[-0.105487	[0.681552	[0.452058	[0.116331
[-0.870343	[-0.912870	[0.5;-0.5-1	[-0.148991	[-0.347105	[-0.105487	[-0.683723	[0.452058	[0.116331
[-0.313496	[-0.520230	[0.5;0.5;0.	[-0.608435	[0.604466	[0.671453	[0.438774	[-0.423338	[0.478535

4812	4814	4830	4946	4950	4952	4954	4958	4994
[0.408;0.4	[0;0.707;-	[-0.639;-0.	[-0.577;0;-	[-0.378;-0.	[0;-0.707;	[0.562;0.3	[-0.506;-0.	[0.5;0.25+
[-0.707;0.7	[0.447;-0.7	[0.0802;-0	[0.773;0.2	[-0.493;0.1	[-0.632;-0.	[-0.577;-0.	[0;-0.707;	[0.5;0.25-
[0.707;-0.7	[0.707;-0.7	[0.707;-0.7	[1;0;0;0]	[1;0;0;0]	[0;0.707;0	[-0.634;0.5	[-0.815;0.3	[0;1;0;0]
[-0.408;-0.	[0.447;0.2	[-0.316;-0.	[0.24;0.62	[0.277;0.5	[0;0.707;0	[-0.264;-0.	[-0.282;-0.	[-0.378;-0.
[0.4082;0.	[0.5426;0.	[-0.5;-0.5;-	[1;0;0;0]	[1;0;0;0]	[-0.5615;-	[0.7701;0.	[0.8925;0.	[-0.3589;-
[0.7071;-0	[-0.7071;0	[-0.7071;0	[-0.2582;0	[-0.3408;0	[0;-0.7071	[-0.2944;0	[0.4076;-0	[0;1;0;0]
[-0.7071;0	[0.01855-	[-0.3162;-	[-0.2294;-	[0;0.7071;	[0;0.7071;	[0.2028;0.	[0;0.7071;	[0.3484-0.
[-0.4082;-	[0.01855+	[-0.2236;0	[-0.1741;0	[-0.1845;-	[-0.3366;-	[0.2327;0.	[-0.1934;-	[0.3484+0.
[0.4082;0.	[-0.3172;-	[0.6165;0.	[1;0;0;0]	[1;0;0;0]	[-0.9015;-	[0.9351;0.	[-0.9585;-	[0;1;0;0]
[-0.7071;0	[0.7071;-0	[0.7071;-0	[0.3007;0.	[0;-0.7071	[0;-0.7071	[-0.2695;-	[0;0.7071;	[-0.5878;-
[-0.7071;0	[0.6821;-0	[0.5;-0.5;-	[-0.3333;0	[0.2638;-0	[0;-0.7071	[-0.2478;0	[-0.2358;0	[-0.5878;-
[0.4082;0.	[-0.3555;-	[0.1911;0.	[0.1126;0.	[-0.1295;-	[0.1156;0.	[-0.1746;-	[0.1603;0.	[0.3349;0.
[0;0;-0.707	[0;0.577;0.	[-0.5;0.5;0	[-0.577;0.5	[-0.756;0.3	[0.888;-0.3	[-0.806;0.4	[0.815;-0.3	[-0.577;0.5
[0;1;0;0]	[1;0;0;0]	[0.688;0.6	[0.657;0.2	[0;0.707;-	[0;0.707;-	[-0.335;-0.	[0;0.707;-	[0;0;-1;0]
[1;0;0;0]	[0;0.789;-	[-0.5;0.5;-	[-0.228;0.4	[-0.562;-0.	[0;0;0;1]	[-0.273;0.1	[0.506;0.3	[-0.707;0;
[0;0;0.707	[0;0.211;0.	[0.162;0.1	[0.429;0.6	[0.337;0.5	[0.46;0.62	[0.405;0.6	[0.282;0.5	[0.408;0.8
[-0.54;-0.5	[0.588;0.5	[-0.602;-0.	[-0.947;-0.	[-0.959;-0.	[0.787;0.2	[-0.838;-0.	[0.892;0.2	[-0.604;-0.
[-0.457;0.4	[0.65;-0.67	[0.691;-0.6	[-0.233;0.6	[0.233;-0.6	[0.512;-0.6	[0.474;-0.7	[0.408;-0.6	[0.65;-0.74
[-0.54;0.54	[0.127;-0.3	[-0.372;-0.	[-0.177;-0.	[0;0.707;-	[0;0.707;-	[-0.152;-0.	[0;0.707;-	[0.457;0.5
[-0.457;-0.	[-0.464;-0.	[-0.152;0.1	[-0.129;0.1	[-0.159;-0.	[-0.344;-0.	[-0.222;-0.	[-0.193;-0.	[-0.0661;0
[0.54;0.54	[0.601;0.6	[0.667;0.6	[-0.958;0.	[-0.972;-0.	[-0.914;-0.	[-0.939;0.	[-0.958;-0.	[0.565;0.6
[0.457;-0.4	[0.602;-0.5	[0.632;-0.6	[0.0696;0.	[0;0.707;-	[0;0.707;-	[0.0711;0.	[0;0.707;-	[0.76;-0.64
[0.54;-0.54	[0.42;-0.3	[-0.316;0.3	[0.249;-0.3	[0.205;-0.5	[0.31;-0.52	[0.275;-0.4	[0.236;-0.5	[0.122;0.0
[0.457;0.4	[0.317;0.3	[0.234;0.2	[0.122;0.4	[0.114;0.4	[0.263;0.4	[0.193;0.3	[0.16;0.38	[0.297;0.4
[0.408;0.4	[0.447;-0.7	[-0.316;-0.	[1;0;0;0]	[1;0;0;0]	[-0.632;-0.	[0.562;0.3	[0;-0.707;	[0.5;0.25-
[0.707;-0.7	[0.707;-0.7	[0.707;-0.7	[-0.577;0;-	[0.277;0.5	[0;-0.707;	[-0.577;-0.	[-0.815;0.3	[-0.378;-0.
[-0.408;-0.	[0.447;0.2	[0.0802;-0	[0.773;0.2	[-0.493;0.1	[0;0.707;0	[-0.264;-0.	[-0.282;-0.	[0;1;0;0]
[-0.707;0.7	[0;0.707;-	[-0.639;-0.	[0.24;0.62	[-0.378;-0.	[0;0.707;0	[-0.634;0.5	[-0.506;-0.	[0.5;0.25+
[0.5;0.5;-0	[-0.373033	[-0.499999	[-0.680089	[-0.632455	[-4.351097	[-0.642968	[-0.597295	[0.600492
[-0.670820	[-0.373033	[-0.615644	[-0.680089	[0.464056	[0.752223	[-0.642968	[4.014029	[0.600492
[0.707106	[0.663631	[0.685994	[-0.964816	[-0.975417	[-0.333653	[0.826323	[-0.958246	[-0.096448
[0.316227	[0.352787	[0.308192	[0.311724	[-0.308606	[-0.288675	[0.331926	[-0.322491	[-0.446042
[-0.316227	[-1.957074	[-0.5;0.499	[0.559417	[-0.649578	[-0.662306	[0.565719	[-0.581757	[0.227750
[0.707106	[0.155963	[-0.5;-0.5;	[0.755063	[-6.513308	[3.745640	[0.599029	[-3.659011	[0.227750
[0.670820	[0.707881	[-0.697485	[0.961416	[0.968962	[-0.081553	[0.733799	[-0.941774	[-0.195646
[0.5;0.5;0.	[-0.683677	[-0.441726	[0.161726	[0.293230	[-0.081553	[-0.207973	[0.324069	[-0.326558

4998	5002	5004	5006	5010	5012	5014	5016	5018
[-0.378;-0.	[0.272-0.2	[0.414;0.5	[0.416;0.6	[0.602;0.2	[-0.577;-0.	[-0.577;-0.	[-0.577;-0.	[-0.577;-0.
[-0.144+0.	[0.272+0.2	[6.18e-17+	[-0.247-0.	[0.602;0.2	[0.249+0.4	[0.109+0.5	[0;1;0;0]	[-0.417;-0.
[-0.144-0.	[0;1;0;0]	[6.18e-17-	[-0.247+0.	[0;1;0;0]	[0.249-0.4	[0.109-0.5	[0;-1;0;0]	[0;1;0;0]
[0.417;0.6	[-0.416;-0.	[-0.414;-0.	[0.406;0.5	[0.298;0.6	[0.307;0.6	[0.314;0.5	[0.258;0.7	[0.313;0.6
[0.6573;0.	[0;-1;0;0]	[-0.2621;-	[0.4029;0.	[0.2897;0.	[0.5615;0.	[0.7142;0.	[0;1;0;0]	[0;1;0;0]
[-0.2214+	[0;1;0;0]	[-0.4048-0	[0.1128-0.	[0;1;0;0]	[-0.5+3.34	[-0.4447;0	[-0.1118;0	[0.1502;-0.
[-0.2214-0	[0.5;0.5;-0	[-0.4048+	[0.1128+0.	[-0.4033;-	[-0.5-3.342	[0;0.7071;	[0;-0.7071	[0.3482;0.
[-0.2866;-	[0.5;0.5;-0	[-0.3874;-	[0.3239;0.	[-0.2678;-	[-0.3366;-	[-0.239;-0.	[0.5008;0.	[-0.3214;-
[-0.5774;-	[0;1;0;0]	[-0.2621;-	[0.3244;0.	[0;1;0;0]	[-0.7417;-	[0.8165;0.	[0;1;0;0]	[0;1;0;0]
[0.2815-0.	[-0.383-0.1	[0.4048+0.	[-0.01648+	[-0.6186;0	[-0.03883-	[0.2795-0.	[-0.2859;0	[0.5774-1.
[0.2815+0.	[-0.383+0.	[0.4048-0.	[-0.01648-	[-0.6186;0	[-0.03883+	[0.2795+0.	[0;0.7071;	[0.5774+1.
[0.2802;0.	[-0.431;-0.	[-0.3874;-	[0.3075;0.	[0.1871;0.	[0.2213;0.	[-0.1885;-	[-0.3589;-	[0.2535;0.
[-0.737;0.5	[0.5;-0.5;-	[-0.851;0.5	[0.655;-0.5	[-0.577;0.5	[0.888;-0.3	[-0.806;0.4	[-0.577;0;	[-0.756;0.3
[0;0;-1;0]	[0.653;-0.2	[0;0;-0.851	[0.498;-0.1	[0.657;0.2	[0;0.707;-	[-0.335;-0.	[0.657;-0.5	[0;0.707;-
[-0.591;-0.	[-0.5;-0.5;	[0;0;0.526;	[-0.542;-0.	[-0.228;0.4	[0;0;0;1]	[-0.273;0.1	[-0.228;-0.	[-0.562;-0.
[0.328;0.7	[0.271;0.6	[0.526;0.8	[0.168;0.4	[0.429;0.6	[0.46;0.62	[0.405;0.6	[0.429;0.5	[0.337;0.5
[0.725;0.3	[0.475;0.5	[-0.524;-0.	[0.593;0.4	[-0.624;-0.	[0.688;0.3	[-0.782;-0.	[0.463;0.6	[-0.442;-0.
[0.639;-0.6	[0.656;-0.7	[0.288;0.4	[0.684;-0.7	[0.682;-0.7	[0.688;-0.6	[0.577;-0.7	[-0.661;0.7	[0.735;-0.6
[0.141;0.5	[0.321;0.3	[0.688;-0.7	[-0.278;-0.	[-0.368;-0.	[0.108;0.4	[-0.187;-0.	[0.346;0.2	[-0.439;-0.
[-0.217;-0.	[-0.492;-0.	[-0.411;-0.	[-0.323;-0.	[-0.101;-0.	[-0.203;-0.	[-0.141;-0.	[-0.479;-0.	[-0.267;-0.
[0.828;0.3	[0.383;0.7	[0.524;0.3	[0.623;0.5	[-0.645;-0.	[0.846;0.3	[-0.925;-0.	[0.395;0.7	[0.286;0.9
[0.51;-0.7	[-0.797;0.4	[0.288;0.4	[0.479;-0.6	[0.723;-0.6	[0.464;-0.8	[0.339;-0.8	[-0.765;0.4	[0.88;-0.32
[-0.102;-0.	[-0.161;0.2	[0.688;-0.7	[0.537;-0.2	[-0.137;-0.	[0.129;0.0	[0.00746;-	[-0.232;0.3	[-0.232;0.1
[0.207;0.5	[0.439;0.2	[0.411;0.4	[0.307;0.3	[0.205;0.2	[0.231;0.4	[0.174;0.3	[0.452;0.2	[0.299;0.2
[-0.144+0.	[0.272+0.2	[-0.414;-0.	[-0.247+0.	[0;1;0;0]	[-0.577;-0.	[0.109-0.5	[0;1;0;0]	[0;1;0;0]
[0.417;0.6	[-0.416;-0.	[6.18e-17+	[0.416;0.6	[0.602;0.2	[0.249+0.4	[0.314;0.5	[0;-1;0;0]	[-0.417;-0.
[-0.378;-0.	[0.272-0.2	[6.18e-17-	[-0.247-0.	[0.298;0.6	[0.249-0.4	[-0.577;-0.	[0.258;0.7	[0.313;0.6
[-0.144-0.	[0;1;0;0]	[0.414;0.5	[0.406;0.5	[0.602;0.2	[0.307;0.6	[0.109+0.5	[-0.577;-0.	[-0.577;-0.
[-0.41746	[0.352847	[-0.52603	[-0.48360	[-0.64917	[-0.66266	[-0.64011	[0.653092	[-0.61721
[-0.67575	[0.352847	[-0.08980	[-0.20227	[-0.64917	[-0.21026	[-0.66495	[0.166731	[-0.44189
[-0.67575	[-0.162937	[-0.08980	[-0.20227	[-0.092362	[-0.210262	[-0.66495	[-0.073957	[0.124015
[0.452759	[-0.420831	[0.446236	[0.420447	[-0.35206	[0.375282	[-0.36060	[-0.34812	[-0.34207
[-0.500604	[0.338112	[-0.44623	[0.493896	[-0.686943	[0.660009	[-0.61262	[0.625543	[-0.644848
[0.143417	[0.338112	[0.089808	[-0.194123	[-0.705607	[0.743722	[-0.359255	[0.053639	[0.388745
[0.143417	[-0.215041	[0.089808	[-0.194123	[0.151654	[0.743722	[-0.359255	[0.053639	[-0.189486
[0.538815	[0.509713	[0.526035	[0.577685	[0.289117	[0.337695	[0.378453	[0.210599	[-0.354039

5020	5022	5058	5062	5064	5066	5068	5070	5074
[-0.532;-0.	[-0.512;-0.	[-0.0544+	[0;-0.707;	[0;0.707;-	[-0.116+0.	[-0.302;-0.	[0;-0.707;	[0.198+0.4
[0.153+0.3	[-0.288-0.2	[-0.0544-0	[0.109+0.5	[0;-0.707;	[-0.116-0.	[-0.707+3.	[0;0.707;-	[0.198-0.4
[0.153-0.3	[-0.288+0.	[0.707;-0.7	[0.109-0.5	[0;-0.707;-	[0.525;-0.	[-0.707-3.5	[0.707;-0.7	[0.707;-0.7
[-0.314;-0.	[0.31;0.53	[-0.258;-0.	[0.314;0.5	[0;0.707;0.	[-0.294;-0.	[0.302;0.6	[-0.323;-0.	[0.215;0.5
[-0.2805;-	[0.378;0.7	[-0.6395;-	[0.7701;0.	[0;-0.7071	[0.3158;0.	[-0.3253;-	[-0.5;-0.5;-	[-0.7282;-
[-0.3343+	[-0.3238;0	[0.5596;-0	[0.5774;-0	[0;-0.7071	[-0.4458;0	[-0.5774;0	[-0.5955;0	[0.3688;-0
[-0.3343-0	[0;0.7071;	[-0.2199+	[-0.2028;0	[0;0.7071;	[-0.06806-	[-0.573;0.	[0;0.7071;	[0.3492;0.
[-0.3321;-	[-0.2465;-	[-0.2199-0	[0;-0.7071	[0;0.7071;	[-0.06806+	[-0.2461;-	[-0.1993;-	[-0.145;0.1
[0.5453;0.	[-0.5405;-	[-0.6111;-	[-0.7716;-	[0;-0.7071	[-0.1036+	[-0.3253;-	[0.3102;0.	[-0.6934;-
[0.2479-0.	[-0.1885+	[-0.6111;-	[0.3027;-0	[0;-0.7071	[-0.1036-0	[0.5774;-0	[0;0.7071;	[-0.6934;-
[0.2479+0.	[-0.1885-0	[0.7536;-0	[0.6273;-0	[0;-0.7071	[0.5419;-0	[0.573;-0.	[0.5;-0.5;-	[0.5774;0;
[-0.2754;-	[0.2137;0.	[0.1263;0.	[-0.1659;-	[0;-0.7071	[-0.259;-0.	[-0.2461;-	[0.2383;0.	[-0.09701;
[-0.806;0.1	[0.815;-0.	[-0.577;0.	[0.657;-0.	[-0.851;0.	[0.657;-0.	[0;0;-0.707	[-0.5;0.5;0	[-0.577;0.
[-0.335;0.	[0;0.707;-	[-0.707;0;	[-0.577;0;-	[0;0;0.526	[-0.577;0;-	[-0.851;0.	[-0.698;0.	[0.72;0.16
[-0.273;-0.	[0.506;0.3	[0;0;-1;0]	[-0.429;-0.	[0;0;-0.851	[-0.429;-0.	[0.526;0.8	[-0.507;-0.	[0;0.577;-
[0.405;0.5	[0.282;0.5	[0.408;0.8	[-0.228;-0.	[0.526;0.8	[-0.228;-0.	[0;0;0.707	[0.0795;0.	[0.385;0.5
[0.574;0.4	[-0.615;-0.	[-0.69;-0.3	[0.79;0.30	[0.557;0.4	[0.589;0.4	[0.605;0.4	[0.673;0.4	[-0.785;-0.
[0.698;-0.7	[0.692;-0.7	[0.64;-0.6	[0.585;-0.	[0.435;-0.	[0.644;-0.7	[0.569;-0.	[0.676;-0.	[0.549;-0.7
[-0.294;-0.	[0.304;0.4	[-0.325;-0.	[-0.101;0.	[-0.53;0.4	[0.244;-0.1	[0.47;-0.5	[-0.0472;0	[-0.285;-0.
[-0.311;-0.	[-0.224;-0.	[0.094;0.4	[-0.152;-0.	[-0.468;-0.	[0.423;0.4	[-0.299;-0.	[-0.297;-0.	[-0.0219;0
[0.652;0.5	[0.676;0.6	[0.853;0.2	[0.906;0.2	[0.557;0.4	[0.644;0.5	[-0.605;-0.	[0.774;0.4	[0.941;0.1
[0.6;-0.385	[0.697;-0.7	[0.368;-0.	[0.378;-0.7	[0.435;-0.	[0.564;-0.	[0.569;-0.	[0.51;-0.6	[0.196;-0.
[-0.335;0.	[0.0238;-0	[0.334;0.1	[-0.133;-0.	[0.53;-0.4	[0.398;-0.2	[0.47;-0.5	[0.28;-0.4	[0.239;-0.
[0.321;0.3	[0.239;0.2	[0.158;0.5	[0.139;0.6	[0.468;0.5	[0.329;0.4	[0.299;0.6	[0.249;0.4	[0.14;0.36
[-0.314;-0.	[-0.512;-0.	[-0.258;-0.	[0.109+0.5	[0;-0.707;	[-0.116-0.	[-0.302;-0.	[-0.323;-0.	[0.198+0.4
[-0.532;-0.	[-0.288-0.2	[-0.0544+	[0.109-0.5	[0;0.707;-	[0.525;-0.	[0.302;0.6	[0;0.707;-	[0.198-0.4
[0.153+0.3	[-0.288+0.	[-0.0544-0	[0.314;0.5	[0;-0.707;-	[-0.294;-0.	[-0.707+3.	[0;-0.707;	[0.707;-0.7
[0.153-0.3	[0.31;0.53	[0.707;-0.7	[0;-0.707;	[0;0.707;0.	[-0.116+0.	[-0.707-3.5	[0.707;-0.7	[0.215;0.5
[0.653728	[-0.593522	[-0.551358	[0.059553	[0.144337	[-0.147348	[-0.338214	[0.116466	[0.485082
[0.090423	[-0.206956	[-0.551358	[0.685690	[0.144337	[-0.147348	[-0.732072	[0.116466	[0.485082
[0.090423	[-0.206956	[0.764291	[0.685690	[0.381332	[0.662811	[-0.785394	[-0.753619	[0.777110
[0.365152	[-0.345841	[0.297315	[-0.317342	[-0.257145	[0.323655	[-0.316274	[0.323900	[-0.273894
[0.572037	[-0.579318	[-0.300964	[0.122738	[-0.257145	[0.140618	[0.316274	[-0.288675	[-0.57735
[0.307739	[-0.226701	[-0.300964	[0.714567	[-0.381332	[0.140618	[0.785394	[-0.288675	[0.757188
[0.307739	[-0.226701	[0.777477	[0.714567	[0.144337	[0.161850	[0.732072	[-0.759068	[0.788275
[-0.382669	[0.407535	[0.208828	[-0.377865	[0.144337	[-0.234753	[0.338214	[-0.437927	[0.172714

5076	5078	5080	5082	5084	5086	6342	6348	6350
[0.371;0.6	[0;-0.707;0	[0;0.707-3.	[-0.422-0.1	[0.429;0.5	[0.345;0.6	[-0.3;0.632	[-0.389+0.	[-0.447;0.7
[0.638;-0.2	[0.392+0.4	[0;0.707+3	[-0.422+0.	[-0.657;0.5	[0;0.707;-0	[-0.19-0.47	[-0.389-0.3	[0.5;-0.25-
[0.638;-0.2	[0.392-0.4	[0;-0.707+	[-0.468;0.7	[0.707;-0.7	[-0.707;0.7	[-0.19+0.4	[0;0;1;0]	[0.5;-0.25+
[0.207;0.6	[0.248;0.5	[0;-0.707-3	[0.234;0.5	[0.228;0.5	[-0.253;-0.	[-0.448;-0.	[-0.444;-0.	[0.447;0.2
[0.7534;0.	[-0.8438;-0	[0;0.8507;	[0.2859;0.	[-0.3825;-0	[0.5251;0.	[-0.4484;-0	[0;0;1;0]	[-0.3162;-0
[-0.6252;0	[0.5114;-0	[0;0.8507;	[-0.3589;0	[0.5148;-0	[-0.5277;0	[-0.1905+0	[0;0;1;0]	[-0.7071-6
[-0.2999;-0	[-0.1956;-0	[0;0.5257;	[0.3589;0.	[-0.2651;0	[0.08074;0	[-0.1905-0	[-0.7071;0	[-0.7071+6
[0.1905;0.	[-0.2101;-0	[0;-0.5257	[-0.2859;-0	[-0.2993;-0	[-0.2399;-0	[-0.3001;0	[0.7071;-0	[0.7071;-0
[-0.8377;-0	[0.9014;0.	[0;0.8507;	[0.05169-0	[0.6248;0.	[-0.7224;-0	[-0.3001;0	[0;0;1;0]	[-0.2209;0
[-0.1301+0	[0.3819;-0	[0;0.8507;	[0.05169+	[-0.4797;0	[0.2484;-0	[-0.1905-0	[-0.3461-0	[0.2887-0.
[-0.1301-0	[-0.3933;0	[0;0.5257;	[0.3868;-0	[0.378;-0.7	[0.3429;-0	[-0.1905+0	[-0.3461+0	[0.2887+0.
[0.1035;0.	[0.1255;0.	[0;-0.5257	[0.162;0.3	[0.1823;0.	[-0.1681;-0	[-0.4484;-0	[-0.66;-0.3	[0.5334;0.
[-0.768;0.2	[-0.768;0.4	[-0.768;0.2	[-0.768;0.4	[-0.577;0;0	[-0.756;0.3	[0;0.737;-0	[0;0;0.707;	[0;0.591;-0
[-0.423;0.5	[0.423;0.6	[-0.423;0.5	[0.423;0.6	[0.645;-0.6	[-0.218;0.6	[1;0;0;0]	[0;0.929;-0	[0;0.737;0.
[-0.312;-0.	[0.366;-0.2	[-0.312;-0.	[0.366;-0.2	[-0.423;-0.	[0.577;0.5	[0;-0.591;-	[1;0;0;0]	[1;0;0;0]
[0.366;0.4	[0.312;0.5	[0.366;0.4	[0.312;0.5	[0.267;0.3	[0.218;0.3	[0;0.328;0.	[0;0.369;0.	[0;0.328;0.
[0.794;0.3	[-0.86;-0.2	[0.594;0.4	[-0.618;-0.	[0.669;0.4	[-0.724;-0.	[-0.563;-0.	[0.482;0.5	[-0.534;-0.
[0.581;-0.5	[0.482;-0.6	[0.644;-0.7	[0.673;-0.7	[0.683;-0.6	[0.639;-0.6	[0.311;-0.6	[0.216;0.1	[-0.0449;0
[-0.114;-0.	[-0.147;-0.	[0.239;-0.1	[-0.378;-0.	[-0.0502;0	[-0.153;-0.	[0.373;-0.5	[0.203;-0.8	[-0.489;0.4
[-0.138;-0.	[-0.0879;-0	[0.417;0.4	[-0.15;0.07	[-0.289;-0.	[-0.21;-0.1	[0.669;0.1	[-0.824;0.1	[-0.688;0.0
[-0.895;-0.	[0.958;0.1	[0.639;0.5	[0.681;0.6	[0.777;0.4	[0.879;0.3	[0.238;-0.6	[0.21;0.48	[0.0987;-0
[0.402;-0.7	[0.227;-0.8	[0.561;-0.6	[0.62;-0.68	[0.497;-0.6	[0.384;-0.8	[0.652;-0.4	[0.38;0.08	[-0.619;-0.
[0.118;-0.0	[-0.116;-0.	[0.403;-0.3	[0.31;-0.22	[0.286;-0.4	[0.197;-0.2	[-0.57;-0.3	[0.535;-0.7	[0.515;-0.5
[0.154;0.6	[0.131;0.4	[0.338;0.4	[0.237;0.3	[0.258;0.4	[0.201;0.3	[0.44;0.48;	[0.725;0.3	[0.584;0.3
[0.371;0.6	[0.392+0.4	[0;0.707-3.	[0.234;0.5	[0.228;0.5	[-0.707;0.7	[-0.448;-0.	[-0.444;-0.	[0.447;0.2
[0.638;-0.2	[0.392-0.4	[0;0.707+3	[-0.468;0.7	[0.429;0.5	[0;0.707;-0	[-0.19-0.47	[-0.389+0.	[0.5;-0.25-
[0.638;-0.2	[0;-0.707;0	[0;-0.707+	[-0.422+0.	[0.707;-0.7	[0.345;0.6	[-0.19+0.4	[-0.389-0.3	[0.5;-0.25+
[0.207;0.6	[0.248;0.5	[0;-0.707-3	[-0.422-0.1	[-0.657;0.5	[-0.253;-0.	[-0.3;0.632	[0;0;1;0]	[-0.447;0.7
[-0.456802	[-0.025542	[0.160065	[0.510680	[0.579381	[-0.471404	[-0.267948	[-0.209707	[-0.410592
[-0.71584	[-0.68815	[0.160065	[0.510680	[-0.677705	[-0.406281	[0.134510	[-0.209707	[-0.575873
[-0.71584	[-0.68815	[0.318907	[-0.561144	[-0.765274	[0.770674	[0.134510	[0.163907	[-0.575873
[-0.26568	[-0.277965	[0.251131	[0.282525	[0.278322	[0.279967	[0.347078	[0.275819	[0.334111
[-0.438432	[-0.53356	[0.313690	[-0.601634	[-0.465727	[0.537346	[-0.229271	[0.377964	[-0.33148
[0.848171	[-0.53356	[-0.48238	[-0.601634	[-0.753952	[-1.14666	[-0.229271	[-0.37508	[-0.33148
[0.848171	[-0.961307	[0.096434	[-0.149562	[-0.745701	[0.762000	[-0.38034	[-0.329292	[-0.350725
[-0.16176	[-0.27645	[0.096434	[0.177617	[-0.177485	[-0.307464	[-0.55465	[0.547454	[-0.602302

6356	6358	6364	6366	6550	6552	6554	6558	6598
[-0.289+0.	[-0.354;0.5	[-0.407+0.	[-0.5;0.5;-0	[0.5+0.333	[-0.707;0;0	[-0.707;0;0	[0.707;0;0;	[-0.369+0.
[-0.289-0.5	[0.289+0.5	[-0.407-0.3	[-0.287+0.4	[0.5-0.333i	[0;1;0;0]	[-0.683;0.4	[-0.707;0;0	[-0.369-0.2
[0;0;1;0]	[0.289-0.5i	[0;0;1;0]	[-0.287-0.4	[0;-0.707;0	[0;0;1;0]	[0;1;0;0]	[0;-0.707;0	[-0.371;0.6
[-0.289;-0.	[-0.354;-0.	[-0.338;-0.	[-0.352;-0.	[0.333;0.5	[0.316;0.6	[0.336;0.5	[0.324;0.4	[-0.339;-0.
[-0.2887;-0	[0.4436;0.4	[-0.1371;-0	[0.2953;0.4	[-0.3288;-0	[0;1;0;0]	[0;1;0;0]	[0.1989;0.0	[-0.4014;-0
[0;0;1;0]	[-0.379+0.4	[0;0;1;0]	[0.7071;-0	[0;-0.7071	[0;0;1;0]	[0.09058;-0	[0;-0.7071	[0;0.7071;-
[-0.2887-0	[-0.379-0.4	[-0.7071;0	[-0.7071;0	[0.7288;-0	[0.3162;-0	[0.6667;-0	[0.7163;-0	[-0.3475-0
[-0.2887+0	[0.3634;-0	[0.7484;-0	[-0.4695;0	[-0.3565;-0	[0.7071;0;0	[-0.4932;-0	[-0.3378;-0	[-0.3475+0
[-0.2887-0	[-0.109+0.5	[0.05977-0	[0;0.7071;-	[0.1571+0.	[0;1;0;0]	[0;1;0;0]	[0.5+1.179	[0.3639-0.
[-0.2887+0	[-0.109-0.5	[0.05977+0	[0.2135+0.	[0.1571-0.4	[0;0;1;0]	[-0.5774;0	[0.5-1.179	[0.3639+0.
[0;0;1;0]	[0;0.7071;-	[0;0;1;0]	[0.2135-0.4	[0;0.7071;-	[-0.7071;0	[-0.5774;0	[0.334;-0.1	[-0.5774;0
[-0.2887;-0	[-0.2627;-0	[-0.4931;-0	[0.3385;0.	[-0.276;-0.	[0.5883;0.	[0.3904;0.	[0.2887;0.	[-0.2627;-0
[-0.5;-0.5;0	[-0.582;-0.	[-0.577;0;0	[-0.691;-0.	[0;0.707;-0	[-0.632;0.3	[0;0.707;-0	[0;0.707;-0	[0.657;-0.5
[0.0674;0.0	[0.707;-0.7	[0.239;0.8	[-0.207;0.8	[0.929;-0.2	[0;0.707;-0	[0.823;-0.3	[0.878;-0.2	[0.577;0;-0
[0.814;-0.4	[-0.299;-0.	[-0.76;0.35	[0.676;-0.1	[0;0;0;1]	[-0.707;0;0	[0.484;0.1	[0.395;0.2	[-0.429;-0.
[0.289;0.2	[0.268;0.2	[0.178;0.3	[0.15;0.29	[0.369;0.6	[0.316;0.6	[0.298;0.6	[0.269;0.5	[0.228;0.5
[-0.541;-0.	[0.608;0.5	[0.528;0.5	[0.553;0.6	[0.593;0.4	[-0.479;-0.	[-0.468;-0.	[0.542;0.5	[0.601;0.4
[-0.707;0.7	[0.607;-0.7	[-0.134;0.7	[0.387;-0.7	[0;0.707;-0	[0;0.707;-0	[-0.227;0.6	[0;0.707;-0	[0.642;-0.0
[-0.408;-0.	[-0.504;-0.	[0.456;-0.3	[0.625;-0.0	[0.794;-0.4	[0.52;-0.47	[0.745;-0.3	[0.793;-0.4	[0.466;-0.5
[0.202;0.2	[-0.0838;-0	[-0.704;0.0	[-0.393;0.0	[-0.13;-0.3	[-0.707;0;0	[-0.418;-0.	[-0.28;-0.2	[-0.102;-0.
[-0.347;-0.	[0.718;-0.6	[0.0829;-0	[0.359;-0.8	[-0.589;0.5	[0.297;-0.6	[0.278;-0.9	[-0.315;0.6	[-0.751;-0.
[0.478;-0.8	[-0.461;-0.	[-0.109;-0.	[-0.209;-0.	[0;0.707;-0	[0;0.707;-0	[-0.0441;-0	[0;0.707;-0	[0.577;-0.5
[0.662;0.0	[0.434;0.6	[0.801;-0.2	[0.813;0.1	[0.761;0.2	[0.707;0;0;	[0.84;0.16;	[-0.884;-0.	[0.115;0.4
[0.461;0.4	[0.289;0.2	[0.583;0.3	[0.408;0.2	[0.273;0.3	[0.642;0.2	[0.465;0.2	[0.346;0.2	[0.298;0.6
[-0.289-0.5	[-0.354;-0.	[0;0;1;0]	[-0.287+0.4	[0.5-0.333i	[-0.707;0;0	[0;1;0;0]	[0;-0.707;0	[-0.369-0.2
[-0.289;-0.	[0.289+0.5	[-0.407+0.	[-0.287-0.4	[0.333;0.5	[0;1;0;0]	[-0.683;0.4	[0.324;0.4	[-0.339;-0.
[0;0;1;0]	[0.289-0.5i	[-0.407-0.3	[-0.5;0.5;-0	[0;-0.707;0	[0;0;1;0]	[0.336;0.5	[-0.707;0;0	[-0.369+0.
[-0.289+0.	[-0.354;0.5	[-0.338;-0.	[-0.352;-0.	[0.5+0.333	[0.316;0.6	[-0.707;0;0	[0.707;0;0;	[-0.371;0.6
[-0.288675	[-0.321992	[-0.352700	[-0.500000	[0.615939	[0.707106	[0.801176	[-0.802610	[-0.375101
[-0.288675	[-0.620127	[-0.352700	[-0.309778	[0.615939	[-0.075101	[0.743016	[-0.802610	[0.405817
[0.229415	[-0.620127	[0.122576	[-0.309778	[0;-0.7071	[-0.075101	[0.213808	[-4.408417	[0.405817
[-0.288675	[0.338461	[-0.299164	[-0.316998	[-0.362488	[-0.316227	[0.329082	[0.334940	[0.320881
[-0.288675	[-0.608651	[-0.495531	[-0.588642	[-0.561920	[-0.707106	[0.679919	[-0.602005	[-0.398814
[-0.288675	[-0.628463	[-0.495531	[-0.291419	[-0.928671	[-0.316227	[-0.242927	[-0.556702	[-0.398814
[-0.288675	[-0.628463	[0.365636	[-0.291419	[0;-0.7071	[-0.279347	[-0.314389	[-3.102726	[-0.518380
[-0.377964	[0.496358	[0.413613	[-0.525797	[0.421959	[0.294862	[0.439447	[-0.445895	[0.429926

6602	6604	6606	6614	6616	6618	6620	6622	6854
[-0.683;0.4	[0.577;0;-	[0.439-0.1	[-0.408-0.	[-0.707;0;	[0.707;0;0;	[0.707;0;0;	[-0.707;0;	[0;-0.707;
[-0.354+0.	[0.683;-0.4	[0.439+0.1	[-0.408+0.	[0;0;1;0]	[-0.433+0.	[0.724;-0.4	[0.619;-0.1	[-0.189-0.5
[-0.354-0.	[0;0;1;0]	[0.603;-0.7	[-0.47;0.5	[0;0;-1;0]	[-0.433-0.1	[0;0;1;0]	[0.619;-0.1	[-0.189+0.
[0.336;0.5	[-0.336;-0.	[-0.338;-0.	[0.285;0.4	[0.213;0.4	[-0.283;-0.	[-0.276;-0.	[0.284;0.4	[0.353;0.5
[0.1994;0.	[0;0;1;0]	[0.2567;0.	[0.4419;0.	[0;0;1;0]	[0.1772;0.	[0.06035;0	[-0.2715;-	[-0.5098;-
[-0.383+0.	[-0.1371;-	[0;0.7071;	[-0.5774;0	[0;0;1;0]	[0.5774;-0	[0;0;1;0]	[-0.2164+	[-0.2549+
[-0.383-0.1	[0.7484;-0	[0.4437;-0	[0.3689;0.	[0;0;1;0]	[-0.5774;0	[0.5791;-0.	[-0.2164-0	[-0.2549-0
[0.7501;-0	[-0.6882;0	[-0.5163;-	[-0.3424;-	[0.7071;0;	[-0.4913;-	[-0.4734;-	[0.3316;0.	[0;-0.7071
[0.06531-	[0;0;1;0]	[0.09725-	[0.08986-	[0;0;1;0]	[-0.5;0.5;-	[0;0;1;0]	[-0.4892;0	[-0.6731;0.
[0.06531+	[0.5774;0;	[0.09725+	[0.08986+	[0;0;-1;0]	[-0.1148+	[-0.5944;0.	[-0.3397+	[-0.6731;0.
[-0.5774;0	[0.7371;-0.	[0.6625;-0	[-0.3095;0	[-0.7071;0.	[-0.1148-0	[-0.5944;0.	[-0.3397-0	[0;0.7071;
[-0.468;-0.	[-0.3846;-	[-0.3323;-	[0.2103;0.	[0.4364;0.	[0.3059;0.	[0.3177;0.	[-0.2489;-	[-0.2129;-
[0.721;-0.	[0;0;-0.707	[0.7;-0.56	[0.229;-0.7	[0.577;-0.	[0.651;-0.	[0.577;0;-	[-0.79;0.14	[0;0.408;0.
[0;0;0.707;	[0.764;-0.	[0.352;-0.1	[0.892;-0.1	[0.289;0.5	[0.559;0.4	[0.484;-0.7	[0.263;-0.7	[0;0.707;-
[0.665;0.4	[-0.632;-0.	[-0.613;-0.	[-0.242;-0.	[-0.707;0;	[0.433;0.0	[-0.614;-0.	[0.512;0.4	[1;0;0;0]
[0.195;0.6	[0.128;0.4	[0.1;0.424;	[0.306;0.5	[0.289;0.5	[0.276;0.5	[0.234;0.5	[0.212;0.4	[0;0.577;0.
[0.522;0.5	[0.467;0.3	[0.553;0.4	[0.664;0.4	[0.529;0.4	[0.551;0.5	[0.556;0.4	[0.602;0.5	[-0.593;-0.
[-0.0458;0	[0.337;0.3	[-0.318;-0.	[0.739;-0.4	[-0.0722;0	[0.334;-0.	[-0.302;-0.	[-0.38;0.82	[-0.74;0.0
[0.49;-0.4	[-0.654;0.7	[0.668;-0.7	[0.0322;0.	[0.463;-0.4	[0.675;-0.1	[0.675;-0.7	[0.651;-0.	[0.319;-0.5
[-0.697;-0.	[-0.49;-0.4	[-0.382;-0.	[-0.108;-0.	[-0.707;0;	[-0.361;-0.	[-0.38;-0.3	[-0.262;-0.	[0;0.707;-
[0.0971;-0	[0.265;0.3	[0.267;0.4	[0.955;-0.2	[0.0406;-0	[-0.368;0.	[0.278;0.3	[0.478;-0.7	[0.624;0.0
[-0.795;0.1	[0.339;0.3	[-0.769;-0.	[-0.112;0.	[-0.707;0;	[0.535;0.3	[0.491;0.3	[0.743;0.0	[0;0.707;-
[-0.0147;0	[0.746;-0.	[-0.406;0.7	[0.143;0.8	[-0.388;0.	[0.634;0.0	[0.714;-0.	[0.345;0.5	[0.745;-0.2
[0.599;0.3	[0.508;0.5	[0.415;0.4	[0.235;0.4	[0.59;0.38	[0.421;0.2	[0.414;0.4	[0.317;0.3	[0.235;0.6
[-0.683;0.4	[0;0;1;0]	[-0.338;-0.	[-0.408-0.	[0;0;1;0]	[0.707;0;0;	[0;0;1;0]	[0.619;-0.1	[-0.189+0.
[0.336;0.5	[0.577;0;-	[0.439-0.1	[-0.408+0.	[0;0;-1;0]	[-0.433+0.	[0.707;0;0;	[0.619;-0.1	[0.353;0.5
[-0.354+0.	[0.683;-0.4	[0.439+0.1	[-0.47;0.5	[-0.707;0;	[-0.433-0.1	[0.724;-0.4	[-0.707;0;	[-0.189-0.5
[-0.354-0.	[-0.336;-0.	[0.603;-0.7	[0.285;0.4	[0.213;0.4	[-0.283;-0.	[-0.276;-0.	[0.284;0.4	[0;-0.707;
[0.766213	[0.605735	[-0.472302	[0.618107	[0.707106	[-0.784213	[0.766315	[-0.77464	[-3.768221
[-0.36236	[0.605735	[-0.472302	[0.618107	[-0.124514	[-0.489824	[0.766315	[0.687568	[-0.16444
[-0.36236	[0.197332	[0.636304	[-0.505321	[-0.136594	[-0.489824	[-0.157432	[0.687568	[-0.16444
[0.304119	[0.282741	[0.309380	[-0.297872	[0.263485	[0.289252	[0.274447	[-0.285754	[-0.22868
[-0.423083	[0.441184	[0.470595	[-0.74214	[0.707106	[0.694586	[0.669622	[-0.648313	[0;0.70710
[-0.26294	[-0.432614	[0.470595	[-0.74214	[0.188982	[0.019050	[0.665376	[-0.38076	[-0.188792
[-0.26294	[-0.326077	[0.461860	[-0.83034	[0.188982	[0.019050	[0.354986	[-0.38076	[-0.188792
[-0.440671	[-0.440794	[-0.464024	[-0.387701	[0.362142	[0.401426	[-0.377964	[-0.415887	[-0.469117

6858	6862	6870	6874	6876	6878	7126	7128	7130
[-0.577;0.5	[0;-0.707;0	[0;-0.707;0	[-0.365;0.5	[-0.241+0.4	[0;0.707;-0	[0;-0.707;0	[-0.0544;-0	[-0.707;0;0
[-0.683;0.4	[0.648;-0.3	[-0.289-0.5	[-0.705;0.2	[-0.241-0.4	[0.619;-0.1	[0.664;-0.0	[0.707;0;0;	[-0.707;0;0
[-0.577;0.5	[0.648;-0.3	[-0.289+0.5	[0.577;-0.5	[-0.577;0.5	[0.619;-0.1	[0.664;-0.0	[0;0.707;0.	[0.577;-0.5
[0.336;0.54	[-0.372;-0.	[-0.275;-0.	[-0.263;-0.	[0.247;0.52	[0.284;0.41	[-0.227;-0.	[0;0.707;0.	[-0.209;-0.
[0.2843;0.0	[-0.3721;-0	[0.5;0.5;0.5	[0.1944;0.7	[-0.2474;-0	[-0.3361;-0	[0.5318;0.5	[0;-0.7071;	[0.2209;0.7
[0.378;-0.7	[0.6478;-0.	[-0.4274+0	[0.3538;-0.	[0.5774;-0.	[0.6826;-0.	[0.6325;-0.	[0;-0.7071;	[0.4082;-0.
[0.6186;-0.	[0.6478;-0.	[-0.4274-0	[-0.7318;0.	[-0.2412-0	[0.5774;0;-	[-0.3091;-0	[-0.7071;0;	[0.5334;0.3
[0.6186;-0.	[0;-0.7071;	[0.06649;-0	[-0.2926;0.	[-0.2412+0	[-0.2357;0.	[0;-0.7071;	[0.101;-0.6	[-0.4082;0;
[-0.3606;0.	[0;-0.7071;	[0.6407;-0.	[-0.2027;0.	[-0.2412+0	[-0.08141+	[-0.6816;0.	[0.05351;-0	[0.683;-0.5
[-0.5774;0;	[-0.6478;0.	[0.6407;-0.	[-0.6755;-0	[-0.2412-0	[-0.08141-	[-0.6816;0.	[0.7071;0;0	[-0.7071+2
[-0.5821;0.	[-0.6478;0.	[0.3422;0.0	[-0.7071;0;	[-0.5774;0.	[0.7071;0;-	[0;0.7071;-	[0;0.7071+	[-0.7071-2
[-0.3687;-0	[-0.3721;-0	[0.1471;0.3	[-0.2064;-0	[0.2474;0.5	[0.2303;0.3	[-0.1311;-0	[0;0.7071-	[-0.183;-0.
[0;0.707;0;	[0;0.5;0.5;-	[0.842;0.18	[0;0.707;0;	[-0.577;0;0	[-0.534;0.3	[-0.871;0.2	[-0.653;0.2	[-0.577;0.5
[1;0;0;0]	[0;0.707;-0	[0;-0.707;0	[0.875;0.12	[-0.577;-0.	[0.625;0.65	[0;0.707;-0	[-0.367;0.6	[0.657;0.42
[0;0.325;-0	[1;0;0;0]	[0.5;-0.5;0	[-0.462;0.4	[0.553;-0.7	[-0.558;0.4	[-0.416;-0.	[0.604;0.30	[-0.429;0.2
[0;0.628;0.	[0;0.5;0.5;0	[0.204;0.40	[0.147;0.55	[0.167;0.38	[0.114;0.45	[0.261;0.64	[0.271;0.65	[0.228;0.65
[-0.537;-0.	[-0.583;-0.	[-0.655;-0.	[-0.551;-0.	[-0.572;-0.	[0.611;0.51	[-0.721;-0.	[0.574;0.41	[-0.609;-0.
[0.129;-0.7	[0.428;-0.5	[0.751;-0.4	[0.295;-0.7	[-0.423;0.5	[0.603;-0.7	[0.687;-0.4	[0.413;-0.5	[0.61;-0.73
[-0.489;-0.	[0;0.707;-0	[0.0793;0.0	[-0.766;0.0	[0.502;-0.5	[-0.453;-0.	[0;-0.707;0	[0.247;0.60	[-0.455;-0.
[-0.675;0.4	[0.691;-0.0	[-0.0319;0.	[-0.15;0.30	[0.492;0.43	[-0.242;0.2	[-0.0872;-0	[0.662;-0.2	[0.222;-0.1
[-0.352;0.6	[0;0.707;-0	[0.902;-0.1	[0.37;-0.72	[0.492;0.55	[0.569;-0.5	[0.977;-0.0	[0.427;0.50	[-0.602;0.5
[0.357;0.59	[-0.691;-0.	[0.339;0.58	[-0.518;-0.	[-0.504;0.4	[-0.624;-0.	[0;0.707;-0	[-0.564;0.4	[-0.585;-0.
[0.682;-0.3	[0.583;-0.4	[0.18;-0.65	[0.678;-0.2	[0.553;-0.5	[0.428;-0.4	[-0.121;0.4	[0.547;-0.4	[0.433;-0.3
[0.533;0.41	[0.428;0.51	[0.197;0.44	[0.368;0.30	[0.446;0.49	[0.321;0.30	[0.177;0.57	[0.448;0.54	[0.329;0.42
[-0.577;0.5	[-0.372;-0.	[0;-0.707;0	[-0.705;0.2	[0.247;0.52	[0.284;0.41	[-0.227;-0.	[0;0.707;0.	[-0.209;-0.
[-0.577;0.5	[0.648;-0.3	[-0.289-0.5	[-0.365;0.5	[-0.577;0.5	[0.619;-0.1	[0.664;-0.0	[0;0.707;0.	[0.577;-0.5
[-0.683;0.4	[0.648;-0.3	[-0.289+0.5	[0.577;-0.5	[-0.241+0.4	[0.619;-0.1	[0.664;-0.0	[0.707;0;0;	[-0.707;0;0
[0.336;0.54	[0;-0.707;0	[-0.275;-0.	[-0.263;-0.	[-0.241-0.4	[0;0.707;-0	[0;-0.707;0	[-0.0544;-0	[-0.707;0;0
[0.6574127	[2.3469415	[-0.065633	[-0.376520	[-0.229775	[-0.122477	[-7.712437	[0.7071067	[0.6730014
[0.6574127	[-0.726056	[-0.629283	[-0.831823	[-0.229775	[-0.685668	[0.7064595	[0.1064388	[0.6730014
[0.7488126	[-0.726056	[-0.629283	[0.7529101	[-0.787269	[-0.685668	[0.7064595	[0.4015435	[0.7909814
[0.2073465	[0.2529154	[-0.245643	[0.2346240	[-0.231799	[0.2514343	[0.2342343	[0.1968825	[0.2272283
[-0.432512	[0;-0.70710	[-0.180183	[0.3645440	[-0.275414	[0.3238835	[7.2957513	[0.7071067	[0.5993929
[-0.447213	[-0.483105	[-0.600151	[-0.529591	[-0.275414	[0.5619702	[0.8268390	[-0.062554	[0.7514585
[-0.088340	[-0.483105	[-0.600151	[0.0968360	[-0.121230	[0.5619702	[0.8268390	[0.2041241	[-0.159111
[0.4472135	[-0.546885	[0.3656805	[-0.351083	[-0.265231	[0.4138130	[-0.286882	[0.2041241	[-0.217413

7134	13140	13150	13150	13150	13260	13260	13280	14680
[-0.671;0.2	[-0.632;-0.	[0.378;0.3	[0.378;0.3	[0.347;0.3	[0.5;0.5;-0	[0.523;0.5	[0.557;0.5	[-0.378;-0.
[-0.5;-0.5;	[0.221;0.2	[-0.614;-0.	[0.378;0.3	[0;0;0.707	[0;0;0.707	[0.197;0.1	[0;0;-0.707	[0.667;2.2
[-0.813;0.3	[1;0;0;0]	[-0.5;0.5;0	[-0.5;0.5;0	[-0.756;0.3	[0.707;-0.7	[0.707;-0.7	[0.707;-0.7	[0.667;2.2
[0.233;0.5	[0.5;0.5;0.	[-0.5;-0.5;-	[-0.5;-0.5;-	[-0.461;-0.	[-0.5;-0.5;-	[0.47;0.47	[-0.435;-0.	[-0.5;-0.5;-
[0.3354;0.	[1;0;0;0]	[0.6325;0.	[-0.7559;-	[0.8116;0.	[0.5;0.5;0.	[0.5318;0.	[-0.5573;-	[0.7035;0.
[0.5911;-0	[0.8629;-0	[-0.6708;0	[-0.7413;0	[-0.809;0.	[0;0;-0.707	[-0.7071;0	[0.7071;-0	[-0.6238;-
[0;-0.7071	[0.2811;0.	[0.5;0.5;-0	[0;0;-0.707	[0.309;0.5	[0.7071;-0	[0.3091;0.	[0.4352;0.	[-0.6238;-
[-0.3203;-	[0;0;-0.707	[0;0;0.707	[-0.2034;-	[0.1297;0.	[-0.5;-0.5;	[-0.4082;-	[0;0;0.707	[-0.2109;-
[-0.6794;-	[1;0;0;0]	[-0.6808;-	[-0.8468;-	[0.9051;0.	[0.5;0.5;-0	[0.5804;0.	[0.6325;0.	[-0.3433+
[0.8165;-0	[0.4563;-0	[-0.5;0.5;-	[0.8165;0;	[0.6087;-0	[0;0;-0.707	[0.7071;-0	[0.7071;-0	[-0.3433-0
[0.683;-0.	[0;0;-0.707	[0;0;0.707	[0;-0.5774	[0.06415;	[0.7071;-0	[0.1527;0.	[0;0;-0.707	[-0.4578;-
[0.183;0.5	[0.2565;0.	[0.3462;0.	[-0.3409;-	[-0.2764;-	[0.5;0.5;0.	[0.4035;0.	[0.3162;0.	[0.3457;0.
[-0.742;0.3	[0.707;-0.7	[0.707;-0.7	[0.707;-0.7	[0.707;-0.7	[-0.707;0.7	[0.707;-0.7	[0.707;-0.7	[0.435;-0.5
[0;0.707;-	[0;0;-0.707	[-0.372;-0.	[-0.227;-0.	[-0.24;-0.2	[0;0;-0.707	[-0.171;-0.	[0;0;0.707	[0.707;0;-
[-0.645;-0.	[-0.435;-0.	[0;0;0.707	[-0.29;-0.2	[0.408;0.4	[0;0;0.707	[-0.518;-0.	[-0.557;-0.	[0;-0.707;
[0.186;0.5	[0.557;0.5	[0.602;0.6	[0.604;0.6	[0.525;0.5	[0.707;0.7	[0.45;0.45	[0.435;0.4	[0.557;0.4
[0.656;0.4	[-0.804;-0.	[-0.543;-0.	[0.596;0.4	[-0.654;-0.	[0.5;0.5;0.	[0.531;0.5	[-0.557;-0.	[0.587;0.4
[0.715;-0.4	[0.553;-0.	[0.703;-0.7	[0.737;-0.	[0.693;-0.	[0;0;-0.707	[0.707;-0.7	[0.707;-0.7	[0.802;-0.4
[0;0.707;-	[-0.218;-0.	[-0.453;-0.	[0.0412;0.	[-0.248;-0.	[0.707;-0.7	[0.00772;	[-0.435;-0.	[0.0931;0.
[-0.242;-0.	[0;0;-0.707	[-0.0745;0	[-0.316;-0.	[-0.176;-0.	[-0.5;-0.5;	[-0.466;-0.	[0;0;0.707	[-0.0696;-
[-0.932;-0.	[0.94;0.16	[-0.637;-0.	[0.712;0.4	[0.852;0.3	[0.5;0.5;-0	[0.582;0.5	[-0.632;-0.	[0.917;-0.1
[0;0.707;-	[0.276;-0.	[0.671;-0.	[0.612;-0.	[0.449;-0.	[0;0;-0.707	[0.707;-0.7	[0.707;-0.7	[0.28;0.31
[0.248;-0.	[0;0;0.707	[-0.224;0.2	[0.176;-0.4	[0.0984;-0	[0.707;-0.7	[0.00867;	[0;0;0.707	[0.0589;-0.
[0.264;0.4	[0.202;0.4	[0.306;0.3	[0.296;0.4	[0.251;0.3	[0.5;0.5;0.	[0.401;0.4	[0.316;0.3	[0.278;0.4
[-0.813;0.3	[1;0;0;0]	[0.378;0.3	[0.378;0.3	[-0.756;0.3	[0.5;0.5;-0	[0.47;0.47	[0.557;0.5	[-0.378;-0.
[0.233;0.5	[0.5;0.5;0.	[-0.5;0.5;0	[-0.5;-0.5;-	[0;0;0.707	[0;0;0.707	[0.523;0.5	[0;0;-0.707	[0.667;2.2
[-0.671;0.2	[-0.632;-0.	[-0.5;-0.5;-	[0.378;0.3	[-0.461;-0.	[0.707;-0.7	[0.707;-0.7	[0.707;-0.7	[-0.5;-0.5;-
[-0.5;-0.5;	[0.221;0.2	[-0.614;-0.	[-0.5;0.5;0	[0.347;0.3	[-0.5;-0.5;-	[0.197;0.1	[-0.435;-0.	[0.667;2.2
[-2.511034	[-5.59713	[9.420554	[-0.36994	[2.706119	[0.5;0.5;-0	[-0.45649	[-2.455237	[-0.355542
[-0.75375	[0.617003	[-0.62413	[-0.36994	[-0.11050	[-0.705821	[-0.45649	[0.597911	[0.687534
[-0.908837	[-0.96476	[0.5;-0.5;-	[0.5;-0.5;-	[-0.83591	[-0.042621	[-0.70710	[-0.70710	[0.687534
[-0.23538	[-0.52738	[0.556882	[-0.53233	[-0.505381	[0.5;0.5;0.	[0.489645	[-0.46829	[-0.54306
[-0.67777	[0.685994	[4.592051	[-0.55349	[0.415053	[0.5;0.5;-0	[0.560504	[0.615917	[0.489050
[-4.13855	[-0.586262	[0.701646	[-0.236277	[-0.317971	[0.042621	[0.262703	[7.226237	[0.805250
[-0.66658	[0.919145	[-0.64993	[-0.717317	[0.918895	[-0.705821	[-0.70710	[0.707106	[0.805250
[-0.31875	[0.5;0.5;0.	[-0.5;-0.5;-	[-0.5;-0.5;-	[0.490577	[-0.5;-0.5;-	[0.502000	[-0.484277	[-0.5;-0.5;-

14690	14790	14800	14810	14810	14810	15260	15260	15310
[-0.347;-0.	[-0.5;-6.25	[0.616;-0.2	[-0.707;0;	[-0.707;0;	[-0.707;0;	[-0.816;0;	[-0.707;0;	[-0.707;0;
[-0.289-0.	[-0.5;-6.25	[0.616;-0.2	[-0.217+0.	[0.702;-0.4	[0.715;-0.3	[0.226;0;-	[-0.518;0;-	[-0.707;0;
[-0.289+0.	[-0.5;0.5;-	[0.606;-0.6	[-0.217-0.2	[0.702;-0.4	[0.715;-0.3	[0;1;0;0]	[0.408;-0.	[-0.408;0.
[-0.461;-0.	[-0.5;-0.5;-	[0.463;0.4	[-0.477;-0.	[0.491;0.4	[0.449;0.4	[0.436;0.6	[-0.408;-0.	[-0.408;-0.
[-0.5251;-	[-0.5;-0.5;-	[-0.3893;-	[-0.2832;-	[0.4024;0.	[0.4044;0.	[0;-1;0;0]	[-0.3162;-	[0.4082;0.
[0.5774;-0	[-0.5;0.5;-	[0.7071;-0	[-0.4082;0	[0.5952;-0	[0.7071;-0	[0;1;0;0]	[-0.3162;0	[-0.4082;0
[0.5774;-0	[-1.388e-1	[-0.7071;0	[0.425;-0.3	[0.7071;-0	[0.7994;-0	[0.8165;0;	[-0.7071;0	[0.7071;0;
[0.2399;0.	[-1.388e-1	[0.8446;-0	[-0.7071;0	[0.7071;0;	[-0.3877;-	[0.2933;0;	[-0.3162;-	[-0.7071;0
[-0.5774;0	[4.441e-16	[0.4133+1.	[0.147+0.1	[0.6374;-0	[-0.09593-	[0;1;0;0]	[0;-0.5774	[-0.7071;0
[-0.5774;0	[4.441e-16	[0.4133-1.	[0.147-0.1	[0.6374;-0	[-0.09593+	[-0.8165;0	[-0.4117;-	[-0.7071;0
[-0.4614;-	[-0.5;0.5;-	[-0.5257;0	[0.7071;0;	[-0.7071;0	[-0.6017;0	[-0.4082;0	[0.4082;0.	[-0.4082;0
[-0.347;-0.	[-0.5;-0.5;-	[-0.5257;-	[-0.5299;-	[0.5648;0.	[-0.4284;-	[0.5298;0.	[-0.4082;-	[-0.4082;-
[0.789;-0.2	[-0.5;0.5;-	[0.423;-0.3	[0.103;-0.8	[0.193;0.3	[0.191;-0.6	[-0.378;0.7	[0.442;-0.7	[0.602;-0.3
[-0.211;0.7	[0.707;0;-	[0.366;-0.7	[-0.707;0;	[0.481;-0.7	[0.855;-0.2	[0.752;0;-	[0.707;0;-	[0.372;-0.6
[0.347;0.3	[0;0.707;0	[0.768;0.3	[-0.575;0.2	[-0.807;-0.	[-0.341;-0.	[0.319;0;0.	[0.36;0.18	[-0.602;-0.
[0.461;0.4	[-0.5;-0.5;-	[0.312;0.4	[0.399;0.5	[0.283;0.5	[0.341;0.4	[0.436;0.6	[0.418;0.6	[0.372;0.6
[0.525;0.5	[0.5;0.5;0.	[0.492;0.5	[-0.46;-0.6	[-0.497;-0.	[-0.516;-0.	[0.461;0.6	[0.514;0.5	[-0.53;-0.4
[-0.211;0.7	[-0.5;0.5;-	[0.179;-0.6	[-0.35;0.75	[0.191;-0.6	[0.403;-0.7	[0.347;-0.7	[0.391;-0.8	[-0.479;0.5
[0.789;-0.2	[-0.433;-0.	[-0.518;0.5	[-0.707;0;	[-0.484;0.5	[0.701;-0.0	[0.0607;0;	[0.707;0;-	[0.468;-0.5
[-0.24;-0.2	[0.559;-0.4	[-0.676;-0.	[-0.407;0.0	[0.694;0.1	[0.283;0.2	[-0.814;0;	[-0.287;-0.	[-0.52;-0.4
[0.789;-0.2	[-0.43;-0.5	[0.326;0.6	[-0.18;0.96	[0.302;0.6	[-0.108;0.9	[-0.165;0.9	[-0.097;0.9	[0.468;0.5
[-0.211;0.7	[0.561;-0.4	[0.413;-0.5	[0.707;0;0	[0.409;-0.4	[0.701;0.0	[0.738;0;-	[0.707;0;-	[-0.52;0.47
[0.461;0.4	[-0.5;0.5;-	[-0.618;0.3	[0.395;0.1	[-0.64;0.36	[-0.541;0.1	[0.35;0;-0.	[-0.531;0.1	[-0.53;0.46
[0.347;0.3	[0.5;0.5;0.	[0.585;0.3	[0.558;0.2	[0.577;0.3	[0.453;0.3	[0.553;0.2	[0.457;0.3	[0.479;0.5
[-0.347;-0.	[-0.5;-6.25	[0.463;0.4	[-0.477;-0.	[0.491;0.4	[0.449;0.4	[-0.816;0;	[-0.408;-0.	[-0.408;-0.
[-0.289-0.	[-0.5;-0.5;-	[0.616;-0.2	[-0.707;0;	[0.702;-0.4	[0.715;-0.3	[0.436;0.6	[0.408;-0.	[-0.707;0;
[-0.289+0.	[-0.5;-6.25	[0.606;-0.6	[-0.217-0.2	[0.702;-0.4	[0.715;-0.3	[0;1;0;0]	[-0.707;0;	[-0.408;0.
[-0.461;-0.	[-0.5;0.5;-	[0.616;-0.2	[-0.217+0.	[-0.707;0;	[-0.707;0;	[0.226;0;-	[-0.518;0;-	[-0.707;0;
[-0.393011	[0.5;2.220	[0.577584	[-0.70710	[-0.57086	[-0.62287	[0.816496	[0.707106	[-0.632455
[-0.288675	[0.5;2.220	[0.577584	[-0.247389	[-0.693463	[0.690496	[0.129868	[-0.457423	[-0.632455
[-0.288675	[-0.5;0.5;-	[-0.70140	[-0.247389	[-0.693463	[0.690496	[-0.138675	[-0.472894	[-0.475421
[-0.481031	[-0.5;-0.5;-	[0.435795	[0.467585	[-0.425398	[-0.438232	[0.436435	[-0.421368	[0.396620
[-0.415053	[0-0.5i;-0.	[0.604744	[0.707106	[-0.711674	[-0.721722	[0.816496	[-0.429877	[-0.67082
[-0.57735	[0+0.5i;-0.	[0.604744	[-0.137769	[0.541704	[-0.739221	[0.143415	[-0.70710	[0.632455
[-0.57735	[-0.5;0.5;-	[0.354687	[-0.137769	[0.541704	[-0.680319	[0.288675	[0.151833	[0.240446
[-0.490577	[0.5;0.5;0.	[-0.482717	[0.517357	[0.540831	[-0.511948	[-0.436435	[0.442193	[-0.438389

15330	31710
[0;0;-0.707	[0.789;-0.211;-0.577;0]
[-0.816;0;0	[-0.211;0.789;-0.577;0]
[-0.845;0.3	[0.289;0.289;0.289;-0.866]
[0.389;0.5	[0.5;0.5;0.5;0.5]
[0.4241;0.	[0.5;0.5;0.5;0.5]
[0.7301;-0	[-0.2113;0.7887;-0.5774;0]
[0.6243;0.	[-0.2887;-0.2887;-0.2887;0.866]
[0;0;-0.707	[0.7887;-0.2113;-0.5774;0]
[0.7709;-0	[-0.03068;0.7219;-0.6913;0]
[0.7709;-0	[-0.8159;0.3814;0.4345;0]
[0;0;-0.707	[0.2887;0.2887;0.2887;-0.866]
[0.3462;0.	[0.5;0.5;0.5;0.5]
[0.645;-0.7	[0.707;-0.707;0;0]
[0;0;0.707	[-0.471;-0.471;0.236;0.707]
[0.67;0.38	[-0.167;-0.167;0.833;-0.5]
[0.368;0.5	[0.5;0.5;0.5;0.5]
[0.56;0.49	[0.5;0.5;0.5;0.5]
[0.67;-0.74	[-0.211;0.789;-0.577;0]
[-0.488;-0.	[-0.289;-0.289;-0.289;0.866]
[0;0;-0.707	[0.789;-0.211;-0.577;0]
[0.546;0.6	[0.789;-0.211;-0.577;0]
[0.743;-0.6	[-0.211;0.789;-0.577;0]
[0;0;-0.707	[0.289;0.289;0.289;-0.866]
[0.388;0.4	[0.5;0.5;0.5;0.5]
[-0.845;0.3	[0.789;-0.211;-0.577;0]
[0.389;0.5	[-0.211;0.789;-0.577;0]
[-0.816;0;0	[0.289;0.289;0.289;-0.866]
[0;0;-0.707	[0.5;0.5;0.5;0.5]
[0;-4.5418	[0.0845509893628813;0.0845509893628811;-0.781474414421557;0.61237243569579
[0.836670	[0.707106781186547;-0.707106781186547;0;0]
[0.836670	[-0.492799279826744;-0.492799279826744;0.373226123957694;0.612372435695794
[-0.397135	[-0.5;-0.5;-0.5;-0.5]
[0.778519	[0.18396242432651;-0.140744645025961;-0.709146118863571;0.665928339563022]
[-1.240126	[-0.137071838177439;-0.758818756867048;0.493196961916072;0.402693633128415
[-0.719758	[0.835086305488583;-0.392918616412182;-0.0621975791931702;-0.3799701098832
[-0.437836	[-0.5;-0.5;-0.5;-0.5]

2435695794]

435695794]

533128415]
010988323]
