# Supplementary material for: Dissecting molecular network structures using a network subgraph approach
Source: PeerJ. 2020 Aug 6;8:e9556. doi: 10.7717/peerj.9556 (PMC7512139; doi:10.7717/peerj.9556)
Supplement: Supplemental Information 8 [file peerj-08-9556-s008.pdf]

pattern id	R	r	a_bar
14	0	-0.3333	0.25
28	0	-0.3333	0.25
30	0	0.25	0.3333
74	0	-0.3333	0.25
76	0	-0.3333	0.25
78	0	-0.5	0.3333
90	0	0.25	0.3333
92	0	-0.5	0.3333
94	0	-0.02857	0.4167
204	0	-0.5	0.3333
206	0	-0.7143	0.4167
222	0	-0.3333	0.5
280	0	-0.3333	0.25
282	0	0.25	0.3333
286	0	0.6571	0.4167
328	0	-0.3333	0.25
330	0	-0.5	0.3333
332	0	0.25	0.3333
334	0	-0.02857	0.4167
344	0	-0.5	0.3333
346	0	-0.02857	0.4167
348	0	-0.02857	0.4167
350	0	0.3333	0.5
390	0	0.25	0.3333
392	0	-0.3333	0.25
394	0	-0.5	0.3333
396	0	0.25	0.3333
398	0	-0.02857	0.4167
404	0	0.25	0.3333
406	0	0.6571	0.4167
408	0	-0.5	0.3333
410	0	-0.02857	0.4167
412	0	-0.02857	0.4167
414	0	0.3333	0.5
454	0	-0.02857	0.4167
456	0	-0.5	0.3333
458	0	-0.7143	0.4167
460	0	-0.02857	0.4167
462	0	-0.3333	0.5
468	0	-0.02857	0.4167
470	0	0.3333	0.5
472	0	-0.7143	0.4167
474	0	-0.3333	0.5
476	0	-0.3333	0.5

478	0	-0.02857	0.5833
856	0	-0.02857	0.4167
858	0	0.3333	0.5
862	0	0.6571	0.5833
904	0	-0.5	0.3333
906	0	-0.7143	0.4167
908	0	-0.02857	0.4167
910	0	-0.3333	0.5
922	0	-0.3333	0.5
924	0	-0.3333	0.5
926	0	-0.02857	0.5833
972	0	0.3333	0.5
974	0	-0.02857	0.5833
990	0	0.25	0.6667
2184	0	-0.3333	0.25
2186	0	-0.5	0.3333
2190	0	-0.7143	0.4167
2202	0	-0.02857	0.4167
2204	0	-0.7143	0.4167
2206	0	-0.3333	0.5
2252	0	-0.7143	0.4167
2254	0	-1	0.5
2270	0	-0.7143	0.5833
2458	0	-0.3333	0.5
2462	0	-0.02857	0.5833
2506	0	-1	0.5
2510	0	-0.7143	0.5833
2524	0	-0.7143	0.5833
2526	0	-0.5	0.6667
3038	0	-0.3333	0.75
4370	0	0.25	0.3333
4374	0	0.6571	0.4167
4382	1	1	0.5
4418	0	-0.5	0.3333
4420	0	0.25	0.3333
4422	0	-0.02857	0.4167
4424	0	0.25	0.3333
4426	0	-0.02857	0.4167
4428	0	0.6571	0.4167
4430	0	0.3333	0.5
4434	0	-0.02857	0.4167
4436	0	-0.02857	0.4167
4438	0	0.3333	0.5
4440	0	-0.02857	0.4167
4442	0	0.3333	0.5

4444	0	0.3333	0.5
4446	0	0.6571	0.5833
4546	0	-0.7143	0.4167
4548	0	-0.02857	0.4167
4550	0	-0.3333	0.5
4556	0	0.3333	0.5
4558	0	-0.02857	0.5833
4562	0	-0.3333	0.5
4564	0	-0.3333	0.5
4566	0	-0.02857	0.5833
4572	0	-0.02857	0.5833
4574	0	0.25	0.6667
4678	0	-0.02857	0.4167
4682	0	0.6571	0.4167
4686	0	0.3333	0.5
4692	0	-0.02857	0.4167
4694	0	0.3333	0.5
4698	1	1	0.5
4700	0	0.3333	0.5
4702	0	0.6571	0.5833
4740	0	-0.5	0.3333
4742	0	-0.7143	0.4167
4748	0	-0.02857	0.4167
4750	0	-0.3333	0.5
4758	0	-0.3333	0.5
4764	0	-0.3333	0.5
4766	0	-0.02857	0.5833
4812	0	0.3333	0.5
4814	0	-0.02857	0.5833
4830	0	0.25	0.6667
4946	0	0.3333	0.5
4950	0	0.6571	0.5833
4952	0	0.3333	0.5
4954	0	0.6571	0.5833
4958	1	1	0.6667
4994	0	-0.7143	0.4167
4998	0	-0.3333	0.5
5002	0	-0.3333	0.5
5004	0	0.3333	0.5
5006	0	-0.02857	0.5833
5010	0	-0.3333	0.5
5012	0	-0.3333	0.5
5014	0	-0.02857	0.5833
5016	0	-0.3333	0.5
5018	0	-0.02857	0.5833

5020	0	-0.02857	0.5833
5022	0	0.25	0.6667
5058	0	-0.3333	0.5
5062	0	-0.02857	0.5833
5064	0	0.3333	0.5
5066	0	-0.02857	0.5833
5068	0	0.6571	0.5833
5070	0	0.25	0.6667
5074	0	-0.02857	0.5833
5076	0	-0.02857	0.5833
5078	0	0.25	0.6667
5080	0	-0.02857	0.5833
5082	0	0.25	0.6667
5084	0	0.25	0.6667
5086	0	0.5556	0.75
6342	0	-1	0.5
6348	0	-0.3333	0.5
6350	0	-0.7143	0.5833
6356	0	-1	0.5
6358	0	-0.7143	0.5833
6364	0	-0.7143	0.5833
6366	0	-0.5	0.6667
6550	0	-0.02857	0.5833
6552	0	-0.3333	0.5
6554	0	-0.02857	0.5833
6558	0	0.25	0.6667
6598	0	-0.7143	0.5833
6602	0	-0.7143	0.5833
6604	0	-0.02857	0.5833
6606	0	-0.5	0.6667
6614	0	-0.5	0.6667
6616	0	-0.7143	0.5833
6618	0	-0.5	0.6667
6620	0	-0.5	0.6667
6622	0	-0.3333	0.75
6854	0	-0.7143	0.5833
6858	0	-0.02857	0.5833
6862	0	-0.5	0.6667
6870	0	-0.5	0.6667
6874	0	0.25	0.6667
6876	0	-0.5	0.6667
6878	0	-0.3333	0.75
7126	0	-0.3333	0.75
7128	0	-0.5	0.6667
7130	0	-0.3333	0.75

7134	0	-0.2	0.8333
13142	0	0.25	0.6667
13146	0	0.25	0.6667
13148	0	0.25	0.6667
13150	0	0.5556	0.75
13260	1	1	0.6667
13262	0	0.5556	0.75
13278	1	1	0.8333
14678	0	-0.5	0.6667
14686	0	-0.3333	0.75
14790	0	-0.5	0.6667
14798	0	-0.3333	0.75
14810	0	-0.3333	0.75
14812	0	-0.3333	0.75
14814	0	-0.2	0.8333
15258	0	-0.3333	0.75
15262	0	-0.2	0.8333
15310	0	-0.2	0.8333
15326	0	-0.09091	0.9167
31710	1	1	1
