# Supplementary material for: Dissecting molecular network structures using a network subgraph approach
Source: PeerJ. 2020 Aug 6;8:e9556. doi: 10.7717/peerj.9556 (PMC7512139; doi:10.7717/peerj.9556)
Supplement: Supplemental Information 9 [file peerj-08-9556-s009.pdf]

ID	E	P	CC=E-N+2P	KC	rank
14	3	3	5	33.80	1
28	3	2	3	35.38	3
30	4	2	4	35.23	2
74	3	2	3	36.32	5
76	3	2	3	36.40	6
78	4	2	4	36.25	4
90	4	2	4	38.16	22
92	4	2	4	38.24	29
94	5	2	5	38.09	18
204	4	2	4	38.26	30
206	5	2	5	38.11	20
222	6	2	6	39.27	66
280	3	1	1	38.17	24
282	4	1	2	38.24	28
286	5	1	3	38.17	24
328	3	1	1	36.71	7
330	4	1	2	36.78	9
332	4	1	2	36.86	10
334	5	1	3	36.71	7
344	4	1	2	38.72	38
346	5	1	3	38.79	40
348	5	1	3	38.87	43
350	6	1	4	38.72	38
390	4	1	2	38.71	37
392	3	1	1	38.49	33
394	4	1	2	38.56	35
396	4	1	2	38.64	36
398	5	1	3	38.49	33
404	4	1	2	39.77	77
406	5	1	3	39.92	83
408	4	1	2	39.70	74
410	5	1	3	39.77	78
412	5	1	3	39.85	80
414	6	1	4	39.70	74
454	5	1	3	39.17	64
456	4	1	2	38.95	48
458	5	1	3	39.02	54
460	5	1	3	39.10	60
462	6	1	4	38.95	48
468	5	1	3	40.29	100
470	6	1	4	40.44	106
472	5	1	3	40.22	94
474	6	1	4	40.29	101
476	6	1	4	40.37	104

478	7	1	5	40.22	94
856	5	1	3	38.86	41
858	6	1	4	38.93	47
862	7	1	5	38.86	41
904	4	1	2	39.41	70
906	5	1	3	39.48	72
908	5	1	3	39.56	73
910	6	1	4	39.41	70
922	6	1	4	41.11	132
924	6	1	4	41.19	133
926	7	1	5	41.04	129
972	6	1	4	39.85	80
974	7	1	5	39.70	74
990	8	1	6	40.54	110
2184	3	1	1	38.07	16
2186	4	1	2	38.15	21
2190	5	1	3	38.07	16
2202	5	1	3	38.97	52
2204	5	1	3	39.05	56
2206	6	1	4	38.90	44
2252	5	1	3	40.07	88
2254	6	1	4	39.92	84
2270	7	1	5	41.06	130
2458	6	1	4	39.98	86
2462	7	1	5	39.91	82
2506	6	1	4	41.39	138
2510	7	1	5	41.32	135
2524	7	1	5	42.70	189
2526	8	1	6	42.55	186
3038	9	1	7	42.79	191
4370	4	0	0	37.95	15
4374	5	0	1	38.10	19
4382	6	0	2	37.88	14
4418	4	0	0	40.25	97
4420	4	0	0	40.25	97
4422	5	0	1	40.39	105
4424	4	0	0	40.17	92
4426	5	0	1	40.25	99
4428	5	0	1	40.33	103
4430	6	0	2	40.17	92
4434	5	0	1	38.97	50
4436	5	0	1	38.97	50
4438	6	0	2	39.12	61
4440	5	0	1	38.90	44
4442	6	0	2	38.97	52

4444	6	0	2	39.05	56
4446	7	0	3	38.90	44
4546	5	0	1	41.77	150
4548	5	0	1	41.77	150
4550	6	0	2	41.92	161
4556	6	0	2	41.85	157
4558	7	0	3	41.70	143
4562	6	0	2	40.15	90
4564	6	0	2	40.15	90
4566	7	0	3	40.30	102
4572	7	0	3	40.23	96
4574	8	0	4	40.08	89
4678	5	0	1	37.67	13
4682	5	0	1	37.53	12
4686	6	0	2	37.45	11
4692	5	0	1	38.23	26
4694	6	0	2	38.38	32
4698	6	0	2	38.24	27
4700	6	0	2	38.32	31
4702	7	0	3	38.16	23
4740	4	0	0	39.23	65
4742	5	0	1	39.38	69
4748	5	0	1	39.32	68
4750	6	0	2	39.16	63
4758	6	0	2	40.02	87
4764	6	0	2	39.95	85
4766	7	0	3	39.80	79
4812	6	0	2	41.36	137
4814	7	0	3	41.21	134
4830	8	0	4	40.86	123
4946	6	0	2	40.57	112
4950	7	0	3	40.72	120
4952	6	0	2	40.50	108
4954	7	0	3	40.57	113
4958	8	0	4	40.50	108
4994	5	0	1	40.62	115
4998	6	0	2	40.77	121
5002	6	0	2	40.62	116
5004	6	0	2	40.70	118
5006	7	0	3	40.55	111
5010	6	0	2	41.80	152
5012	6	0	2	41.80	152
5014	7	0	3	41.95	162
5016	6	0	2	41.73	145
5018	7	0	3	41.80	155

5020	7	0	3	41.88	158
5022	8	0	4	41.73	145
5058	6	0	2	41.80	152
5062	7	0	3	41.95	162
5064	6	0	2	41.73	145
5066	7	0	3	41.80	155
5068	7	0	3	41.88	158
5070	8	0	4	41.73	145
5074	7	0	3	42.37	178
5076	7	0	3	42.37	178
5078	8	0	4	42.52	185
5080	7	0	3	42.30	174
5082	8	0	4	42.37	180
5084	8	0	4	42.45	182
5086	9	0	5	42.30	174
6342	6	0	2	40.66	117
6348	6	0	2	40.60	114
6350	7	0	3	40.44	107
6356	6	0	2	42.15	170
6358	7	0	3	42.30	176
6364	7	0	3	42.23	172
6366	8	0	4	42.08	167
6550	7	0	3	39.28	67
6552	6	0	2	39.06	58
6554	7	0	3	39.13	62
6558	8	0	4	39.06	58
6598	7	0	3	42.11	168
6602	7	0	3	41.97	165
6604	7	0	3	42.05	166
6606	8	0	4	41.89	160
6614	8	0	4	41.72	144
6616	7	0	3	41.50	139
6618	8	0	4	41.57	141
6620	8	0	4	41.65	142
6622	9	0	5	41.50	139
6854	7	0	3	42.47	183
6858	7	0	3	42.32	177
6862	8	0	4	42.25	173
6870	8	0	4	40.93	126
6874	8	0	4	40.78	122
6876	8	0	4	40.86	124
6878	9	0	5	40.71	119
7126	9	0	5	43.15	195
7128	8	0	4	42.93	192
7130	9	0	5	43.00	194

7134	10	0	6	42.93	192
13142	8	0	4	41.10	131
13146	8	0	4	40.95	127
13148	8	0	4	41.03	128
13150	9	0	5	40.88	125
13260	8	0	4	43.32	197
13262	9	0	5	43.16	196
13278	10	0	6	42.50	184
14678	8	0	4	41.95	162
14686	9	0	5	41.73	145
14790	8	0	4	42.38	181
14798	9	0	5	42.16	171
14810	9	0	5	42.62	188
14812	9	0	5	42.70	189
14814	10	0	6	42.55	186
15258	9	0	5	43.74	199
15262	10	0	6	43.67	198
15310	10	0	6	42.12	169
15326	11	0	7	41.32	135
31710	12	0	8	39.04	55
