# Supplementary material for: Dissecting molecular network structures using a network subgraph approach
Source: PeerJ. 2020 Aug 6;8:e9556. doi: 10.7717/peerj.9556 (PMC7512139; doi:10.7717/peerj.9556)
Supplement: Supplemental Information 10 — A pseudo-count was added if the number of embedded is zero, because the odds ratio is not well-defined. Odds ratio and total number of genes are listed in the last column. An odds ratio greater than one implies that driver genes are enriched in subgraph module. [file peerj-08-9556-s010.pdf]

Supplementary Table 9. The results of driver genes embedded in subgraph module and non-subgraph module. A pseudo-count was added if the number of embedded is zero, because the odds ratio is not well-defined. Odds ratio and total number of genes are listed in the last column. An odds ratio greater than one implies that driver genes are enriched in subgraph module.

Cancer Networks		Genes embedded in subgraph	Genes not embedded in subgraph	Odds ratio/total
Acute myeloid leukemia [hsa05221]	Driver gene	18	0+1 (pseudo-count)	0.720
	Non-driver gene	25	0+1 (pseudo-count)	
	Total	43	2	45
Basal cell carcinoma [hsa05217]	Driver gene	8	1	0.615
	Non-driver gene	13	1	
	Total	21	2	23
Breast cancer [hsa05224]	Driver gene	23	4	1.643
	Non-driver gene	28	8	
	Total	51	12	63
Choline metabolism in cancer [hsa05231]	Driver gene	10	2	2.353
	Non-driver gene	17	8	
	Total	27	10	37
Chronic myeloid leukemia [hsa05220]	Driver gene	16	5	0.762
	Non-driver gene	21	5	
	Total	37	10	47
Colorectal cancer [hsa05210]	Driver gene	16	5	0.914
	Non-driver gene	21	6	
	Total	37	11	48
Endometrial cancer [hsa05213]	Driver gene	14	5	0.560
	Non-driver gene	15	3	
	Total	29	8	37
Gastric cancer [hsa05226]	Driver gene	21	7	1.355
	Non-driver gene	31	14	
	Total	52	21	73
Glioma [hsa05214]	Driver gene	13	4	0.181
	Non-driver gene	18	1	
	Total	31	5	36
Hepatocellular carcinoma [hsa05225]	Driver gene	21	11	1.074
	Non-driver gene	32	18	
	Total	53	29	82
Melanoma [hsa05218]	Driver gene	15	3	0.500
	Non-driver gene	10	0+1 (pseudo-count)	
	Total	25	4	29
Non-small cell lung cancer [hsa05223]	Driver gene	18	2	2.500
	Non-driver gene	18	5	
	Total	36	7	43
Pancreatic cancer [hsa05212]	Driver gene	19	2	0.704
	Non-driver gene	27	2	
	Total	46	4	50
Pathways in cancer [hsa05200]	Driver gene	52	2	2.187
	Non-driver gene	107	9	

	Total	159	11	170
Prostate cancer [hsa05215]	Driver gene	16	7	1.143
	Non-driver gene	20	10	
	Total	36	17	53
Renal cell carcinoma [hsa05211]	Driver gene	11	6	0.509
	Non-driver gene	18	5	
	Total	29	11	40
Small cell lung cancer [hsa05222]	Driver gene	8	6	0.286
	Non-driver gene	28	6	
	Total	36	12	48

STN		Genes embedded in subgraph	Genes not embedded in subgraph	Odds ratio/total
Adipocytokine signaling pathway [hsa04920]	Driver gene	4	0+1 (pseudo-count)	0.414
	Non-driver gene	29	3	
	Total	33	4	37
AMPK signaling pathway [hsa04152]	Driver gene	4	4	0.262
	Non-driver gene	42	11	
	Total	46	15	61
Apelin signaling pathway [hsa04371]	Driver gene	8	1	1.436
	Non-driver gene	39	7	
	Total	47	8	55
B cell receptor signaling pathway [hsa04662]	Driver gene	13	3	1.857
	Non-driver gene	21	9	
	Total	34	12	46
Calcium signaling pathway [hsa04020]	Driver gene	5	0+1 (pseudo-count)	5.263
	Non-driver gene	19	20	
	Total	24	21	45
cAMP signaling pathway [hsa04024]	Driver gene	12	1	1.309
	Non-driver gene	55	6	
	Total	67	7	74
cGMP-PKG signaling pathway [hsa04022]	Driver gene	5	0+1 (pseudo-count)	1.136
	Non-driver gene	44	10	
	Total	49	11	60
Chemokine signaling pathway [hsa04062]	Driver gene	12	0+1 (pseudo-count)	1.000
	Non-driver gene	36	3	
	Total	48	4	52
C-type lectin receptor signaling pathway [hsa04625]	Driver gene	18	0+1 (pseudo-count)	1.000
	Non-driver gene	54	3	
	Total	72	4	76
ErbB signaling pathway [hsa04012]	Driver gene	16	1	0.444
	Non-driver gene	36	0+1 (pseudo-count)	
	Total	52	2	54
Estrogen signaling pathway [hsa04915]	Driver gene	11	1	1.737
	Non-driver gene	19	3	
	Total	30	4	34
	Driver gene	10	0+1 (pseudo-count)	5.000

Fc epsilon RI signaling pathway [hsa04664]	Non-driver gene	20	10	
	Total	30	11	41
FoxO signaling pathway [hsa04068]	Driver gene	15	2	0.708
	Non-driver gene	53	5	
	Total	68	7	74
Glucagon signaling pathway [hsa04922]	Driver gene	4	1	1.926
	Non-driver gene	27	13	
	Total	31	14	45
GnRH signaling pathway [hsa04912]	Driver gene	11	0+1 (pseudo-count)	1.760
	Non-driver gene	25	4	
	Total	36	5	41
Hedgehog signaling pathway [hsa04340]	Driver gene	6	0+1 (pseudo-count)	0.353
	Non-driver gene	17	0+1 (pseudo-count)	
	Total	23	2	25
HIF-1 signaling pathway [hsa04066]	Driver gene	8	4	0.273
	Non-driver gene	44	6	
	Total	52	10	62
Hippo signaling pathway [hsa04390]	Driver gene	8	5	0.4
	Non-driver gene	52	13	
	Total	60	18	78
Insulin signaling pathway [hsa04910]	Driver gene	9	2	0.587
	Non-driver gene	46	6	
	Total	55	8	63
Jak-STAT signaling pathway [hsa04630]	Driver gene	11	0+1 (pseudo-count)	0.500
	Non-driver gene	22	1	
	Total	33	2	35
MAPK signaling pathway [hsa04010]	Driver gene	21	0+1 (pseudo-count)	0.457
	Non-driver gene	92	2	
	Total	113	3	116
mTOR signaling pathway [hsa04150]	Driver gene	10	3	0.580
	Non-driver gene	46	8	
	Total	56	11	67
Neurotrophin signaling pathway [hsa04722]	Driver gene	21	1	0.840
	Non-driver gene	50	2	
	Total	71	3	74
NF-kappa B signaling pathway [hsa04064]	Driver gene	8	6	0.538
	Non-driver gene	62	25	
	Total	70	31	101
NOD-like receptor signaling pathway [hsa04621]	Driver gene	9	0+1 (pseudo-count)	1.862
	Non-driver gene	87	18	
	Total	96	19	115
Notch signaling pathway [hsa04330]	Driver gene	1	2	0.250
	Non-driver gene	16	8	
	Total	17	10	27
Oxytocin signaling pathway [hsa04921]	Driver gene	11	1	1.571
	Non-driver gene	35	5	
	Total	46	6	52

p53 signaling pathway [hsa04115]	Driver gene	14	3	0.491
	Non-driver gene	38	4	
	Total	52	7	59
Phosphatidylinositol signaling system [hsa04070]	Driver gene	2	0+1 (pseudo-count)	0.240
	Non-driver gene	25	3	
	Total	27	4	31
Phospholipase D signaling pathway [hsa04072]	Driver gene	12	1	2.710
	Non-driver gene	31	7	
	Total	43	8	51
PI3K-Akt signaling pathway [hsa04151]	Driver gene	20	6	1.020
	Non-driver gene	49	15	
	Total	69	21	90
PPAR signaling pathway [hsa03320]	Driver gene	1	0+1 (pseudo-count)	0.075
	Non-driver gene	53	4	
	Total	54	5	58
Prolactin signaling pathway [hsa04917]	Driver gene	11	0+1 (pseudo-count)	0.407
	Non-driver gene	27	1	
	Total	38	2	40
Rap1 signaling pathway [hsa04015]	Driver gene	13	1	3.434
	Non-driver gene	53	14	
	Total	66	15	81
Ras signaling pathway [hsa04014]	Driver gene	15	0+1 (pseudo-count)	0.900
	Non-driver gene	50	3	
	Total	65	4	69
Relaxin signaling pathway [hsa04926]	Driver gene	11	1	1.447
	Non-driver gene	38	5	
	Total	49	6	55
RIG-I-like receptor signaling pathway [hsa04622]	Driver gene	3	2	1.050
	Non-driver gene	30	21	
	Total	33	23	56
Sphingolipid signaling pathway [hsa04071]	Driver gene	12	0+1 (pseudo-count)	3.636
	Non-driver gene	33	10	
	Total	45	11	56
T cell receptor signaling pathway [hsa04660]	Driver gene	14	3	1.640
	Non-driver gene	37	13	
	Total	51	16	67
TGF-beta signaling pathway [hsa04350]	Driver gene	8	2	1.517
	Non-driver gene	29	11	
	Total	37	13	50
Thyroid hormone signaling pathway [hsa04919]	Driver gene	18	3	2.516
	Non-driver gene	31	13	
	Total	49	16	65
TNF signaling pathway [hsa04668]	Driver gene	6	3	1.778
	Non-driver gene	36	32	
	Total	42	35	77
	Driver gene	7	0+1 (pseudo-count)	0.831
	Non-driver gene	59	7	

Toll-like receptor signaling pathway [hsa04620]	Total	66	8	74
VEGF signaling pathway [hsa04370]	Driver gene	10	0+1 (pseudo-count)	0.556
	Non-driver gene	18	0+1 (pseudo-count)	
	Total	28	2	30
Wnt signaling pathway [hsa04310]	Driver gene	13	1	0.491
	Non-driver gene	53	2	
	Total	66	3	69

Cellular Processes		Genes embedded in subgraph	Genes not embedded in subgraph	Odds ratio/total
Adherens junction [hsa04520]	Driver gene	13	3	0.699
	Non-driver gene	31	5	
	Total	44	8	52
Apoptosis [hsa04210]	Driver gene	17	1	2.194
	Non-driver gene	62	8	
	Total	79	9	88
Cell cycle [hsa04110]	Driver gene	12	5	1.670
	Non-driver gene	46	32	
	Total	58	37	95
Cellular senescence [hsa04218]	Driver gene	19	5	1.086
	Non-driver gene	49	14	
	Total	68	19	87
Focal adhesion [hsa04510]	Driver gene	15	0+1 (pseudo-count)	0.333
	Non-driver gene	45	1	
	Total	60	2	62
Gap junction [hsa04540]	Driver gene	9	0+1 (pseudo-count)	0.360
	Non-driver gene	25	0+1 (pseudo-count)	
	Total	34	2	36
Necroptosis [hsa04217]	Driver gene	8	0+1 (pseudo-count)	1.098
	Non-driver gene	51	7	
	Total	59	8	67
Regulation of actin cytoskeleton [hsa04810]	Driver gene	11	0+1 (pseudo-count)	2.933
	Non-driver gene	45	12	
	Total	56	13	69
Signaling pathways regulating pluripotency of stem cells [hsa04550]	Driver gene	19	4	5.700
	Non-driver gene	20	24	
	Total	39	28	67
