# Supplementary material for: Dissecting molecular network structures using a network subgraph approach
Source: PeerJ. 2020 Aug 6;8:e9556. doi: 10.7717/peerj.9556 (PMC7512139; doi:10.7717/peerj.9556)
Supplement: Supplemental Information 11 — Table R1 summarized the results of subgraphs associated with two to six edges, the in-degree and out-degree information. Table R2 summarized the theoretical results of the statistical average and standard deviation of the graph energies for the 3-node subgraphs. Table R3 summarized the theoretical results of the statistical average and standard deviation of the reciprocity for the 3-node subgraphs after using the randomization concept. [file peerj-08-9556-s011.pdf]

We stated in section 1.1 that our analysis does not consider randomized versions of the studied networks. This concept also apply to both 3-node subgraphs and 4-node subgraphs analysis. Essentially, there is no need to compute the statistical average and standard deviation of the graph energies and reciprocity.

In case of using the randomization approach, one begins by considering a set of subgraphs with the same number of edges (*e*), then the edges are randomly assigned to connect the nodes. in which the total number of in-degree and out-degree of a subgraph are kept the same as the original subgraph. Given the randomized version of the subgraph, one computes the nine graph energies and reciprocity of the randomized version. The randomization process repeated; hence, the statistical average and standard deviation can be obtained.

From Table 4 (in the manuscript), we found that there are three 3-node subgraphs with two edges, that is, subgraphs: 'id_6', 'id_12' and 'id_36'. Table R1 summarized the results of subgraphs associated with two to six edges, the in-degree and out-degree information. Now, assuming that we pick the 3-node subgraph 'id_6' and randomly reassign the two edges in which the total number of in-degree and out-degree are kept the same. Theoretically, we have one third of the chance getting one of the three subgraphs: 'id_6', 'id_12' and 'id_36'. Then, the statistical average and standard deviation of the graph energies and reciprocity can be obtained. In case of subgraphs with three and four edges, we have one fourth of the chance getting one of the four subgraphs.

Table R1. The results of the subgraphs' IDs associated with two to six edges.

Number of edges, *e*	Subgraph ID	in-degree	Out-degree
2	6, 12, 36	2	2
3	14, 38, 74, 98	3	3
4	476, 78, 102, 108	4	4
5	110	5	5
6	238	6	6

Table R2 summarized the theoretical results of the statistical average and standard deviation of the graph energies for the 3-node subgraphs**.** Since there is only one possible pattern for subgraphs with five and six edges, therefore, the statistical average and standard deviation for subgraphs 'id_110' and 'id_238' are not available. From Table R2, we noted that the degree of degeneracy for the graph energies increase. For

instance, a total of five and nine LQ_t values are associated with and without the randomize process respectively. In other words, the discrimination power decreases after randomization.

Table R2. The theoretical results of the mean and standard deviation of the nine graph energies for the 3-node subgraphs after using the randomization concept.

ID	E	LE	QE	AA_t	LL_t	QQ_t	AL_t	AQ_t	LQ_t
6	0.00±0.00	2.67±0.00	2.67±0.00	1.61±0.34	4.33±0.01	4.33±0.01	1.61±0.34	1.61±0.34	3.86±0.06
12	0.00±0.00	2.67±0.00	2.67±0.00	1.61±0.34	4.33±0.01	4.33±0.01	1.61±0.34	1.61±0.34	3.86±0.06
14	1,75±1.26	4.07±0.15	4.12±0.23	2.52±0.33	6.24±0.13	6.26±0.16	3.22±0.71	3.18±0.84	5.73±0.32
36	0.00±0.00	2.67±0.00	2.67±0.00	1.61±0.34	4.33±0.01	4.33±0.01	1.61±0.34	1.61±0.34	3.86±0.06
38	1,75±1.26	4.07±0.15	4.12±0.23	2.52±0.33	6.24±0.13	6.26±0.16	3.22±0.71	3.18±0.84	5.73±0.32
46	2.47±0.55	5.33±0.00	5.39±0.12	2.88±0.24	8.16±0.11	8.14±0.10	3.16 ± 1.38	4.15 ± 0.58	7.51±0.15
74	1,75±1.26	4.07±0.15	4.12±0.23	2.52±0.33	6.24±0.13	6.26±0.16	3.22±0.71	3.18±0.84	5.73±0.32
78	2.47±0.55	5.33±0.00	5.39±0.12	2.88±0.24	8.16±0.11	8.14±0.10	3.16 ± 1.38	4.15 ± 0.58	7.51±0.15
98	1,75±1.26	4.07±0.15	4.12±0.23	2.52±0.33	6.24±0.13	6.26±0.16	3.22±0.71	3.18±0.84	5.73±0.32
102	2.47±0.55	5.33±0.00	5.39±0.12	2.88±0.24	8.16±0.11	8.14±0.10	3.16 ± 1.38	4.15 ± 0.58	7.51±0.15
108	2.47±0.55	5.33±0.00	5.39±0.12	2.88±0.24	8.16±0.11	8.14±0.10	3.16 ± 1.38	4.15 ± 0.58	7.51±0.15
110	3.24	6.67	6.72	3.49	10.09	10.09	5.38	5.70	9.40
238	4.00	8.00	8.00	4.00	12.00	12.00	6.47	6.93	11.21

Table R3 summarized the theoretical results of the statistical average and standard deviation of the reciprocity for the 3-node subgraphs after using the randomization concept. Using the randomization approach, it was found that the number of distinct *R* is four, which is two times more than the non-randomized results. For reciprocity *r*, the number of distinct *r* is five, which is the same as the non-randomized results.

Table R3. The theoretical results of the mean and standard deviation of the traditional reciprocity (*R*), reciprocity (*r*) for the 3-node subgraphs after using the randomization concept.

ID	R	r	edge (e)	average reciprocity, $\bar{a}$
6	0.00±0.00	-0.50±0.00	2	1/3
12	0.00±0.00	-0.50±0.00	2	1/3
14	0.00±0.00	-0.33±0.77	3	0.5
36	0.00±0.00	-0.50±0.00	2	1/3
38	0.00±0.00	-0.33±0.77	3	0.5
46	0.25±0.50	-0.13±0.75	4	2/3
74	0.00±0.00	-0.33±0.77	3	0.5
78	0.25±0.50	-0.13±0.75	4	2/3
98	0.00±0.00	-0.33±0.77	3	0.5
102	0.25±0.50	-0.13±0.75	4	2/3
108	0.25±0.50	-0.13±0.75	4	2/3
110	0	-0.2	5	5/6
238	1	1	6	1
