# Supplementary material for: Dissecting molecular network structures using a network subgraph approach
Source: PeerJ. 2020 Aug 6;8:e9556. doi: 10.7717/peerj.9556 (PMC7512139; doi:10.7717/peerj.9556)
Supplement: Supplemental Information 12 [file peerj-08-9556-s012.pdf]

3.2 Signal transduction
04014 Ras signaling pathway
04015 Rap1 signaling pathway
04010 MAPK signaling pathway
04012 ErbB signaling pathway
04310 Wnt signaling pathway
04330 Notch signaling pathway
04340 Hedgehog signaling pathway
04350 TGF-beta signaling pathway
04390 Hippo signaling pathway
04370 VEGF signaling pathway
04371 Apelin signaling pathway
04630 NF-kappa B signaling pathway
04668 TNF signaling pathway
04066 HIF-1 signaling pathway
04068 FoxO signaling pathway
04020 Calcium signaling pathway
04070 Phosphatidylinositol signaling system
04072 Phospholipase D signaling pathway
04071 Sphingolipid signaling pathway
04024 cAMP signaling pathway
04022 cGMP-PKG signaling pathway
04151 PI3K-Akt signaling pathway
04152 AMPK signaling pathway
04150 mTOR signaling pathway

4.2 Cell growth and death
04110 Cell cycle
04210 Apoptosis
04217 Necroptosis
04115 p53 signaling pathway
04218 Cellular senescence

4.3 Cellular community – eukaryotes
04510 Focal adhesion
04520 Adherens junction
04540 Gap junction
04550 Signaling pathways regulating pluripotency of stem cells

4.5 Cell motility
04810 Regulation of actin cytoskeleton

5.1 Immune system
04620 Toll-like receptor signaling pathway
04621 NOD-like receptor signaling pathway
04622 RIG-I-like receptor signaling pathway
04625 C-type lectin receptor signaling pathway
04660 T cell receptor signaling pathway
04662 B cell receptor signaling pathway
04664 Fc epsilon RI signaling pathway
04062 Chemokine signaling pathway

5.2 Endocrine system
04910 Insulin signaling pathway
04922 Glucagon signaling pathway
04920 Adipocytokine signaling pathway
03320 PPAR signaling pathway
04912 GnRH signaling pathway
04915 Estrogen signaling pathway
04921 Oxytocin signaling pathway
04917 Prolactin signaling pathway
04926 Relaxin signaling pathway
04919 Thyroid hormone signaling pathway

5.6 Nervous system
04722 Neurotrophin signaling pathway

6.1 Cancer: overview
05200 Pathways in cancer
05231 Choline metabolism in cancer

6.2 Cancer: specific types
05210 Colorectal cancer
05212 Pancreatic cancer
05225 Hepatocellular carcinoma
05226 Gastric cancer
05214 Glioma
05221 Acute myeloid leukemia
05220 Chronic myeloid leukemia
05217 Basal cell carcinoma
05218 Melanoma
05211 Renal cell carcinoma
05215 Prostate cancer
05213 Endometrial cancer
05224 Breast cancer
05222 Small cell lung cancer
05223 Non-small cell lung cancer
